# Fingerprinting Uranium Oxides with Electron Energy Loss Spectroscopy Supported by Theoretical Computations

**DOI:** 10.1021/acs.jpca.5c07789

**Published:** 2026-03-04

**Authors:** Jacopo Carbone, Barbora Bártová, Thomas La Grange, Katharina Reinhold, Gregory Leinders, Pau Torruella, Cécile Hébert, Michel Sassi, Rizlan Bernier-Latmani, Kevin M. Rosso

**Affiliations:** † Environmental Microbiology Laboratory, École Polytechnique Fédérale de Lausanne (EPFL), Lausanne CH-1015, Switzerland; ‡ Physical and Computational Sciences Directorate, 6865 Pacific Northwest National Laboratory, Richland, Washington 99354, United States; ¶ School of Basic Sciences, Institute of Physics, Laboratory for Ultrafast Microscopy and Electron Scattering, École Polytechnique Fédérale de Lausanne, Lausanne CH-1015, Switzerland; § Institute for Nuclear Energy Technology, Belgian Nuclear Research Centre (SCK CEN), Boeretang 190, Mol 2400, Belgium; ¥ Electron Spectrometry and Microscopy Laboratory, 27218 EPFL, Lausanne CH-1015, Switzerland

## Abstract

Uranium oxides occur in a variety of phases that differ in their crystal structure and uranium oxidation states. Electron energy loss spectroscopy (EELS) is one of the few techniques that has sufficient spatial resolution and sensitivity to electronic structure to distinguish among phases at the nanoscale. However, beam-sensitive materials, such as uranium oxides, are subject to spectral modification due to interactions with the electron beam. Therefore, theory support is essential to reliably exclude the impact of beam damage and generate true reference data sets. Here, we use a comparison of theoretical and experimental spectra to probe the impact of beam damage on the O *K*-edge and U *N*-edge (*N*_6,7_ and *N*_4,5_) EELS spectra of various single-valent and mixed-valence uranium oxide bulk phases. Using a low-dose experimental setup, we show that the *K*-edge theoretical spectra are in excellent agreement with experiment for both peak positions and relative intensities of respective peaks. In contrast, U *N*-edge features are less distinguishing due to the partially localized nature of the U 5f orbitals and overlapping multiplet and spin–orbit coupling effects. This work demonstrates that O *K*-edge EELS is sufficiently diagnostic to distinguish a wide range of uranium oxides and that the experimental approach used here minimizes beam damage and allows valence state discrimination across the U­(IV), U­(V), and U­(VI) series. When combined with imaging modes available in electron microscopy, this work enables a detailed investigation and characterization of uranium redox transformations at the nanoscale.

## Graphical abstract

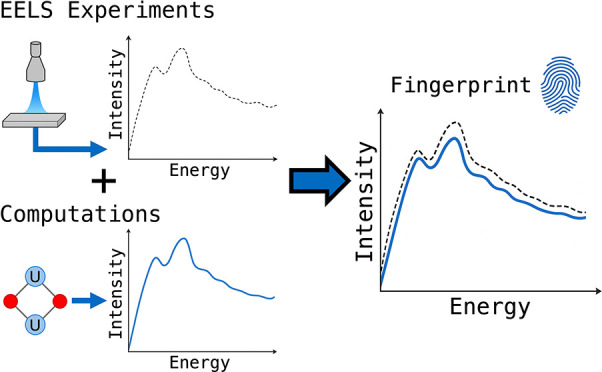

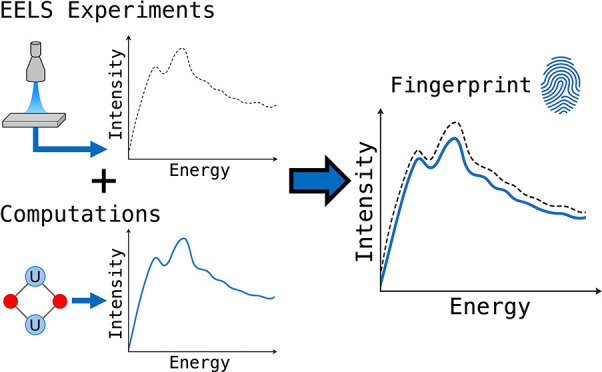

## Introduction

Understanding the fundamental physical phenomena within materials requires precise microstructural characterization across relevant scales. Advanced analytical techniques, such as electron energy loss spectroscopy (EELS), provide insights into materials at the nanoscale, enabling high-resolution spatial characterization. EELS is usually performed via transmission electron microscopy (TEM) working in scanning TEM (STEM) mode and uses inelastic electron interactions within samples to probe electronic excitations, bonding environments, and local chemical properties with nanometer-scale spatial resolution.^1^ EELS integrated with TEM achieves exceptionally high spatial resolution, reaching approximately 0.1 nm.^2^ The typical energy resolution is around 1 eV, but it can be significantly enhanced to about 0.1 eV with the use of an electron-beam monochromator.^3–5^

In the EELS process, high-energy electrons (60–300 kV) interact with the sample either elastically, without energy loss, or inelastically, where energy is transferred to the material. The latter interactions are useful for investigating electronic transitions, ranging from low-energy phenomena, such as plasmons or phonons, to high-energy core-level excitations.^6^ Thus, EELS measurements are widely used in materials science to probe the electronic structure, bonding, and chemical composition of materials with high spatial and energy resolution. In particular, it allows the study of oxidation states, chemical environments, bonding variations, and defects at the nanoscale for material characterization.^7^

EELS is well-suited for analyzing many materials, including oxides, semiconductors, nanostructures, and energy materials. Despite its versatility, EELS analysis is not without challenges. The potential for beam damage is a salient concern especially for thin materials subject to redox reactions.^8^ Sample thickness plays a significant role in spectral quality, as increasing thickness leads to multiple scattering events that reduce the signal-to-noise (SNR) ratio and self-convolve the spectrum, complicating the identification of fine spectral features.^9^ Accurate interpretation of EELS data relies on comparing experimental results to known spectral characteristics including edge positions, fine structures, and energy-loss characteristics. For many materials, particularly newly synthesized compounds and nanostructures, such comprehensive reference data sets are incomplete or missing, hampering a straightforward interpretation of observed features.

Overcoming this limitation requires that the experimental data of reference materials be supported by theoretical calculations. Computations based on first-principles methods can provide a fairly complete description of such complex electronic scattering phenomena.^10^ However, model accuracy is typically directly tied to computational expense. Therefore, the present study is motivated by the need to address both experimental and computational limitations by (a) benchmarking computational methods for EELS spectral prediction for complex materials, and (b) designing experimental conditions that preclude significant beam damage to sensitive reference materials.

Several computational codes are available to model EELS, some of which are also capable of computing X-ray absorption near edge structure (XANES) spectra, each offering distinct methods and approximations to calculate the excited state electronic structure (see Text S1 for a summary). Theoretical XANES spectra can be effectively compared to measured EELS spectra within the dipole approximation, as both techniques probe the same unoccupied electronic states.^11^ The extent of correlation between theory and experiment, particularly for fine spectroscopic details, determines the ability to accurately and comprehensively describe the chemical, crystallographic, and electronic structures of target materials.

Here, we compare theoretical predictions versus experimentally measured EELS spectra for uranium (U) oxides as model systems to establish a set of references for U oxides of varying valence states and to demonstrate the combined potential of EELS measurements and calculations in fingerprinting U oxide valence. Ultimately, this study aims to set the stage for leveraging the spatial resolution of EELS and characterizing the U valence state at the nanoscale. Metal oxides, particularly those of actinides, are particularly challenging due to their intricate electronic structures. EELS has been used to unravel the 5f occupancy of actinides at the micro- and nanoscale^12–17^ but has not been successfully applied to the discrimination among valence states, likely due to the absence of standard spectra. In the present study, the spectral characteristics of six pristine U oxide standards were studied with high accuracy: the single valent compounds U­(IV)­O_2_, KU­(V)­O_2_, and BaU­(VI)­O_4_ and the mixed valence compounds U_4_O_9_[2U­(IV); 2U­(V)], U_3_O_7_[U­(IV); 2U­(V)], and U_3_O_8_[2U­(V); U­(VI)]. Specifically, using EELS, the oxygen (O) *K*-edge and U *N*_6,7_-edge and *N*_4,5_-edge spectra of individual U oxide nanoparticles were analyzed, allowing for the characterization of U valence states at the nanoscale. All theoretical XANES spectra were generated using the FDMNES code^10^ within the Muffin-Tin approximation, a choice that optimized the balance between accuracy and computational cost. Distinct edges, the O *K*-edge and the U *N*-edges, were calculated using an excited core-hole self-consistent field (SCF_exc_) approach for the former and time-dependent density functional theory (TDDFT) for the latters. These combined approaches were chosen to ensure an accurate theoretical description of ligand charge transfer effects (SCF_exc_ for the O *K*-edge) and localized f-electron excitations (TDDFT for the U *N*-edges) in U oxide phases.

The experimentally measured EELS spectra were collected at two electron doses to evaluate the impact of the beam on the spectral signal. These were subsequently compared with theoretical XANES simulations. Overall, our results show that at the lowest electron dose achievable, the O *K*-edge provides distinct spectral signatures that reliably differentiate U oxidation states, suggesting minimal beam damage and reflecting the hybridization of O with U orbitals and its sensitivity to local bonding environments. Therefore, it is an effective diagnostic tool for fingerprinting the oxidation states of U in U oxide phases. In contrast, the U *N*_6,7_ and U *N*_4,5_ edges are governed by strong spin–orbit coupling and electron correlation effects intrinsic to the 5f elements and are less diagnostic of U oxidation states. In this work, our emphasis is on establishing and validating EELS spectral standards suitable for fingerprinting U oxidation states in U oxides. Accordingly, we concentrate on reproducing the dominant spectral characteristics relevant to oxidation state discrimination. A full electronic structure assignment of each spectral feature, which would necessitate extensive multiplet, ligand-field, and many-body analysis of the U 5f/6d–O 2p manifold, is beyond the present scope. Furthermore, the overall good agreement obtained between theory and experiments provides a strong basis for future work focusing on the identification of U oxide phases within mixed phase products that typify, for example, environmentally relevant redox transitions of U.

## Materials and Methods

### Computational Details

O *K*-edge and U *N*-edge *ab initio* calculations were performed using the FDMNES code. The calculations employed the MST approach within the Muffin-Tin approximation. Real Hedin–Lundqvist potentials were used to model the exchange-correlation.^18^ For the U *N*_4,5_ and *N*_6,7_-edges, TDDFT calculations as implemented in FDMNES were performed.^19^ Dipoles, quadrupoles, core–hole, and spin–orbit coupling contributions and a fully relativistic treatment were considered. The LSDA + *U* formalism was also applied to the investigated U oxides.^20^ As described by Dudarev et al., this formalism was used for the U atoms to correct the poor description of the Coulomb repulsion of the 5f electrons in standard DFT. The Hubbard parameter *U*, describing the Coulomb interaction, was fixed to 4.5 eV, while the screened exchange energy, *J*, was fixed to 0.54 eV (*U*_eff_ = *U* – *J* = 3.96 eV).^21^ Since the O *K*-edge and U *N*-edges probe fundamentally different aspects of the electronic structure, a single computational approach cannot accurately describe both. The SCF_exc_ approach was used to investigate the local electronic structure and the O coordination environment. Unlike conventional SCF calculations, where the core–hole is introduced after the self-consistent cycle, assuming a neutral cluster, SCF_exc_ explicitly includes the core–hole during the SCF process. This approach accounts for charge redistribution from ligands to the absorbing atom, which is crucial in many insulating and highly covalent systems.^10^ On the other hand, capturing the U environment is challenging, primarily due to the strong correlations among its 5f electrons. Standard DFT often struggles to describe its hybridization with ligand orbitals. TDDFT, implemented in the FDMNES code, improves this by introducing a dynamic response function that accounts for electron–electron interactions, modifying the transition matrix elements, and better representing screening effects.^22^ FDMNES is a real-space symmetrized code that directly accounts for the spatial arrangement of atoms. Whether applied to an isolated molecule or to a periodic crystal structure, the code requires the local environment of the absorbing atom to be modeled as a cluster with the target atom at the center. This cluster is defined by a sphere with a radius that directly influences the accuracy of the calculation. Thus, a large cluster radius ensures that the calculations adequately account for the interactions between the absorbing atom and its neighboring atoms. U oxides exhibit an intricate electronic configuration that allows a variety of chemical interactions, e.g., the formation of both ionic and covalent bonds.^23^ The near-degeneracy of the U 5f, 6d, and 7s orbitals provides a large pool of valence electrons that can participate in bonding, especially with the O 2p orbital.^24^ Potentials and Fermi energies were determined independently using a 7 Å radius and applied consistently across all oxides. Table 1 shows the number of atoms within a 7 Å cluster for various U oxides. This radius provides a good compromise between the accuracy of the spectral features and computational cost. For O *K*-edge and U *N*-edge calculations in U oxides, the dominant contributions to the near-edge features arise from the first and second coordination shells around the absorbing atom, which are fully contained within a 7 Å sphere for all structures considered here. This radius therefore captures all relevant U–O and O–U–O multiple-scattering pathways that influence the pre-edge and main-edge regions.^25,26^

**Table 1:** Number of Atoms per Cluster within a 7 Å Cluster Radius from the Target Atom for Various U Oxides

U oxide	Number of atoms per cluster
UO_2_	99
U_4_O_9_	123
U_3_O_7_	125
U_3_O_8_	89
KUO_3_	87
BaUO_4_	87

The U oxide crystal structures used in this work were selected based on the availability of comprehensive experimental data.^14,27–31^ Among the possible choices, we have specifically chosen structures for which the experimental studies sufficiently specified their sample preparation methods, analysis methods, and uncertainties, including stability under an electron beam. The latter is the reason that BaUO_4_ was chosen instead of UO_3_.^32^

### Experimental Details

#### U Oxide Sample Preparation

Six U oxide compounds with well-established U valence states were synthesized as spectroscopic reference samples: UO_2_, U_4_O_9_, U_3_O_7_, U_3_O_8_, KUO_3_, and BaUO_4_. Stoichiometric UO_2_ was obtained from hyperstoichiometric UO_2+*x*_ (ASTM C753–04 grade)^33,34^ by thermal reduction in Ar/5% H_2_ using a simultaneous thermal analyzer under a glovebox-controlled atmosphere (O_2_, H_2_O < 20 ppm). The final stoichiometry (O/U = 2.02 ± 0.01) was confirmed by mass change analysis and powder X-ray diffraction. The higher oxides U_4_O_9_, U_3_O_7_, and U_3_O_8_, as well as KUO_3_, were prepared by solid-state synthesis following established protocols.^35–37^ BaUO_4_ was obtained by annealing a stoichiometric U_3_O_8_–BaCO_3_ mixture at 1173 K in air.^38^ Detailed descriptions of the sample composition, U valence states, and preparation parameters are provided in the Supporting Information (Text S2 and Tables S1–S2).

Samples for microscopy for all U oxide standards were prepared in an anoxic chamber (Coy Laboratory Products Inc.) supplied with a gas mixture of 95% N_2_ and 5% H_2_ and O_2_ < 5 ppm. Spectroscopic grade ethanol was flushed for 1 h with N_2_ gas before being transferred to the anoxic chamber. The U oxide powder samples were suspended in ethanol, briefly homogenized using a dedicated mortar and pestle for each mineral, sealed anoxically, and sonicated for 15 min to prevent aggregation. Several drops of the sonicated suspension were then deposited onto single-crystalline Si grids (SiMPore Precision Membrane Technologies). The grids were heat-sealed into Mylar bags and stored in an anoxic environment until the spectromicroscopic measurement. The grids were plasma-ashed using a downstream Mobile Cubic Asher (MCA, ibss Group, Inc.) at 35W for 2 min before the suspension deposition to make the grids hydrophilic and remove surface contaminants. The same gentle conditions were used after the suspension deposition, just before inserting the sample into the TEM, to prevent the buildup of hydrocarbons during the EELS experiment. We minimized the time during which the samples were exposed to air, from mounting them in the TEM holder to transferring them to the plasma asher and TEM.

#### EELS Acquisition

STEM-EELS measurements were performed in a double-aberration corrected Titan 60–300 transmission electron microscope (ThermoFisher Scientific) operated at 300 keV and equipped with a GIF continuum K3 direct electron (detection) counting detector (Gatan, Inc., Ametek). The microscope was operated at several beam currents ranging from 25 to 90 pA beam to evaluate the extent of beam damage and to identify the lowest current at which the acquisition could be performed. Low beam currents were necessary because of the samples’ susceptibility to electron beam-induced damage and necessitated adjusting the spectrometer-detector alignment to optimize sensitivity and SNR. This was achieved by increasing the dose rate per pixel and setting the counting filter thresholds appropriately. The GIF continuum K3 detector has high sensitivity, and the standard setup spreads the electron dose across 2000 vertical pixels in one energy channel. At these low doses, the signal would be under the detection limit for a single pixel (<0.01 electrons/pixel/s). We compressed the spectrum image from 2000 to 160 vertical pixels, allowing us to increase the dose rate per pixel above the noise floor. We collected low-loss and high-loss 3D-spectral hypercubes of the O K-edge, U N_6,7_-, and N_4,5_-edges for U oxide standards using a 0.18 eV dispersion, a 5 mm entrance aperture, a 0.8 nm pixel (probe step) size, 600–800 pixels, and 0.5 s pixel dwell times.

#### EELS Analysis

Due to the low doses required to minimize beam damage, the spectrum images (SIs) obtained were noisy. To maximize the SNR and minimize artifacts, a series of processing steps was performed. First, the dual EELS SI peaks were aligned by their zero-loss peak, and X-ray spikes were removed. Then, 5 to 10 SIs for each sample were merged by interpolating the energy axis so that the energy channels for different SIs fell at the same energies. Subsequently, an experimental reference vacuum spectrum was subtracted from every pixel (e.g., Figure S1). The signal obtained when scanning over the vacuum originates from the energy distribution of the field-emission electron probe,^39^ and although very low, as shown in Figure S1 (an average contribution of less than one count per pixel per energy channel), it is observable in the SNR of the measurements. At this point, denoising was applied by performing principal component analysis^40^ and rebuilding the spectra with components with a variance above the noise level. Subsequently, cluster analysis was performed on the collection of spectrum images for each sample, classifying the spectra contained in every pixel by similarity after normalization of the spectra by intensity.^41^ This procedure allowed segmenting spectra obtained from the vacuum, regions damaged by the electron beam, and most importantly suitable areas for measurement (Figure S2). One cluster was selected for each sample, therefore obtaining one representative spectrum. At this stage, the average spectrum was fitted with PyEELSMODEL^42^ to subtract the background and obtain the O *K*- and the U *N*_6,7_-, and *N*_4,5_-edges. This was necessary due to the overlap of the tail of the U *N*_6,7_-edge and the O *K*-edge, which did not allow the use of a typical power-law background.

## Results and Discussion

Experimentally, the EELS technique is attractive to unravel the 5f occupancy of nanoscale U oxides because of its high spatial resolution. By analyzing energy-loss characteristics associated with core-level excitations, EELS provides direct access to oxidation states, coordination geometry, and the degree of hybridization between U and O orbitals. A number of prior X-ray absorption spectroscopy (XAS) studies have examined the electronic structure underlying the O *K*-edge and the U *N*_4,5_- and *N*_6,7_-edges. They include multiplet-based treatments of the *N*_6,7_-edge in molecular U­(IV) complexes, experimental *N*_6,7_-edge surveys of actinide oxides, and model Hamiltonian or impurity approaches for metallic carbides.^43–46^ These works have established that the U *N*-edges are dominated by strong spin–orbit coupling, extensive multiplet structure, and hybridization effects, which often limit their chemical diagnostic capacity. Conversely, O *K*-edge XANES studies on oxides and related materials consistently demonstrate that ligand 2p–metal hybridization yields well-resolved, oxidation-state-dependent features.^47^ Previous work was centered around the study of the O *K*-edge XAS for some of the U oxides studied in this work.^48,49^ Our data generally show good agreement with respect to peak positions, while some of the spectral features such as line shape and peak sharpness cannot be directly compared due to differences in measurement technique (EELS *vs* XAS), related instrumental energy resolution, and spectral broadening conventions. While these insights provide valuable context, the present study is not intended as a comprehensive review or a full multiplet analysis. Instead, the aim of this work is to reproduce and interpret experimental EELS spectra of U oxides through first-principles XANES simulations across the O *K*-edge and U *N*-edges, to establish reliable spectroscopic benchmarks for distinguishing U oxidation states during the reduction process, and to unravel the evolution of U valence states.

To assess the EELS data quality for the studied U oxides and ensure that spectral features are not an artifact of electron beam damage, we conducted a systematic beam sensitivity analysis, as described in the following paragraph.

### Beam Sensitivity

The O *K*-edge was collected for all six U oxides at two currents to assess beam sensitivity (Figure 1). Beam damage with 300 keV electrons at low STEM beam currents primarily manifests as dose-related knock-on damage, reducing the oxide nanoparticle samples, which changes the oxidation state and shape of the O *K*-edge. By comparing the spectral features of this edge at 25 and 50 pA for U_3_O_8_, U_3_O_7_, and U_4_O_9_, all of which are mixed-valence U oxides, it becomes apparent that there are substantial changes in the spectra. For instance, for U_3_O_7_, a distinct peak appears at 533.06 eV at a current of 50 pA, indicating reduction, while a shoulder, shifted to higher energy (534.68 eV), is observed at 25 pA current. For these three U­(V)-containing U oxides, we conclude that a lower current (25 pA) is required to minimize beam damage. A beam current of 25 pA is at the SNR limit for the atomic-scale core-loss EELS measurements that can be achieved with this setup and represents the best possible spectra that are collectable at present. In contrast, the uranate compounds BaUO_4_ and KUO_3_ showed little sensitivity to beam damage even at 50 and 90 pA, respectively. In these compounds, the U valence (U­(VI) and U­(V)) is stabilized by the secondary cation while forming a symmetric octahedral coordination environment, which may increase the threshold energy for knock-on displacements, as is the case in other perovskite systems like SrTiO_3_.^50^ Spectra were only collected at 50 pA for UO_2_ as it is the fully reduced U oxide allotrope and is not susceptible to beam damage. Subsequent experimental data were collected at 25 pA except for KUO_3_ and UO_2_, for which they were collected at 50 pA.

**Figure 1:**
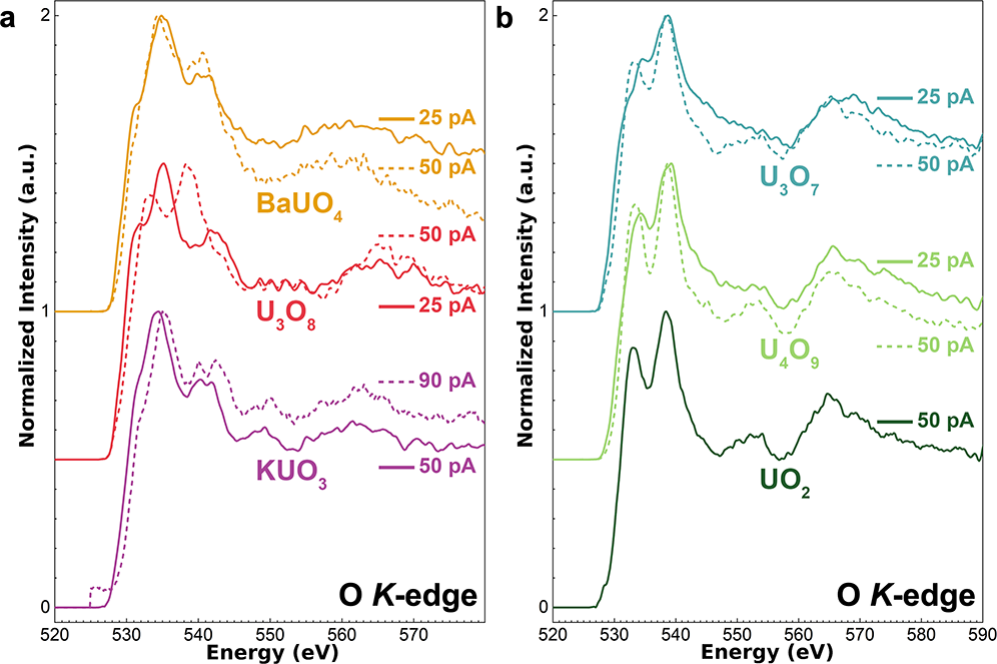
Comparison of experimental EELS spectra at the O *K*-edge for a series of U oxides at a lower (solid lines) and higher (dashed lines) current. The type of U oxide and the current values used are indicated on the graph. (a) Uranate and layered-structure type oxides: (a) BaUO_4_, U_3_O_8_, and KUO_3_. (b) Fluorite-structure type oxides: U_3_O_7_, U_4_O_9_, and UO_2_. All spectra were collected at a 0.18 eV dispersion to reveal more detail, except for KUO_3_ at 90 pA and BaUO_4_ at 50 pA that were measured at a 0.09 eV dispersion.

### Symmetrically Nonequivalent U and O Sites

U oxides exhibit remarkable structural complexity with some phases possessing several symmetrically nonequivalent U and O sites. While experimental spectra arise from an average of all possible sites, theoretical simulations require that each nonsymmetrically equivalent site be calculated within a single modeled cluster. For instance, Figure 2 illustrates the impact of the 14 symmetry-unique O clusters of the U_4_O_9_ crystal structure on the computed XANES signal. For each distinct O cluster, the *K*-edge XANES spectra were calculated and combined to generate a spectrum for the O K-edge that is comparable to the one observed experimentally.

**Figure 2:**
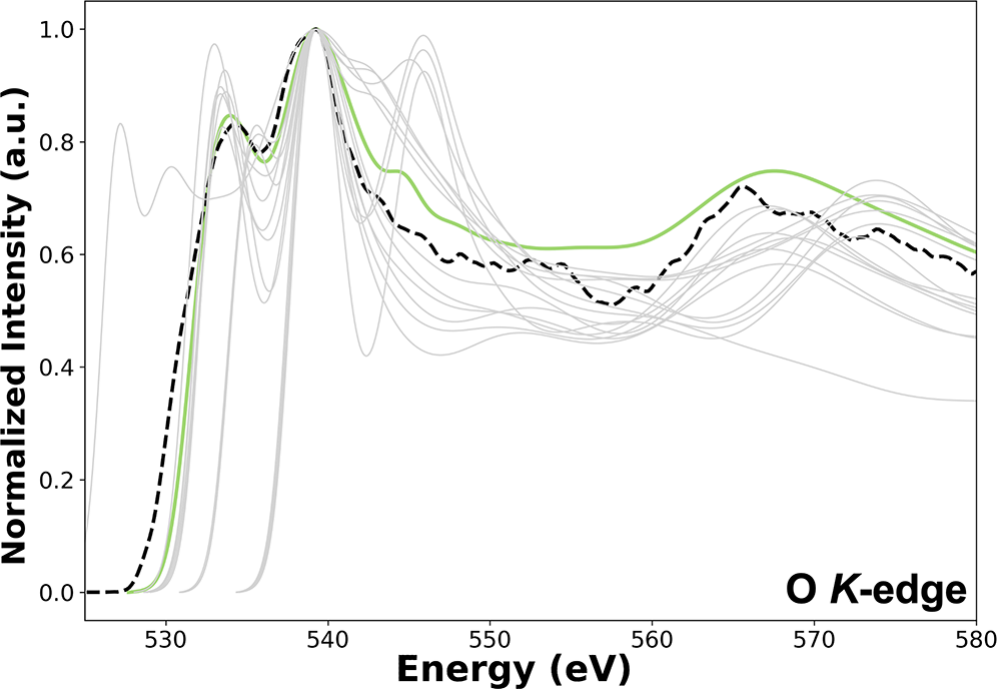
Comparison of the experimental EELS spectrum (black dashed line) with theoretical XANES spectra of the 14 symmetry-unique O clusters (gray solid lines), and the resulting weighted average spectrum (green solid line) for the O *K*-edge of U_4_O_9_, computed using the SCF_exc_ approach.

In this approach, a weighted average of the individual contribution from each of the 14 O clusters has been calculated based on site multiplicity. Calculating the spectrum for each cluster is essential as the unique chemical surroundings and bonding configurations of each target atom can lead to subtle but critical variations of the spectral features. Table 2 shows the number of unique O and U atom clusters for various U oxides.

**Table 2:** Number of Unique O and U Atom Clusters for Various U Oxides

U oxides	O unique clusters	U unique clusters
UO_2_ ^27^	1	1
U_4_O_9_ ^28^	14	7
U_3_O_7_ ^29^	22	8
U_3_O_8_ ^30^	5	2
KUO_3_ ^14^	1	1
BaUO_4_ ^31^	3	1

Therefore, the weighted average was established and applied to each oxide to generate the representative theoretical spectra. We next evaluated the robustness of the calculated O *K*-edge and U *N*-edge spectra across the U oxide series by performing a systematic sensitivity analysis of the Gaussian broadening and core–hole potential. The systematic variation of Gaussian broadening and core–hole lifetime strength is detailed in the Supporting Information (Text S3), with the corresponding results shown in Figures S3–S20. Tables S3–S5 summarize the calculated and experimental peak positions and relative intensities across the U oxide series and are discussed in Supporting Information Text S4. Tables S6–S8 report the percentage errors for both the peak separation and relative intensity ratio, with the absolute error defined as the sum of the absolute percentage deviations between calculated and experimental values, providing a cumulative measure of overall agreement. The final parameter selections were determined by balancing the quantitative agreement with physically realistic broadening parameters and experimental resolution. Further details of the analysis and definitions are provided in the Supporting Information (Text S5).

### O *K*-Edge

The O *K*-edge proved to be a good indirect fingerprint of the U oxidation state and the local coordination environment of O species in U oxides. Figure 3 compares the experimental and theoretical O *K*-edge spectra across the series. Excellent agreement is observed in peak positions and relative intensities, particularly for fluorite-type compounds (UO_2_, U_4_O_9_, U_3_O_7_), because their cubic or pseudocubic symmetry creates a uniform local environment around U atoms, stabilizing U­(IV) and mixed U­(IV)/U­(V) states that can be accurately captured in the model. In contrast, the layered-type oxide U_3_O_8_ and the ternary oxides (KUO_3_ and BaUO_4_) exhibit lower symmetry and more complex coordination environments, with several nonequivalent U and O sites (Table 2). These structural and electronic complexities make the spectra more challenging to reproduce theoretically, leading to larger deviations between calculations and experiment.

**Figure 3:**
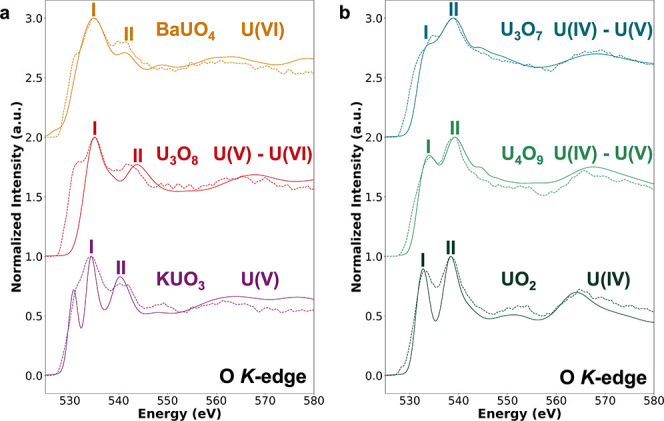
Comparison of experimental EELS (dashed lines) and calculated XANES (solid lines) spectra at the O *K*-edge for a series of U oxides. I and II indicate the first and second calculated intensity maxima in the theoretical spectra, respectively. Spectra were computed using the SCF_exc_ approach, normalized, and aligned with the experimental spectra at the first intensity maximum (peak I). (a) Uranate and layered-structure type oxides: BaUO_4_, U_3_O_8_, and KUO_3_. (b) Fluorite-structure type oxides: U_3_O_7_, U_4_O_9_, and UO_2_.

Nonetheless, the O *K*-edge is highly sensitive to both U oxidation state and structural symmetry, making it a reliable diagnostic for distinguishing among U oxides, fluorite-type, and layered-type alike. Beyond the near-edge region, the evolution of the O *K*-edge line shape provides additional insight into how increasing the oxidation state and structural complexity influences the distribution of unoccupied states at higher energies.

### U *N*_6,7_-Edge

The U *N*_6,7_-edge also varies systematically with the oxidation state but exhibits less pronounced differences between standards as compared to the O *K*-edge. Figure 4 compares the experimental and theoretical U *N*_6,7_-edge spectra across the U oxides. Theoretical TDDFT simulations capture the overall spectral shape, with differences due to the multiplet effects, strong spin–orbit coupling, and partial localization of U 5f orbitals.

**Figure 4:**
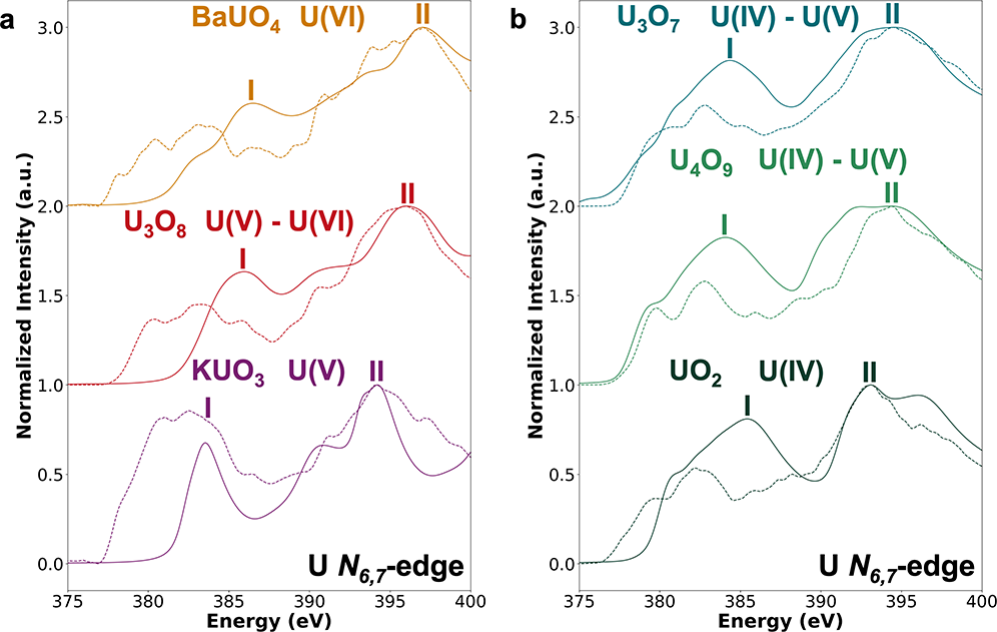
Comparison of experimental EELS (dashed lines) and calculated XANES (solid lines) spectra at the U *N*_6,7_-edge for a series of U oxides. I and II indicate the first and second calculated intensity maxima in the theoretical spectra, respectively. Spectra were computed using TDDFT, normalized, and aligned with the experimental data at the most intense feature (peak II). (a) Uranate and layered-structure type oxides: BaUO_4_, U_3_O_8_, and KUO_3_. (b) Fluorite-structure type oxides: U_3_O_7_, U_4_O_9_, and UO_2_.

### U *N*_4,5_-Edge

The U *N*_4,5_-edge is less problematic than the *N*_6,7_-edge, with peak energies being reproduced more reliably. However, systematic discrepancies in intensity ratios still limit its diagnostic usefulness compared to the O *K*-edge. Figure 5 compares experimental and theoretical U *N*_4,5_-edge spectra across U oxides. In the specific case of BaUO_4_, the second peak of the *N*_4,5_-edge is masked by the Ba *M*_4_-edge contribution, complicating the interpretation of the experimental spectra. While TDDFT reproduces the general trends across the experimental series, the similarity of spectral shapes between compounds with different oxidation states limits its use for discrimination among U oxides. The absence of distinctive features, together with multiplet and spin–orbit coupling effects inherent to the U 5f orbital, limits the diagnostic value of the U *N*_4,5_-edges.

**Figure 5:**
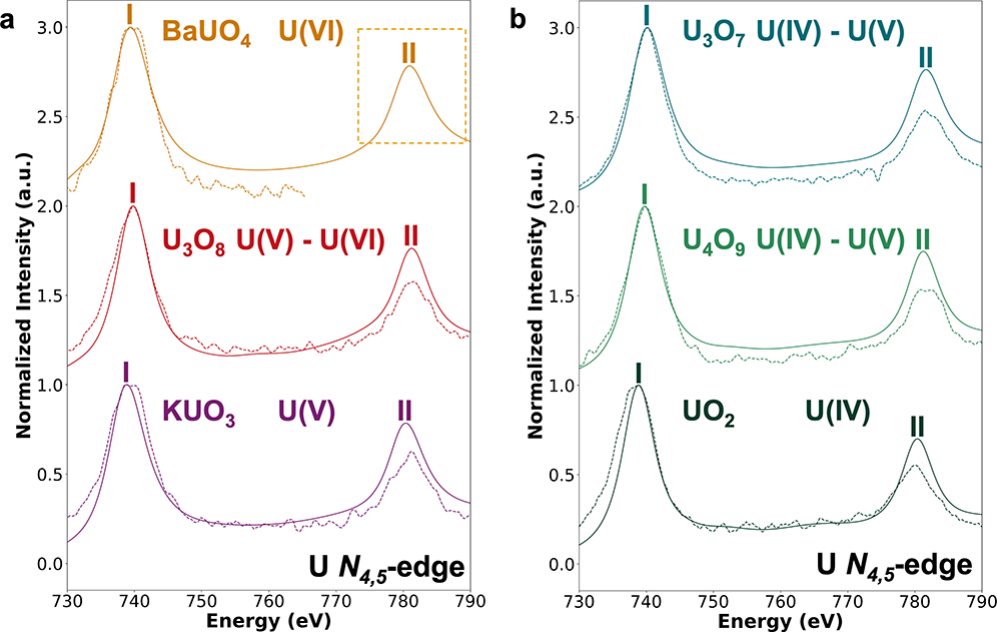
Comparison of experimental EELS (dashed lines) and calculated XANES (solid lines) spectra at the U *N*_4,5_-edge for a series of U oxides. I and II indicate the first and second calculated intensity maxima in the theoretical spectra. Spectra were computed by using TDDFT, normalized, and aligned with the experimental spectra at the first intensity maximum (peak I). (a) Uranate and layered-structure type oxides: BaUO_4_, U_3_O_8_, and KUO_3_. (b) Fluorite-structure type oxides: U_3_O_7_, U_4_O_9_, and UO_2_. The dashed box highlights the region where the experimental U *N*_4_-edge feature of BaUO_4_ would appear, but it is obscured by an overlap with the Ba *M*_4_-edge.

The results show that while the experimental O *K*-edge spectra can be reproduced with high fidelity, the computed U *N*-edges show systematic deviations, particularly in peak intensity and energy separation, reflecting the residual shortcomings of current approximations. The quantitative comparison of peak separations and relative intensity ratios confirms these trends (Tables S6–S8), with a detailed overview provided in the Supporting Information. We next examined each edge in detail.

### Selection of Gaussian Broadening and Core–Hole Lifetime Parameters

The agreement between simulated and experimental spectra was evaluated using the absolute error, defined as the sum of the absolute percentage deviations in Δ*Peak* (Peak II – Peak I, in eV) and *R*_*Intensity*_ (Intensity I/Intensity II, in arbitrary units, a. u.). This enables direct comparison across different combinations of Gaussian broadening and core–hole lifetime parameters.

However, because Δ*Peak* and *R*_*Intensity*_ do not contribute equally to the overall spectral agreement, the parameter set yielding the mathematically lowest absolute error was not always adopted as the final choice. For the O *K*-edge, a core–hole lifetime of 0.2 eV is physically reasonable, while for the U *N*_6,7_- and *N*_4,5_-edge, the values are 0.3 and 5.5 eV, respectively. Spectra computed near these physically meaningful core–hole lifetimes were prioritized, and the final selections were made by considering both quantitative agreement and consistency with realistic broadening parameters and experimental resolution. The optimized parameter sets for each oxide and edge are summarized in Tables S6–S8.

At the O *K*-edge, calculated and experimental spectra agree closely, especially for fluorite-type oxides (Table S6). For example, UO_2_ shows a Δ*Peak* overestimation of 0.38 eV (5.60 eV calc. *vs* 5.22 eV expt.). The relative intensity ratio *R*_*Intensity*_ is 0.90 (calc.) *vs* 0.88 (expt), corresponding to an error of 2.27%, which is in excellent agreement. For the layered oxide U_3_O_8_, a larger Δ*Peak* overestimation of 2.18 eV (8.66 eV calc. *vs* 6.48 eV expt.) is observed and the *R*_*Intensity*_ is 1.30 (calc.) *vs* 1.30 (expt), giving an error of 0%.

At the U *N*_6,7_-edge, discrepancies are more systematic and involve an underestimation of peak separation (Table S7). For UO_2_, Δ*Peak* is underestimated by 3.34 eV (7.64 eV calc. *vs* 10.98 eV expt.). The *R*_*Intensity*_ is 0.81 (calc.) *vs* 0.53 (expt), corresponding to an error of 52.83%, a trend that extends across most oxides. For KUO_3_, Δ*Peak* is underestimated by 1.06 eV (10.63 eV calc. *vs* 11.69 eV expt.), and the *R*_*Intensity*_ is 0.68 (calc.) *vs* 0.86 (expt), corresponding to an underestimation of 20.93%.

At the U *N*_4,5_-edge, the agreement in peak separations is generally good, although the calculated intensity ratios remain consistently lower than in the experiment (Table S8). For instance, in U_4_O_9_, the calculated Δ*Peak* is 41.60 eV (calc.) *vs* 41.38 eV (expt.), overestimated by 0.22 eV, while the *R*_*Intensity*_ is 1.39 (calc.) *vs* 1.82 (expt), corresponding to an underestimation of 23.61%. For KUO_3_, Δ*Peak* is underestimated by 0.8 eV (41.52 eV calc. *vs* 42.32 eV expt.), and the *R*_*Intensity*_ is 1.28 (calc.) *vs* 1.59 (expt.), corresponding to an underestimation of 19.23%. Overall, while this edge captures systematic trends across the series, its diagnostic value remains weaker than that of the O *K*-edge. A detailed oxide-by-oxide analysis is provided in the Supporting Information (Texts S3–S5 and Tables S3–S8).

### Strengths and Limitations of a Combined Experimental and Computational Approach

Overall, the combination of experiments and calculations is essential. Experimental EELS provides nanoscale sensitivity to the oxidation state and coordination, while *ab initio* calculations disentangle overlapping features and isolate the contributions of distinct electronic interactions and, when needed, can be used as predictive tools to validate experimental spectra. However, both have limitations, hence, the value of using them in combination.

The experimental data have evidenced the effect of beam damage on the collected spectra (see Figure 1). At 25 pA, the lowest current that generates sufficient SNR, we cannot exclude some reductive damage. However, the reasonable match with computational results, particularly at the O *K*-edge, offers support for the results and suggests an indirect but robust and diagnostic spectral fingerprint for U oxides. Hence, the computational work enabled the detection of beam-induced reduction and provided a basis for determining the sensitivity of various U oxides to atomic sized EELS probes at effectively very low doses.

Similarly, although DFT has become the standard theory to investigate the electronic structure of solids, it faces significant challenges when applied to U compounds. The partially filled 5f orbitals lie at the boundary between localized and delocalized behavior, making their description highly sensitive to the chosen functional and leading to systematic discrepancies between theory and experiment. Unlike lanthanides with fully localized f orbitals, the U 5f electrons exhibit both spatial overlap with ligand states and strong on-site Coulomb repulsion. This duality leads to competing electronic interactions that are poorly captured by conventional exchange-correlation functionals.^51–54^ Standard local or semilocal DFT functionals (LDA or GGA) tend to overdelocalize the 5f electrons, artificially mixing them with the conduction band. As a result, they underestimate band gaps, distort oxidation states and energetics, and misrepresent hybridization trends with O 2p orbitals. These deficiencies translate directly into discrepancies between the theoretical and experimental spectra. For example, while the O *K*-edge can often be reproduced with reasonable fidelity due to its dominant O 2p character, the U *N*-edges, which probe transitions into unoccupied 5f states, remain challenging to reproduce. In particular, the U *N*_6,7_- and *N*_4,5_-edges are heavily influenced by strong spin–orbit coupling, multiplet splitting, and localized correlation effect phenomena beyond the scope of standard DFT. The failure of standard DFT approximations to capture the physics of strongly correlated materials,^55,56^ such as actinide compounds, has motivated the development of improved methods, most notably the DFT + *U* formalism. The Hubbard *U* term explicitly accounts for on-site Coulomb interactions, restoring partial localization of the 5f orbitals and improving the description of oxidation states and magnetic ordering.

Yet, even with such corrections, further complications remain. For most U oxides, the precise magnetic ground state, whether antiferromagnetic, ferromagnetic, or nonmagnetic, and the magnitude of the associated magnetic moments are not firmly established. The absence of these constraints complicates theoretical work as simulations must be performed without an apparent reference to the true magnetic state. These uncertainties become particularly critical for the U *N*_6,7_-edge, which lies at low excitation energies: here, the interplay of magnetic correlations, spin–orbit coupling, and final-state interactions makes discrepancies between theory and experiment more evident. For this reason, specialized theoretical approaches are required. In this work, the FDMNES code offers methods designed to address such excitations. The SCF_exc_ formalism, applied to the O *K*-edge, explicitly includes the core–hole during the SCF cycle and therefore accounts for the charge redistribution from the ligand to the absorbing atom. For the U *N*-edges, TDDFT was used, which incorporates dynamical screening and improves the description of transitions into localized f states. These methodologies go beyond conventional DFT, enabling a closer match with experimental spectra and providing insights into the origin of the spectral features.

Nevertheless, it is essential to note that these calculations remain rooted in the DFT formalism. They are extensions that introduce explicit corrections for excitations but do not alter the underlying fact that DFT is a mean-field theory designed primarily for the ground state. As such, the inherent challenges of actinide systems, strong electron correlation, relativistic effects, and multiplet structures still place limits on predictive accuracy. For U oxides, the integration of DFT + *U* corrections with FDMNES-based SCF_exc_ and TDDFT approaches provides the most reliable framework currently available at a reasonable computational cost, even though further methodological developments, such as full many-body treatments or beyond-DFT approaches, will be necessary for a quantitatively exact description of actinide spectroscopy.

## Conclusions

The combined experimental and theoretical analysis demonstrates that the O *K*-edge provides an indirect but robust and diagnostic spectral fingerprint for U oxides using EELS at low electron dose. The O *K*-edge accurately captures the oxidation-state-dependent features through strong O 2p–U hybridization, enabling consistent discrimination of U­(IV), U­(V), and U­(VI) oxidation states. This reliability stems from the dominant O 2p–U hybridization, which is well captured by the SCF_exc_ approach. By contrast, both U *N*-edges (*N*_6,7_ and *N*_4,5_) are less predictive. They show larger deviations between theory and experiment, mainly due to multiplet effects, strong spin–orbit coupling, and partial 5f-electron localization.

More broadly, the results emphasize the need to integrate experimental EELS with theoretical computations: experimental spectra provide nanoscale sensitivity to the oxidation state and coordination, while modeling helps guide the choice of experimental conditions. Overall, this integrated data set establishes a benchmark reference framework for U oxide spectromicroscopy, providing both a validated library of experimental standards and a computational strategy that, while not quantitatively exact for all edges, captures the systematic spectral evolution across oxidation states. In summary, this work provides a robust framework for simulating U oxides and establishes a reference point for future work, particularly for nanoscale U valence state mapping.

## Supplementary Material





## Data Availability

The experimental EELS spectra generated in this work have been deposited in the EELS Atlas database, where they are publicly accessible.

## Notes

The Supporting Information (PDF) is available free of charge on the ACS Publications Web site. All files are provided as a compressed archive downloadable from the Supporting Information. The Supporting Information is available free of charge at https://pubs.acs.org/doi/10.1021/acs.jpca.5c07789.

Text S1, summarizing popular computational codes currently available for the calculation of EELS spectra; Text S2, describing the U oxide reference compounds investigated in this study, together with Table S1, listing the reference U compounds selected, and Table S2, reporting the annealing conditions used for their preparation; Figure S1, showing the signal obtained when scanning over the vacuum; Figure S2, showing the cluster analysis segmentation. Text S3, describing the sensitivity analysis of Gaussian broadening and core–hole lifetime, and Figures S3–S20, showing the systematic variation of these parameters; Text S4 and Tables S3–S5, summarizing calculated and experimental peak positions and relative intensity ratios across the U oxide series; and Text S5 and Tables S6–S8, providing a detailed description of the error analysis and the selection of Gaussian broadening and core–hole lifetime parameters (PDF)
The Supporting Information also includes the crystallographic input structures and the input files used for the FDMNES calculations (ZIP)
