# Supplementary material for: Fingerprinting Uranium Oxides with Electron Energy Loss Spectroscopy Supported by Theoretical Computations
Source: J Phys Chem A. 2026 Mar 4;130(11):2329–37. doi: 10.1021/acs.jpca.5c07789 (PMC13007028; doi:10.1021/acs.jpca.5c07789)
Supplement: Supplementary file 1 [file jp5c07789_si_001.pdf]

Supporting Information for

Fingerprinting Uranium Oxides with Electron Energy Loss Spectroscopy Supported by Theoretical Computations

Jacopo Carbone†‡*, Barbora Bártová†, Thomas La Grange¶, Katharina Reinhold†, Gregory Leinders§, Pau Torruella¥, Cécile Hébert¥, Michel Sassi‡, Rizlan Bernier-Latmani†, Kevin M. Rosso‡*

† Environmental Microbiology Laboratory, École Polytechnique Fédérale de Lausanne (EPFL), Lausanne CH-1015, Switzerland

‡ Physical and Computational Sciences Directorate, Pacific Northwest National Laboratory, Richland, Washington 99354, United States

¶ School of Basic Sciences, Institute of Physics, Laboratory for Ultrafast Microscopy and Electron Scattering, École Polytechnique Fédérale de Lausanne, Lausanne CH-1015, Switzerland

§ Institute for Nuclear Energy Technology, Belgian Nuclear Research Centre (SCK CEN), Boeretang 200, Mol 2400, Belgium

¥ Electron Spectrometry and Microscopy Laboratory, EPFL, Lausanne CH-1015, Switzerland.

Email: jacopo.carbone@epfl.ch
Email: kevin.rosso@pnnl.gov

KEYWORDS Uranium, Uranium Oxides, EELS, XANES, FDMNES, DFT, DFT+U, TDDFT

Text S1: Summary of popular codes currently available to compute EELS

(1) The Finite Difference Method Near Edge Structure (FDMNES) code[1] offers two approaches to solve the electronic structure. The Finite Difference Method (FDM) relies on the full potential by constructing a spatial grid and discretizing the Schrödinger equation across the grid points. This approach avoids approximations to the potential and achieves a high level of accuracy but at a significant computational cost. On the other hand, the Multiple Scattering Theory (MST) operates within the Muffin-Tin approximation, which assumes spherical symmetry around atoms and a constant potential in the interstitial space. MST solves a radial Schrödinger equation within each sphere, providing a balance between accuracy and computational cost.[2] (2) The FEFF code is based on a real-space Green's function formalism. This ab initio multiple-scattering method incorporates features such as screened core-holes, inelastic losses, self-energy shifts, and Debye-Waller factors.[3] (3) WIEN2k and (4) CASTEP are both band-structure based codes. WIEN2k uses the Full-Potential Linearized Augmented Plane Wave (FLAPW) method to calculate electron wavefunctions and eigenvalues with high precision. It treats the potential and charge density without approximations.[4] CASTEP, in contrast, employs a plane-wave basis set, with the interactions between valence electrons and the atomic core modeled using pseudopotentials.[5] (5) The CTM4XAS code specializes in multiplet calculations and is specifically designed to simulate core-level spectroscopy for transition metal complexes, with a particular focus on multiplet structures arising from strong electron-electron interactions.[6] (6) The ORCA code employs the Multi-Reference Configuration Interaction (MR-CI) approach, which is highly accurate for systems with strong electron correlation or multi-reference character but is computationally very expensive.[7]

Some of these codes, like WIEN2k and CASTEP, can calculate EELS spectra, including non-dipole transitions,[8] others can generate theoretical XANES spectra, which can be effectively compared to measured EELS spectra within the dipole approximation, as both techniques probe the same unoccupied electronic states.[9] This not only enables a direct comparison between theory and experiment, but also provides critical insights into the target chemical, crystallographic, and electronic structure. Such comparisons can be used to interpret observed trends and fine structural features in metal oxides. This enables the rationalization of complex phenomena, such as hybridization, crystal field effects, and changes in oxidation state, with greater accuracy. Among the various materials that can be analyzed by spectroscopic techniques, metal oxides, particularly those of actinides, are especially challenging due to their complex electronic structures.

Text S2: Uranium oxide compounds

Six uranium oxide compounds with distinct and well-established uranium valence states were selected as reference materials for spectroscopic analysis. Their phase composition, oxygen-to-uranium ratio (O/U), and dominant uranium oxidation states are summarized in Table S1.

All samples were derived from depleted UO_{2+x} powder of nuclear-purity grade (ASTM C753-04), supplied by FBFC International (Dessel, Belgium). The as-received UO_{2+x} was hyperstoichiometric (O/U > 2) due to slow oxidation to higher oxides such as U_4O_9 and U_3O_7 during storage.[10,11]

Stoichiometric UO_2 was obtained by reduction in a nuclearized glovebox (underpressure −3 to −5 mbar, continuously purified N_2, with O_2 and H_2O levels below 20 ppm). Approximately 50 mg powder was treated in a Netzsch 449 F1 Jupiter® STA using Ar/5% H_2 as carrier gas (50 mL min^{-1}). The profile consisted of an isotherm at 313 K (30 min), followed by heating to 973 K at 10 K min^{-1}, a 60 min dwell, and then cooling to 313 K at −10 K min^{-1} with a final dwell of 60 min. The mass change corresponded to O/U = 2.02 ± 0.01 after correction for drift/buoyancy and accounting for slight oxygen uptake during cooling. The phase-pure fluorite structure (Fm3m) was confirmed by X-ray diffraction (Bruker D8 Advance, glovebox-mounted).

Binary oxides U_4O_9, U_3O_7, and U_3O_8 as well as **KUO_3** were synthesized via solid-state chemical routes as described previously.[12–14] **$BaUO_4$** was prepared by mixing stoichiometric U_3O_8 and $BaCO_3$ (ACS reagent grade, Sigma-Aldrich) powders, followed by annealing in air at 1173 K.[15]

A summary of the annealing and treatment conditions is provided in Table S2.

Text S3: Sensitivity analysis of Gaussian broadening and core-hole lifetime

Systematic variation of Gaussian broadening and core-hole lifetime strengths was performed for O *K*- and U *N*-edges across uranium oxides. This analysis identifies parameter sets that best reproduce experimental spectra and clarifies how spectral features evolve with broadening and screening.

Oxygen *K*-edge

To assess the accuracy of the calculated O *K*-edge spectra across the uranium oxide series, we systematically varied both the Gaussian broadening (G) and the strength of the core-hole lifetime potential. calculations were carried out using Gaussian broadenings of 0.5, 1.0, 1.5, 2.0, 2.5, and 3.0 eV, combined with core-hole lifetime potentials of 0.0, 0.2, 0.4, 0.6, and 0.8 eV. The results are presented in a set of five-panel figures, where each panel corresponds to a fixed core-hole value and displays the spectra for the different broadening conditions. Experimental spectra are overlaid for direct comparison. This analysis enables the identification of parameter combinations that best reproduce experimental features and clarifies how spectral shapes are influenced by broadening and core-hole screening.

Uranium $N_{6,7}$-edge

To assess the accuracy of the calculated U $N_{6,7}$-edge spectra across the uranium oxide series, we systematically varied the Gaussian broadening (G) and the strength of the core-hole lifetime. Calculations were performed using broadenings of 0.5, 1.0, 1.5, 2.0, 2.5, and 3.0 eV, combined with core-holes of 0.0, 0.3, 0.4, 0.5, and 0.6 eV. The results are displayed in five-panel figures, where each panel corresponds to

a fixed core-hole lifetime and includes all broadening conditions. The experimental spectrum is overlaid in each case for direct comparison.

Uranium $N_{4,5}$-edge

To assess the accuracy of the calculated U $N_{4,5}$-edge spectra, we systematically varied Gaussian broadening (G) and the strength of the core-hole lifetime. Calculations were performed with G = 0.5, 1.0, 1.5, 2.0, 2.5, and 3.0 eV, combined with core-holes of 0.0, 2.5, 3.5, 4.5, and 5.5 eV. The results are presented in five-panel figures, with each panel corresponding to a fixed core-hole and containing the full set of broadenings. Experimental spectra are overlaid in each case.

Text S4: Peak positions and intensities for uranium oxides

The calculated and experimental EELS spectra of uranium oxides were analyzed to compare the positions and relative intensities of the main spectral features across the O *K*-, U $N_{6,7}$-, and U $N_{4,5}$-edges. For each edge, the first (Peak I) and second (Peak II) maxima were identified, and their normalized intensities were extracted from both experiment and theory. This comparison enables direct assessment of the influence of Gaussian broadening and core-hole lifetime on the calculated spectral profiles and their agreement with experimental data. The values reported in Tables S1–S3 summarize the calculated and experimental peak positions and relative intensities for the uranium oxide series.

Text S5: Percentage error and absolute error evaluation of calculated vs experimental spectral features

To quantify the agreement between calculated and experimental spectra, we evaluated the percentage error for both the peak separation $\Delta Peak$ (Peak II – Peak I, in eV) and the relative intensity ratio $R_{Intensity}$ (Intensity I / Intensity II, in arbitrary units, a. u.). The percentage error for $\Delta Peak$ was defined as

$$\Delta Peak\ (\%) = \frac{\Delta Peak_{calc} - \Delta \mathrm{Peak_{expt}}}{\Delta \mathrm{Peak_{expt}}} \times 100\% \quad (1)$$

and for the $R_{Intensity}$ as

$$R_{Intensity}\ (\%) = \frac{R^{Calc}_{Intensity} - R^{Expt}_{Intensity}}{R^{Expt}_{Intensity}} \times 100\% \quad (2)$$

Absolute error was defined as the sum of the absolute percentage differences between calculated and experimental values for both $\Delta Peak$ and $R_{Intensity}$ across all uranium oxides, providing a cumulative measure of overall deviation.

$$Absolute\ Error = |\Delta Peak\ (\%)| + |R_{Intensity}(\%)| \quad (3)$$

Selection of Gaussian broadening and core-hole lifetime parameters

The agreement between simulated and experimental spectra was quantified using the absolute error, defined as the sum of the absolute percentage deviations in $\Delta Peak$ and $R_{Intensity}$. This single metric allows direct comparison across different Gaussian broadenings and core-hole lifetimes. Since the mathematical solution is not always physically meaningful, and since it is difficult to provide a proper weight on the overall error for $\Delta Peak$ (energy separation) and $R_{Intensity}$ (intensity ratio), the mathematically determined minimum absolute error was not always taken as the final solution. The final solution was selected based on the overall visual agreement between the theoretically calculated spectra and the experimental spectra, with a preference given to a better agreement at the edge energies rather than at the tail of the spectra. For the O *K*-edge, a core-hole lifetime of 0.2 eV is physically reasonable, while for the U $N_{6,7}$- and $N_{4,5}$-edge the values are 0.3 eV and 5.5 eV, respectively. Spectra computed near these physically core-hole lifetimes were prioritized, and the final selections were made considering both quantitative agreement and consistency with realistic broadening parameters and experimental resolution.

Figures

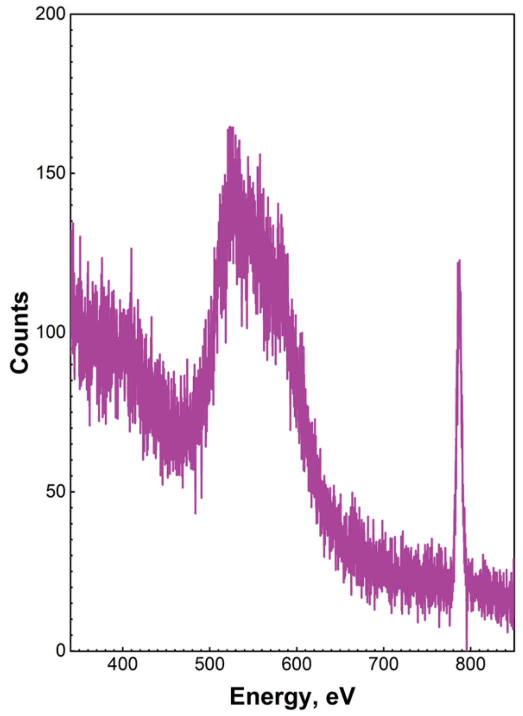

Figure S1: Signal obtained when scanning over the vacuum.

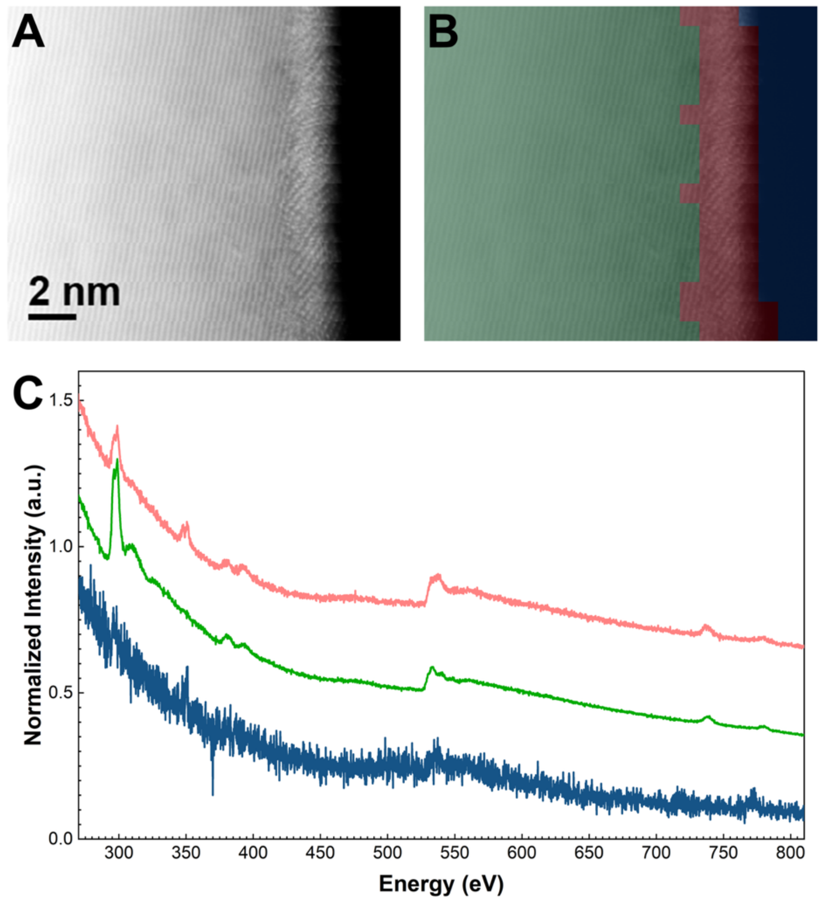

Figure S2: (A) Original STEM-EELS spectrum image of a KUO_3 crystal with a 2 nm scale bar. (B) Cluster analysis segmentation for a spectrum image of KUO_3, showing three components, the vacuum (dark blue), the surface (red), and the bulk (green). (C) The associated spectra are shown and exemplify the distinct signatures of the components. The bulk signature was considered to be the accurate representation of this mineral.

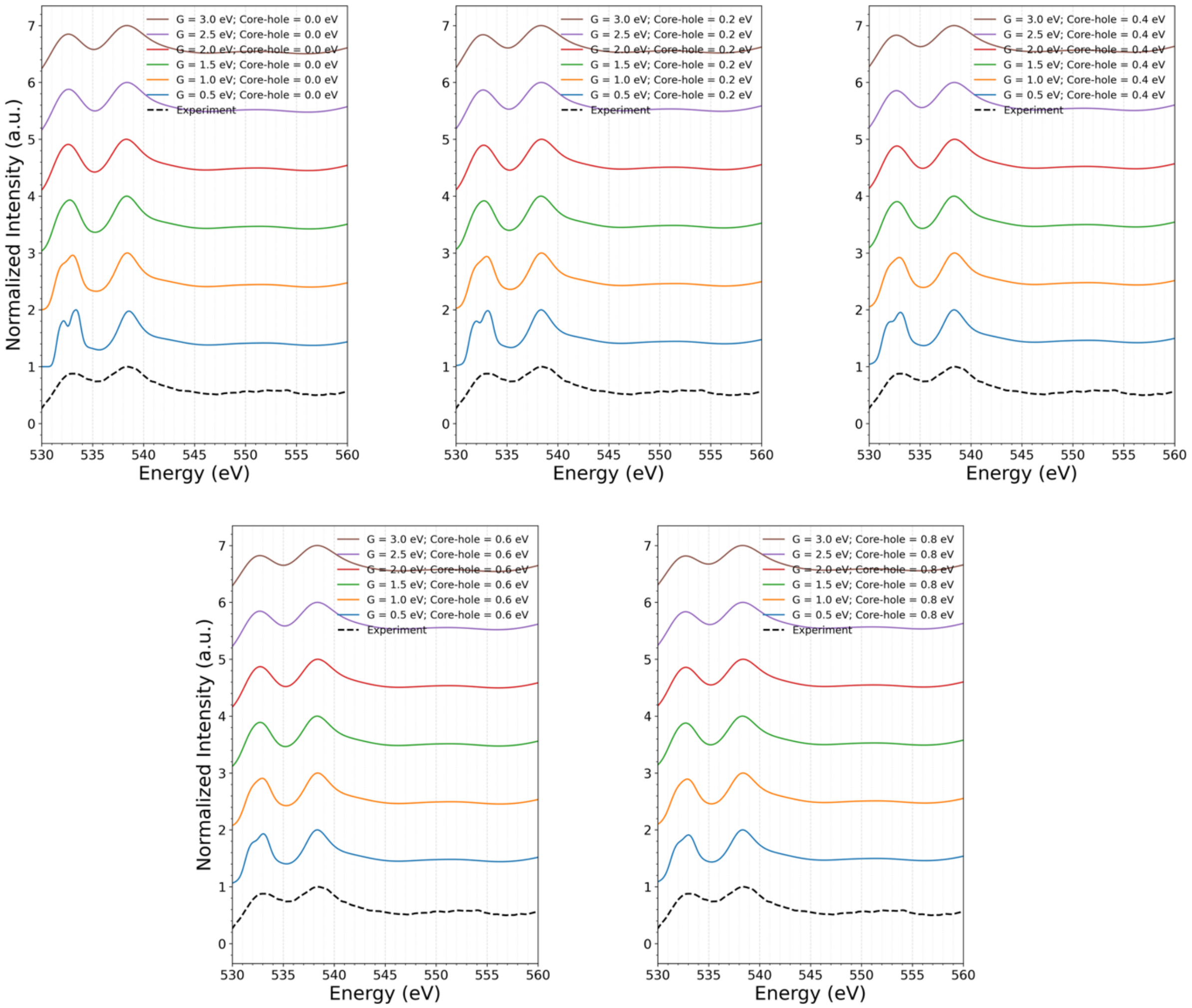

Figure S3: Comparison between experimental EELS and calculated O *K*-edge for **UO_2 O *K*-edge**. The best agreement is obtained with Gaussian broadening G = 2.0 eV and core-hole lifetime = 0.2 eV. Under these conditions, the calculated spectrum reproduces the main edge onset and the relative intensity of the first two peaks, consistent with the fluorite-type U(IV) environment.

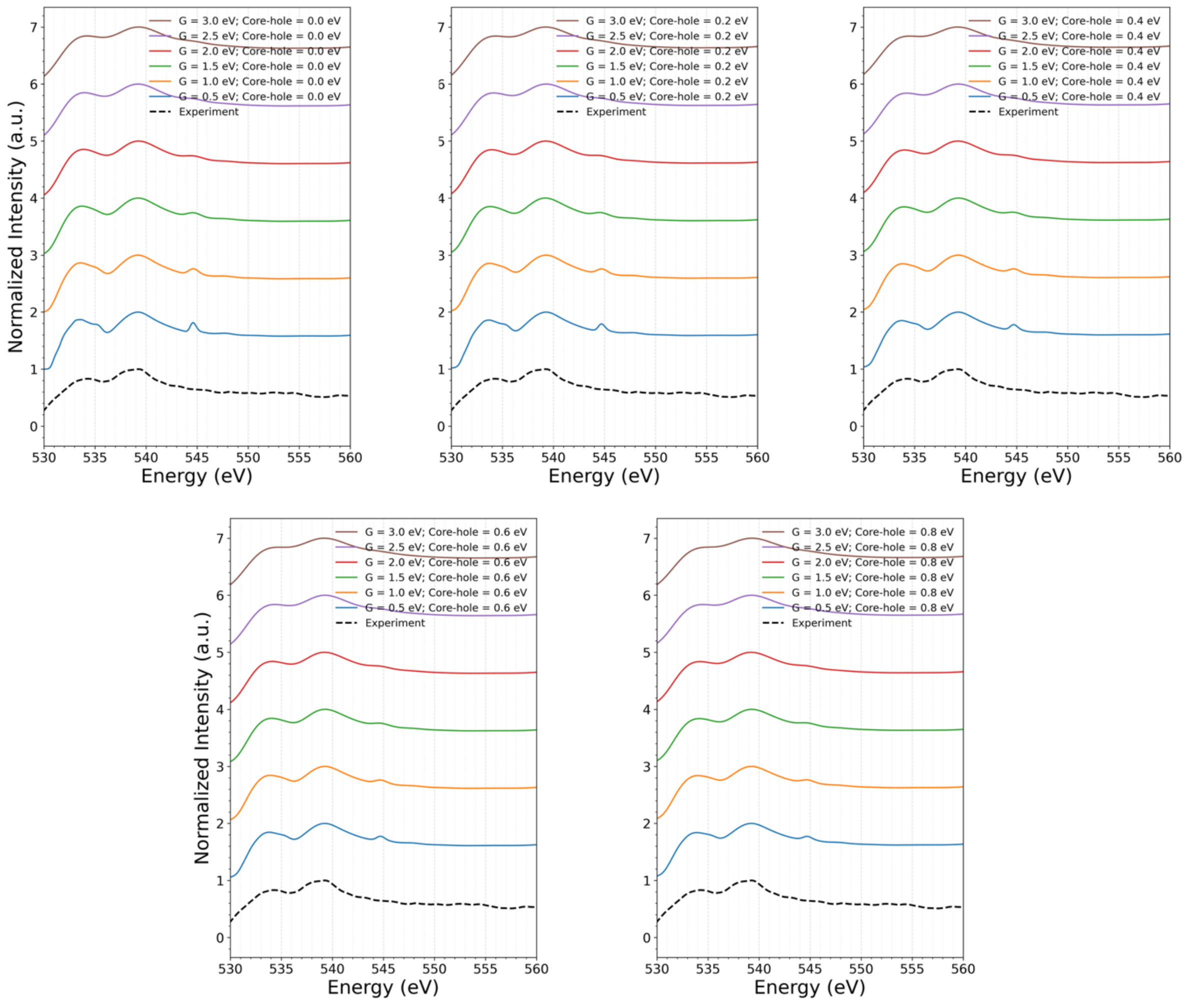

Figure S4: Comparison between experimental and calculated O *K*-edge spectra for **U_4O_9 O *K*-edge**. The calculation with G = 2.0 eV and core-hole = 0.2 eV closely reproduces the experimental peak positions and relative intensities, capturing the mixed U(IV)/U(V) character of this oxide. The good agreement highlights the importance of including moderate broadening and screening to account for the multiple non-equivalent oxygen environments in the structure.

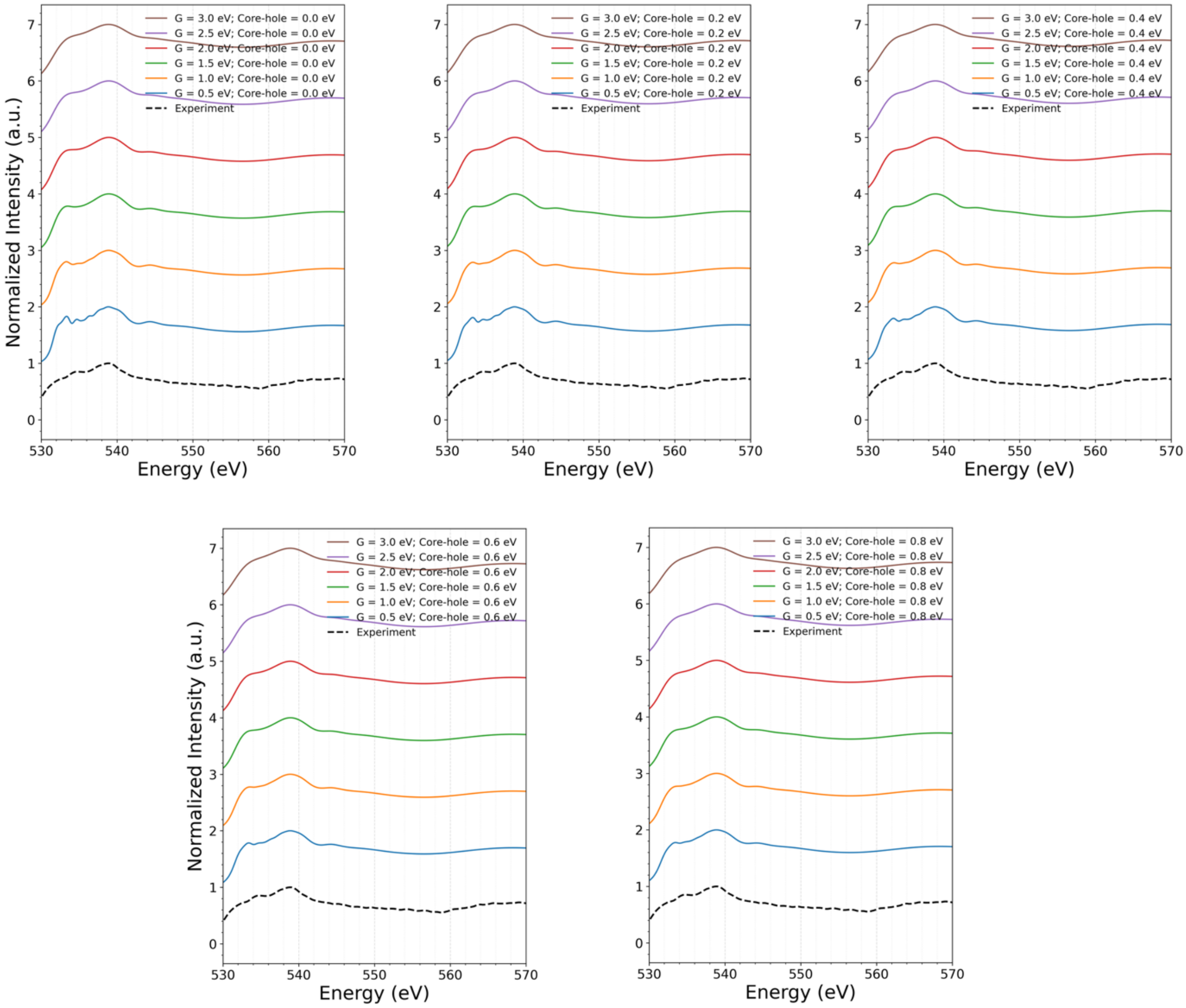

Figure S5: Comparison between experimental EELS and calculated spectra for **U_3O_7 O *K*-edge**. The calculation with G = 2.0 eV and core-hole = 0.2 eV yields the best match to experiment, reproducing the fine structure near the edge onset and relative peak intensities. These features reflect the pseudo-cubic fluorite distortion and the presence of mixed-valence uranium U(IV)/U(V).

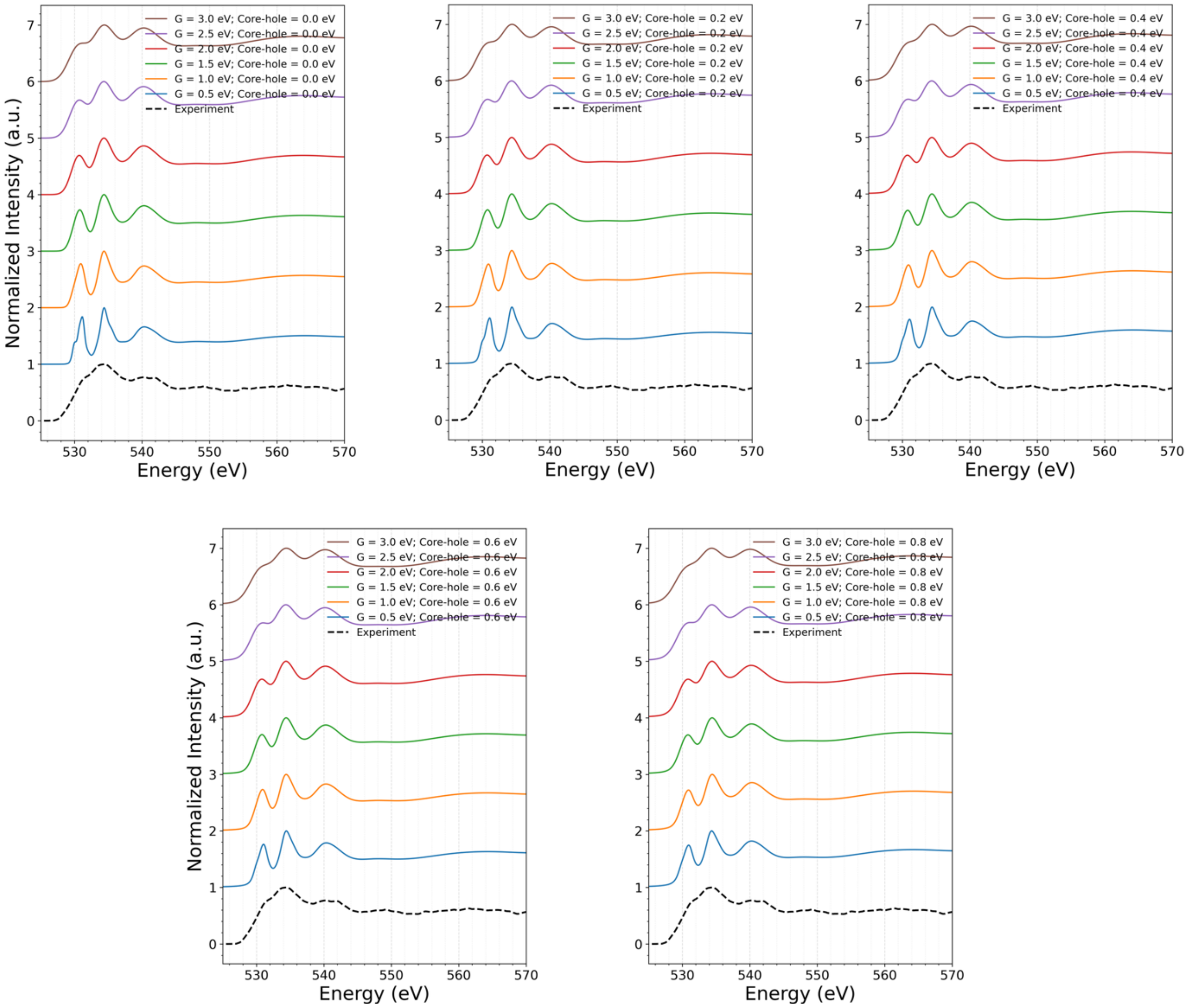

Figure S6: Comparison between experimental EELS and calculated spectra for **KUO_3 O *K*-edge**. The spectrum calculated with G = 1.5 eV and core-hole = 0.2 eV reproduces the experimental peak positions reasonably well. The characteristic features correspond to U(V) coordination within the layered perovskite-type structure.

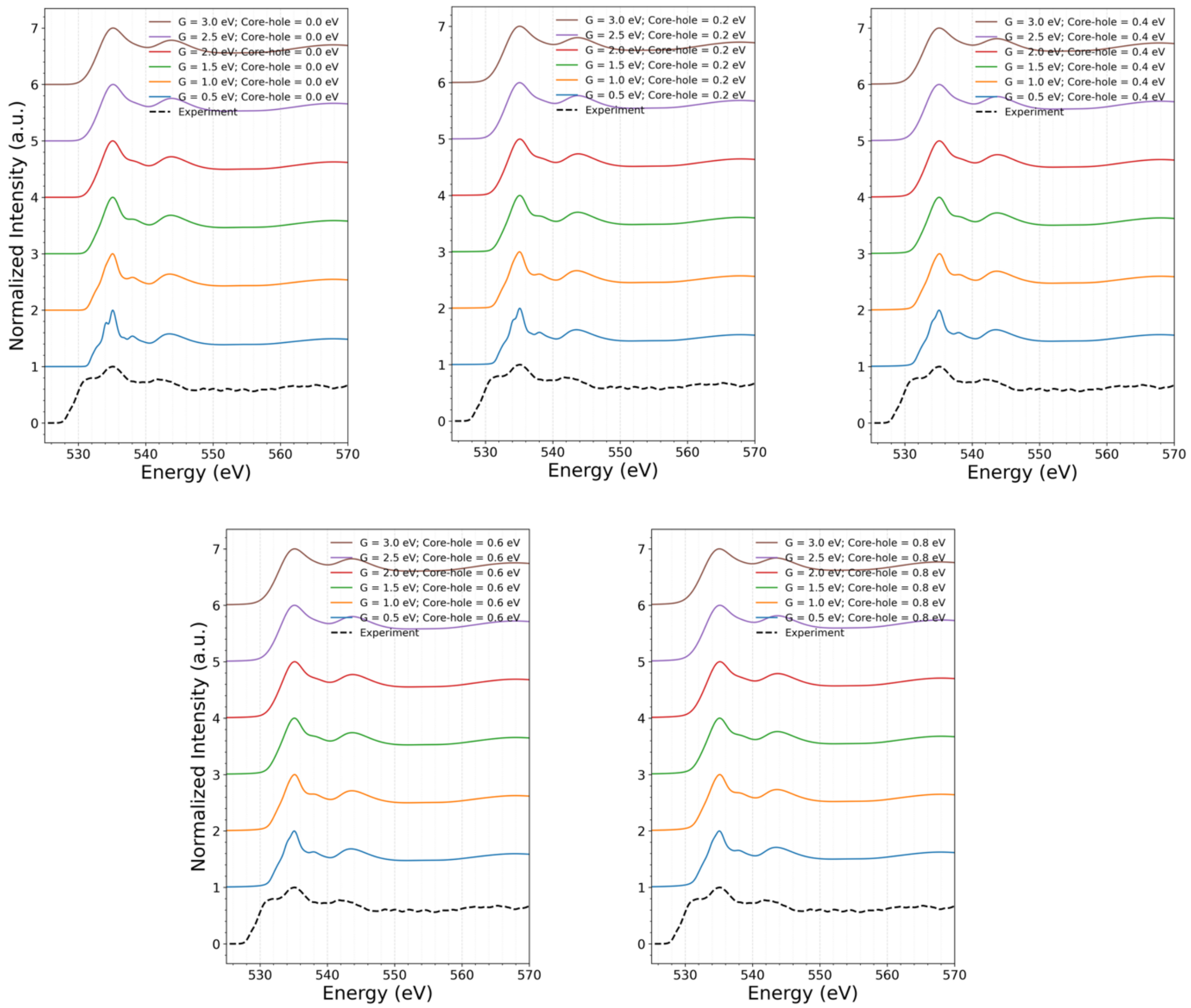

Figure S7: Comparison between experimental EELS and calculated spectra for **U_3O_8 O *K*-edge**. The best agreement is obtained with G = 2.5 eV and core-hole = 0.2 eV, capturing the layered-type structural anisotropy and the contributions of mixed U(V)/U(VI) environments.

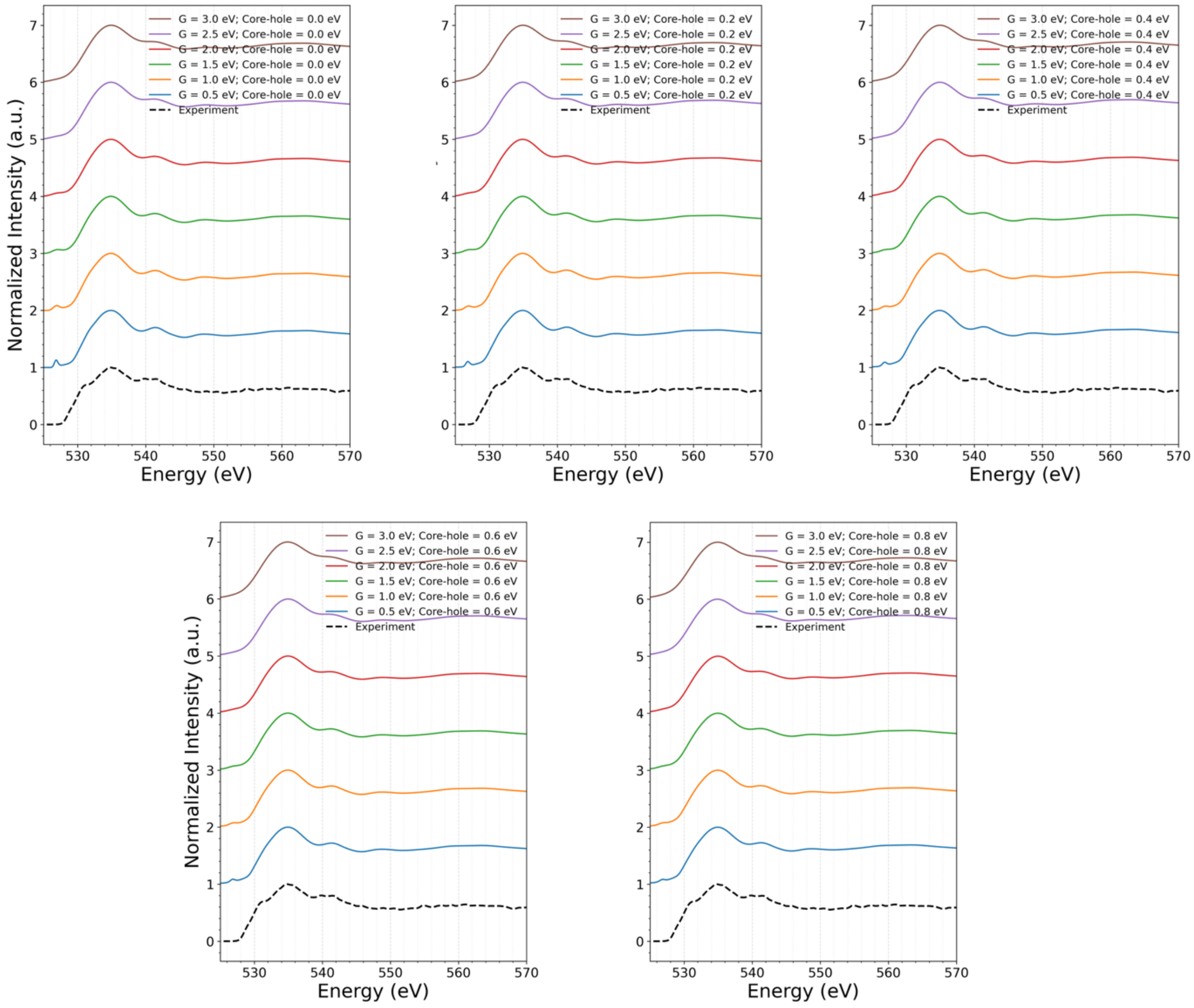

Figure S8: Comparison between experimental EELS and calculated spectra for **$BaUO_4$ O K-edge**. The calculation using G = 2.0 eV and core-hole = 0.2 eV gives the closest match, reproducing the edge onset and intensity distribution of the spectral features. The agreement reflects the U(VI) environment and layered coordination in this compound.

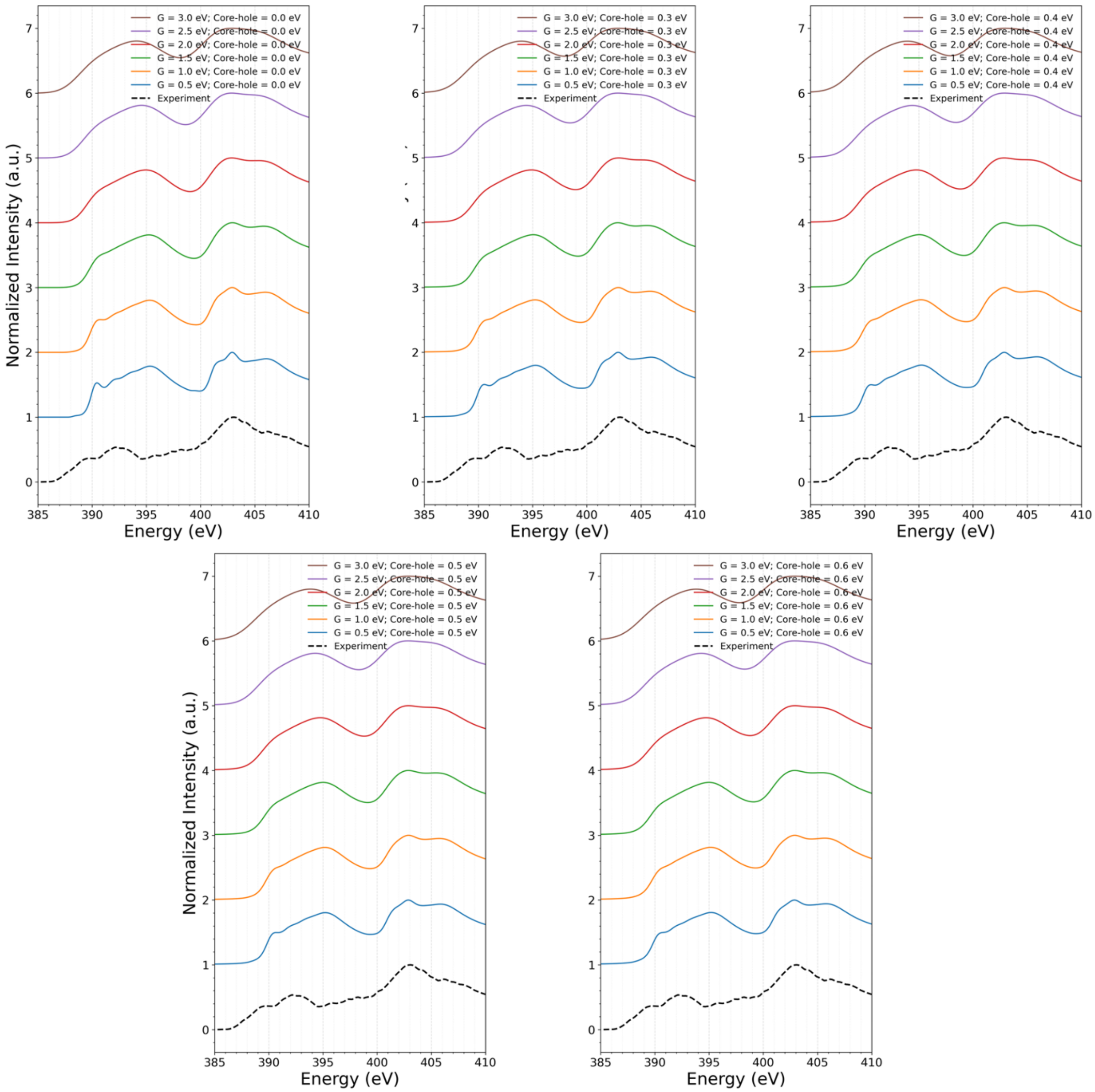

Figure S9: Comparison between experimental EELS and calculated spectra for **UO_2 U *$N_{6,7}$*-edge**. The best agreement is achieved with G = 1.0 eV and core-hole = 0.3 eV, which reproduces the edge onset and relative peak distribution consistent with U(IV) in the fluorite structure.

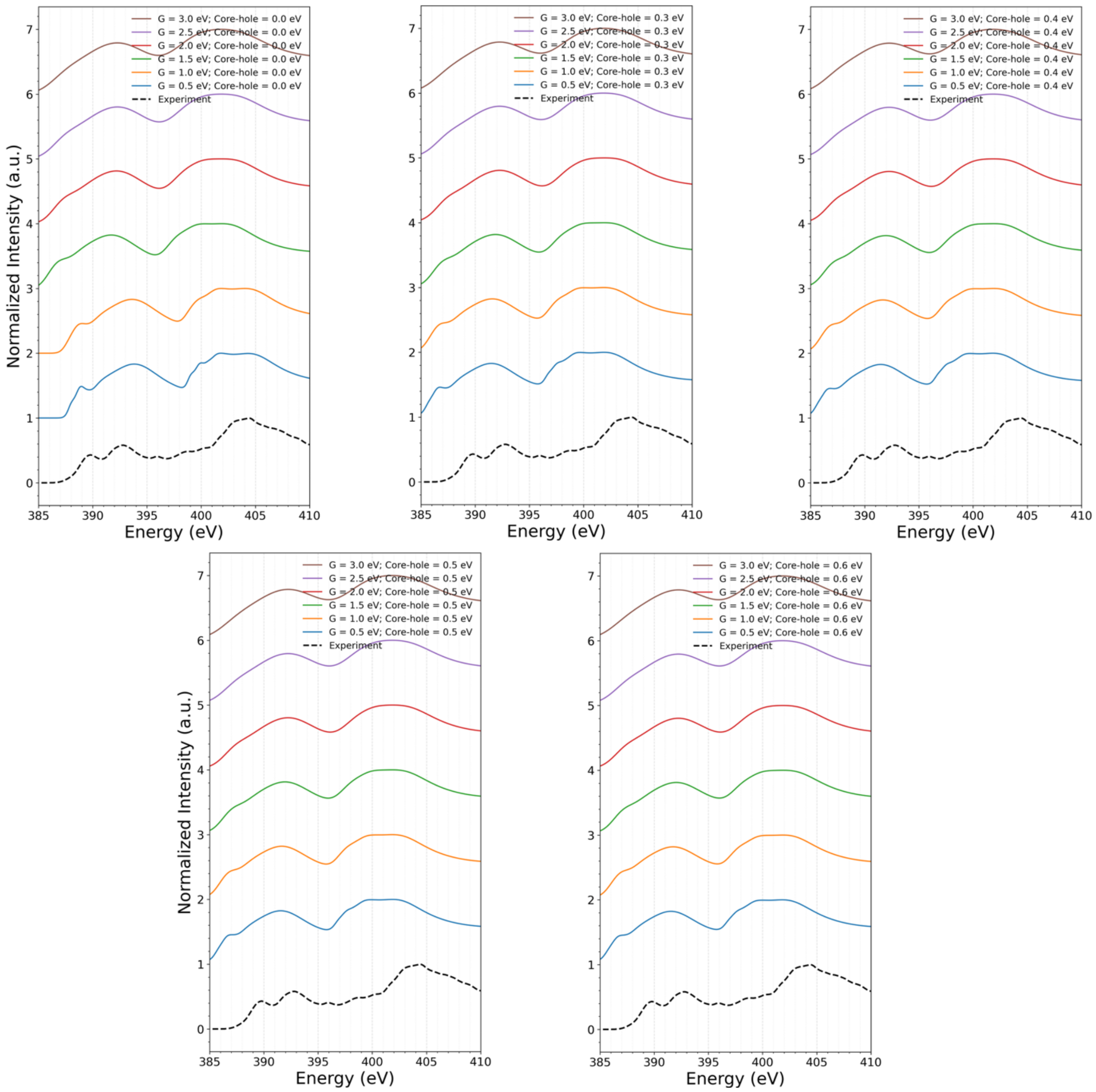

Figure S10: Comparison between experimental EELS and calculated spectra for **U_4O_9 U $N_{6,7}$-edge**. The calculation with G = 1.0 eV and core-hole = 0.3 eV captures the main experimental features, reflecting the mixed-valence U(IV)/U(V) environment. Discrepancies in higher-energy features indicate residual limitations of the TDDFT approach.

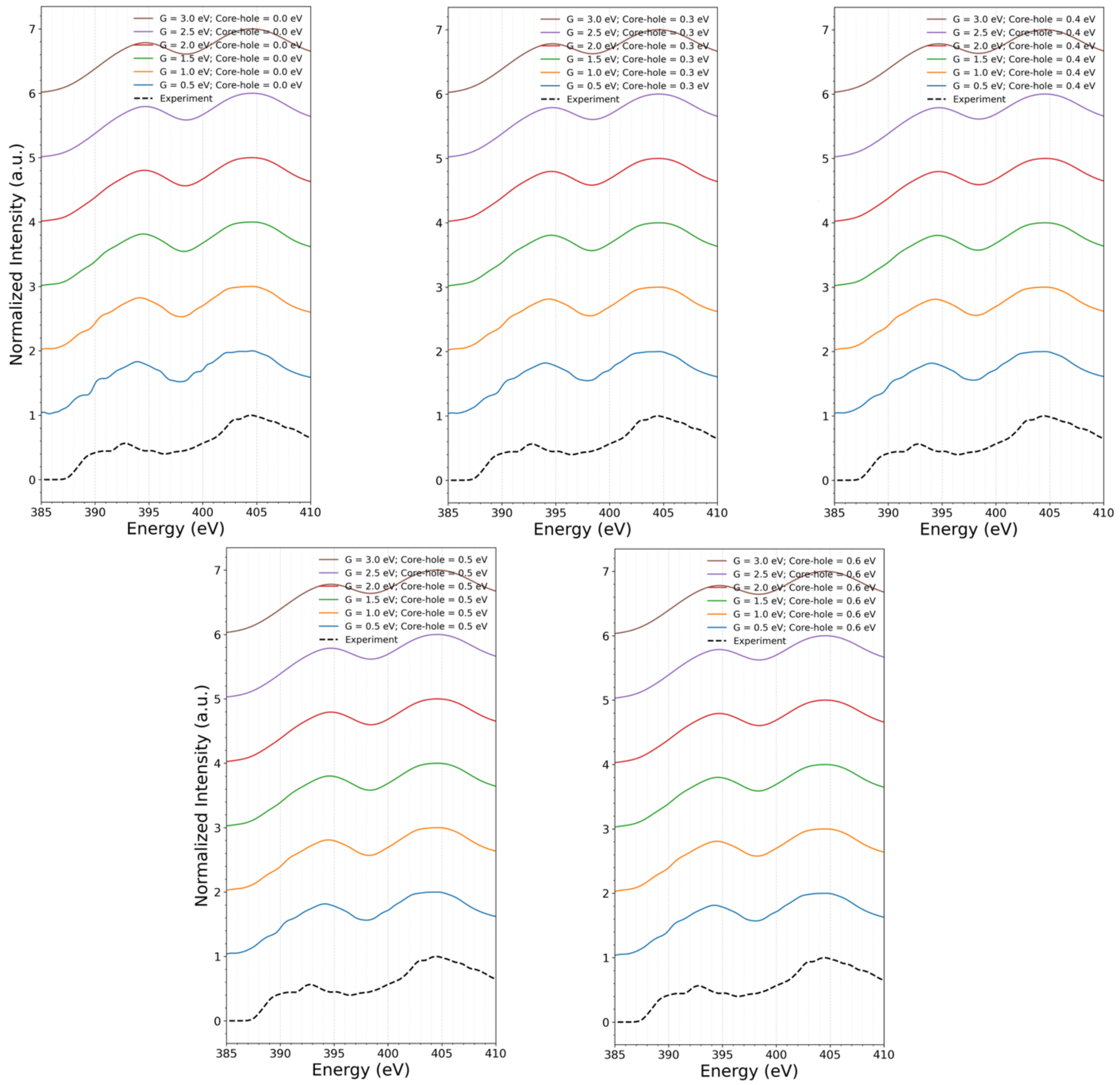

Figure S11: Comparison between experimental EELS and calculated spectra for **U_3O_7 U $N_{6,7}$-edge**. The calculation with G = 1.0 eV and core-hole = 0.3 eV best reproduces the experimental spectral shape, in particular the intensity ratio of the main doublet. This reflects the pseudo-cubic structure and mixed-valence uranium states U(IV)/U(V).

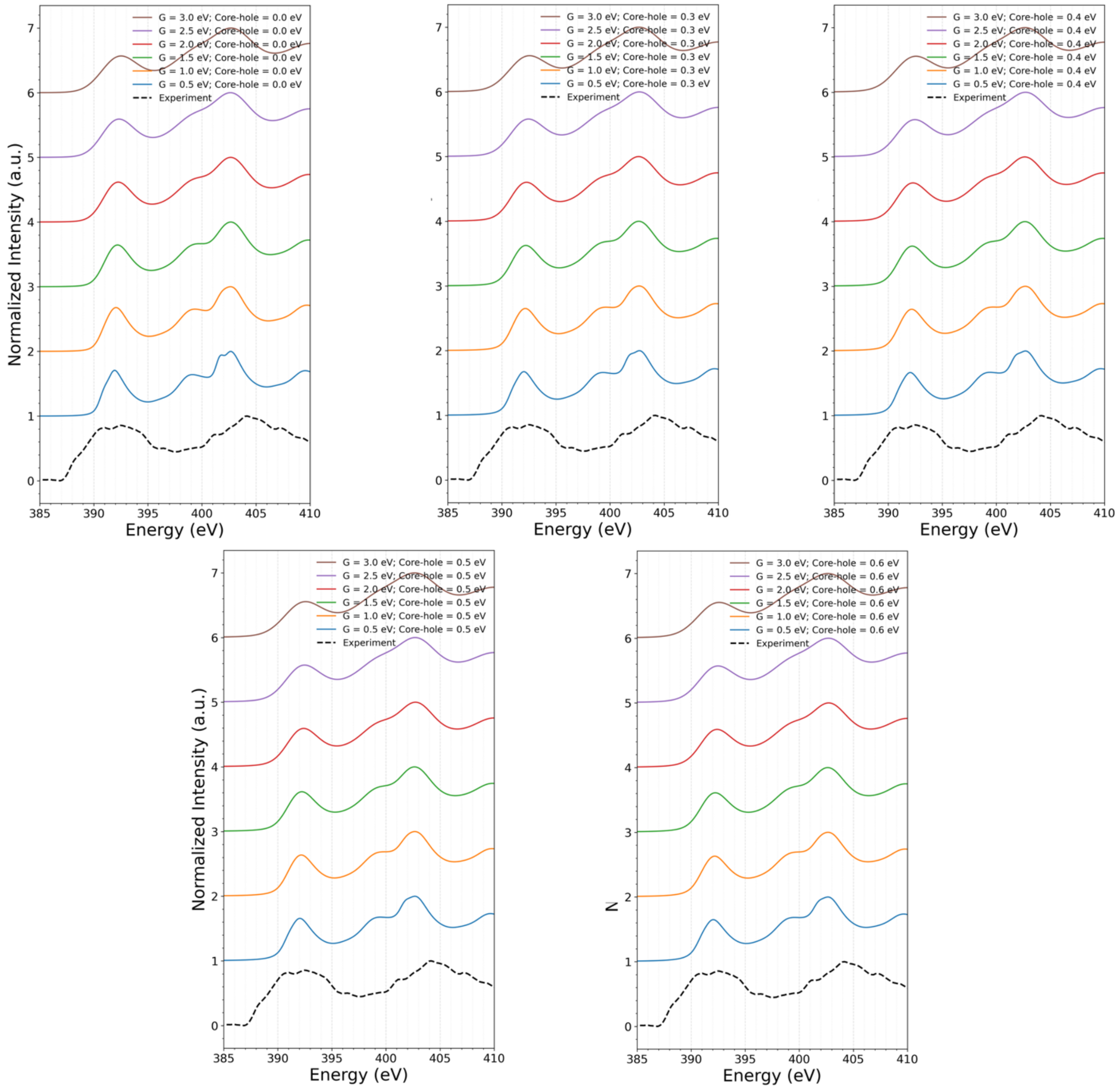

Figure S12: Comparison between experimental EELS and calculated spectra for **KUO_3 U *$N_{6,7}$*-edge**. The calculation using G = 0.5 eV and core-hole = 0.3 eV provides the closest match, reproducing the experimental line shape associated with the U(V) oxidation state in the layered perovskite structure.

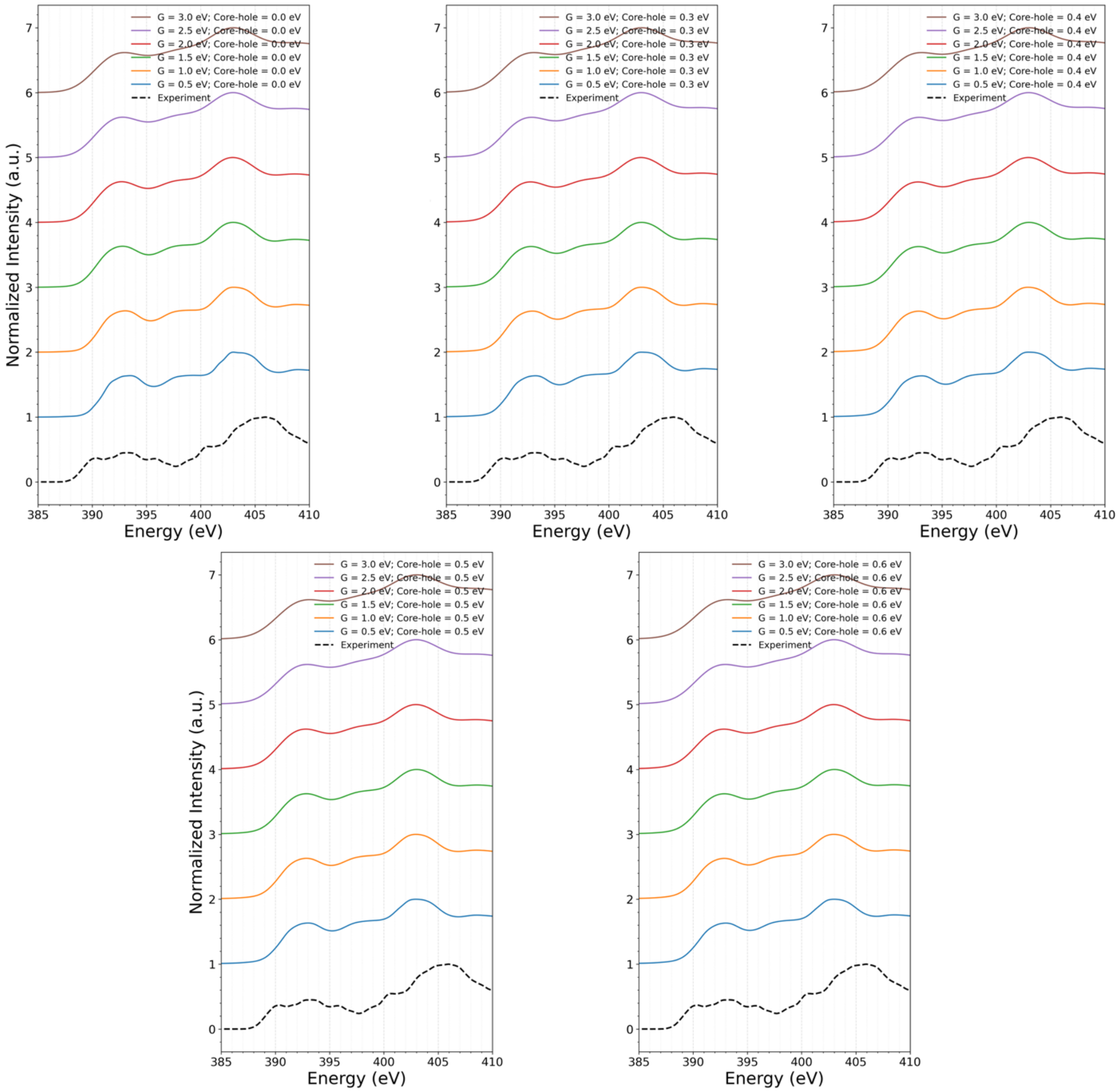

Figure S13: Comparison between experimental EELS and calculated spectra for **U_3O_8 U *$N_{6,7}$*-edge**. The parameter choice of G = 1.0 eV and core-hole = 0.3 eV gives the best agreement, reproducing both the edge onset and relative intensity distribution.

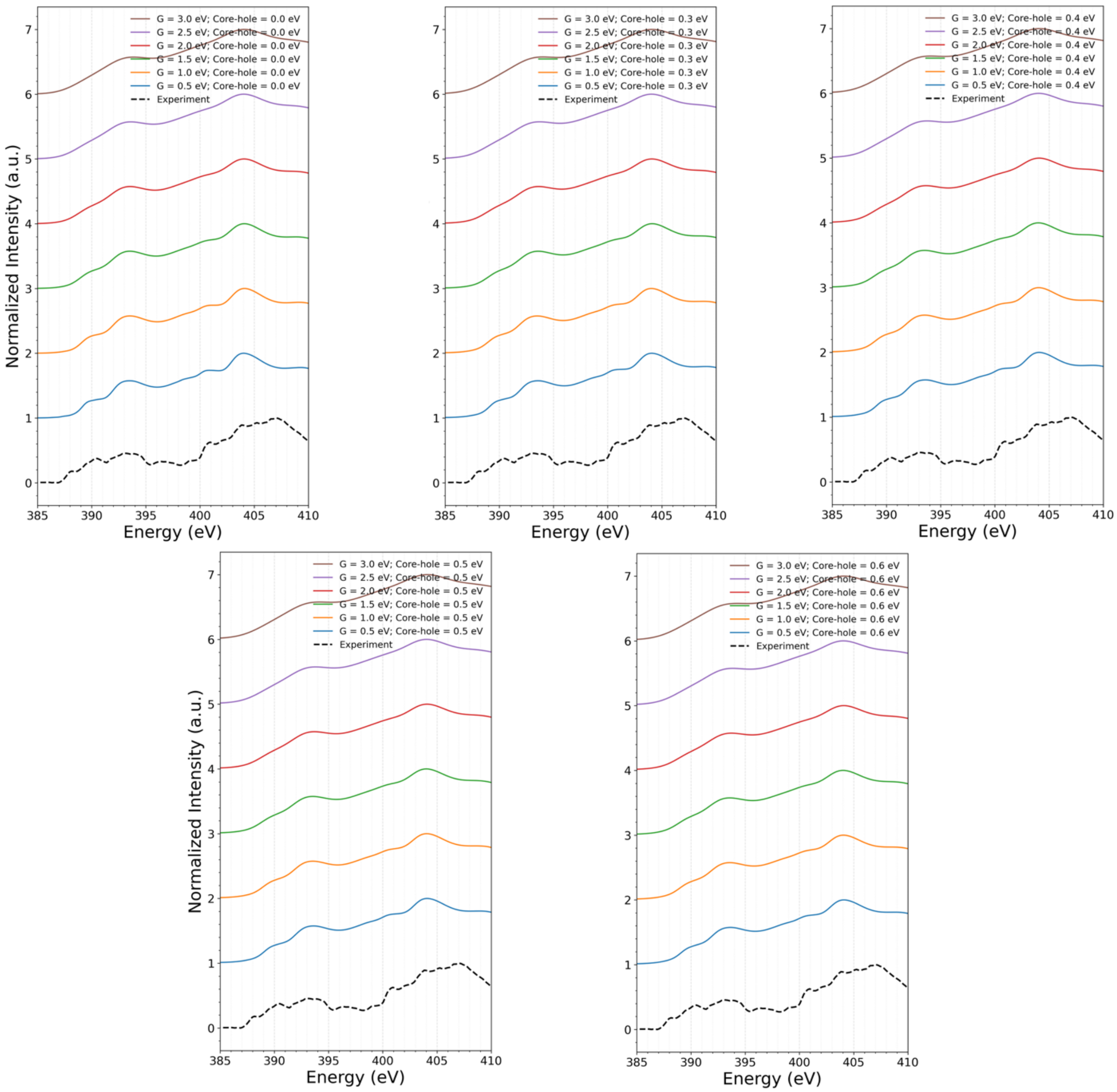

Figure S14: Comparison between experimental EELS and calculated spectra for **$BaUO_4$ U $N_{6,7}$-edge**. The calculation with G = 1.0 eV and core-hole = 0.3 eV reproduces the experimental onset and captures the dominant spectral features associated with U(VI).

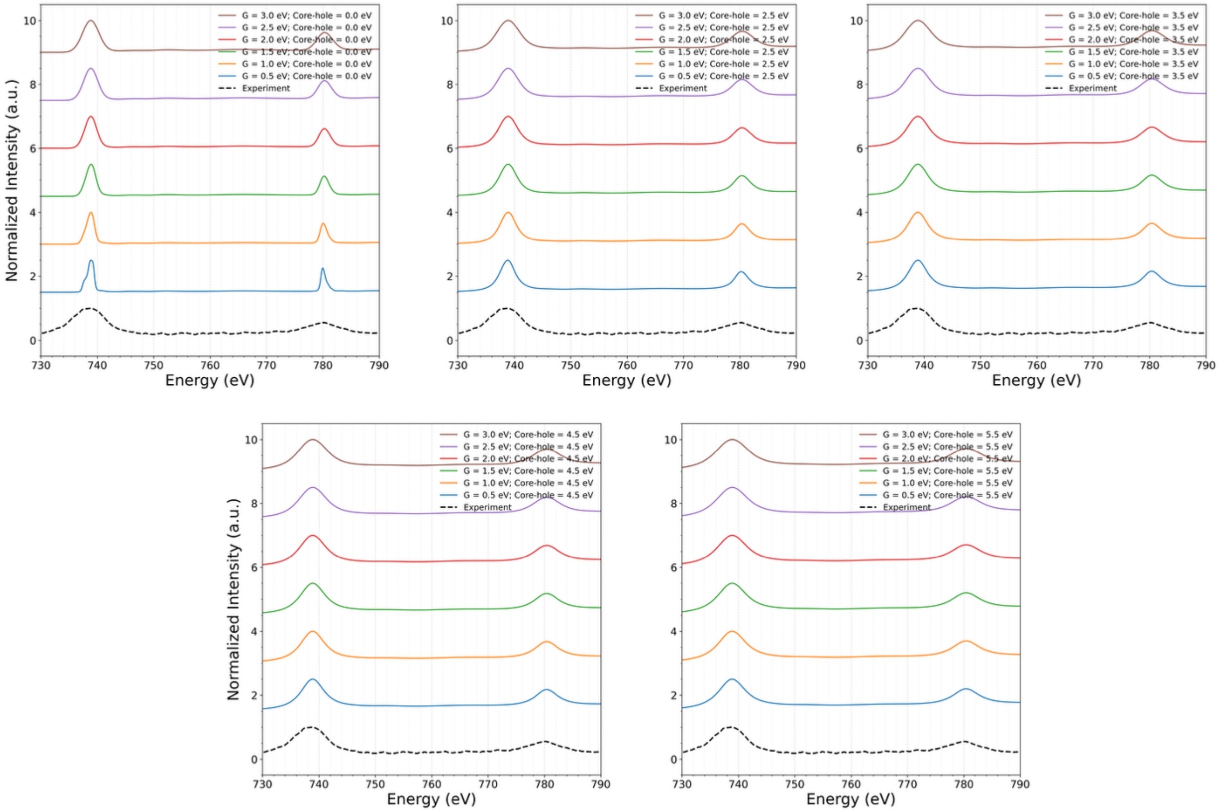

Figure S15: Comparison between experimental EELS and calculated spectra for **UO_2 U *$N_{4,5}$*-edge**. The best match is obtained with G = 1.0 eV and core-hole = 5.5 eV, reproducing the experimental edge onset and relative intensity ratio of the main doublet associated with U(IV).

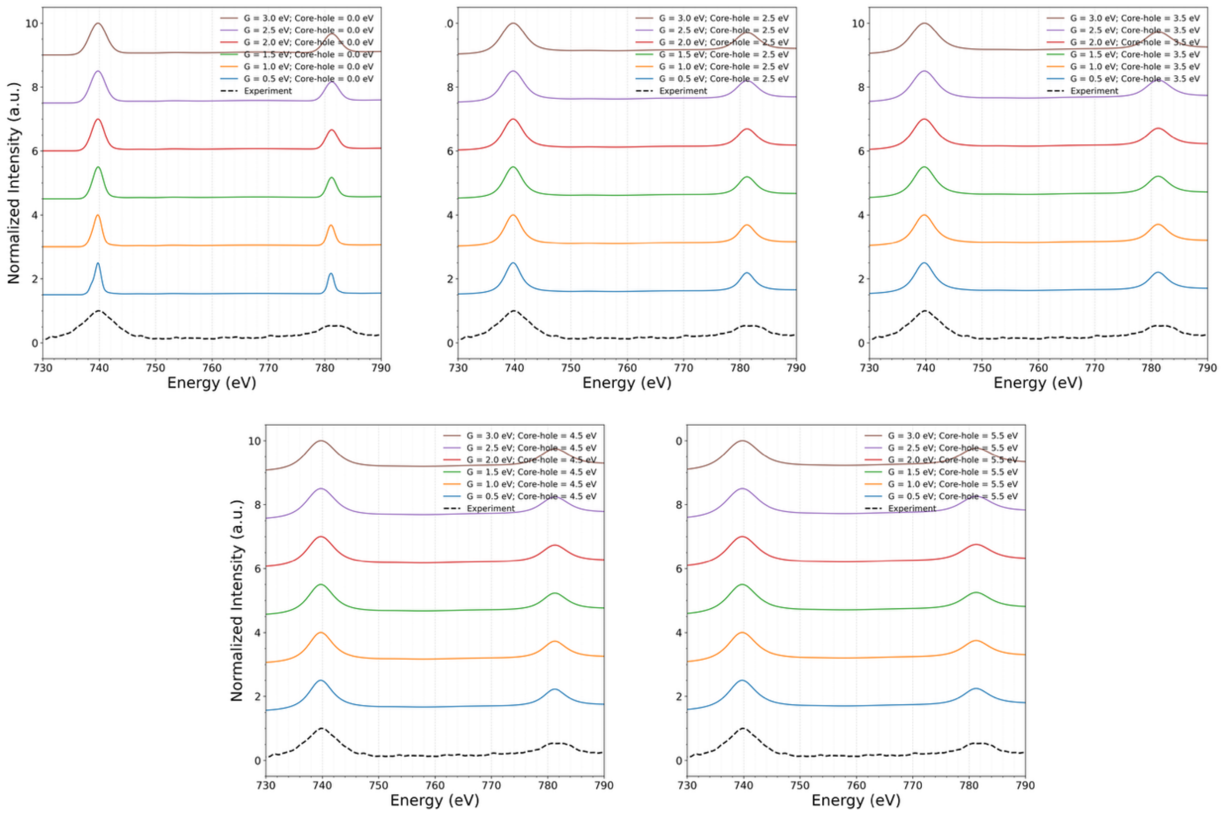

Figure S16: Comparison between experimental EELS and calculated spectra for **U_4O_9 U *N*$_{4,5}$-edge**. The best agreement is obtained with G = 1.0 eV and a core-hole lifetime of 5.5 eV, which reproduces the mixed U(IV)/U(V) environment.

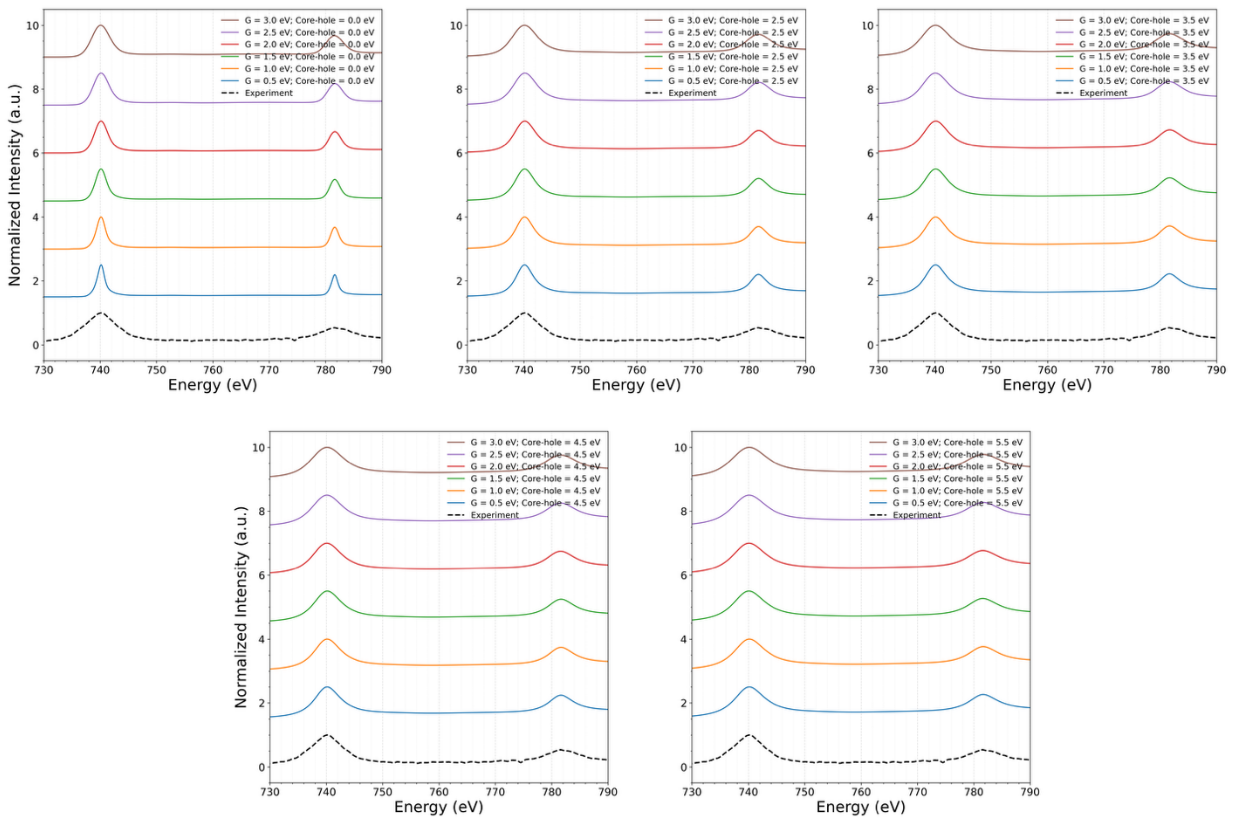

Figure S17: Comparison between experimental EELS and calculated spectra for **U_3O_7 U $N_{4,5}$-edge**. The parameter set G = 1.0 eV, core-hole = 5.5 eV reproduces the spectral shape and relative intensity, consistent with its pseudo-cubic structure and mixed-valence uranium U(IV)/U(V).

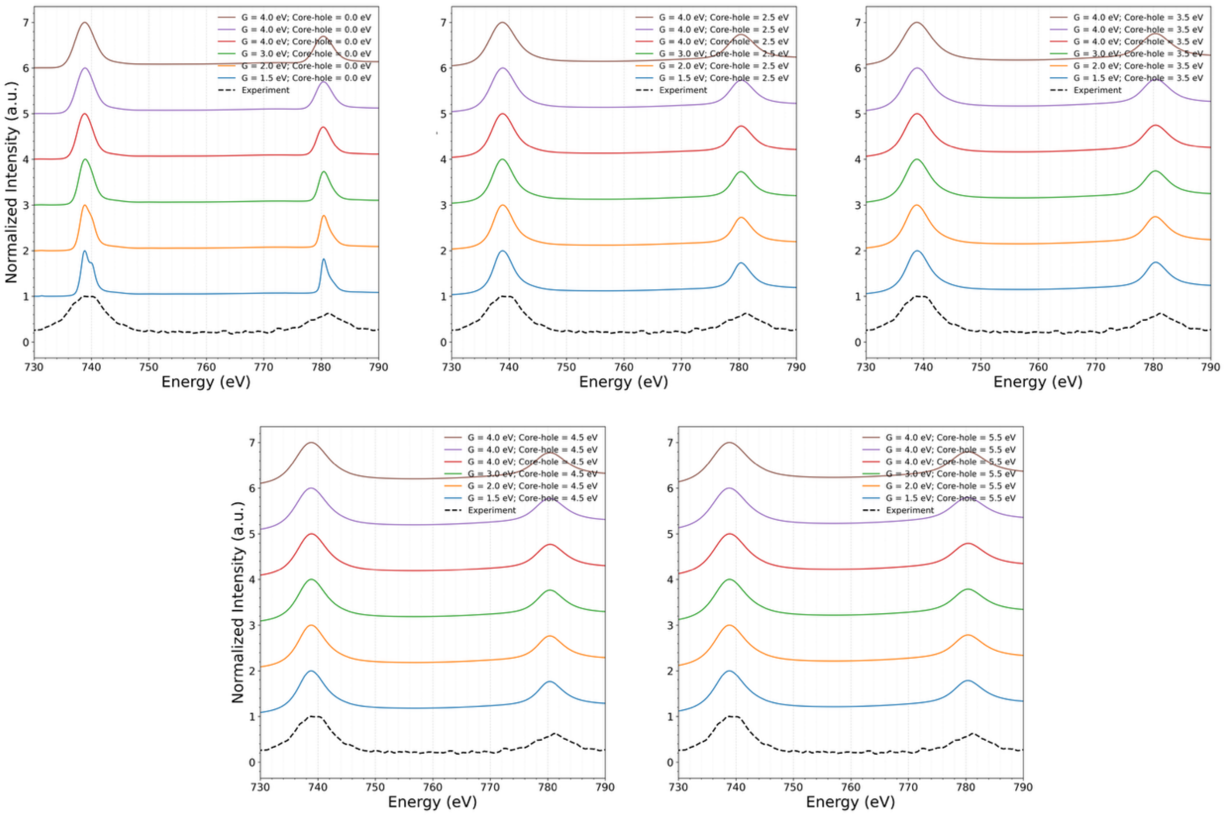

Figure S18: Comparison between experimental EELS and calculated spectra for **KUO$_3$ U *N*$_{4,5}$-edge**. The best agreement is obtained with G = 1.0 eV and a core-hole lifetime of 5.5 eV, which reproduces the edge onset and relative intensity distribution characteristic of U(V) in the layered perovskite structure.

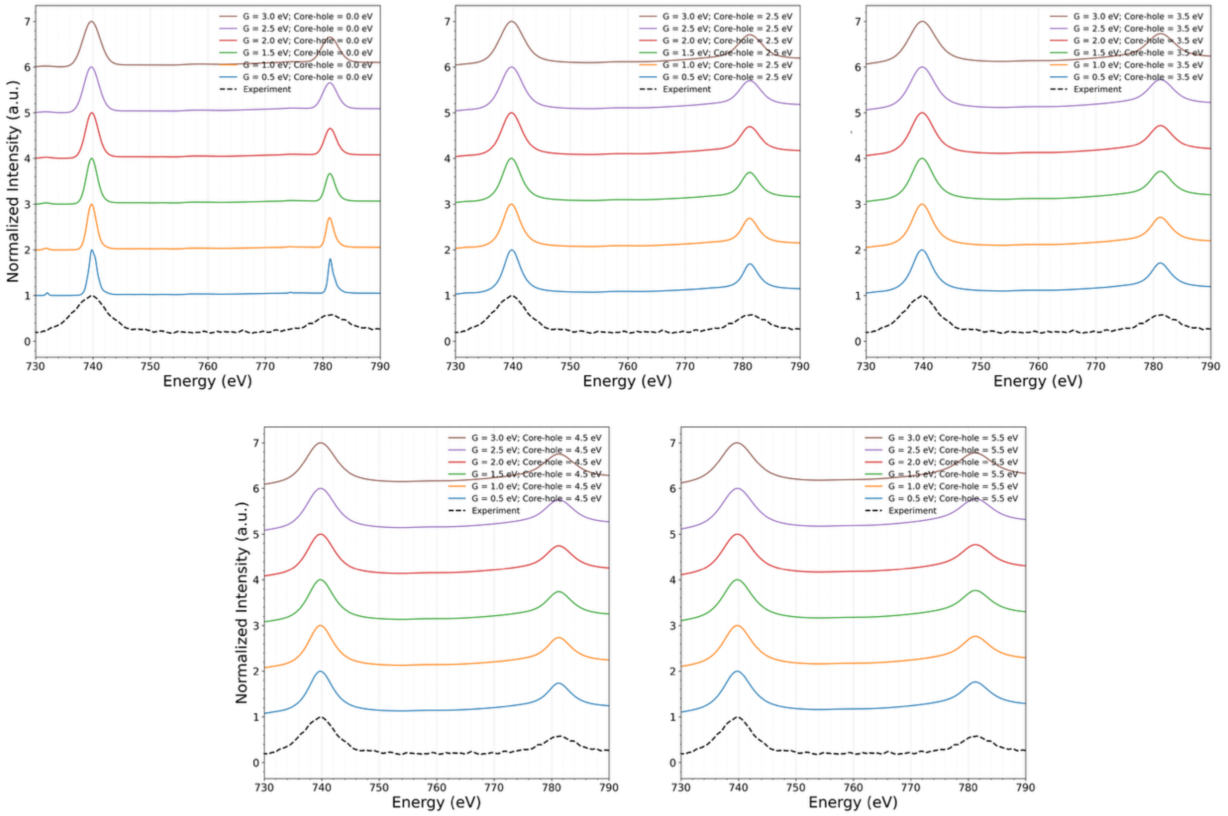

Figure S19: Comparison between experimental EELS and calculated spectra for **U_3O_8 U $N_{4,5}$-edge**. The chosen parameters, G = 1.0 eV, core-hole = 5.5 eV, reproduce the main experimental features.

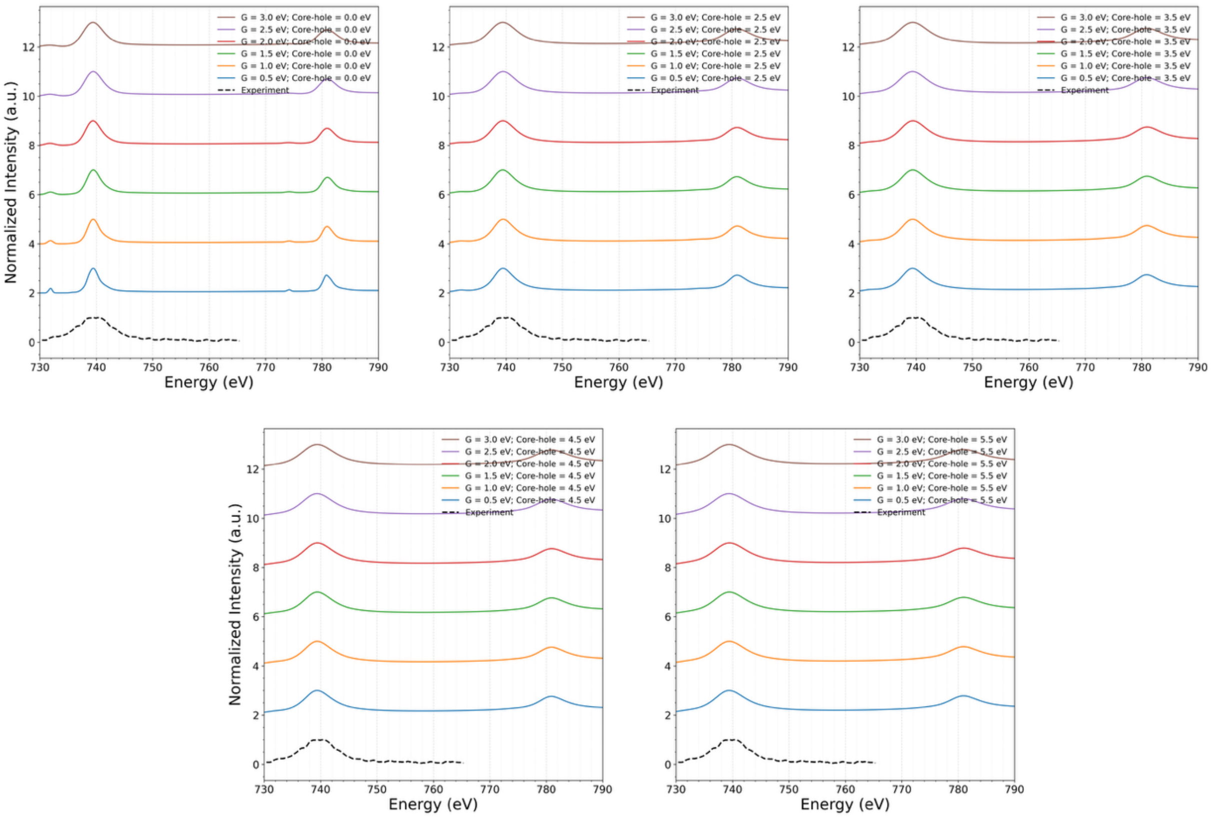

Figure S20: Comparison between experimental EELS and calculated spectra for **$BaUO_4$ U $N_{4,5}$-edge**. The best agreement is achieved with G = 1.0 eV and a core-hole lifetime of 5.5 eV. For $BaUO_4$ at the U $N_{4,5}$-edge, no comparison is possible because the experimental U N_4-edge is overlapped and obscured by the Ba M_4-edge.

Tables

Table S1: Reference uranium compounds selected in this study.

Sample	Phase Composition	Structure type	O/U Ratio	U Valence State(S)
**UO_2**	$UO_{2.02}$	Fluorite	2.02	4+
**U_4O_9**	$U_{64}O_{143}$	Fluorite-type	2.23	4+/5+ (1:1)
**U_3O_7**	$U_{60}O_{140}$	Fluorite-type	2.33	4+/5+ (1:2)
**KUO_3**	KUO_3	Perovskite (archetypal)	3.00	5+
**U_3O_8**	U_6O_{16}	Layered	2.67	5+/6+ (2:1)
**$BaUO_4$**	$Ba_4U_4O_{16}$	Perovskite (distorted)	4.00	6+

Table S2: Annealing conditions for the preparation of reference uranium compounds.

Sample	Temperature (K)	Atmosphere (V/V)	Time (min)	Reference
**UO_2**	973	Ar/5% H_2	60	This work
**U_4O_9**	473	Ar/0.01% O_2	1440	[12]
**U_3O_7**	523	He/0.5% O_2	840	[13]
**KUO_3**	1073	Ar/2.41% H_2 /0.26% O_2	600	[14]
**U_3O_8**	923	N_2/21% O_2	30	[12]
**$BaUO_4$**	1173	N_2/21% O_2	4200	[15]

Table S3: Calculated and experimental **O *K*-edge** peak positions (eV) and normalized intensities for uranium oxides. For each oxide, the first (Peak I) and second (Peak II) maxima, together with their relative intensities (Intensity I and Intensity II, in arbitrary units, a. u.). The Gaussian broadening and core-hole lifetime values used in each calculation are also indicated.

Oxygen *K*-edge										
**Uranium Oxides**	**Parameters**		**Calculation**				**Experiment**			
	Gaussian broadening	Core-hole lifetime	Peak I	Peak II	Intensity I	Intensity II	Peak I	Peak II	Intensity I	Intensity II
	0.50	0.00	533.16	538.36	0.98	1.00	533.14	538.36	0.88	1.00
	1.00	0.00	533.07	538.36	0.96	1.00	533.14	538.36	0.88	1.00
	1.50	0.00	532.76	538.36	0.93	1.00	533.14	538.36	0.88	1.00
	2.00	0.00	532.62	538.36	0.90	1.00	533.14	538.36	0.88	1.00
	2.50	0.00	532.65	538.36	0.88	1.00	533.14	538.36	0.88	1.00
	3.00	0.00	532.66	538.36	0.85	1.00	533.14	538.36	0.88	1.00
	0.50	0.20	533.15	538.36	0.99	1.00	533.14	538.36	0.88	1.00
	1.00	0.20	533.05	538.36	0.94	1.00	533.14	538.36	0.88	1.00
	1.50	0.20	532.76	538.36	0.92	1.00	533.14	538.36	0.88	1.00
	2.00	0.20	532.76	538.36	0.90	1.00	533.14	538.36	0.88	1.00
	2.50	0.20	532.67	538.36	0.87	1.00	533.14	538.36	0.88	1.00
	3.00	0.20	532.66	538.36	0.84	1.00	533.14	538.36	0.88	1.00
	0.50	0.40	533.06	538.36	0.96	1.00	533.14	538.36	0.88	1.00
	1.00	0.40	532.96	538.36	0.92	1.00	533.14	538.36	0.88	1.00
**UO_2**	1.50	0.40	532.76	538.36	0.90	1.00	533.14	538.36	0.88	1.00
	2.00	0.40	532.76	538.36	0.88	1.00	533.14	538.36	0.88	1.00
	2.50	0.40	532.66	538.36	0.86	1.00	533.14	538.36	0.88	1.00
	3.00	0.40	532.67	538.36	0.83	1.00	533.14	538.36	0.88	1.00
	0.50	0.60	533.06	538.36	0.93	1.00	533.14	538.36	0.88	1.00
	1.00	0.60	532.96	538.36	0.91	1.00	533.14	538.36	0.88	1.00
	1.50	0.60	532.76	538.36	0.90	1.00	533.14	538.36	0.88	1.00
	2.00	0.60	532.76	538.36	0.87	1.00	533.14	538.36	0.88	1.00
	2.50	0.60	532.72	538.36	0.84	1.00	533.14	538.36	0.88	1.00
	3.00	0.60	532.76	538.36	0.82	1.00	533.14	538.36	0.88	1.00
	0.50	0.80	533.06	538.36	0.91	1.00	533.14	538.36	0.88	1.00
	1.00	0.80	532.96	538.36	0.89	1.00	533.14	538.36	0.88	1.00
	1.50	0.80	532.76	538.36	0.88	1.00	533.14	538.36	0.88	1.00
	2.00	0.80	532.76	538.36	0.86	1.00	533.14	538.36	0.88	1.00
	2.50	0.80	532.76	538.36	0.84	1.00	533.14	538.36	0.88	1.00
	3.00	0.80	532.72	538.36	0.82	1.00	533.14	538.36	0.88	1.00
	0.50	0.00	533.66	539.26	0.87	1.00	534.23	539.26	0.83	1.00
	1.00	0.00	533.56	539.26	0.86	1.00	534.23	539.26	0.83	1.00
	1.50	0.00	533.76	539.26	0.86	1.00	534.23	539.26	0.83	1.00
	2.00	0.00	533.86	539.26	0.85	1.00	534.23	539.26	0.83	1.00
	2.50	0.00	533.96	539.26	0.85	1.00	534.23	539.26	0.83	1.00
	3.00	0.00	534.26	539.26	0.84	1.00	534.23	539.26	0.83	1.00
	0.50	0.20	533.76	539.26	0.86	1.00	534.23	539.26	0.83	1.00
	1.00	0.20	533.76	539.26	0.86	1.00	534.23	539.26	0.83	1.00
	1.50	0.20	533.76	539.26	0.85	1.00	534.23	539.26	0.83	1.00
	2.00	0.20	533.96	539.26	0.85	1.00	534.23	539.26	0.83	1.00
	2.50	0.20	534.16	539.26	0.84	1.00	534.23	539.26	0.83	1.00
	3.00	0.20	534.36	539.26	0.84	1.00	534.23	539.26	0.83	1.00

	0.50	0.40	533.76	539.26	0.85	1.00	534.23	539.26	0.83	1.00
	1.00	0.40	533.86	539.26	0.85	1.00	534.23	539.26	0.83	1.00
U_4O_9	1.50	0.40	533.96	539.26	0.85	1.00	534.23	539.26	0.83	1.00
	2.00	0.40	534.06	539.26	0.84	1.00	534.23	539.26	0.83	1.00
	2.50	0.40	534.27	539.26	0.84	1.00	534.23	539.26	0.83	1.00
	3.00	0.40	534.46	539.26	0.84	1.00	534.23	539.26	0.83	1.00
	0.50	0.60	533.86	539.26	0.84	1.00	534.23	539.26	0.83	1.00
	1.00	0.60	533.96	539.26	0.84	1.00	534.23	539.26	0.83	1.00
	1.50	0.60	534.06	539.26	0.84	1.00	534.23	539.26	0.83	1.00
	2.00	0.60	534.16	539.26	0.84	1.00	534.23	539.26	0.83	1.00
	2.50	0.60	534.36	539.26	0.84	1.00	534.23	539.26	0.83	1.00
	3.00	0.60	534.76	539.26	0.84	1.00	534.23	539.26	0.83	1.00
	0.50	0.80	533.96	539.26	0.84	1.00	534.23	539.26	0.83	1.00
	1.00	0.80	534.06	539.26	0.84	1.00	534.23	539.26	0.83	1.00
	1.50	0.80	534.16	539.26	0.84	1.00	534.23	539.26	0.83	1.00
	2.00	0.80	534.26	539.26	0.84	1.00	534.23	539.26	0.83	1.00
	2.50	0.80	534.46	539.26	0.84	1.00	534.23	539.26	0.83	1.00
	3.00	0.80	534.71	539.26	0.84	1.00	534.23	539.26	0.83	1.00

	0.50	0.00	533.40	538.90	0.83	1.00	534.76	538.90	0.85	1.00
	1.00	0.00	533.30	538.90	0.80	1.00	534.76	538.90	0.85	1.00
	1.50	0.00	533.50	538.90	0.78	1.00	534.76	538.90	0.85	1.00
	2.00	0.00	533.90	538.90	0.78	1.00	534.76	538.90	0.85	1.00
	2.50	0.00	534.00	538.90	0.79	1.00	534.76	538.90	0.85	1.00
	3.00	0.00	534.04	538.90	0.79	1.00	534.76	538.90	0.85	1.00
	0.50	0.20	533.40	538.90	0.81	1.00	534.76	538.90	0.85	1.00
	1.00	0.20	533.40	538.90	0.79	1.00	534.76	538.90	0.85	1.00
	1.50	0.20	533.70	538.90	0.78	1.00	534.76	538.90	0.85	1.00
	2.00	0.20	533.58	538.90	0.77	1.00	534.76	538.90	0.85	1.00
	2.50	0.20	533.95	538.90	0.78	1.00	534.76	538.90	0.85	1.00
	3.00	0.20	534.27	538.90	0.80	1.00	534.76	538.90	0.85	1.00
	0.50	0.40	533.40	538.90	0.80	1.00	534.76	538.90	0.85	1.00
	1.00	0.40	533.50	538.90	0.78	1.00	534.76	538.90	0.85	1.00
U_3O_7	1.50	0.40	533.41	538.90	0.77	1.00	534.76	538.90	0.85	1.00
	2.00	0.40	533.95	538.90	0.78	1.00	534.76	538.90	0.85	1.00
	2.50	0.40	534.00	538.90	0.78	1.00	534.76	538.90	0.85	1.00
	3.00	0.40	534.07	538.90	0.79	1.00	534.76	538.90	0.85	1.00
	0.50	0.60	533.40	538.90	0.79	1.00	534.76	538.90	0.85	1.00
	1.00	0.60	533.60	538.90	0.78	1.00	534.76	538.90	0.85	1.00
	1.50	0.60	533.80	538.90	0.78	1.00	534.76	538.90	0.85	1.00
	2.00	0.60	533.77	538.90	0.77	1.00	534.76	538.90	0.85	1.00
	2.50	0.60	534.05	538.90	0.79	1.00	534.76	538.90	0.85	1.00
	3.00	0.60	534.00	538.90	0.78	1.00	534.76	538.90	0.85	1.00
	0.50	0.80	533.50	538.90	0.78	1.00	534.76	538.90	0.85	1.00
	1.00	0.80	533.61	538.90	0.77	1.00	534.76	538.90	0.85	1.00
	1.50	0.80	533.80	538.90	0.78	1.00	534.76	538.90	0.85	1.00
	2.00	0.80	534.00	538.90	0.78	1.00	534.76	538.90	0.85	1.00
	2.50	0.80	534.23	538.90	0.79	1.00	534.76	538.90	0.85	1.00
	3.00	0.80	534.14	538.90	0.79	1.00	534.76	538.90	0.85	1.00

	0.50	0.00	534.40	540.33	1.00	0.66	534.40	540.33	1.00	0.77
	1.00	0.00	534.40	540.30	1.00	0.74	534.40	540.33	1.00	0.77
	1.50	0.00	534.40	540.30	1.00	0.80	534.40	540.33	1.00	0.77
	2.00	0.00	534.40	540.31	1.00	0.86	534.40	540.33	1.00	0.77
	2.50	0.00	534.40	540.24	1.00	0.91	534.40	540.33	1.00	0.77

	3.00	0.00	534.40	540.28	1.00	0.95	534.40	540.33	1.00	0.77
	0.50	0.20	534.40	540.34	1.00	0.71	534.40	540.33	1.00	0.77
	1.00	0.20	534.40	540.30	1.00	0.77	534.40	540.33	1.00	0.77
	1.50	0.20	534.40	540.30	1.00	0.83	534.40	540.33	1.00	0.77
	2.00	0.20	534.40	540.27	1.00	0.88	534.40	540.33	1.00	0.77
	2.50	0.20	534.40	540.22	1.00	0.92	534.40	540.33	1.00	0.77
	3.00	0.20	534.40	540.26	1.00	0.96	534.40	540.33	1.00	0.77
	0.50	0.40	534.40	540.35	1.00	0.75	534.40	540.33	1.00	0.77
	1.00	0.40	534.40	540.31	1.00	0.80	534.40	540.33	1.00	0.77
**KUO_3**	1.50	0.40	534.40	540.30	1.00	0.85	534.40	540.33	1.00	0.77
	2.00	0.40	534.40	540.28	1.00	0.90	534.40	540.33	1.00	0.77
	2.50	0.40	534.40	540.21	1.00	0.94	534.40	540.33	1.00	0.77
	3.00	0.40	534.40	540.20	1.00	0.97	534.40	540.33	1.00	0.77
	0.50	0.60	534.40	540.40	1.00	0.79	534.40	540.33	1.00	0.77
	1.00	0.60	534.40	540.30	1.00	0.83	534.40	540.33	1.00	0.77
	1.50	0.60	534.40	540.30	1.00	0.87	534.40	540.33	1.00	0.77
	2.00	0.60	534.40	540.21	1.00	0.91	534.40	540.33	1.00	0.77
	2.50	0.60	534.40	540.21	1.00	0.95	534.40	540.33	1.00	0.77
	3.00	0.60	534.40	540.21	1.00	0.97	534.40	540.33	1.00	0.77
	0.50	0.80	534.40	540.27	1.00	0.82	534.40	540.33	1.00	0.77
	1.00	0.80	534.40	540.31	1.00	0.85	534.40	540.33	1.00	0.77
	1.50	0.80	534.40	540.27	1.00	0.90	534.40	540.33	1.00	0.77
	2.00	0.80	534.40	540.21	1.00	0.93	534.40	540.33	1.00	0.77
	2.50	0.80	534.40	540.16	1.00	0.96	534.40	540.33	1.00	0.77
	3.00	0.80	534.40	540.10	1.00	0.98	534.40	540.33	1.00	0.77
	0.50	0.00	535.12	543.53	1.00	0.58	535.12	541.60	1.00	0.77
	1.00	0.00	535.12	543.57	1.00	0.64	535.12	541.60	1.00	0.77
	1.50	0.00	535.12	543.72	1.00	0.68	535.12	541.60	1.00	0.77
	2.00	0.00	535.12	543.81	1.00	0.82	535.12	541.60	1.00	0.77
	2.50	0.00	535.12	543.92	1.00	0.75	535.12	541.60	1.00	0.77
	3.00	0.00	543.12	543.82	1.00	0.78	535.12	541.60	1.00	0.77
	0.50	0.20	535.12	543.52	1.00	0.62	535.12	541.60	1.00	0.77
	1.00	0.20	535.12	543.56	1.00	0.67	535.12	541.60	1.00	0.77
	1.50	0.20	535.12	543.72	1.00	0.70	535.12	541.60	1.00	0.77
	2.00	0.20	535.12	543.81	1.00	0.74	535.12	541.60	1.00	0.77
	2.50	0.20	535.12	543.78	1.00	0.77	535.12	541.60	1.00	0.77
	3.00	0.20	535.12	543.73	1.00	0.80	535.12	541.60	1.00	0.77
	0.50	0.40	535.12	543.52	1.00	0.65	535.12	541.60	1.00	0.77
	1.00	0.40	535.12	543.67	1.00	0.69	535.12	541.60	1.00	0.77
**U_3O_8**	1.50	0.40	535.12	543.72	1.00	0.72	535.12	541.60	1.00	0.77
	2.00	0.40	535.12	543.80	1.00	0.76	535.12	541.60	1.00	0.77
	2.50	0.40	535.12	543.74	1.00	0.79	535.12	541.60	1.00	0.77
	3.00	0.40	535.12	543.70	1.00	0.81	535.12	541.60	1.00	0.77
	0.50	0.60	535.12	543.52	1.00	0.68	535.12	541.60	1.00	0.77
	1.00	0.60	535.12	543.62	1.00	0.71	535.12	541.60	1.00	0.77
	1.50	0.60	535.12	543.72	1.00	0.74	535.12	541.60	1.00	0.77
	2.00	0.60	535.12	543.77	1.00	0.77	535.12	541.60	1.00	0.77
	2.50	0.60	535.12	543.72	1.00	0.80	535.12	541.60	1.00	0.77
	3.00	0.60	535.12	543.69	1.00	0.82	535.12	541.60	1.00	0.77
	0.50	0.80	535.12	543.51	1.00	0.71	535.12	541.60	1.00	0.77
	1.00	0.80	535.12	543.67	1.00	0.73	535.12	541.60	1.00	0.77
	1.50	0.80	535.12	543.71	1.00	0.76	535.12	541.60	1.00	0.77
	2.00	0.80	535.12	543.77	1.00	0.79	535.12	541.60	1.00	0.77
	2.50	0.80	535.12	543.75	1.00	0.81	535.12	541.60	1.00	0.77

	3.00	0.80	535.12	543.56	1.00	0.84	535.12	541.60	1.00	0.77
	0.50	0.00	534.94	541.44	1.00	0.70	534.94	539.80	1.00	0.80
	1.00	0.00	534.94	541.44	1.00	0.70	534.94	539.80	1.00	0.80
	1.50	0.00	534.94	541.40	1.00	0.70	534.94	539.80	1.00	0.80
	2.00	0.00	534.94	541.40	1.00	0.70	534.94	539.80	1.00	0.80
	2.50	0.00	534.94	541.34	1.00	0.71	534.94	539.80	1.00	0.80
	3.00	0.00	534.94	541.48	1.00	0.71	534.94	539.80	1.00	0.80
	0.50	0.20	534.94	541.45	1.00	0.71	534.94	539.80	1.00	0.80
	1.00	0.20	534.94	541.42	1.00	0.71	534.94	539.80	1.00	0.80
	1.50	0.20	534.94	541.39	1.00	0.71	534.94	539.80	1.00	0.80
	2.00	0.20	534.94	541.37	1.00	0.71	534.94	539.80	1.00	0.80
	2.50	0.20	534.94	541.33	1.00	0.71	534.94	539.80	1.00	0.80
	3.00	0.20	534.94	541.40	1.00	0.72	534.94	539.80	1.00	0.80
	0.50	0.40	534.94	541.42	1.00	0.71	534.94	539.80	1.00	0.80
	1.00	0.40	534.94	541.39	1.00	0.71	534.94	539.80	1.00	0.80
**$BaUO_4$**	1.50	0.40	534.94	541.38	1.00	0.71	534.94	539.80	1.00	0.80
	2.00	0.40	534.94	541.30	1.00	0.72	534.94	539.80	1.00	0.80
	2.50	0.40	534.94	541.31	1.00	0.72	534.94	539.80	1.00	0.80
	3.00	0.40	534.94	541.40	1.00	0.73	534.94	539.80	1.00	0.80
	0.50	0.60	534.94	541.39	1.00	0.72	534.94	539.80	1.00	0.80
	1.00	0.60	534.94	541.38	1.00	0.72	534.94	539.80	1.00	0.80
	1.50	0.60	534.94	541.33	1.00	0.72	534.94	539.80	1.00	0.80
	2.00	0.60	534.94	541.31	1.00	0.73	534.94	539.80	1.00	0.80
	2.50	0.60	534.94	541.25	1.00	0.73	534.94	539.80	1.00	0.80
	3.00	0.60	534.94	541.20	1.00	0.74	534.94	539.80	1.00	0.80
	0.50	0.80	534.94	541.38	1.00	0.73	534.94	539.80	1.00	0.80
	1.00	0.80	534.94	541.31	1.00	0.73	534.94	539.80	1.00	0.80
	1.50	0.80	534.94	541.23	1.00	0.73	534.94	539.80	1.00	0.80
	2.00	0.80	534.94	541.17	1.00	0.74	534.94	539.80	1.00	0.80
	2.50	0.80	534.94	541.18	1.00	0.74	534.94	539.80	1.00	0.80
	3.00	0.80	534.94	541.18	1.00	0.75	534.94	539.80	1.00	0.80

Table S4: Calculated and experimental **U $N_{6,7}$-edge** peak positions (eV) and normalized intensities for uranium oxides. For each oxide, the first (Peak I) and second (Peak II) maxima, together with their relative intensities (Intensity I and Intensity II, in arbitrary units, a. u.). The Gaussian broadening and core-hole lifetime values used in each calculation are also indicated.

Uranium $N_{6,7}$-edge

Uranium Oxides	Parameters		Calculation				Experiment			
	Gaussian broadening	Core-hole lifetime	Peak I	Peak II	Intensity I	Intensity II	Peak I	Peak II	Intensity I	Intensity II
UO_2	0.50	0.00	385.55	393.10	0.79	1.00	382.12	393.10	0.53	1.00
	1.00	0.00	385.54	393.10	0.80	1.00	382.12	393.10	0.53	1.00
	1.50	0.00	385.46	393.10	0.81	1.00	382.12	393.10	0.53	1.00
	2.00	0.00	385.19	393.10	0.81	1.00	382.12	393.10	0.53	1.00
	2.50	0.00	384.81	393.10	0.81	1.00	382.12	393.10	0.53	1.00
	3.00	0.00	384.33	393.10	0.80	1.00	382.12	393.10	0.53	1.00
	0.50	0.30	385.50	393.10	0.80	1.00	382.12	393.10	0.53	1.00
	1.00	0.30	385.46	393.10	0.81	1.00	382.12	393.10	0.53	1.00
	1.50	0.30	385.30	393.10	0.81	1.00	382.12	393.10	0.53	1.00
	2.00	0.30	385.15	393.10	0.82	1.00	382.12	393.10	0.53	1.00
	2.50	0.30	384.63	393.10	0.81	1.00	382.12	393.10	0.53	1.00
	3.00	0.30	384.20	393.10	0.80	1.00	382.12	393.10	0.53	1.00
	0.50	0.40	385.50	393.10	0.80	1.00	382.12	393.10	0.53	1.00
	1.00	0.40	385.41	393.10	0.81	1.00	382.12	393.10	0.53	1.00
	1.50	0.40	385.26	393.10	0.82	1.00	382.12	393.10	0.53	1.00
	2.00	0.40	385.00	393.10	0.81	1.00	382.12	393.10	0.53	1.00
	2.50	0.40	384.60	393.10	0.81	1.00	382.12	393.10	0.53	1.00
	3.00	0.40	384.16	393.10	0.80	1.00	382.12	393.10	0.53	1.00
	0.50	0.50	385.44	393.10	0.81	1.00	382.12	393.10	0.53	1.00
	1.00	0.50	385.40	393.10	0.81	1.00	382.12	393.10	0.53	1.00
	1.50	0.50	385.23	393.10	0.82	1.00	382.12	393.10	0.53	1.00
	2.00	0.50	384.96	393.10	0.81	1.00	382.12	393.10	0.53	1.00
	2.50	0.50	384.50	393.10	0.81	1.00	382.12	393.10	0.53	1.00
	3.00	0.50	384.06	393.10	0.80	1.00	382.12	393.10	0.53	1.00
	0.50	0.60	385.40	393.10	0.81	1.00	382.12	393.10	0.53	1.00
	1.00	0.60	385.36	393.10	0.81	1.00	382.12	393.10	0.53	1.00
	1.50	0.60	385.20	393.10	0.82	1.00	382.12	393.10	0.53	1.00
	2.00	0.60	385.00	393.10	0.81	1.00	382.12	393.10	0.53	1.00
	2.50	0.60	384.50	393.10	0.80	1.00	382.12	393.10	0.53	1.00
	3.00	0.60	384.04	393.10	0.80	1.00	382.12	393.10	0.53	1.00
	0.50	0.00	386.34	394.36	0.83	1.00	382.84	394.36	0.88	1.00
	1.00	0.00	383.16	394.36	0.83	1.00	382.84	394.36	0.88	1.00
	1.50	0.00	383.24	394.36	0.82	1.00	382.84	394.36	0.88	1.00
	2.00	0.00	384.72	394.36	0.81	1.00	382.84	394.36	0.88	1.00
	2.50	0.00	384.81	394.36	0.80	1.00	382.84	394.36	0.88	1.00
	3.00	0.00	384.80	394.36	0.80	1.00	382.84	394.36	0.88	1.00
	0.50	0.30	384.00	394.36	0.83	1.00	382.84	394.36	0.88	1.00
	1.00	0.30	384.10	394.36	0.82	1.00	382.84	394.36	0.88	1.00
	1.50	0.30	384.40	394.36	0.82	1.00	382.84	394.36	0.88	1.00
	2.00	0.30	384.78	394.36	0.81	1.00	382.84	394.36	0.88	1.00
	2.50	0.30	384.76	394.36	0.80	1.00	382.84	394.36	0.88	1.00
	3.00	0.30	384.77	394.36	0.79	1.00	382.84	394.36	0.88	1.00

	0.50	0.40	384.00	394.36	0.83	1.00	382.84	394.36	0.88	1.00
	1.00	0.40	384.19	394.36	0.83	1.00	382.84	394.36	0.88	1.00
U_4O_9	1.50	0.40	384.49	394.36	0.82	1.00	382.84	394.36	0.88	1.00
	2.00	0.40	384.77	394.36	0.81	1.00	382.84	394.36	0.88	1.00
	2.50	0.40	384.76	394.36	0.80	1.00	382.84	394.36	0.88	1.00
	3.00	0.40	384.77	394.36	0.79	1.00	382.84	394.36	0.88	1.00
	0.50	0.50	384.07	394.36	0.82	1.00	382.84	394.36	0.88	1.00
	1.00	0.50	384.21	394.36	0.82	1.00	382.84	394.36	0.88	1.00
	1.50	0.50	384.46	394.36	0.81	1.00	382.84	394.36	0.88	1.00
	2.00	0.50	384.77	394.36	0.80	1.00	382.84	394.36	0.88	1.00
	2.50	0.50	384.76	394.36	0.79	1.00	382.84	394.36	0.88	1.00
	3.00	0.50	384.77	394.36	0.79	1.00	382.84	394.36	0.88	1.00
	0.50	0.60	384.07	394.36	0.82	1.00	382.84	394.36	0.88	1.00
	1.00	0.60	384.30	394.36	0.82	1.00	382.84	394.36	0.88	1.00
	1.50	0.60	384.56	394.36	0.82	1.00	382.84	394.36	0.88	1.00
	2.00	0.60	384.74	394.36	0.80	1.00	382.84	394.36	0.88	1.00
	2.50	0.60	384.76	394.36	0.79	1.00	382.84	394.36	0.88	1.00
	3.00	0.60	384.75	394.36	0.79	1.00	382.84	394.36	0.88	1.00
	0.50	0.00	383.94	394.54	0.83	1.00	382.84	394.54	0.57	1.00
	1.00	0.00	384.14	394.54	0.82	1.00	382.84	394.54	0.57	1.00
	1.50	0.00	384.50	394.54	0.81	1.00	382.84	394.54	0.57	1.00
	2.00	0.00	384.60	394.54	0.80	1.00	382.84	394.54	0.57	1.00
	2.50	0.00	384.69	394.54	0.79	1.00	382.84	394.54	0.57	1.00
	3.00	0.00	384.67	394.54	0.79	1.00	382.84	394.54	0.57	1.00
	0.50	0.30	384.05	394.54	0.82	1.00	382.84	394.54	0.57	1.00
	1.00	0.30	384.38	394.54	0.82	1.00	382.84	394.54	0.57	1.00
	1.50	0.30	384.60	394.54	0.81	1.00	382.84	394.54	0.57	1.00
	2.00	0.30	384.58	394.54	0.80	1.00	382.84	394.54	0.57	1.00
	2.50	0.30	384.70	394.54	0.79	1.00	382.84	394.54	0.57	1.00
	3.00	0.30	384.68	394.54	0.78	1.00	382.84	394.54	0.57	1.00
	0.50	0.40	384.04	394.54	0.82	1.00	382.84	394.54	0.57	1.00
	1.00	0.40	384.39	394.54	0.81	1.00	382.84	394.54	0.57	1.00
U_3O_7	1.50	0.40	384.60	394.54	0.80	1.00	382.84	394.54	0.57	1.00
	2.00	0.40	384.71	394.54	0.80	1.00	382.84	394.54	0.57	1.00
	2.50	0.40	384.69	394.54	0.79	1.00	382.84	394.54	0.57	1.00
	3.00	0.40	384.73	394.54	0.78	1.00	382.84	394.54	0.57	1.00
	0.50	0.50	384.14	394.54	0.82	1.00	382.84	394.54	0.57	1.00
	1.00	0.50	384.49	394.54	0.81	1.00	382.84	394.54	0.57	1.00
	1.50	0.50	383.64	394.54	0.80	1.00	382.84	394.54	0.57	1.00
	2.00	0.50	384.79	394.54	0.79	1.00	382.84	394.54	0.57	1.00
	2.50	0.50	384.73	394.54	0.79	1.00	382.84	394.54	0.57	1.00
	3.00	0.50	384.72	394.54	0.78	1.00	382.84	394.54	0.57	1.00
	0.50	0.60	384.26	394.54	0.81	1.00	382.84	394.54	0.57	1.00
	1.00	0.60	384.47	394.54	0.81	1.00	382.84	394.54	0.57	1.00
	1.50	0.60	384.62	394.54	0.80	1.00	382.84	394.54	0.57	1.00
	2.00	0.60	384.73	394.54	0.79	1.00	382.84	394.54	0.57	1.00
	2.50	0.60	384.69	394.54	0.79	1.00	382.84	394.54	0.57	1.00
	3.00	0.60	384.73	394.54	0.78	1.00	382.84	394.54	0.57	1.00
	0.50	0.00	383.44	394.18	0.71	1.00	382.49	394.18	0.86	1.00
	1.00	0.00	383.58	394.18	0.68	1.00	382.49	394.18	0.86	1.00
	1.50	0.00	383.71	394.18	0.65	1.00	382.49	394.18	0.86	1.00
	2.00	0.00	383.77	394.18	0.62	1.00	382.49	394.18	0.86	1.00
	2.50	0.00	383.84	394.18	0.59	1.00	382.49	394.18	0.86	1.00

	3.00	0.00	384.04	394.18	0.57	1.00	382.49	394.18	0.86	1.00
	0.50	0.30	383.55	394.18	0.68	1.00	382.49	394.18	0.86	1.00
	1.00	0.30	383.68	394.18	0.65	1.00	382.49	394.18	0.86	1.00
	1.50	0.30	383.72	394.18	0.63	1.00	382.49	394.18	0.86	1.00
	2.00	0.30	383.79	394.18	0.60	1.00	382.49	394.18	0.86	1.00
	2.50	0.30	383.97	394.18	0.58	1.00	382.49	394.18	0.86	1.00
	3.00	0.30	384.06	394.18	0.56	1.00	382.49	394.18	0.86	1.00
	0.50	0.40	383.55	394.18	0.67	1.00	382.49	394.18	0.86	1.00
	1.00	0.40	383.67	394.18	0.64	1.00	382.49	394.18	0.86	1.00
**KUO_3**	1.50	0.40	383.72	394.18	0.62	1.00	382.49	394.18	0.86	1.00
	2.00	0.40	383.78	394.18	0.60	1.00	382.49	394.18	0.86	1.00
	2.50	0.40	383.98	394.18	0.58	1.00	382.49	394.18	0.86	1.00
	3.00	0.40	384.07	394.18	0.56	1.00	382.49	394.18	0.86	1.00
	0.50	0.50	383.54	394.18	0.66	1.00	382.49	394.18	0.86	1.00
	1.00	0.50	383.69	394.18	0.64	1.00	382.49	394.18	0.86	1.00
	1.50	0.50	383.73	394.18	0.62	1.00	382.49	394.18	0.86	1.00
	2.00	0.50	383.90	394.18	0.59	1.00	382.49	394.18	0.86	1.00
	2.50	0.50	383.97	394.18	0.57	1.00	382.49	394.18	0.86	1.00
	3.00	0.50	384.06	394.18	0.56	1.00	382.49	394.18	0.86	1.00
	0.50	0.60	383.56	394.18	0.65	1.00	382.49	394.18	0.86	1.00
	1.00	0.60	383.69	394.18	0.63	1.00	382.49	394.18	0.86	1.00
	1.50	0.60	383.74	394.18	0.61	1.00	382.49	394.18	0.86	1.00
	2.00	0.60	383.90	394.18	0.59	1.00	382.49	394.18	0.86	1.00
	2.50	0.60	384.00	394.18	0.57	1.00	382.49	394.18	0.86	1.00
	3.00	0.60	384.07	394.18	0.55	1.00	382.49	394.18	0.86	1.00

	0.50	0.00	386.39	395.99	0.64	1.00	383.22	395.99	0.45	1.00
	1.00	0.00	385.93	395.99	0.64	1.00	383.22	395.99	0.45	1.00
	1.50	0.00	385.74	395.99	0.63	1.00	383.22	395.99	0.45	1.00
	2.00	0.00	385.63	395.99	0.63	1.00	383.22	395.99	0.45	1.00
	2.50	0.00	385.69	395.99	0.62	1.00	383.22	395.99	0.45	1.00
	3.00	0.00	385.75	395.99	0.62	1.00	383.22	395.99	0.45	1.00
	0.50	0.30	386.12	395.99	0.64	1.00	383.22	395.99	0.45	1.00
	1.00	0.30	385.88	395.99	0.63	1.00	383.22	395.99	0.45	1.00
	1.50	0.30	385.78	395.99	0.63	1.00	383.22	395.99	0.45	1.00
	2.00	0.30	385.70	395.99	0.62	1.00	383.22	395.99	0.45	1.00
	2.50	0.30	385.72	395.99	0.62	1.00	383.22	395.99	0.45	1.00
	3.00	0.30	385.77	395.99	0.71	1.00	383.22	395.99	0.45	1.00
	0.50	0.40	386.01	395.99	0.63	1.00	383.22	395.99	0.45	1.00
	1.00	0.40	385.79	395.99	0.63	1.00	383.22	395.99	0.45	1.00
**U_3O_8**	1.50	0.40	385.74	395.99	0.63	1.00	383.22	395.99	0.45	1.00
	2.00	0.40	385.72	395.99	0.62	1.00	383.22	395.99	0.45	1.00
	2.50	0.40	385.80	395.99	0.62	1.00	383.22	395.99	0.45	1.00
	3.00	0.40	385.79	395.99	0.61	1.00	383.22	395.99	0.45	1.00
	0.50	0.50	385.89	395.99	0.63	1.00	383.22	395.99	0.45	1.00
	1.00	0.50	385.86	395.99	0.63	1.00	383.22	395.99	0.45	1.00
	1.50	0.50	385.80	395.99	0.62	1.00	383.22	395.99	0.45	1.00
	2.00	0.50	385.70	395.99	0.62	1.00	383.22	395.99	0.45	1.00
	2.50	0.50	385.87	395.99	0.62	1.00	383.22	395.99	0.45	1.00
	3.00	0.50	385.88	395.99	0.62	1.00	383.22	395.99	0.45	1.00
	0.50	0.60	385.95	395.99	0.63	1.00	383.22	395.99	0.45	1.00
	1.00	0.60	385.81	395.99	0.63	1.00	383.22	395.99	0.45	1.00
	1.50	0.60	385.81	395.99	0.63	1.00	383.22	395.99	0.45	1.00
	2.00	0.60	385.74	395.99	0.62	1.00	383.22	395.99	0.45	1.00
	2.50	0.60	385.83	395.99	0.62	1.00	383.22	395.99	0.45	1.00

	3.00	0.60	385.91	395.99	0.62	1.00	383.22	395.99	0.45	1.00
**BaUO$_4$**	0.50	0.00	386.42	397.06	0.57	1.00	383.43	397.06	0.60	1.00
	1.00	0.00	386.45	397.06	0.58	1.00	383.43	397.06	0.60	1.00
	1.50	0.00	386.44	397.06	0.58	1.00	383.43	397.06	0.60	1.00
	2.00	0.00	386.47	397.06	0.57	1.00	383.43	397.06	0.60	1.00
	2.50	0.00	386.51	397.06	0.57	1.00	383.43	397.06	0.60	1.00
	3.00	0.00	386.55	397.06	0.57	1.00	383.43	397.06	0.60	1.00
	0.50	0.30	386.45	397.06	0.57	1.00	383.43	397.06	0.60	1.00
	1.00	0.30	386.39	397.06	0.57	1.00	383.43	397.06	0.60	1.00
	1.50	0.30	386.48	397.06	0.57	1.00	383.43	397.06	0.60	1.00
	2.00	0.30	386.47	397.06	0.57	1.00	383.43	397.06	0.60	1.00
	2.50	0.30	386.56	397.06	0.57	1.00	383.43	397.06	0.60	1.00
	3.00	0.30	386.64	397.06	0.57	1.00	383.43	397.06	0.60	1.00
	0.50	0.40	386.46	397.06	0.57	1.00	383.43	397.06	0.60	1.00
	1.00	0.40	386.43	397.06	0.57	1.00	383.43	397.06	0.60	1.00
	1.50	0.40	386.40	397.06	0.57	1.00	383.43	397.06	0.60	1.00
	2.00	0.40	386.51	397.06	0.57	1.00	383.43	397.06	0.60	1.00
	2.50	0.40	386.47	397.06	0.57	1.00	383.43	397.06	0.60	1.00
	3.00	0.40	386.69	397.06	0.57	1.00	383.43	397.06	0.60	1.00
	0.50	0.50	386.50	397.06	0.57	1.00	383.43	397.06	0.60	1.00
	1.00	0.50	386.47	397.06	0.58	1.00	383.43	397.06	0.60	1.00
	1.50	0.50	386.47	397.06	0.58	1.00	383.43	397.06	0.60	1.00
	2.00	0.50	386.51	397.06	0.57	1.00	383.43	397.06	0.60	1.00
	2.50	0.50	386.54	397.06	0.57	1.00	383.43	397.06	0.60	1.00
	3.00	0.50	386.57	397.06	0.57	1.00	383.43	397.06	0.60	1.00
	0.50	0.60	386.57	397.06	0.57	1.00	383.43	397.06	0.60	1.00
	1.00	0.60	386.49	397.06	0.57	1.00	383.43	397.06	0.60	1.00
	1.50	0.60	386.48	397.06	0.58	1.00	383.43	397.06	0.60	1.00
	2.00	0.60	386.61	397.06	0.57	1.00	383.43	397.06	0.60	1.00
	2.50	0.60	386.61	397.06	0.58	1.00	383.43	397.06	0.60	1.00
	3.00	0.60	386.61	397.06	0.57	1.00	383.43	397.06	0.60	1.00

Table S5: Calculated and experimental **U $N_{4,5}$-edge** peak positions (eV) and normalized intensities for uranium oxides. For each oxide, the first (Peak I) and second (Peak II) maxima, together with their relative intensities (Intensity I and Intensity II, in arbitrary units, a. u.). The Gaussian broadening and core-hole lifetime values used in each calculation are also indicated.

Uranium $N_{4,5}$-edge											
**Uranium Oxides**	**Parameters**		**Calculation**				**Experiment**				
	Gaussian broadening	Core-hole lifetime	Peak I	Peak II	Intensity I	Intensity II	Peak I	Peak II	Intensity I	Intensity II	
	0.50	0.00	738.88	779.90	1.00	0.76	738.88	780.20	1.00	0.55	
	1.00	0.00	738.88	780.09	1.00	0.66	738.88	780.20	1.00	0.55	
	1.50	0.00	738.88	780.20	1.00	0.63	738.88	780.20	1.00	0.55	
	2.00	0.00	738.88	780.29	1.00	0.62	738.88	780.20	1.00	0.55	
	2.50	0.00	738.88	780.29	1.00	0.62	738.88	780.20	1.00	0.55	
	3.00	0.00	738.88	780.32	1.00	0.62	738.88	780.20	1.00	0.55	
	0.50	2.50	738.88	780.24	1.00	0.64	738.88	780.20	1.00	0.55	
	1.00	2.50	738.88	780.35	1.00	0.64	738.88	780.20	1.00	0.55	
	1.50	2.50	738.88	780.39	1.00	0.64	738.88	780.20	1.00	0.55	
	2.00	2.50	738.88	780.39	1.00	0.65	738.88	780.20	1.00	0.55	
	2.50	2.50	738.88	780.42	1.00	0.65	738.88	780.20	1.00	0.55	
	3.00	2.50	738.88	780.45	1.00	0.66	738.88	780.20	1.00	0.55	
	0.50	3.50	738.88	780.36	1.00	0.66	738.88	780.20	1.00	0.55	
	1.00	3.50	738.88	780.38	1.00	0.66	738.88	780.20	1.00	0.55	
**UO_2**	1.50	3.50	738.88	780.39	1.00	0.66	738.88	780.20	1.00	0.55	
	2.00	3.50	738.88	780.39	1.00	0.66	738.88	780.20	1.00	0.55	
	2.50	3.50	738.88	780.43	1.00	0.67	738.88	780.20	1.00	0.55	
	3.00	3.50	738.88	780.45	1.00	0.68	738.88	780.20	1.00	0.55	
	0.50	4.50	738.88	780.38	1.00	0.68	738.88	780.20	1.00	0.55	
	1.00	4.50	738.88	780.40	1.00	0.68	738.88	780.20	1.00	0.55	
	1.50	4.50	738.88	780.41	1.00	0.68	738.88	780.20	1.00	0.55	
	2.00	4.50	738.88	780.44	1.00	0.69	738.88	780.20	1.00	0.55	
	2.50	4.50	738.88	780.43	1.00	0.69	738.88	780.20	1.00	0.55	
	3.00	4.50	738.88	780.45	1.00	0.70	738.88	780.20	1.00	0.55	
	0.50	5.50	738.88	780.41	1.00	0.70	738.88	780.20	1.00	0.55	
	1.00	5.50	738.88	780.42	1.00	0.70	738.88	780.20	1.00	0.55	
	1.50	5.50	738.88	780.42	1.00	0.70	738.88	780.20	1.00	0.55	
	2.00	5.50	738.88	780.44	1.00	0.71	738.88	780.20	1.00	0.55	
	2.50	5.50	738.88	780.46	1.00	0.71	738.88	780.20	1.00	0.55	
	3.00	5.50	738.88	780.47	1.00	0.72	738.88	780.20	1.00	0.55	
	0.50	0.00	738.88	780.47	1.00	0.72	738.88	780.26	1.00	0.55	
	1.00	0.00	738.88	780.47	1.00	0.72	738.88	780.26	1.00	0.55	
	1.50	0.00	738.88	780.47	1.00	0.72	738.88	780.26	1.00	0.55	
	2.00	0.00	738.88	780.49	1.00	0.72	738.88	780.26	1.00	0.55	
	2.50	0.00	738.88	780.45	1.00	0.72	738.88	780.26	1.00	0.55	
	3.00	0.00	738.88	780.46	1.00	0.72	738.88	780.26	1.00	0.55	
	0.50	2.50	738.88	780.48	1.00	0.72	738.88	780.26	1.00	0.55	
	1.00	2.50	738.88	780.49	1.00	0.71	738.88	780.26	1.00	0.55	
	1.50	2.50	738.88	780.48	1.00	0.72	738.88	780.26	1.00	0.55	
	2.00	2.50	738.88	780.46	1.00	0.72	738.88	780.26	1.00	0.55	

	2.50	2.50	738.88	780.46	1.00	0.72	738.88	780.26	1.00	0.55
	3.00	2.50	738.88	780.47	1.00	0.72	738.88	780.26	1.00	0.55
	0.50	3.50	738.88	780.47	1.00	0.72	738.88	780.26	1.00	0.55
	1.00	3.50	738.88	780.48	1.00	0.72	738.88	780.26	1.00	0.55
U_4O_9	1.50	3.50	738.88	780.46	1.00	0.72	738.88	780.26	1.00	0.55
	2.00	3.50	738.88	780.51	1.00	0.71	738.88	780.26	1.00	0.55
	2.50	3.50	738.88	780.46	1.00	0.72	738.88	780.26	1.00	0.55
	3.00	3.50	738.88	780.45	1.00	0.72	738.88	780.26	1.00	0.55
	0.50	4.50	738.88	780.45	1.00	0.72	738.88	780.26	1.00	0.55
	1.00	4.50	738.88	780.46	1.00	0.72	738.88	780.26	1.00	0.55
	1.50	4.50	738.88	780.47	1.00	0.72	738.88	780.26	1.00	0.55
	2.00	4.50	738.88	780.48	1.00	0.72	738.88	780.26	1.00	0.55
	2.50	4.50	738.88	780.48	1.00	0.72	738.88	780.26	1.00	0.55
	3.00	4.50	738.88	780.45	1.00	0.72	738.88	780.26	1.00	0.55
	0.50	5.50	738.88	780.47	1.00	0.72	738.88	780.26	1.00	0.55
	1.00	5.50	738.88	780.48	1.00	0.72	738.88	780.26	1.00	0.55
	1.50	5.50	738.88	780.46	1.00	0.71	738.88	780.26	1.00	0.55
	2.00	5.50	738.88	780.46	1.00	0.71	738.88	780.26	1.00	0.55
	2.50	5.50	738.88	780.48	1.00	0.72	738.88	780.26	1.00	0.55
	3.00	5.50	738.88	780.47	1.00	0.72	738.88	780.26	1.00	0.55
	0.50	0.00	740.14	781.64	1.00	0.69	740.14	781.54	1.00	0.54
	1.00	0.00	740.14	781.64	1.00	0.68	740.14	781.54	1.00	0.54
	1.50	0.00	740.14	781.64	1.00	0.67	740.14	781.54	1.00	0.54
	2.00	0.00	740.14	781.65	1.00	0.67	740.14	781.54	1.00	0.54
	2.50	0.00	740.14	781.65	1.00	0.67	740.14	781.54	1.00	0.54
	3.00	0.00	740.14	781.59	1.00	0.67	740.14	781.54	1.00	0.54
	0.50	2.50	740.14	781.62	1.00	0.70	740.14	781.54	1.00	0.54
	1.00	2.50	740.14	781.64	1.00	0.70	740.14	781.54	1.00	0.54
	1.50	2.50	740.14	781.67	1.00	0.70	740.14	781.54	1.00	0.54
	2.00	2.50	740.14	781.68	1.00	0.71	740.14	781.54	1.00	0.54
	2.50	2.50	740.14	781.70	1.00	0.71	740.14	781.54	1.00	0.54
	3.00	2.50	740.14	781.64	1.00	0.72	740.14	781.54	1.00	0.54
	0.50	3.50	740.14	781.65	1.00	0.72	740.14	781.54	1.00	0.54
	1.00	3.50	740.14	781.65	1.00	0.72	740.14	781.54	1.00	0.54
U_3O_7	1.50	3.50	740.14	781.67	1.00	0.72	740.14	781.54	1.00	0.54
	2.00	3.50	740.14	781.66	1.00	0.73	740.14	781.54	1.00	0.54
	2.50	3.50	740.14	781.59	1.00	0.73	740.14	781.54	1.00	0.54
	3.00	3.50	740.14	781.59	1.00	0.74	740.14	781.54	1.00	0.54
	0.50	4.50	740.14	781.64	1.00	0.74	740.14	781.54	1.00	0.54
	1.00	4.50	740.14	781.66	1.00	0.74	740.14	781.54	1.00	0.54
	1.50	4.50	740.14	781.68	1.00	0.75	740.14	781.54	1.00	0.54
	2.00	4.50	740.14	781.60	1.00	0.75	740.14	781.54	1.00	0.54
	2.50	4.50	740.14	781.60	1.00	0.75	740.14	781.54	1.00	0.54
	3.00	4.50	740.14	781.59	1.00	0.76	740.14	781.54	1.00	0.54
	0.50	5.50	740.14	781.67	1.00	0.76	740.14	781.54	1.00	0.54
	1.00	5.50	740.14	781.67	1.00	0.76	740.14	781.54	1.00	0.54
	1.50	5.50	740.14	781.59	1.00	0.77	740.14	781.54	1.00	0.54
	2.00	5.50	740.14	781.60	1.00	0.77	740.14	781.54	1.00	0.54
	2.50	5.50	740.14	781.63	1.00	0.78	740.14	781.54	1.00	0.54
	3.00	5.50	740.14	781.66	1.00	0.78	740.14	781.54	1.00	0.54
	0.50	0.00	738.88	780.39	1.00	0.82	738.88	781.20	1.00	0.63
	1.00	0.00	738.88	780.49	1.00	0.77	738.88	781.20	1.00	0.63
	1.50	0.00	738.88	780.49	1.00	0.73	738.88	781.20	1.00	0.63

	2.00	0.00	738.88	780.34	1.00	0.71	738.88	781.20	1.00	0.63
	2.50	0.00	738.88	780.41	1.00	0.70	738.88	781.20	1.00	0.63
	3.00	0.00	738.88	780.37	1.00	0.70	738.88	781.20	1.00	0.63
	0.50	2.50	738.88	780.38	1.00	0.73	738.88	781.20	1.00	0.63
	1.00	2.50	738.88	780.39	1.00	0.73	738.88	781.20	1.00	0.63
	1.50	2.50	738.88	780.36	1.00	0.73	738.88	781.20	1.00	0.63
	2.00	2.50	738.88	780.38	1.00	0.73	738.88	781.20	1.00	0.63
	2.50	2.50	738.88	780.44	1.00	0.73	738.88	781.20	1.00	0.63
	3.00	2.50	738.88	780.37	1.00	0.74	738.88	781.20	1.00	0.63
	0.50	3.50	738.88	780.42	1.00	0.74	738.88	781.20	1.00	0.63
	1.00	3.50	738.88	780.34	1.00	0.74	738.88	781.20	1.00	0.63
**KUO_3**	1.50	3.50	738.88	780.38	1.00	0.75	738.88	781.20	1.00	0.63
	2.00	3.50	738.88	780.40	1.00	0.75	738.88	781.20	1.00	0.63
	2.50	3.50	738.88	780.43	1.00	0.75	738.88	781.20	1.00	0.63
	3.00	3.50	738.88	780.35	1.00	0.76	738.88	781.20	1.00	0.63
	0.50	4.50	738.88	780.33	1.00	0.76	738.88	781.20	1.00	0.63
	1.00	4.50	738.88	780.35	1.00	0.76	738.88	781.20	1.00	0.63
	1.50	4.50	738.88	780.38	1.00	0.77	738.88	781.20	1.00	0.63
	2.00	4.50	738.88	780.40	1.00	0.77	738.88	781.20	1.00	0.63
	2.50	4.50	738.88	780.34	1.00	0.77	738.88	781.20	1.00	0.63
	3.00	4.50	738.88	780.39	1.00	0.78	738.88	781.20	1.00	0.63
	0.50	5.50	738.88	780.35	1.00	0.78	738.88	781.20	1.00	0.63
	1.00	5.50	738.88	780.40	1.00	0.78	738.88	781.20	1.00	0.63
	1.50	5.50	738.88	780.39	1.00	0.79	738.88	781.20	1.00	0.63
	2.00	5.50	738.88	780.41	1.00	0.79	738.88	781.20	1.00	0.63
	2.50	5.50	738.88	780.36	1.00	0.79	738.88	781.20	1.00	0.63
	3.00	5.50	738.88	780.37	1.00	0.80	738.88	781.20	1.00	0.63
	0.50	0.00	739.78	781.29	1.00	0.80	739.78	781.61	1.00	0.58
	1.00	0.00	739.78	781.19	1.00	0.70	739.78	781.61	1.00	0.58
	1.50	0.00	739.78	781.25	1.00	0.67	739.78	781.61	1.00	0.58
	2.00	0.00	739.78	781.29	1.00	0.66	739.78	781.61	1.00	0.58
	2.50	0.00	739.78	781.22	1.00	0.65	739.78	781.61	1.00	0.58
	3.00	0.00	739.78	781.24	1.00	0.66	739.78	781.61	1.00	0.58
	0.50	2.50	739.78	781.29	1.00	0.69	739.78	781.61	1.00	0.58
	1.00	2.50	739.78	781.20	1.00	0.69	739.78	781.61	1.00	0.58
	1.50	2.50	739.78	781.22	1.00	0.69	739.78	781.61	1.00	0.58
	2.00	2.50	739.78	781.25	1.00	0.70	739.78	781.61	1.00	0.58
	2.50	2.50	739.78	781.27	1.00	0.70	739.78	781.61	1.00	0.58
	3.00	2.50	739.78	781.28	1.00	0.71	739.78	781.61	1.00	0.58
	0.50	3.50	739.78	781.22	1.00	0.71	739.78	781.61	1.00	0.58
	1.00	3.50	739.78	781.24	1.00	0.71	739.78	781.61	1.00	0.58
**U_3O_8**	1.50	3.50	739.78	781.25	1.00	0.72	739.78	781.61	1.00	0.58
	2.00	3.50	739.78	781.26	1.00	0.72	739.78	781.61	1.00	0.58
	2.50	3.50	739.78	781.27	1.00	0.73	739.78	781.61	1.00	0.58
	3.00	3.50	739.78	781.29	1.00	0.73	739.78	781.61	1.00	0.58
	0.50	4.50	739.78	781.26	1.00	0.73	739.78	781.61	1.00	0.58
	1.00	4.50	739.78	781.27	1.00	0.74	739.78	781.61	1.00	0.58
	1.50	4.50	739.78	781.27	1.00	0.74	739.78	781.61	1.00	0.58
	2.00	4.50	739.78	781.27	1.00	0.74	739.78	781.61	1.00	0.58
	2.50	4.50	739.78	781.28	1.00	0.75	739.78	781.61	1.00	0.58
	3.00	4.50	739.78	781.30	1.00	0.76	739.78	781.61	1.00	0.58
	0.50	5.50	739.78	781.27	1.00	0.76	739.78	781.61	1.00	0.58
	1.00	5.50	739.78	781.28	1.00	0.76	739.78	781.61	1.00	0.58
	1.50	5.50	739.78	781.29	1.00	0.77	739.78	781.61	1.00	0.58

	2.00	5.50	739.78	781.27	1.00	0.77	739.78	781.61	1.00	0.58
	2.50	5.50	739.78	781.27	1.00	0.77	739.78	781.61	1.00	0.58
	3.00	5.50	739.78	781.18	1.00	0.78	739.78	781.61	1.00	0.58

	0.50	0.00	739.42	780.95	1.00	0.78	739.42	N/A	1.00	N/A
	1.00	0.00	739.42	780.96	1.00	0.78	739.42	N/A	1.00	N/A
	1.50	0.00	739.42	780.97	1.00	0.79	739.42	N/A	1.00	N/A
	2.00	0.00	739.42	781.00	1.00	0.79	739.42	N/A	1.00	N/A
	2.50	0.00	739.42	780.94	1.00	0.79	739.42	N/A	1.00	N/A
	3.00	0.00	739.42	780.95	1.00	0.80	739.42	N/A	1.00	N/A
	0.50	2.50	739.42	780.95	1.00	0.72	739.42	N/A	1.00	N/A
	1.00	2.50	739.42	781.00	1.00	0.72	739.42	N/A	1.00	N/A
	1.50	2.50	739.42	780.91	1.00	0.73	739.42	N/A	1.00	N/A
	2.00	2.50	739.42	780.95	1.00	0.73	739.42	N/A	1.00	N/A
	2.50	2.50	739.42	781.00	1.00	0.73	739.42	N/A	1.00	N/A
	3.00	2.50	739.42	780.93	1.00	0.73	739.42	N/A	1.00	N/A
	0.50	3.50	739.42	780.89	1.00	0.74	739.42	N/A	1.00	N/A
	1.00	3.50	739.42	780.91	1.00	0.74	739.42	N/A	1.00	N/A
**$BaUO_4$**	1.50	3.50	739.42	780.94	1.00	0.74	739.42	N/A	1.00	N/A
	2.00	3.50	739.42	780.97	1.00	0.75	739.42	N/A	1.00	N/A
	2.50	3.50	739.42	780.91	1.00	0.75	739.42	N/A	1.00	N/A
	3.00	3.50	739.42	780.94	1.00	0.76	739.42	N/A	1.00	N/A
	0.50	4.50	739.42	780.91	1.00	0.76	739.42	N/A	1.00	N/A
	1.00	4.50	739.42	780.96	1.00	0.76	739.42	N/A	1.00	N/A
	1.50	4.50	739.42	780.97	1.00	0.77	739.42	N/A	1.00	N/A
	2.00	4.50	739.42	781.01	1.00	0.77	739.42	N/A	1.00	N/A
	2.50	4.50	739.42	780.92	1.00	0.77	739.42	N/A	1.00	N/A
	3.00	4.50	739.42	780.99	1.00	0.78	739.42	N/A	1.00	N/A
	0.50	5.50	739.42	780.97	1.00	0.78	739.42	N/A	1.00	N/A
	1.00	5.50	739.42	780.98	1.00	0.78	739.42	N/A	1.00	N/A
	1.50	5.50	739.42	781.00	1.00	0.78	739.42	N/A	1.00	N/A
	2.00	5.50	739.42	781.00	1.00	0.79	739.42	N/A	1.00	N/A
	2.50	5.50	739.42	780.95	1.00	0.79	739.42	N/A	1.00	N/A
	3.00	5.50	739.42	780.98	1.00	0.80	739.42	N/A	1.00	N/A

Table S6: Comparison of calculations with experimental EELS results for the **O *K*-edge**. Energy separation between the first and second peaks $\Delta Peak$ (Peak II – Peak I, in eV) and relative intensity ratios $R_{Intensity}$ (Intensity I / Intensity II, in arbitrary units, a. u.) for uranium oxides. Percentage error of calculated vs experimental values for the peak separation $\Delta Peak$ and the $R_{Intensity}$ across the uranium oxide series. Errors were computed using Eq. (1) for $\Delta Peak$ error (%) and Eq. (2) for $R_{Intensity}$ error (%). Positive values indicate overestimation, while negative values indicate underestimation relative to experiment. The last column reports the total absolute error, defined as the sum of the absolute percentage errors in $\Delta Peak$ and $R_{Intensity}$, according to Eq. (3). Values highlighted in bold correspond to the selected Gaussian broadening and core-hole lifetime parameters that best reproduce the experimental spectra and preserve physical relevance.

Oxygen *K*-edge

Uranium Oxides	Parameters		Calculation		Experiment		Percentage Error (%)		Absolute error
	Gaussian broadening	Core-hole lifetime	$\Delta Peak$	$R_{Intensity}$	$\Delta Peak$	$R_{Intensity}$	$\Delta Peak$ (%)	$R_{Intensity}$ (%)	$\lvert\Delta Peak\ (\%)\rvert + \lvert R_{Intensity}(\%)\rvert$
UO_2	0.50	0.00	5.20	0.98	5.22	0.88	-0.38	11.36	11.75
	1.00	0.00	5.29	0.96	5.22	0.88	1.34	9.09	10.43
	1.50	0.00	5.60	0.93	5.22	0.88	7.28	5.68	12.96
	2.00	0.00	5.74	0.90	5.22	0.88	9.96	2.27	12.23
	2.50	0.00	5.71	0.88	5.22	0.88	9.39	0.00	9.39
	3.00	0.00	5.70	0.85	5.22	0.88	9.20	-3.41	12.60
	0.50	0.20	5.21	0.99	5.22	0.88	-0.19	12.50	12.69
	1.00	0.20	5.31	0.94	5.22	0.88	1.72	6.82	8.54
	1.50	0.20	5.60	0.92	5.22	0.88	7.28	4.55	11.83
	**2.00**	**0.20**	**5.60**	**0.90**	**5.22**	**0.88**	**7.28**	**2.27**	**9.55**
	2.50	0.20	5.69	0.87	5.22	0.88	9.00	-1.14	10.14
	3.00	0.20	5.70	0.84	5.22	0.88	9.20	-4.55	13.74
	0.50	0.40	5.30	0.96	5.22	0.88	1.53	9.09	10.62
	1.00	0.40	5.40	0.92	5.22	0.88	3.45	4.55	7.99
	1.50	0.40	5.60	0.90	5.22	0.88	7.28	2.27	9.55
	2.00	0.40	5.60	0.88	5.22	0.88	7.28	0.00	7.28
	2.50	0.40	5.70	0.86	5.22	0.88	9.20	-2.27	11.47
	3.00	0.40	5.69	0.83	5.22	0.88	9.00	-5.68	14.69
	0.50	0.60	5.30	0.93	5.22	0.88	1.53	5.68	7.21
	1.00	0.60	5.40	0.91	5.22	0.88	3.45	3.41	6.86
	1.50	0.60	5.60	0.90	5.22	0.88	7.28	2.27	9.55
	2.00	0.60	5.60	0.87	5.22	0.88	7.28	-1.14	8.42
	2.50	0.60	5.64	0.84	5.22	0.88	8.05	-4.55	12.59
	3.00	0.60	5.60	0.82	5.22	0.88	7.28	-6.82	14.10
	0.50	0.80	5.30	0.91	5.22	0.88	1.53	3.41	4.94
	1.00	0.80	5.40	0.89	5.22	0.88	3.45	1.14	4.58
	1.50	0.80	5.60	0.88	5.22	0.88	7.28	0.00	7.28
	2.00	0.80	5.60	0.86	5.22	0.88	7.28	-2.27	9.55
	2.50	0.80	5.60	0.84	5.22	0.88	7.28	-4.55	11.83
	3.00	0.80	5.64	0.82	5.22	0.88	8.05	-6.82	14.86
	0.50	0.00	5.60	0.87	5.03	0.83	11.33	4.82	16.15
	1.00	0.00	5.70	0.86	5.03	0.83	13.32	3.61	16.93
	1.50	0.00	5.50	0.86	5.03	0.83	9.34	3.61	12.96
	2.00	0.00	5.40	0.85	5.03	0.83	7.36	2.41	9.77
	2.50	0.00	5.30	0.85	5.03	0.83	5.37	2.41	7.78
	3.00	0.00	5.00	0.84	5.03	0.83	-0.60	1.20	1.80
	0.50	0.20	5.50	0.86	5.03	0.83	9.34	3.61	12.96

	1.00	0.20	5.50	0.86	5.03	0.83	9.34	3.61	12.96
	1.50	0.20	5.50	0.85	5.03	0.83	9.34	2.41	11.75
	**2.00**	**0.20**	**5.30**	**0.85**	**5.03**	**0.83**	**5.37**	**2.41**	**7.78**
	2.50	0.20	5.10	0.84	5.03	0.83	1.39	1.20	2.60
	3.00	0.20	4.90	0.84	5.03	0.83	-2.58	1.20	3.79
	0.50	0.40	5.50	0.85	5.03	0.83	9.34	2.41	11.75
	1.00	0.40	5.40	0.85	5.03	0.83	7.36	2.41	9.77
**U_4O_9**	1.50	0.40	5.30	0.85	5.03	0.83	5.37	2.41	7.78
	2.00	0.40	5.20	0.84	5.03	0.83	3.38	1.20	4.58
	2.50	0.40	4.99	0.84	5.03	0.83	-0.80	1.20	2.00
	3.00	0.40	4.80	0.84	5.03	0.83	-4.57	1.20	5.78
	0.50	0.60	5.40	0.84	5.03	0.83	7.36	1.20	8.56
	1.00	0.60	5.30	0.84	5.03	0.83	5.37	1.20	6.57
	1.50	0.60	5.20	0.84	5.03	0.83	3.38	1.45	4.83
	2.00	0.60	5.10	0.84	5.03	0.83	1.39	1.20	2.60
	2.50	0.60	4.90	0.84	5.03	0.83	-2.58	0.96	3.55
	3.00	0.60	4.50	0.84	5.03	0.83	-10.54	1.45	11.98
	0.50	0.80	5.30	0.84	5.03	0.83	5.37	1.08	6.45
	1.00	0.80	5.20	0.84	5.03	0.83	3.38	0.96	4.34
	1.50	0.80	5.10	0.84	5.03	0.83	1.39	0.96	2.36
	2.00	0.80	5.00	0.84	5.03	0.83	-0.60	0.84	1.44
	2.50	0.80	4.80	0.84	5.03	0.83	-4.57	0.84	5.42
	3.00	0.80	4.55	0.84	5.03	0.83	-9.54	1.20	10.75

	0.50	0.00	5.50	0.83	4.14	0.85	32.85	-2.12	34.97
	1.00	0.00	5.60	0.80	4.14	0.85	35.27	-6.00	41.27
	1.50	0.00	5.40	0.78	4.14	0.85	30.43	-7.88	38.32
	2.00	0.00	5.00	0.78	4.14	0.85	20.77	-8.24	29.01
	2.50	0.00	4.90	0.79	4.14	0.85	18.36	-7.06	25.42
	3.00	0.00	4.86	0.79	4.14	0.85	17.39	-7.06	24.45
	0.50	0.20	5.50	0.81	4.14	0.85	32.85	-4.59	37.44
	1.00	0.20	5.50	0.79	4.14	0.85	32.85	-7.29	40.14
	1.50	0.20	5.20	0.78	4.14	0.85	25.60	-8.35	33.96
	**2.00**	**0.20**	**5.32**	**0.77**	**4.14**	**0.85**	**28.50**	**-9.41**	**37.91**
	2.50	0.20	4.95	0.78	4.14	0.85	19.57	-8.24	27.80
	3.00	0.20	4.63	0.80	4.14	0.85	11.84	-5.88	17.72
	0.50	0.40	5.50	0.80	4.14	0.85	32.85	-6.35	39.20
	1.00	0.40	5.40	0.78	4.14	0.85	30.43	-8.24	38.67
**U_3O_7**	1.50	0.40	5.49	0.77	4.14	0.85	32.61	-9.41	42.02
	2.00	0.40	4.95	0.78	4.14	0.85	19.57	-8.24	27.80
	2.50	0.40	4.90	0.78	4.14	0.85	18.36	-8.24	26.59
	3.00	0.40	4.83	0.79	4.14	0.85	16.67	-7.06	23.73
	0.50	0.60	5.50	0.79	4.14	0.85	32.85	-7.65	40.50
	1.00	0.60	5.30	0.78	4.14	0.85	28.02	-8.82	36.84
	1.50	0.60	5.10	0.78	4.14	0.85	23.19	-8.24	31.42
	2.00	0.60	5.13	0.77	4.14	0.85	23.91	-9.41	33.32
	2.50	0.60	4.85	0.79	4.14	0.85	17.15	-7.06	24.21
	3.00	0.60	4.90	0.78	4.14	0.85	18.36	-8.24	26.59
	0.50	0.80	5.40	0.78	4.14	0.85	30.43	-8.59	39.02
	1.00	0.80	5.29	0.77	4.14	0.85	27.78	-9.41	37.19
	1.50	0.80	5.10	0.78	4.14	0.85	23.19	-8.24	31.42
	2.00	0.80	4.90	0.78	4.14	0.85	18.36	-8.24	26.59
	2.50	0.80	4.67	0.79	4.14	0.85	12.80	-7.06	19.86
	3.00	0.80	4.76	0.79	4.14	0.85	14.98	-7.06	22.03

	0.50	0.00	5.93	1.52	5.93	1.30	0.00	16.67	16.67
	1.00	0.00	5.93	1.35	5.93	1.30	-0.51	4.05	4.56
	1.50	0.00	5.93	1.25	5.93	1.30	-0.51	-3.75	4.26
	2.00	0.00	5.93	1.16	5.93	1.30	-0.34	-10.47	10.80
	2.50	0.00	5.93	1.10	5.93	1.30	-1.52	-15.38	16.90
	3.00	0.00	5.93	1.05	5.93	1.30	-0.84	-18.95	19.79
	0.50	0.20	5.93	1.41	5.93	1.30	0.17	8.45	8.62
	1.00	0.20	5.93	1.30	5.93	1.30	-0.51	0.00	0.51
	**1.50**	**0.20**	**5.93**	**1.20**	**5.93**	**1.30**	**-0.51**	**-7.23**	**7.73**
	2.00	0.20	5.93	1.14	5.93	1.30	-1.01	-12.50	13.51
	2.50	0.20	5.93	1.09	5.93	1.30	-1.85	-16.30	18.16
	3.00	0.20	5.93	1.04	5.93	1.30	-1.18	-19.79	20.97
	0.50	0.40	5.93	1.33	5.93	1.30	0.34	2.67	3.00
	1.00	0.40	5.93	1.25	5.93	1.30	-0.34	-3.75	4.09
**KUO_3**	1.50	0.40	5.93	1.18	5.93	1.30	-0.51	-9.41	9.92
	2.00	0.40	5.93	1.11	5.93	1.30	-0.84	-14.44	15.29
	2.50	0.40	5.93	1.06	5.93	1.30	-2.02	-18.09	20.11
	3.00	0.40	5.93	1.03	5.93	1.30	-2.19	-20.62	22.81
	0.50	0.60	5.93	1.27	5.93	1.30	1.18	-2.53	3.71
	1.00	0.60	5.93	1.20	5.93	1.30	-0.51	-7.23	7.73
	1.50	0.60	5.93	1.15	5.93	1.30	-0.51	-11.49	12.00
	2.00	0.60	5.93	1.10	5.93	1.30	-2.02	-15.38	17.41
	2.50	0.60	5.93	1.05	5.93	1.30	-2.02	-18.95	20.97
	3.00	0.60	5.93	1.03	5.93	1.30	-2.02	-20.62	22.64
	0.50	0.80	5.93	1.22	5.93	1.30	-1.01	-6.10	7.11
	1.00	0.80	5.93	1.18	5.93	1.30	-0.34	-9.41	9.75
	1.50	0.80	5.93	1.11	5.93	1.30	-1.01	-14.44	15.46
	2.00	0.80	5.93	1.08	5.93	1.30	-2.02	-17.20	19.23
	2.50	0.80	5.93	1.04	5.93	1.30	-2.87	-19.79	22.66
	3.00	0.80	5.93	1.02	5.93	1.30	-3.88	20.36	24.24
	0.50	0.00	8.41	1.72	6.48	1.30	29.78	32.76	62.54
	1.00	0.00	8.45	1.56	6.48	1.30	30.40	20.31	50.71
	1.50	0.00	8.60	1.47	6.48	1.30	32.72	13.24	45.95
	2.00	0.00	8.69	1.22	6.48	1.30	34.10	-6.10	40.20
	2.50	0.00	8.80	1.33	6.48	1.30	35.80	2.67	38.47
	3.00	0.00	0.70	1.28	6.48	1.30	-89.20	-1.28	90.48
	0.50	0.20	8.40	1.61	6.48	1.30	29.63	24.19	53.82
	1.00	0.20	8.44	1.49	6.48	1.30	30.25	14.93	45.17
	1.50	0.20	8.60	1.43	6.48	1.30	32.72	10.00	42.72
	2.00	0.20	8.69	1.35	6.48	1.30	34.10	4.05	38.16
	**2.50**	**0.20**	**8.66**	**1.30**	**6.48**	**1.30**	**33.64**	**0.00**	**33.64**
	3.00	0.20	8.61	1.25	6.48	1.30	32.87	-3.75	36.62
	0.50	0.40	8.40	1.54	6.48	1.30	29.63	18.46	48.09
	1.00	0.40	8.55	1.45	6.48	1.30	31.94	11.59	43.54
**U_3O_8**	1.50	0.40	8.60	1.39	6.48	1.30	32.72	6.94	39.66
	2.00	0.40	8.68	1.32	6.48	1.30	33.95	1.32	35.27
	2.50	0.40	8.62	1.27	6.48	1.30	33.02	-2.53	35.56
	3.00	0.40	8.58	1.23	6.48	1.30	32.41	-4.94	37.35
	0.50	0.60	8.40	1.47	6.48	1.30	29.63	13.24	42.86
	1.00	0.60	8.50	1.41	6.48	1.30	31.17	8.45	39.62
	1.50	0.60	8.60	1.35	6.48	1.30	32.72	4.05	36.77
	2.00	0.60	8.65	1.30	6.48	1.30	33.49	0.00	33.49
	2.50	0.60	8.60	1.25	6.48	1.30	32.72	-3.75	36.47
	3.00	0.60	8.57	1.22	6.48	1.30	32.25	-6.10	38.35

	0.50	0.80	8.39	1.41	6.48	1.30	29.48	8.45	37.93
	1.00	0.80	8.55	1.37	6.48	1.30	31.94	5.48	37.42
	1.50	0.80	8.59	1.32	6.48	1.30	32.56	1.32	33.88
	2.00	0.80	8.65	1.27	6.48	1.30	33.49	-2.53	36.02
	2.50	0.80	8.63	1.23	6.48	1.30	33.18	-4.94	38.12
	3.00	0.80	8.44	1.19	6.48	1.30	30.25	-8.33	38.58
	0.50	0.00	6.50	1.43	4.86	1.25	33.74	14.29	48.03
	1.00	0.00	6.50	1.43	4.86	1.25	33.74	14.29	48.03
	1.50	0.00	6.46	1.43	4.86	1.25	32.92	14.29	47.21
	2.00	0.00	6.46	1.43	4.86	1.25	32.92	14.29	47.21
	2.50	0.00	6.40	1.41	4.86	1.25	31.69	12.68	44.36
	3.00	0.00	6.54	1.41	4.86	1.25	34.57	12.68	47.24
	0.50	0.20	6.51	1.41	4.86	1.25	33.95	12.68	46.63
	1.00	0.20	6.48	1.41	4.86	1.25	33.33	12.68	46.01
	1.50	0.20	6.45	1.41	4.86	1.25	32.72	12.68	45.39
	**2.00**	**0.20**	**6.43**	**1.41**	**4.86**	**1.25**	**32.30**	**12.68**	**44.98**
	2.50	0.20	6.39	1.41	4.86	1.25	31.48	12.68	44.16
	3.00	0.20	6.46	1.39	4.86	1.25	32.92	11.11	44.03
	0.50	0.40	6.48	1.41	4.86	1.25	33.33	12.68	46.01
	1.00	0.40	6.45	1.41	4.86	1.25	32.72	12.68	45.39
**$BaUO_4$**	1.50	0.40	6.44	1.41	4.86	1.25	32.51	12.68	45.19
	2.00	0.40	6.36	1.39	4.86	1.25	30.86	11.11	41.98
	2.50	0.40	6.37	1.39	4.86	1.25	31.07	11.11	42.18
	3.00	0.40	6.46	1.37	4.86	1.25	32.92	9.59	42.51
	0.50	0.60	6.45	1.39	4.86	1.25	32.72	11.11	43.83
	1.00	0.60	6.44	1.39	4.86	1.25	32.51	11.11	43.62
	1.50	0.60	6.39	1.39	4.86	1.25	31.48	11.11	42.59
	2.00	0.60	6.37	1.37	4.86	1.25	31.07	9.59	40.66
	2.50	0.60	6.31	1.37	4.86	1.25	29.84	9.59	39.42
	3.00	0.60	6.26	1.35	4.86	1.25	28.81	8.11	36.91
	0.50	0.80	6.44	1.37	4.86	1.25	32.51	9.59	42.10
	1.00	0.80	6.37	1.37	4.86	1.25	31.07	9.59	40.66
	1.50	0.80	6.29	1.37	4.86	1.25	29.42	9.59	39.01
	2.00	0.80	6.23	1.35	4.86	1.25	28.19	8.11	36.30
	2.50	0.80	6.24	1.35	4.86	1.25	28.40	8.11	36.50
	3.00	0.80	6.24	1.33	4.86	1.25	28.40	6.67	35.06

Table S7: Comparison of calculations with experimental EELS results for the **U $N_{6,7}$-edge**. Energy separation between the first and second peaks $\Delta Peak$ (Peak II – Peak I, in eV) and relative intensity ratios $R_{Intensity}$ (Intensity I / Intensity II, in arbitrary units, a. u.) for uranium oxides. Percentage error of calculated vs experimental values for the peak separation $\Delta Peak$ and the $R_{Intensity}$ across the uranium oxide series. Errors were computed using Eq. (1) for $\Delta Peak$ error (%) and Eq. (2) for $R_{Intensity}$ error (%). Positive values indicate overestimation, while negative values indicate underestimation relative to experiment. The last column reports the total absolute error, defined as the sum of the absolute percentage errors in $\Delta Peak$ and $R_{Intensity}$, according to Eq. (3). Values highlighted in bold correspond to the selected Gaussian broadening and core-hole lifetime parameters that best reproduce the experimental spectra and preserve physical relevance.

Uranium $N_{6,7}$-edge

Uranium Oxides	Parameters		Calculation		Experiment		Percentage Error (%)		Absolute error
	Gaussian broadening	Core-hole lifetime	$\Delta Peak$	$R_{Intensity}$	$\Delta Peak$	$R_{Intensity}$	$\Delta Peak$ (%)	$R_{Intensity}$ (%)	$\lvert\Delta Peak\ (\%)\rvert + \lvert R_{Intensity}(\%)\rvert$
UO_2	0.50	0.00	7.55	0.79	10.98	0.53	-31.24	49.06	80.30
	1.00	0.00	7.56	0.80	10.98	0.53	-31.15	50.94	82.09
	1.50	0.00	7.64	0.81	10.98	0.53	-30.42	52.83	83.25
	2.00	0.00	7.91	0.81	10.98	0.53	-27.96	52.83	80.79
	2.50	0.00	8.29	0.81	10.98	0.53	-24.50	52.83	77.33
	3.00	0.00	8.77	0.80	10.98	0.53	-20.13	50.94	71.07
	0.50	0.30	7.60	0.80	10.98	0.53	-30.78	50.94	81.73
	**1.00**	**0.30**	**7.64**	**0.81**	**10.98**	**0.53**	**-30.42**	**52.83**	**83.25**
	1.50	0.30	7.80	0.81	10.98	0.53	-28.96	52.83	81.79
	2.00	0.30	7.95	0.82	10.98	0.53	-27.60	54.72	82.31
	2.50	0.30	8.47	0.81	10.98	0.53	-22.86	52.83	75.69
	3.00	0.30	8.90	0.80	10.98	0.53	-18.94	50.94	69.89
	0.50	0.40	7.60	0.80	10.98	0.53	-30.78	50.94	81.73
	1.00	0.40	7.69	0.81	10.98	0.53	-29.96	52.83	82.79
	1.50	0.40	7.84	0.82	10.98	0.53	-28.60	54.72	83.31
	2.00	0.40	8.10	0.81	10.98	0.53	-26.23	52.83	79.06
	2.50	0.40	8.50	0.81	10.98	0.53	-22.59	52.83	75.42
	3.00	0.40	8.94	0.80	10.98	0.53	-18.58	50.94	69.52
	0.50	0.50	7.66	0.81	10.98	0.53	-30.24	52.83	83.07
	1.00	0.50	7.70	0.81	10.98	0.53	-29.87	52.83	82.70
	1.50	0.50	7.87	0.82	10.98	0.53	-28.32	54.72	83.04
	2.00	0.50	8.14	0.81	10.98	0.53	-25.87	52.83	78.70
	2.50	0.50	8.60	0.81	10.98	0.53	-21.68	52.83	74.51
	3.00	0.50	9.04	0.80	10.98	0.53	-17.67	50.94	68.61
	0.50	0.60	7.70	0.81	10.98	0.53	-29.87	52.83	82.70
	1.00	0.60	7.74	0.81	10.98	0.53	-29.51	52.83	82.34
	1.50	0.60	7.90	0.82	10.98	0.53	-28.05	54.72	82.77
	2.00	0.60	8.10	0.81	10.98	0.53	-26.23	52.83	79.06
	2.50	0.60	8.60	0.80	10.98	0.53	-21.68	50.94	72.62
	3.00	0.60	9.06	0.80	10.98	0.53	-17.49	50.94	68.43
	0.50	0.00	8.02	0.83	11.52	0.88	-30.38	-5.68	36.06
	1.00	0.00	11.20	0.83	11.52	0.88	-2.78	-5.68	8.46
	1.50	0.00	11.12	0.82	11.52	0.88	-3.47	-6.82	10.29
	2.00	0.00	9.64	0.81	11.52	0.88	-16.32	-7.95	24.27
	2.50	0.00	9.55	0.80	11.52	0.88	-17.10	-9.09	26.19
	3.00	0.00	9.56	0.80	11.52	0.88	-17.01	-9.09	26.10
	0.50	0.30	10.36	0.83	11.52	0.88	-10.07	-5.68	15.75
	**1.00**	**0.30**	**10.26**	0.82	11.52	0.88	**-10.94**	**-6.82**	**17.76**

	1.50	0.30	9.96	0.82	11.52	0.88	-13.54	-6.82	20.36
	2.00	0.30	9.58	0.81	11.52	0.88	-16.84	-7.95	24.79
	2.50	0.30	9.60	0.80	11.52	0.88	-16.67	-9.09	25.76
	3.00	0.30	9.59	0.79	11.52	0.88	-16.75	-10.23	26.98
	0.50	0.40	10.36	0.83	11.52	0.88	-10.07	-5.68	15.75
	1.00	0.40	10.17	0.83	11.52	0.88	-11.72	-5.68	17.40
U_4O_9	1.50	0.40	9.87	0.82	11.52	0.88	-14.32	-6.82	21.14
	2.00	0.40	9.59	0.81	11.52	0.88	-16.75	-7.95	24.71
	2.50	0.40	9.60	0.80	11.52	0.88	-16.67	-9.09	25.76
	3.00	0.40	9.59	0.79	11.52	0.88	-16.75	-10.23	26.98
	0.50	0.50	10.29	0.82	11.52	0.88	-10.68	-6.82	17.50
	1.00	0.50	10.15	0.82	11.52	0.88	-11.89	-6.82	18.71
	1.50	0.50	9.90	0.81	11.52	0.88	-14.06	-7.95	22.02
	2.00	0.50	9.59	0.80	11.52	0.88	-16.75	-9.09	25.84
	2.50	0.50	9.60	0.79	11.52	0.88	-16.67	-10.23	26.89
	3.00	0.50	9.59	0.79	11.52	0.88	-16.75	-10.23	26.98
	0.50	0.60	10.29	0.82	11.52	0.88	-10.68	-6.82	17.50
	1.00	0.60	10.06	0.82	11.52	0.88	-12.67	-6.82	19.49
	1.50	0.60	9.80	0.82	11.52	0.88	-14.93	-6.82	21.75
	2.00	0.60	9.62	0.80	11.52	0.88	-16.49	-9.09	25.58
	2.50	0.60	9.60	0.79	11.52	0.88	-16.67	-10.23	26.89
	3.00	0.60	9.61	0.79	11.52	0.88	-16.58	-10.23	26.81
	0.50	0.00	10.60	0.83	11.70	0.57	-9.4	45.6	55.0
	1.00	0.00	10.40	0.82	11.70	0.57	-11.1	43.9	55.0
	1.50	0.00	10.04	0.81	11.70	0.57	-14.2	42.1	56.3
	2.00	0.00	9.94	0.80	11.70	0.57	-15.0	40.4	55.4
	2.50	0.00	9.85	0.79	11.70	0.57	-15.8	38.6	54.4
	3.00	0.00	9.87	0.79	11.70	0.57	-15.6	38.6	54.2
	0.50	0.30	10.49	0.82	11.70	0.57	-10.3	43.9	54.2
	**1.00**	**0.30**	**10.16**	**0.82**	**11.70**	**0.57**	**-13.2**	**43.9**	**57.0**
	1.50	0.30	9.94	0.81	11.70	0.57	-15.0	42.1	57.1
	2.00	0.30	9.96	0.80	11.70	0.57	-14.9	40.4	55.2
	2.50	0.30	9.84	0.79	11.70	0.57	-15.9	38.6	54.5
	3.00	0.30	9.86	0.78	11.70	0.57	-15.7	36.8	52.6
	0.50	0.40	10.50	0.82	11.70	0.57	-10.3	43.9	54.1
	1.00	0.40	10.15	0.81	11.70	0.57	-13.2	42.1	55.4
U_3O_7	1.50	0.40	9.94	0.80	11.70	0.57	-15.0	40.4	55.4
	2.00	0.40	9.83	0.80	11.70	0.57	-16.0	40.4	56.3
	2.50	0.40	9.85	0.79	11.70	0.57	-15.8	38.6	54.4
	3.00	0.40	9.81	0.78	11.70	0.57	-16.2	36.8	53.0
	0.50	0.50	10.40	0.82	11.70	0.57	-11.1	43.9	55.0
	1.00	0.50	10.05	0.81	11.70	0.57	-14.1	42.1	56.2
	1.50	0.50	10.90	0.80	11.70	0.57	-6.8	40.4	47.2
	2.00	0.50	9.75	0.79	11.70	0.57	-16.7	38.6	55.3
	2.50	0.50	9.81	0.79	11.70	0.57	-16.2	38.6	54.8
	3.00	0.50	9.82	0.78	11.70	0.57	-16.1	36.8	52.9
	0.50	0.60	10.28	0.81	11.70	0.57	-12.1	42.1	54.2
	1.00	0.60	10.07	0.81	11.70	0.57	-13.9	42.1	56.0
	1.50	0.60	9.92	0.80	11.70	0.57	-15.2	40.4	55.6
	2.00	0.60	9.81	0.79	11.70	0.57	-16.2	38.6	54.8
	2.50	0.60	9.85	0.79	11.70	0.57	-15.8	38.6	54.4
	3.00	0.60	9.81	0.78	11.70	0.57	-16.2	36.8	53.0
	0.50	0.00	10.74	0.71	11.69	0.86	-8.13	-17.44	25.57

	1.00	0.00	10.60	0.68	11.69	0.86	-9.32	-20.93	30.25
	1.50	0.00	10.47	0.65	11.69	0.86	-10.44	-24.42	34.85
	2.00	0.00	10.41	0.62	11.69	0.86	-10.95	-27.91	38.86
	2.50	0.00	10.34	0.59	11.69	0.86	-11.55	-31.40	42.94
	3.00	0.00	10.14	0.57	11.69	0.86	-13.26	-33.72	46.98
	**0.50**	**0.30**	**10.63**	**0.68**	**11.69**	**0.86**	**-9.07**	**-20.93**	**30.00**
	1.00	0.30	10.50	0.65	11.69	0.86	-10.18	-24.42	34.60
	1.50	0.30	10.46	0.63	11.69	0.86	-10.52	-26.74	37.27
	2.00	0.30	10.39	0.60	11.69	0.86	-11.12	-30.23	41.35
	2.50	0.30	10.21	0.58	11.69	0.86	-12.66	-32.56	45.22
	3.00	0.30	10.12	0.56	11.69	0.86	-13.43	-34.88	48.31
	0.50	0.40	10.63	0.67	11.69	0.86	-9.07	-22.09	31.16
	1.00	0.40	10.51	0.64	11.69	0.86	-10.09	-25.58	35.68
**KUO_3**	1.50	0.40	10.46	0.62	11.69	0.86	-10.52	-27.91	38.43
	2.00	0.40	10.40	0.60	11.69	0.86	-11.04	-30.23	41.27
	2.50	0.40	10.20	0.58	11.69	0.86	-12.75	-32.56	45.30
	3.00	0.40	10.11	0.56	11.69	0.86	-13.52	-34.88	48.40
	0.50	0.50	10.64	0.66	11.69	0.86	-8.98	-23.26	32.24
	1.00	0.50	10.49	0.64	11.69	0.86	-10.27	-25.58	35.85
	1.50	0.50	10.45	0.62	11.69	0.86	-10.61	-27.91	38.51
	2.00	0.50	10.28	0.59	11.69	0.86	-12.06	-31.40	43.46
	2.50	0.50	10.21	0.57	11.69	0.86	-12.66	-33.72	46.38
	3.00	0.50	10.12	0.56	11.69	0.86	-13.43	-34.88	48.31
	0.50	0.60	10.62	0.65	11.69	0.86	-9.15	-24.42	33.57
	1.00	0.60	10.49	0.63	11.69	0.86	-10.27	-26.74	37.01
	1.50	0.60	10.44	0.61	11.69	0.86	-10.69	-29.07	39.76
	2.00	0.60	10.28	0.59	11.69	0.86	-12.06	-31.40	43.46
	2.50	0.60	10.18	0.57	11.69	0.86	-12.92	-33.72	46.64
	3.00	0.60	10.11	0.55	11.69	0.86	-13.52	-36.05	49.56

	0.50	0.00	9.60	0.64	12.77	0.45	-24.82	42.22	67.05
	1.00	0.00	10.06	0.64	12.77	0.45	-21.22	42.22	63.44
	1.50	0.00	10.25	0.63	12.77	0.45	-19.73	40.00	59.73
	2.00	0.00	10.36	0.63	12.77	0.45	-18.87	40.00	58.87
	2.50	0.00	10.30	0.62	12.77	0.45	-19.34	37.78	57.12
	3.00	0.00	10.24	0.62	12.77	0.45	-19.81	37.78	57.59
	0.50	0.30	9.87	0.64	12.77	0.45	-22.71	42.22	64.93
	**1.00**	**0.30**	**10.11**	**0.63**	**12.77**	**0.45**	**-20.83**	**40.00**	**60.83**
	1.50	0.30	10.21	0.63	12.77	0.45	-20.05	40.00	60.05
	2.00	0.30	10.29	0.62	12.77	0.45	-19.42	37.78	57.20
	2.50	0.30	10.27	0.62	12.77	0.45	-19.58	37.78	57.35
	3.00	0.30	10.22	0.71	12.77	0.45	-19.97	57.78	77.75
	0.50	0.40	9.98	0.63	12.77	0.45	-21.85	40.00	61.85
	1.00	0.40	10.20	0.63	12.77	0.45	-20.13	40.00	60.13
**U_3O_8**	1.50	0.40	10.25	0.63	12.77	0.45	-19.73	40.00	59.73
	2.00	0.40	10.27	0.62	12.77	0.45	-19.58	37.78	57.35
	2.50	0.40	10.19	0.62	12.77	0.45	-20.20	37.78	57.98
	3.00	0.40	10.20	0.61	12.77	0.45	-20.13	35.56	55.68
	0.50	0.50	10.10	0.63	12.77	0.45	-20.91	40.00	60.91
	1.00	0.50	10.13	0.63	12.77	0.45	-20.67	40.00	60.67
	1.50	0.50	10.19	0.62	12.77	0.45	-20.20	37.78	57.98
	2.00	0.50	10.29	0.62	12.77	0.45	-19.42	37.78	57.20
	2.50	0.50	10.12	0.62	12.77	0.45	-20.75	37.78	58.53
	3.00	0.50	10.11	0.62	12.77	0.45	-20.83	37.78	58.61
	0.50	0.60	10.04	0.63	12.77	0.45	-21.38	40.00	61.38

	1.00	0.60	10.18	0.63	12.77	0.45	-20.28	40.00	60.28
	1.50	0.60	10.18	0.63	12.77	0.45	-20.28	40.00	60.28
	2.00	0.60	10.25	0.62	12.77	0.45	-19.73	37.78	57.51
	2.50	0.60	10.16	0.62	12.77	0.45	-20.44	37.78	58.22
	3.00	0.60	10.08	0.62	12.77	0.45	-21.06	37.78	58.84
**$BaUO_4$**	0.50	0.00	10.64	0.57	13.63	0.60	-21.94	-5.00	26.94
	1.00	0.00	10.61	0.58	13.63	0.60	-22.16	-3.33	25.49
	1.50	0.00	10.62	0.58	13.63	0.60	-22.08	-3.33	25.42
	2.00	0.00	10.59	0.57	13.63	0.60	-22.30	-5.00	27.30
	2.50	0.00	10.55	0.57	13.63	0.60	-22.60	-5.00	27.60
	3.00	0.00	10.51	0.57	13.63	0.60	-22.89	-5.00	27.89
	0.50	0.30	10.61	0.57	13.63	0.60	-22.16	-5.00	27.16
	**1.00**	**0.30**	**10.67**	**0.57**	**13.63**	**0.60**	**-21.72**	**-5.00**	**26.72**
	1.50	0.30	10.58	0.57	13.63	0.60	-22.38	-5.00	27.38
	2.00	0.30	10.59	0.57	13.63	0.60	-22.30	-5.00	27.30
	2.50	0.30	10.50	0.57	13.63	0.60	-22.96	-5.00	27.96
	3.00	0.30	10.42	0.57	13.63	0.60	-23.55	-5.00	28.55
	0.50	0.40	10.60	0.57	13.63	0.60	-22.23	-5.00	27.23
	1.00	0.40	10.63	0.57	13.63	0.60	-22.01	-5.00	27.01
	1.50	0.40	10.66	0.57	13.63	0.60	-21.79	-5.00	26.79
	2.00	0.40	10.55	0.57	13.63	0.60	-22.60	-5.00	27.60
	2.50	0.40	10.59	0.57	13.63	0.60	-22.30	-5.00	27.30
	3.00	0.40	10.37	0.57	13.63	0.60	-23.92	-5.00	28.92
	0.50	0.50	10.56	0.57	13.63	0.60	-22.52	-5.00	27.52
	1.00	0.50	10.59	0.58	13.63	0.60	-22.30	-3.33	25.64
	1.50	0.50	10.59	0.58	13.63	0.60	-22.30	-3.33	25.64
	2.00	0.50	10.55	0.57	13.63	0.60	-22.60	-5.00	27.60
	2.50	0.50	10.52	0.57	13.63	0.60	-22.82	-5.00	27.82
	3.00	0.50	10.49	0.57	13.63	0.60	-23.04	-5.00	28.04
	0.50	0.60	10.49	0.57	13.63	0.60	-23.04	-5.00	28.04
	1.00	0.60	10.57	0.57	13.63	0.60	-22.45	-5.00	27.45
	1.50	0.60	10.58	0.58	13.63	0.60	-22.38	-3.33	25.71
	2.00	0.60	10.45	0.57	13.63	0.60	-23.33	-5.00	28.33
	2.50	0.60	10.45	0.58	13.63	0.60	-23.33	-3.33	26.66
	3.00	0.60	10.45	0.57	13.63	0.60	-23.33	-5.00	28.33

Table S8: Comparison of calculations with experimental EELS results for the **U $N_{4,5}$-edge**. Energy separation between the first and second peaks $\Delta Peak$ (Peak II – Peak I, in eV) and relative intensity ratios $R_{Intensity}$ (Intensity I / Intensity II, in arbitrary units, a. u.) for uranium oxides. Percentage error of calculated vs experimental values for the peak separation ΔPeak and the $R_{Intensity}$ across the uranium oxide series. Errors were computed using Eq. (1) for $\Delta Peak$ error (%) and Eq. (2) for $R_{Intensity}$ error (%). Positive values indicate overestimation, while negative values indicate underestimation relative to experiment. The last column reports the total absolute error, defined as the sum of the absolute percentage errors in $\Delta Peak$ and $R_{Intensity}$, according to Eq. (3). Values highlighted in bold correspond to the selected Gaussian broadening and core-hole lifetime parameters that best reproduce the experimental spectra and preserve physical relevance. For $BaUO_4$ at the U $N_{4,5}$-edge, no comparison is possible because the experimental U N_4-edge is overlapped and obscured by the Ba M_4-edge.

Uranium $N_{4,5}$-edge

Uranium Oxides	Parameters		Calculation		Experiment		Percentage Error (%)		Absolute error
	Gaussian broadening	Core-hole lifetime	$\Delta Peak$	$R_{Intensity}$	$\Delta Peak$	$R_{Intensity}$	$\Delta Peak$ (%)	$R_{Intensity}$ (%)	$\lvert\Delta Peak\ (\%)\rvert + \lvert R_{Intensity}(\%)\rvert$
	0.50	0.00	41.02	1.32	41.32	1.82	-0.73	-27.63	28.36
	1.00	0.00	41.21	1.52	41.32	1.82	-0.27	-16.67	16.93
	1.50	0.00	41.32	1.59	41.32	1.82	0.00	-12.70	12.70
	2.00	0.00	41.41	1.61	41.32	1.82	0.22	-11.29	11.51
	2.50	0.00	41.41	1.61	41.32	1.82	0.22	-11.29	11.51
	3.00	0.00	41.44	1.61	41.32	1.82	0.29	-11.29	11.58
	0.50	2.50	41.36	1.56	41.32	1.82	0.10	-14.06	14.16
	1.00	2.50	41.47	1.56	41.32	1.82	0.36	-14.06	14.43
	1.50	2.50	41.51	1.56	41.32	1.82	0.46	-14.06	14.52
	2.00	2.50	41.51	1.54	41.32	1.82	0.46	-15.38	15.84
	2.50	2.50	41.54	1.54	41.32	1.82	0.53	-15.38	15.92
	3.00	2.50	41.57	1.52	41.32	1.82	0.61	-16.67	17.27
	0.50	3.50	41.48	1.52	41.32	1.82	0.39	-16.67	17.05
	1.00	3.50	41.50	1.52	41.32	1.82	0.44	-16.67	17.10
**UO_2**	1.50	3.50	41.51	1.52	41.32	1.82	0.46	-16.67	17.13
	2.00	3.50	41.51	1.52	41.32	1.82	0.46	-16.67	17.13
	2.50	3.50	41.55	1.49	41.32	1.82	0.56	-17.91	18.47
	3.00	3.50	41.57	1.47	41.32	1.82	0.61	-19.12	19.72
	0.50	4.50	41.50	1.47	41.32	1.82	0.44	-19.12	19.55
	1.00	4.50	41.52	1.47	41.32	1.82	0.48	-19.12	19.60
	1.50	4.50	41.53	1.47	41.32	1.82	0.51	-19.12	19.63
	2.00	4.50	41.56	1.45	41.32	1.82	0.58	-20.29	20.87
	2.50	4.50	41.55	1.45	41.32	1.82	0.56	-20.29	20.85
	3.00	4.50	41.57	1.43	41.32	1.82	0.61	-21.43	22.03
	0.50	5.50	41.53	1.43	41.32	1.82	0.51	-21.43	21.94
	**1.00**	**5.50**	**41.54**	**1.43**	**41.32**	**1.82**	**0.53**	**-21.43**	**21.96**
	1.50	5.50	41.54	1.43	41.32	1.82	0.53	-21.43	21.96
	2.00	5.50	41.56	1.41	41.32	1.82	0.58	-22.54	23.12
	2.50	5.50	41.58	1.41	41.32	1.82	0.63	-22.54	23.16
	3.00	5.50	41.59	1.39	41.32	1.82	0.65	-23.61	24.26
	0.50	0.00	41.59	1.39	41.38	1.82	0.51	-23.61	24.12
	1.00	0.00	41.59	1.39	41.38	1.82	0.51	-23.61	24.12
	1.50	0.00	41.59	1.39	41.38	1.82	0.51	-23.61	24.12
	2.00	0.00	41.61	1.39	41.38	1.82	0.56	-23.61	24.17
	2.50	0.00	41.57	1.39	41.38	1.82	0.46	-23.61	24.07

	3.00	0.00	41.58	1.39	41.38	1.82	0.48	-23.61	24.09
	0.50	2.50	41.60	1.39	41.38	1.82	0.53	-23.61	24.14
	1.00	2.50	41.61	1.41	41.38	1.82	0.56	-22.54	23.09
	1.50	2.50	41.60	1.39	41.38	1.82	0.53	-23.61	24.14
	2.00	2.50	41.58	1.39	41.38	1.82	0.48	-23.61	24.09
	2.50	2.50	41.58	1.39	41.38	1.82	0.48	-23.61	24.09
	3.00	2.50	41.59	1.39	41.38	1.82	0.51	-23.61	24.12
	0.50	3.50	41.59	1.39	41.38	1.82	0.51	-23.61	24.12
	1.00	3.50	41.60	1.39	41.38	1.82	0.53	-23.61	24.14
**U_4O_9**	1.50	3.50	41.58	1.39	41.38	1.82	0.48	-23.61	24.09
	2.00	3.50	41.63	1.41	41.38	1.82	0.60	-22.54	23.14
	2.50	3.50	41.58	1.39	41.38	1.82	0.48	-23.61	24.09
	3.00	3.50	41.57	1.39	41.38	1.82	0.46	-23.61	24.07
	0.50	4.50	41.57	1.39	41.38	1.82	0.46	-23.61	24.07
	1.00	4.50	41.58	1.39	41.38	1.82	0.48	-23.61	24.09
	1.50	4.50	41.59	1.39	41.38	1.82	0.51	-23.61	24.12
	2.00	4.50	41.60	1.39	41.38	1.82	0.53	-23.61	24.14
	2.50	4.50	41.60	1.39	41.38	1.82	0.53	-23.61	24.14
	3.00	4.50	41.57	1.39	41.38	1.82	0.46	-23.61	24.07
	0.50	5.50	41.59	1.39	41.38	1.82	0.51	-23.61	24.12
	**1.00**	**5.50**	**41.60**	**1.39**	**41.38**	**1.82**	**0.53**	**-23.61**	**24.14**
	1.50	5.50	41.58	1.41	41.38	1.82	0.48	-22.54	23.02
	2.00	5.50	41.58	1.41	41.38	1.82	0.48	-22.54	23.02
	2.50	5.50	41.60	1.39	41.38	1.82	0.53	-23.61	24.14
	3.00	5.50	41.59	1.39	41.38	1.82	0.51	-23.61	24.12

	0.50	0.00	41.50	1.45	41.40	1.85	0.24	-21.74	21.98
	1.00	0.00	41.50	1.47	41.40	1.85	0.24	-20.59	20.83
	1.50	0.00	41.50	1.49	41.40	1.85	0.24	-19.40	19.64
	2.00	0.00	41.51	1.49	41.40	1.85	0.27	-19.40	19.67
	2.50	0.00	41.51	1.49	41.40	1.85	0.27	-19.40	19.67
	3.00	0.00	41.45	1.49	41.40	1.85	0.12	-19.40	19.52
	0.50	2.50	41.48	1.43	41.40	1.85	0.19	-22.86	23.05
	1.00	2.50	41.50	1.43	41.40	1.85	0.24	-22.86	23.10
	1.50	2.50	41.53	1.43	41.40	1.85	0.31	-22.86	23.17
	2.00	2.50	41.54	1.41	41.40	1.85	0.34	-23.94	24.28
	2.50	2.50	41.56	1.41	41.40	1.85	0.39	-23.94	24.33
	3.00	2.50	41.50	1.39	41.40	1.85	0.24	-25.00	25.24
	0.50	3.50	41.51	1.39	41.40	1.85	0.27	-25.00	25.27
	1.00	3.50	41.51	1.39	41.40	1.85	0.27	-25.00	25.27
**U_3O_7**	1.50	3.50	41.53	1.39	41.40	1.85	0.31	-25.00	25.31
	2.00	3.50	41.52	1.37	41.40	1.85	0.29	-26.03	26.32
	2.50	3.50	41.45	1.37	41.40	1.85	0.12	-26.03	26.15
	3.00	3.50	41.45	1.35	41.40	1.85	0.12	-27.03	27.15
	0.50	4.50	41.50	1.35	41.40	1.85	0.24	-27.03	27.27
	1.00	4.50	41.52	1.35	41.40	1.85	0.29	-27.03	27.32
	1.50	4.50	41.54	1.33	41.40	1.85	0.34	-28.00	28.34
	2.00	4.50	41.46	1.33	41.40	1.85	0.14	-28.00	28.14
	2.50	4.50	41.46	1.33	41.40	1.85	0.14	-28.00	28.14
	3.00	4.50	41.45	1.32	41.40	1.85	0.12	-28.95	29.07
	0.50	5.50	41.53	1.32	41.40	1.85	0.31	-28.95	29.26
	**1.00**	**5.50**	**41.53**	**1.32**	**41.40**	**1.85**	**0.31**	**-28.95**	**29.26**
	1.50	5.50	41.45	1.30	41.40	1.85	0.12	-29.87	29.99
	2.00	5.50	41.46	1.30	41.40	1.85	0.14	-29.87	30.02
	2.50	5.50	41.49	1.28	41.40	1.85	0.22	-30.77	30.99

	3.00	5.50	41.52	1.28	41.40	1.85	0.29	-30.77	31.06
	0.50	0.00	41.51	1.22	42.32	1.59	-1.91	-23.17	25.08
	1.00	0.00	41.61	1.30	42.32	1.59	-1.68	-18.18	19.86
	1.50	0.00	41.61	1.37	42.32	1.59	-1.68	-13.70	15.38
	2.00	0.00	41.46	1.41	42.32	1.59	-2.03	-11.27	13.30
	2.50	0.00	41.53	1.43	42.32	1.59	-1.87	-10.00	11.87
	3.00	0.00	41.49	1.43	42.32	1.59	-1.96	-10.00	11.96
	0.50	2.50	41.50	1.37	42.32	1.59	-1.94	-13.70	15.64
	1.00	2.50	41.51	1.37	42.32	1.59	-1.91	-13.70	15.61
	1.50	2.50	41.48	1.37	42.32	1.59	-1.98	-13.70	15.68
	2.00	2.50	41.50	1.37	42.32	1.59	-1.94	-13.70	15.64
	2.50	2.50	41.56	1.37	42.32	1.59	-1.80	-13.70	15.49
	3.00	2.50	41.49	1.35	42.32	1.59	-1.96	-14.86	16.83
	0.50	3.50	41.54	1.35	42.32	1.59	-1.84	-14.86	16.71
	1.00	3.50	41.46	1.35	42.32	1.59	-2.03	-14.86	16.90
**KUO_3**	1.50	3.50	41.50	1.33	42.32	1.59	-1.94	-16.00	17.94
	2.00	3.50	41.52	1.33	42.32	1.59	-1.89	-16.00	17.89
	2.50	3.50	41.55	1.33	42.32	1.59	-1.82	-16.00	17.82
	3.00	3.50	41.47	1.32	42.32	1.59	-2.01	-17.11	19.11
	0.50	4.50	41.45	1.32	42.32	1.59	-2.06	-17.11	19.16
	1.00	4.50	41.47	1.32	42.32	1.59	-2.01	-17.11	19.11
	1.50	4.50	41.50	1.30	42.32	1.59	-1.94	-18.18	20.12
	2.00	4.50	41.52	1.30	42.32	1.59	-1.89	-18.18	20.07
	2.50	4.50	41.46	1.30	42.32	1.59	-2.03	-18.18	20.21
	3.00	4.50	41.51	1.28	42.32	1.59	-1.91	-19.23	21.14
	0.50	5.50	41.47	1.28	42.32	1.59	-2.01	-19.23	21.24
	**1.00**	**5.50**	**41.52**	**1.28**	**42.32**	**1.59**	**-1.89**	**-19.23**	**21.12**
	1.50	5.50	41.51	1.27	42.32	1.59	-1.91	-20.25	22.17
	2.00	5.50	41.53	1.27	42.32	1.59	-1.87	-20.25	22.12
	2.50	5.50	41.48	1.27	42.32	1.59	-1.98	-20.25	22.24
	3.00	5.50	41.49	1.25	42.32	1.59	-1.96	-21.25	23.21
	0.50	0.00	41.51	1.25	41.83	1.72	-0.77	-27.50	28.27
	1.00	0.00	41.41	1.43	41.83	1.72	-1.00	-17.14	18.15
	1.50	0.00	41.47	1.49	41.83	1.72	-0.86	-13.43	14.29
	2.00	0.00	41.51	1.52	41.83	1.72	-0.77	-12.12	12.89
	2.50	0.00	41.44	1.54	41.83	1.72	-0.93	-10.77	11.70
	3.00	0.00	41.46	1.52	41.83	1.72	-0.88	-12.12	13.01
	0.50	2.50	41.51	1.45	41.83	1.72	-0.77	-15.94	16.71
	1.00	2.50	41.42	1.45	41.83	1.72	-0.98	-15.94	16.92
	1.50	2.50	41.44	1.45	41.83	1.72	-0.93	-15.94	16.87
	2.00	2.50	41.47	1.43	41.83	1.72	-0.86	-17.14	18.00
	2.50	2.50	41.49	1.43	41.83	1.72	-0.81	-17.14	17.96
	3.00	2.50	41.50	1.41	41.83	1.72	-0.79	-18.31	19.10
	0.50	3.50	41.44	1.41	41.83	1.72	-0.93	-18.31	19.24
	1.00	3.50	41.46	1.41	41.83	1.72	-0.88	-18.31	19.19
**U_3O_8**	1.50	3.50	41.47	1.39	41.83	1.72	-0.86	-19.44	20.31
	2.00	3.50	41.48	1.39	41.83	1.72	-0.84	-19.44	20.28
	2.50	3.50	41.49	1.37	41.83	1.72	-0.81	-20.55	21.36
	3.00	3.50	41.51	1.37	41.83	1.72	-0.77	-20.55	21.31
	0.50	4.50	41.48	1.37	41.83	1.72	-0.84	-20.55	21.38
	1.00	4.50	41.49	1.35	41.83	1.72	-0.81	-21.62	22.43
	1.50	4.50	41.49	1.35	41.83	1.72	-0.81	-21.62	22.43
	2.00	4.50	41.49	1.35	41.83	1.72	-0.81	-21.62	22.43

	2.50	4.50	41.50	1.33	41.83	1.72	-0.79	-22.67	23.46
	3.00	4.50	41.52	1.32	41.83	1.72	-0.74	-23.68	24.43
	0.50	5.50	41.49	1.32	41.83	1.72	-0.81	-23.68	24.50
	**1.00**	**5.50**	**41.50**	**1.32**	**41.83**	**1.72**	**-0.79**	**-23.68**	**24.47**
	1.50	5.50	41.51	1.30	41.83	1.72	-0.77	-24.68	25.44
	2.00	5.50	41.49	1.30	41.83	1.72	-0.81	-24.68	25.49
	2.50	5.50	41.49	1.30	41.83	1.72	-0.81	-24.68	25.49
	3.00	5.50	41.40	1.28	41.83	1.72	-1.03	-25.64	26.67
	0.50	0.00	41.53	1.28	N/A	N/A	N/A	N/A	N/A
	1.00	0.00	41.54	1.28	N/A	N/A	N/A	N/A	N/A
	1.50	0.00	41.55	1.27	N/A	N/A	N/A	N/A	N/A
	2.00	0.00	41.58	1.27	N/A	N/A	N/A	N/A	N/A
	2.50	0.00	41.52	1.27	N/A	N/A	N/A	N/A	N/A
	3.00	0.00	41.53	1.25	N/A	N/A	N/A	N/A	N/A
	0.50	2.50	41.53	1.39	N/A	N/A	N/A	N/A	N/A
	1.00	2.50	41.58	1.39	N/A	N/A	N/A	N/A	N/A
	1.50	2.50	41.49	1.37	N/A	N/A	N/A	N/A	N/A
	2.00	2.50	41.53	1.37	N/A	N/A	N/A	N/A	N/A
	2.50	2.50	41.58	1.37	N/A	N/A	N/A	N/A	N/A
	3.00	2.50	41.51	1.37	N/A	N/A	N/A	N/A	N/A
	0.50	3.50	41.47	1.35	N/A	N/A	N/A	N/A	N/A
	1.00	3.50	41.49	1.35	N/A	N/A	N/A	N/A	N/A
**$BaUO_4$**	1.50	3.50	41.52	1.35	N/A	N/A	N/A	N/A	N/A
	2.00	3.50	41.55	1.33	N/A	N/A	N/A	N/A	N/A
	2.50	3.50	41.49	1.33	N/A	N/A	N/A	N/A	N/A
	3.00	3.50	41.52	1.32	N/A	N/A	N/A	N/A	N/A
	0.50	4.50	41.49	1.32	N/A	N/A	N/A	N/A	N/A
	1.00	4.50	41.54	1.32	N/A	N/A	N/A	N/A	N/A
	1.50	4.50	41.55	1.30	N/A	N/A	N/A	N/A	N/A
	2.00	4.50	41.59	1.30	N/A	N/A	N/A	N/A	N/A
	2.50	4.50	41.50	1.30	N/A	N/A	N/A	N/A	N/A
	3.00	4.50	41.57	1.28	N/A	N/A	N/A	N/A	N/A
	0.50	5.50	41.55	1.28	N/A	N/A	N/A	N/A	N/A
	1.00	5.50	41.56	1.28	N/A	N/A	N/A	N/A	N/A
	1.50	5.50	41.58	1.28	N/A	N/A	N/A	N/A	N/A
	2.00	5.50	41.58	1.27	N/A	N/A	N/A	N/A	N/A
	2.50	5.50	41.53	1.27	N/A	N/A	N/A	N/A	N/A
	3.00	5.50	41.56	1.25	N/A	N/A	N/A	N/A	N/A

References

(1) Bunău, O.; Ramos, A. Y.; Joly, Y. The FDMNES Code. **2024**.
(2) Bunău, O.; Joly, Y. Self-Consistent Aspects of x-Ray Absorption Calculations. *Journal of Physics: Condensed Matter* **2009**, *21* (34), 345501.
(3) Rehr, J. J.; Kas, J. J.; Prange, M. P.; Sorini, A. P.; Takimoto, Y.; Vila, F. Ab Initio Theory and Calculations of X-Ray Spectra. *Comptes Rendus Physique* **2009**, *10* (6), 548–559.
(4) Blaha, P.; Schwarz, K.; Tran, F.; Laskowski, R.; Madsen, G. K.; Marks, L. D. WIEN2k: An APW+ Lo Program for Calculating the Properties of Solids. *The Journal of chemical physics* **2020**, *152* (7).
(5) Clark, S. J.; Segall, M. D.; Pickard, C. J.; Hasnip, P. J.; Probert, M. I.; Refson, K.; Payne, M. C. First Principles Methods Using CASTEP. *Zeitschrift für kristallographie-crystalline materials* **2005**, *220* (5–6), 567–570.
(6) Stavitski, E.; De Groot, F. M. The CTM4XAS Program for EELS and XAS Spectral Shape Analysis of Transition Metal L Edges. *Micron* **2010**, *41* (7), 687–694.
(7) Neese, F. The ORCA Program System. *WIRES Comput. Molec. Sci.*, 2012, *2*, 73–78. https://doi.org/10.1002/wcms.81.
(8) Hébert-Souche, C.; Louf, P.-H.; Blaha, P.; Nelhiebel, M.; Luitz, J.; Schattschneider, P.; Schwarz, K.; Jouffrey, B. The Orientation-Dependent Simulation of ELNES. *Ultramicroscopy* **2000**, *83* (1–2), 9–16. https://doi.org/10.1016/S0304-3991(99)00168-0.
(9) Henderson, G. S.; De Groot, F. M.; Moulton, B. J. X-Ray Absorption near-Edge Structure (XANES) Spectroscopy. *Reviews in Mineralogy and Geochemistry* **2014**, *78* (1), 75–138.
(10) Bannister, M. J. The Storage Behaviour of Uranium Dioxide Powders — Review Article. *Journal of Nuclear Materials* **1968**, *26* (2), 174–184. https://doi.org/10.1016/0022-3115(68)90069-X.
(11) Leinders, G.; Cardinaels, T.; Binnemans, K.; Verwerft, M. Low-Temperature Oxidation of Fine UO_2 Powders: Thermochemistry and Kinetics. *Inorg. Chem.* **2018**, *57* (7), 4196–4204. https://doi.org/10.1021/acs.inorgchem.8b00517.
(12) Leinders, G.; Pakarinen, J.; Delville, R.; Cardinaels, T.; Binnemans, K.; Verwerft, M. Low-Temperature Oxidation of Fine UO_2 Powders: A Process of Nanosized Domain Development. *Inorg. Chem.* **2016**, *55* (8), 3915–3927. https://doi.org/10.1021/acs.inorgchem.6b00127.
(13) Leinders, G.; Bes, R.; Kvashnina, K. O.; Verwerft, M. Local Structure in U(IV) and U(V) Environments: The Case of U_3O_7. *Inorg. Chem.* **2020**, *59* (7), 4576–4587. https://doi.org/10.1021/acs.inorgchem.9b03702.
(14) Bes, R.; Leinders, G.; Kvashnina, K. Application of Multi-Edge HERFD-XAS to Assess the Uranium Valence Electronic Structure in Potassium Uranate (KUO_3). *J Synchrotron Rad* **2022**, *29* (1), 21–29. https://doi.org/10.1107/S1600577521012431.
(15) Daemen, F. Bereiding En Karakterisatie van Ternaire Uranium-Zuurstof Verbindingen, Hogeschool Antwerpen, 2005.
