# Supplementary material for: Fingerprinting Uranium Oxides with Electron Energy Loss Spectroscopy Supported by Theoretical Computations
Source: J Phys Chem A. 2026 Mar 4;130(11):2329–37. doi: 10.1021/acs.jpca.5c07789 (PMC13007028; doi:10.1021/acs.jpca.5c07789)
Supplement: Supplementary file 2 [file jp5c07789_si_002.zip › FDMNES_files/Manual_Eng.pdf]

# FDMNES
# User's Guide

Yves Joly

yves.joly@neel.cnrs.fr
Institut Néel, CNRS, BP 166
38042 Grenoble Cedex 9, France

October 2025

# Outline


| | |
|---|---|
| Introduction | 5 |
| A) General Presentation | 7 |
| B) Some advices to make the best possible simulation | 11 |
| C) Main input file | 15 |
| D) Convolution | 61 |
| E) Parameter optimization | 71 |
| F) Extraction of DAFS scan and spectra | 77 |
| G) Unit cell modification | 79 |
| H) FDMX user's guide | 81 |
| I) 2D diffraction | 85 |
| List of the *fdmnes* keywords | 95 |

# Introduction

The FDMNES program calculates the spectra of different spectroscopies related to the real or virtual absorption of x-ray in material. It gives the absorption cross sections around the ionization edges, that is in the XANES energy range. Calculations can be performed along all the conditions of linear or circular polarization. In the same way, it calculates the structure factors and intensities of anomalous or resonant diffraction spectra (DAFS or RXD) for 3D diffraction and for surface diffraction (SRXRD). FDMNES also allows the comparison of the simulated spectra to experimental ones with the help of objective criteria.

FDMNES is mainly a fully relativistic DFT-LSDA code. Optionally Hubbard correction (LSDA+U) can be used. It uses two techniques. The first one is based on the Finite Difference Method (FDM) to solve the Schrödinger equation. In that way the shape of the potential is free and in particular avoid the muffin-tin approximation. The second one uses the Green formalism (multiple scattering) on a muffin-tin potential. This approach can be less precise but is faster. The program includes also a multi-electronic extension using the "Time-Dependent DFT" with a local kernel. The program is symmetrized. Symmetry operations are calculated automatically.

The FDMNES program can be freely downloaded at the web address:
https://fdmnes.neel.cnrs.fr

In case of publication related to the use of the program thanks to cite:
O. Bunau and Y. Joly
*Self-consistent aspects of x-ray absorption calculations*
J. Phys. : Condens. Matter **21**, 345501 (2009).

The FDMNES program highly benefited from the scientific contribution of Calogero Natoli who has provided a constant and essential help. He is in particular at the origin of all the developments using the multiple scattering theory and the extensions to resonant diffraction and magnetism. Oana Bunau realized the extension towards TD-DFT and participated to the inclusion of self-consistency and Hubbard correction. The program also benefited from the expertise of Delphine Cabaret, Hubert Renevier, Sergio Di Matteo, Christian Brouder, Stéphane Grenier, Emilio Lorenzo, Yvonne Soldo and Yvonne Gründer, without whom many of the advances would not have been possible. Finally, this work was greatly facilitated by the support of Denis Raoux.

From March 2015, Guda, Soldatov *et al.* (Rostov-on-Don, Russia) make the code far faster by the use of the MUMS library which solves systems of linear equations for sparse matrices. This method is now the default option used when running in the finite difference method mode. When using it thanks to cite also:

Sergey A. Guda, Alexander A. Guda, Mikhail A. Soldatov, Kirill A. Lomachenko, Aram L. Bugaev, Carlo Lamberti, Wojciech Gawelda, Christian Bressler, Grigory Smolentsev, Alexander V. Soldatov, Yves Joly
"*Optimized Finite Difference Method for the Full-Potential XANES Simulations: Application to Molecular Adsorption Geometries in MOFs and Metal-Ligand Intersystem Crossing Transients*" J. Chem. Theory Comput. **11**, 4512-4521 (2015).
and
P. R. Amestoy, A. Guermouche, J.-Y. L'Excellent and S. Pralet
*Hybrid scheduling for the parallel solution of linear systems*
Parallel Computing Vol 32 (2), pp 136-156 (2006).

From October 2015, the code also includes the FDMX extension from J. Bourke and Ch. Chantler. When using it, thanks to cite also:

Jay Daniel Bourke, Christopher Thomas Chantler and Yves Joly
"*Extended X-ray Absorption Fine Structure Calculations Using the Finite Difference Method*"
J. Synchrotron Rad. 23, 551-559 (2016).

To have an overview of X-ray absorption spectroscopy, we recommend the reading of:
*X-Ray Absorption and X-ray Emission Spectroscopy: Theory and Applications*
Edited by J. A. van Bokhoven and C. Lamberti, Wiley (2016).
ISBN: 978-1-118-84423-6.

The work presented here concerns more specifically the chapter 4:
"*Theory of X-ray Absorption Near Edge Structure*" by Yves Joly and Stéphane Grenier.

Another of our publications gives the main concepts of resonant diffraction:
"*Basics of Resonant Elastic X-ray Scattering theory*"
by S. Grenier and Y. Joly, J. Phys.: Conference Series **519**, 012001 (2014).

Surface resonant x-ray diffraction and x-ray Raman spectroscopy are respectively given in:
Yves Joly, Antoine Abisset, Aude Bailly, Maurizio De Santis, Farid Fettar, Stéphane Grenier, Danny Mannix, Aline Y. Ramos, Marie-Claire Saint-Lager, Yvonne Soldo-Olivier, Jean-Marc Tonnerre, Sergey A. Guda, and Yvonne Gründer.
"*Simulation of surface resonant x-ray diffraction*"
J. Chem. Theory Comput. **14**, 973-980 (2018).
DOI: 10.1021/acs.jctc.7b01032.

Yves Joly, Chiara Cavallari, Sergey A. Guda, and Christoph J. Sahle
"*Full potential simulation of x-ray Raman scattering spectroscopy*"
J. Chem. Theory Comput. 13, 2172-2177 (2017).
DOI: 10.1021/acs.jctc.7b00203.

The FDMNES code and the Finite Difference method are presented in 2 sections of the *International Tables for Crystallography, Volume I: X-ray Absorption Spectroscopy and Related Techniques* (2021 & 2022)
ISBN: 978-1-119-43394-1

*The FDMNES code*
by O. Bunău, A. Y. Ramos, and Y. Joly
DOI: 10.1107/S1574870720003304

*Finite-difference method for the calculation of X-ray spectroscopies*
by Y. Joly, A. Y. Ramos, and O. Bunău
DOI: 10.1107/S1574870722001598

# A- General presentation

## I- Computer configuration

FDMNES run on all the computers, under LINUX, Mac or Windows. The programming language is Fortran 2003. Executables are furnished for Windows 64 bits, Linux 64 bits for sequential calculations and for parallel calculations under MPI.

Users can also compile the code themselves. The code needs then MUMPS, LAPACK and BLAS libraries (and MPI for parallel).

## II- The packages

Different packages for the different operating systems (Windows 64, Linux 64, Mac OS) can be downloaded. They contain the corresponding executable, a set of examples of input files, the user's guide and other information:

- *fdmnes_win64.exe*: executable for Windows 64 bits,
- *fdmnes_linux64*: executable for linux 64 bits,
- *fdmnes_mac:* executable for Mac OS,
- *run_fdmnes_command*: command for Mac OS to execute fdmnes_mac. When clicking on it, the working directory of fdmnes_mac is the current.
- *fdmfile.txt*: input file,
- *Sim/Test_stand/in*: directory containing a set of examples of *fdmnes* input files.
- *Doc*: directory containing information on the code, the manual, the file "FDMNES_Modifications.txt" where are listed the modifications done in the code along the time. For Mac contains also *"Instructions_for_Mac*" which gives explanations to make the mac version running

The fortran routines with example of makefile can also be downloaded as well as an executable for the parallel version of the code

## IV- Compilation

For those who do not want or cannot use the executables of the package, it is possible to compile the fortran routines. It uses the MUMPS library. All the routines, but *mat_solve_gaussian.f90*, *sub_util.f* and *not_mpi.f90*, must then be compiled and linked with the call to this library and the ones whom it depends (SCOTCH, METIS, BLAS, LAPACK…). The files included in this step (*mpif.h*, …) must be set in the same directory (or in a dedicated one).

An example of "makefile" for linux is provided.

## V- Parallelization

Thanks to Sergey Guda, Keisuke Hatada, Kuniko Hayakawa and Rainer Wilcke, the users having the access to a cluster of computers can, using the MPI library, run the program in parallel mode.

## VI- Running

After compilation, the program can be run following the usual procedure available on your system.

As soon as the program is running, it calls the file "*fdmfile.txt*". This file must also be in the same directory than the executable file. It only contains the number of independent calculation to perform, followed the name of the input file of each of these calculations. For example:

| | |
|---|---|
| *! Input file for fdmnes* | |
| *1* | → number of input files |
| *Sim/cu/in/cu_inp.txt* | → name of the input file |

When running in parallel, with MPI, one can use the executable given for this purpose, or another one compiled by the user with the MUMPS library. There are 2 levels of parallelization in FDMNES. The first one is a simple one inside the loop over energy. The second one, optional works inside the MUMPS library. It is useful only when working using the finite difference method.

Typically, in the first case, the command to send the job is:

```
mpirun   -np 16   fdmnes
```

The calculation uses then 16 processors in parallel.

To use the 2 parallelism one typically must write:

```
mpirun   -np 16 -x HOST_NUM_FOR_MUMPS=2   fdmnes
```

In that case, 16 processors are used, 8 energies will be calculated in parallel. For each of them, 2 processors run the MUMPS library.

## VII- Troubleshooting

When the program stops without reason, check if an "*fdmnes_error.txt*" file has been created. When created, this file contains a message explaining the trouble. Most often it is due to an error in the input file. It can also be due to the fact that the input file is not found. One has to check several points:

1) The called files are in the good directory. The name must contain all the paths.
2) The extensions are correct.
3) Their names are OK (under Linux, upper and lower cases must be respected).

4) When using downloaded input files, some problems of compatibility between systems can occur. It can be better to write again completely these files.

When the program stops without "*fdmnes_error.txt*" file, it can be due to a problem of space memory. Sometimes one gets a message with "stacking fault". In this case try again putting in the input file the keyword "Memory_save". This keyword can be useful when there are many non-equivalent atoms. An approximation (in fact very good) is then done on the potential calculation. This option saves some memory space.

## VIII- Structure of the calculation

For a complete calculation one has the following scheme:

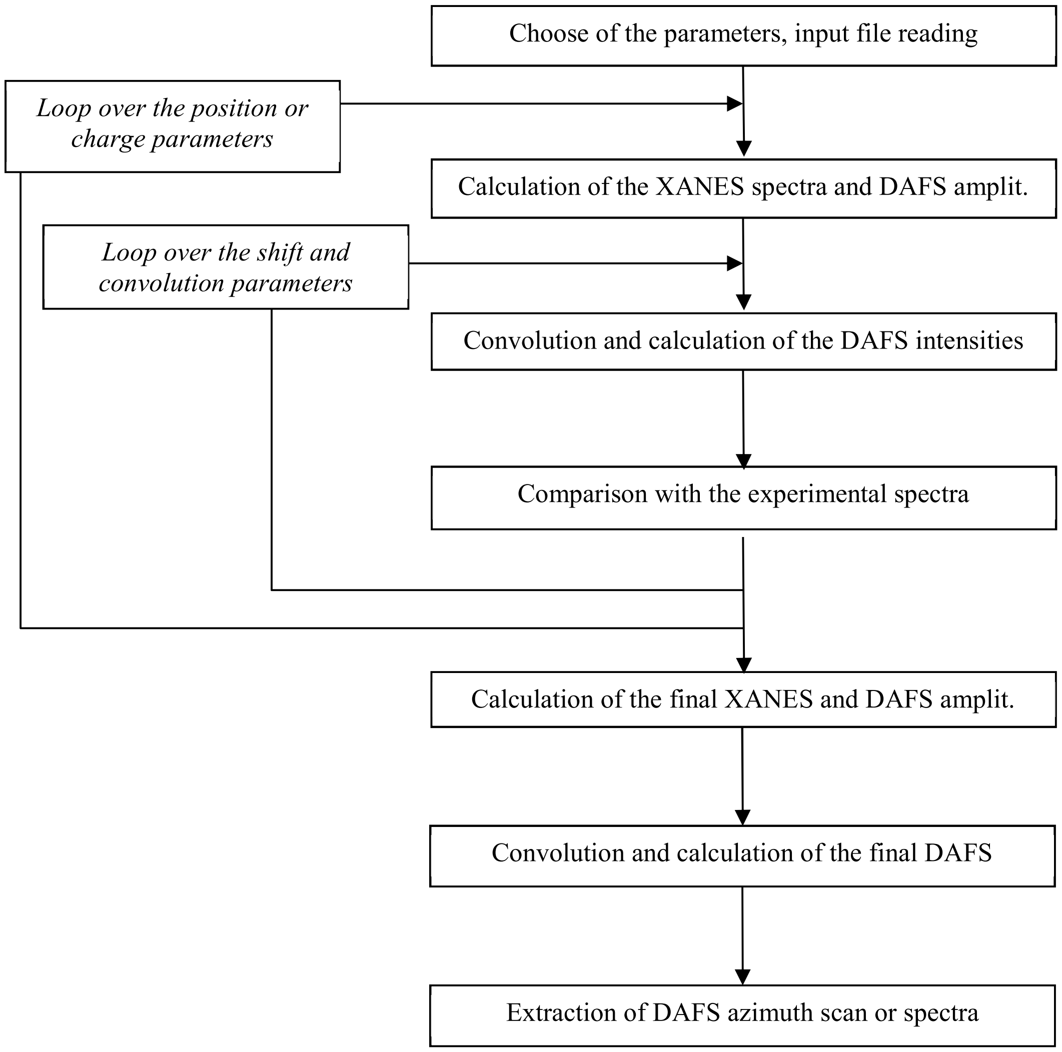


The program allows the calculation of spectra from a grid of parameters (position, charge…), to convolute them with other parameters (convolution width, energy shift…), then to compare them to experimental spectra with the help of objective criteria. These different steps can be performed together or separately. The comparison with the experimental spectra is also not mandatory. When used, the parameter fit must be performed with care. In practice

many calculations are limited to the step "XANES and DAFS calculation" and "Convolution and calculation of DAFS intensities". These two steps can also be performed together or separately.

In the output files, the absorption cross section are in Mbarn (1 Mbarn = $10^{-18}$ $cm^2$) and summed up over the atoms of same chemical specie in the unit cell or in the cluster. To convert in number of electron one has to multiply by $C = \frac{\hbar\omega_{eV}}{800\pi^2 a_0^2 \alpha R} = 0.004555352 \times \hbar\omega_{eV}$, where $R$, $\alpha$ and $a_0$ are respectively the Rydberg constant, the fine structure constant and the Bohr radius in Angstrom, $\hbar\omega_{eV}$ is the photon energy in eV. One has also to divide by the number of atoms if one wants the result per atom. The intensities of the reflections are in square of number of electrons.

The next chapter treats about the principal input file for the step "XANES and DAFS calculation". Generally, this file is sufficient to describe all the necessary data for the calculation because the program calculates its atomic bases and the potential. Nevertheless, the user can prefer use its own atomic bases or uses directly the potential calculated by the band structure program FLAPW WIEN-2k. In both cases, some other files must be furnished. They are described further in the manual. The input necessary for the steps "Convolution", "comparison with the experimental spectra" and "Extraction of azimuth scan or spectra" can be set in the same input file, but they are explained separately in the sections D, E and F.

# B-Some advices to make the best possible simulation

The purpose of this little section is to help the user of this code to obtain the best possible simulation, considering the limitations of the program.

## 1) DFT

FDMNES is a DFT program with a TDDFT extension. DFT is a ground state theory. It is thus in principal false to calculate the exciting states, the ones probed by x-ray absorption spectroscopies …
But we do it anyway. Indeed, at least for the K-edges of all elements and the $L_2$ and $L_3$ edges of heavy elements (that is from, say, Z = 31), and with some tried and tested options, it is possible to obtain very satisfactory agreement. This leads to the following setting:

➔ **The simulation of K (or $L_1$) edge of all elements or $L_2$ or $L_3$ edges of heavy elements must give a convenient result**

By convenient, one means that most features of the data must be reproduced when the atomic structure is known. They must be reproduced in intensity at typically less than 10% and in position at less than some eV. Often the agreement can be even better.

We can anyway also calculate the other edges. For example $L_{23}$ edges of the first half of the *3d* elements is improved by the use of the theory (and keyword) "*TDDFT*", especially for the ratio $L_2/L_3$. One nevertheless must not expect a reproduction of the excitonic states which gives the so-called "multiplet" features often observed for example in oxides.
We can also get interesting results playing with screening parameters and option of self-consistency for the heavier elements.

## 2) First calculation

For the first calculation of a new material, one must always start with the simplest input file, that is:

1) Keep all the *fdmnes* default options and so describing only the atomic structure, the energy range and terminating with a "*Convolution*"
2) Make the calculation with a small radius ("*Radius*" = 3, for example)
3) Use the multiple scattering theory (keyword "*Green*")
4) No self-consistent calculation

An example for magnetite:

```
Filout
Sim/Test/Fe3O4

 Range
  -5. 0.1 5. 0.2  10.  0.5 20. 1. 30. 2. 60. 4. 100.

 Radius
  3.0

 Green

Spgroup
  Fd-3m:1

 Crystal
     8.3940   8.3940   8.3940   90.0   90.0   90.0

  26   .6250    .6250    .6250
  26   .0000    .0000    .0000
   8   .3800    .3800    .3800

 Convolution

End
```

One must remember that by default:

- The absorbing chemical specie is the first one in the list under "*Crystal*" (or "*Molecule*"). If it is not the case, use the keyword "*Z_absorber*" and below write the absorbing atomic number.
- The absorption edge is K, in the other case use the keyword "*Edge*".

The spectrum one gets by such a calculation must already "look like" the experimental spectrum. Pre-edges can be not well reproduced, some structures can be missing or shifted, but the general envelope of the spectra must be good. In the other case, there is a problem…
If it is the case, the first thing to check is the atomic structure. It can be the case for example when the structure comes from a *cif* file obtained by the use of "*Cif_file*" keyword. *Cif* files often come from structural refinements and they may contain multiple configurations with occupancy rate less than 1.  This leads to atoms apparently too close, and the XANES calculation takes all the atoms…
In any event, the output file with the extension "*bav*" contains all the useful information on the structure.

- Looking for "Symsite" one finds the information related to the space group, the number of non-equivalent atoms and for each of them the symmetry operations making the relation to its equivalent atoms.
- Further, and for each absorbing atom, non-equivalent by symmetry, one finds the cluster description around it. For this, search "Agregat". Just below it is written the punctual group and a bit further the cluster arranged by increasing distance from its center. When there is a mistake in the structure, very often pathological positions are evident (for

example the number of first neighbors are not conform, or interatomic distances are evidently false because not physical.

➔ **For any new calculation check in the "*bav*" file the structure**

This checking is in any case interesting because you can find there the distances, the number of atoms and the chemical species of the different shells.

### 3) Following calculations

The previous step being done, one has now to improve the result. For this try the following procedures roughly in this order (see in the manual the significance and the precise syntax of the keywords given below):

1) When there are heavy elements in the structure (say Z >50) (not necessarily the absorbing atom) use the keyword "*Relativism*" or "*Spinorbit*" (with this last, the calculation is more expensive…)

2) For the calculation of molecules which are not in gas phase, use the keyword "*Vmax*" with typical value -6 eV written below.

3) Progressively increase the calculation radius, up to convergence (after convolution). Typically, one needs to go up to 5-7 Å.

4) Try the exchange-correlation potential GGA, PBE96, in principle better with the keyword « PBE96 ».

5) If agreement is insufficient, use the finite difference method mode (suppressing the keyword "*Green*"); begin again with the small radius then increase it…

6) Make a self-consistent calculation with keyword "*SCF*". In most cases at this stage, the agreement is already sufficient, but possibly at the very beginning of the edge.

7) By default, in *fdmnes*, at the K, $L_1$, $M_1$ and $O_1$ edges, self-consistency is done without core-hole, the core-hole plus its screening charge being added just at the XANES step. When the agreement is not yet sufficiently good, particularly at the rising edge, this can be modified using "*SCFexc*" (core-hole already present during the SCF), then "S*CFexc*v plus a screening smaller than 1 with the keyword "*Screening*". For insulating compounds, this last mode can be better. Some software for XANES use the equivalent of "*SCFexc*" and "*Screening*" = 0 as default option. Note that for $L_{23}$, $M_{23}$ and $M_{45}$ edges the complete calculation is done by default in the ground state (no core-hole). Check in the manual the significance of "*SCFexc*", "*Nonexc*" and "*Excited*" options to modify all this. Finally, there is no half core-hole option in *fdmnes*, because the author does not understand what this means…
Sometimes, and specifically for oxides, it can be noted that SCF leads to solutions not converging versus the cluster radius. One observes for instance a beating phenomenon on the atomic charges, versus the number of atomic shells incoming in the calculation area. This can be overcome by the use of the keyword "*Full_atom*" which suppress an approximation, taking the cluster atoms equivalent by the space group symmetry instead of the punctual group symmetry.

8) For the K-edge of *3d*, *4d* or *5d* elements or $L_{23}$ edges of heavy elements, test the occurrence of quadrupolar transitions (keyword "*Quadrupole*").

9) Convolution can be improved. This one has a default width a bit too large. One can reduce it by the keyword "*Gamma_max*". When comparing with an experiment recorded in fluorescence high resolution mode, one must reduce the core level width with the keyword "*Gamma_hole*". Sometimes it is convenient to shift the Fermi level (or cutting energy) which is badly defined due to the core-hole, using the keyword "*E_cut*". This is especially the case when using "*Screening*" option. The temperature effect can be roughly considered with the keyword "*Abs_U_iso*". Note that it is automatically considered when the mean square displacement, U, is included in the *cif* file. One must remember that the efficient *U* in XANES is smaller than in diffraction. Finally note that the convolution can be performed a part from the main calculation. It is a way to save a lot of time (see chapter Convolution).

## 4) Other outputs

1) Dichroism can be calculated using keyword « *Polarized* ». For the calculation of XMCD and for magnetic material in general, the magnetic configuration state must be imposed (with keywords "*Magnetism*" or "*Spinorbit*" and "*Atom_conf*" or "*Atom*"). We recall here that without spin-orbit (on the conduction band states), there is no XMCD at K or $L_1$ edges.

2) Because the absorption spectroscopy calculations need first the calculation of the density of states, this one can be given as extra output of the code. The projected density of states can be obtained by keyword the "*Density*" and "*Density_all*". The Crystal overlap orbital population can be obtained with keywords "*COOP*".

3) When a main calculation has been done (with a specific radius), many results on the absorbing atoms are in the *bav* file. Consequently, new calculations on the same structure and with the same radius can be done very quickly reading this *bav* file with the keyword "*Extract*". By this way one can calculate specific polarizations (or DAFS reflections), suppress or include the calculation of quadrupole, or, dipole, demand for output of the Density (but not the COOP).

4) Results on XES (only valence to core), X-ray Raman, resonant diffraction and surface resonant diffraction can also be obtained (see the corresponding sections).

# C- Main input file

## I- General Structure

It contains most of the inputs necessary for the calculation. All the data in input and output are in Angstrom and electron-Volt. Many options are chosen by default. One can modify or add other options using keywords. Text can be in upper or lower case. Blank lines or beginning by "!" are not considered. Between number, one must put at least one blank. When getting problem when opening these input files, one has to check if their name is correct. Moreover, some compilers do not like files written under other system (MAC, DOS, LINUX…). In case of difficulties when the program wants to open one of these downloaded files, it can be useful to completely write them again.

The input file contains several blocks of data, each one starting with a specific keyword. The end of the input file is noted by the keyword "*End*". Whatever is after is not red. Here comes an example of input file:

*! Fdmnes input file*
*! Calculation for the copper K-edge in copper cfc*

| | |
|---|---|
| *Filout* | |
| *Sim/Cu/Cu_out* | → Name of the output files (without extension) |
| | |
| *Range* | |
| *-2. 0.2 5. 0.5 10. 1. 40.* | |
| | |
| *Radius* | → Cluster radius |
| *3.0* | |
| | |
| *Crystal* | → crystal structure |
| *3.610 3.610 3.610 90. 90. 90.* | → Unit cell parameters (Å and degrees) : a, b, c, α, β, γ |
| *29 0.0 0.0 0.0* | → Atomic number, position |
| *29 0.5 0.5 0.0* | |
| *29 0.5 0.0 0.5* | |
| *29 0.0 0.5 0.5* | |
| | |
| *Convolution* | → to get convoluted spectra |
| | |
| *End* | → end of the input file |

Two blocks are necessary for any calculations. The first one starts with the keyword "*Radius*" followed by the value of the radius of the sphere surrounding the area of calculation. The second one is necessary to describe the material structure. Which can be a molecule or a 3D or 2D periodical structure. This description follows respectively the keywords "*Molecule*", "*Crystal*" or "Surface".

All the keywords related to the convolution or to the fit of the parameters are treated in chapter C and D.

## Output file names

By default the output file name is *fdmnes_out*. This name can be modified by the use of the keyword "*filout*" followed by the name we want (without extension). Then one gets several output files with the extensions:

| | |
|---|---|
| _bav.txt | output file giving details |
| .txt | contains only the spectra by column |
| _nrixs.txt | contains only the spectra by column for NRIXS simulations |

If a calculation is performed on several non-equivalent crystallographic sites, one gets the extensions:

_i.txt, _j.txt … in which i and j are the index of the sites (see keyword *absorber*)

In option or depending on the type of calculation, one can also get the files:

| | |
|---|---|
| _conv.txt | convoluted spectra scan (keyword *Convolution*). |
| _scan.txt | dafs versus angles for azimuthal scan (keyword *DAFS*). |
| _sda.txt | state density for the atom number a (keyword *Density*). |
| _atoma.txt | results for one atom at position number 'a' (keyword *Allsite*). |
| _atoma_scan.txt | *DAFS* scan results for the atom a (keyword *Allsite* and *DAFS*). |
| _tddft.txt | output with the TDDFT option (keyword *Tddft*). |
| _tddft_scan.txt | azimuthal scan in the TDDFT option (keyword *DAFS* and *Tddft*). |
| _tddft_conv.txt | convoluted spectra in TDDFT (keyword *Convolution* and *Tddft*). |

For the analysis of the spherical tensors (keyword *Spherical* and *Sphere_all*)

| | |
|---|---|
| _sph_atoma.txt | spherical tensors of the atom a. |
| _sph_atoma_int.txt | integral of the spherical tensors of the atom a. |
| _sph_signal_atoma_xan.txt | contribution of each atomic spherical tensor on the average xanes signal. |
| _sph_signal_atoma_poli.txt | contribution of each atomic spherical tensor on the xanes polarisation number i. |
| _sph_signal_atoma_rxsi.txt | contribution of each atomic spherical tensor on the *DAFS* reflection number i. |
| _sph_signal_xtal_xan.txt | contribution of the crystal spherical tensor on the average xanes signal. |
| _sph_signal_xtal_rxsi.txt | contribution of the crystal spherical tensor on the *DAFS* reflexion number i. |
| _sph_xtal.txt | spherical tensors of the crystal. |
| _sph_xtal_int.txt | integral of the spherical tensors of the crystal. |
| _sph_xtal_rxsi.txt | spherical tensor of the crystal for the *DAFS* reflexion number i. |

For the analysis of the cartesian tensors (keyword *cartesian*):

| | |
|---|---|
| _car_atoma.txt | cartesian tensors of the atom a. |
| _car_xtal.txt | cartesian tensors for the crystal |
| _car_xtal_rxsi.txt | cartesian tensors for the crystal for the *DAFS* reflection number i. |

# II- Basic keywords

### 1) Output file names

The different output files have names with the same root. The extensions automatically added depending on the chosen option. To define this root use :

***Filout*** → or "*File_out*"
*Sim/Cu/Cu_out* → Name of the output files (without extension)

The files can eventually be in a subdirectory.

### 2) Radius of the cluster

The final states are calculated inside a sphere, whose radius is defined with the keyword "*Radius*". Only the atoms inside this sphere are considered. By default, the sphere is centered on the absorbing atom.

***Radius*** → Mandatory keyword preceding the radius of the cluster.
*3.5* → value in Angstrom of the cluster radius.

For calculations on large energy range (typically used in EXAFS), it can be convenient using a decreasing radius when energy increases. For this purpose, one can set energy ranges with decreasing radius:

***Radius***
*8.3 100 7 250 5 500 4* → 8.3: radius up to 100 eV, 7 radius between 100 and 250 eV…
4 : radius beyond 500 eV

### 3) Cluster or crystal structure

Under "*Crystal*" or "*Molecule*" stand all the data describing respectively the unit cell or the molecule. If the calculation is done using the FLAPW output, this block is useless because the structure is red in one of the FLAPW output files. Under the keyword, come the mesh parameters (Å) and the angles (degrees). Then come all the atoms (and not only the non-equivalent ones, but when is specified the space group using the keyword "*Spgroup*"). By default and in the absence of the keyword "*Z_absorber*", the absorbing atom chemical specie corresponds to the first atom in the list.

Example 1: cfc copper crystal:

***Crystal*** → Crystal structure
*3.610 3.610 3.610 90. 90. 90.* → a, b, c, α, β, γ
*29 0.0 0.0 0.0* → Atomic number, position
*29 0.5 0.5 0.0*
*29 0.5 0.0 0.5*
*29 0.0 0.5 0.5*

Example 2: $FeO_6$ octahedron:

***Molecule***

```
      1.900  1.900  1.900   90.  90.  90.      → a, b, c, α, β, γ
 26   0.0    0.0    0.0                        → Atomic number, position
  8   1.0    0.0    0.0
  8  -1.0    0.0    0.0
  8   0.0    1.0    0.0
  8   0.0   -1.0    0.0
  8   0.0    0.0    1.0
  8   0.0    0.0   -1.0
```

The atomic structure can also be given in cylindrical or spherical coordinates. To use cylindrical coordinates, it is sufficient to give only two numbers under "*Molecule*". The program will understand they are *a* and *c* and that the positions of the atoms are given by r, ϕ and z. Thus to describe the same octahedron as previously:

***Molecule***

```
      1.900  1.900              → a, c
 26   0.0      0.0    0.0       → Atomic number, position (r, ϕ and z)
  8   1.0      0.0    0.0
  8   1.0    180.0    0.0
  8   1.0     90.0    0.0
  8   1.0    -90.0    0.0
  8   0.0      0.0    1.0
  8   0.0      0.0   -1.0
```

To use spherical coordinates, only one number (*a*) must be set after "*Molecule*". The position of the atoms is then given by r, θ, ϕ. For the same octahedron:

***Molecule***

```
      1.900                     → a
 26   0.0      0.0     0.0      → Atomic number, position (r, θ, ϕ)
  8   1.0     90.0     0.0
  8   1.0     90.0    90.0
  8   1.0     90.0   180.0
  8   1.0     90.0   270.0
  8   1.0      0.0     0.0
  8   1.0    180.0     0.0
```

When working with 2D film, one just has to replace « ***Crystal*** » by « ***Film*** ».

It is possible, in the non-magnetic case, to specify only the non-equivalent atoms. Then one has to give the space group under the keyword "*Spgroup*". The complete name, as in the international table, must be given. For example, for magnetite, one gets:

***Spgroup***

```
 Fd-3m:1            → it is also possible to write 227:1
```

***Crystal***
*8.3940 8.3940 8.3940 90.0 90.0 90.0*

*26 .6250 .6250 .6250 ! Fe 16d*
*26 .0000 .0000 .0000 ! Fe 8a*
*8 .3800 .3800 .3800 ! O 32e*

Note that for the calculation of the symmetry, it is important de define the atom position with a sufficient number of digits (say 10). For instance, for graphite one has to write:

***Spgroup***
*P63mc*

***Crystal***
*2.456 2.456 6.696 90. 90. 120. = a, b, c, alpha, beta, gamma*
*6 0.0 0.0 0.0*
*6 0.3333333333 0.6666666667 0.0*

Writing for example 0.3333 in the last line, would create false atoms. Program would stop immediately with an error message.

It is also possible to have an occupancy rate not full for the atoms. For this one must write the keyword "*Occupancy*" and a fifth column after the atom position containing this weight:

***Occupancy***

***Crystal*** → or ***Molecule*** or ***Film*** or ***Surface*** or ***Interface***
*2.456 2.456 6.696 90. 90. 120.*
*6 0.0 0.0 0.0 0.8* → *weight 80 %*
*6 0.25 0.25 0.0 1.* → *weight 100%*

During the building of the cluster, when 2 atoms are at the same position, only the one with the highest weight is kept. The final signal is taken with the given weights.

It is possible to use directly the structure given in *pdb* or *cif* files. These ones contain all the geometry, the space group… In this case use, one of the following way:

***Pdb_file*** (or ***Film_Pdb_file***, *when working with a 2D film*)
*File_name.pdb*

***Cif_file*** (or ***Film_Cif_file***, *when working with a 2D film*)
*File_name.cif*

It is also possible to describe a surface structure with a 2D periodicity using the keywords "*Surface*", "*Film*" or "*Bulk*". See these keywords presented in section "2D Diffraction". They can also be used for XANES.

### 4) Atomic electronic densities

An electronic configuration is used by default for all the atoms. It is possible to modify it by the use of the keywords "*Atom*" or "*Atom_conf*". Moreover under "*Crystal*" or "*Molecule*" one must not anymore put the atomic number, by the atom type number. For example in case of a $FeO_6$ octahedron with the Fe $3d^6 4s^2$ and O $2s^2 2p^4$ configuration:

***Atom*** → keyword preceding the atomic electronic densities
*26 2 3 2 6. 4 0 2.* → atomic number of the chemical specie of type 1, number
*8 2 2 0 2. 2 1 4.* of valence orbital and (n,l,pop) of each of these orbitals

***Molecule***
*1.900 1.900 1.900 90. 90. 90.* → a, b, c, α, β, γ
*1 0.0 0.0 0.0* → Atom type, position
*2 1.0 0.0 0.0*
*2 -1.0 0.0 0.0*
*2 0.0 1.0 0.0*
*2 0.0 -1.0 0.0*
*2 0.0 0.0 1.0*
*2 0.0 0.0 -1.0*

Important remark: contrary to what one can think, the formal charges attributed to the atoms in the ionic compounds are far from the true charge. Thus one has to perform exchange of charge between atoms with care and in a moderate way. A good technique is, for example for 3d elements, the good number of "d" electron, following the formal charge, but keeping the neutral atom, putting electrons in the large radius 4s or 4p orbitals.

Another possibility permits to keep the atomic number in front of each atom in the list after "*Crystal*" or "*Molecule*" and to give the electronic configuration only for some of them. For this use the keyword "*Atom_conf*" with just below
the number of atom with the following configuration (nbr)
the indexes of these atoms in the list under "Crystal" or "Molecule" ($ind_1$,$ind_2$…)
the number of atomics orbitals with a specific configuration (no)
the quantum numbers $n_i$ and $\ell_i$ and the occupancy, $p_i$, for each specific orbital i.

***Atom_conf***
*2 1 2 2 3 2 5. 4 1 1.* → nbr, $ind_1$, $ind_2$, no, $n_1$, $\ell_1$, $p_1$, $n_2$, $\ell_2$, $p_2$
*1 3 2 3 2 6. 4 1 0.* → another configuration for the third atom in the list below

*Crystal*
*4. 4. 4. 90 90 90*
*26 0. 0. 0.*
*26 0.5 0. 0.*
*26 0. 0.5 0.*
*26 0. 0. 0.5*

In the example above the atoms 1 and 2 of the list under "*Crystal*" have the configuration $3d^5 4p^1$. The atom 3 has the configuration $3d^6$. The remaining atoms have the default configuration.

When one wants to give a configuration for a doping element (see keyword "*Doping* "), one must write « 0 » for the atom index:

***Atom_conf***

*1 0 2 3 2 5. 4 1 1.* → nbr of atom (1), then index = 0

### 5) Absorbing atoms

All the atoms present in the structure participate to the absorption or scattering. By default, the calculated spectra correspond to the sum of the scattering or absorption produced by all the atoms of the same atomic number than the first one in the list under "*Crystal*" or "*Molecule*".

For clarity, or when the structure is given in a *cif* or *pdb* files, it can be convenient to define explicitly the atomic number by the use of the keyword "*Z_absorber*":

***Z_absorber***

*26* → all the atoms with Z = 26 are absorbing atoms

With the same keyword, it is also possible to calculate the spectra and their sum of atoms of different atomic number (but of the same edge, K…):

***Z_absorber***

*26 27* → all the atoms with Z = 26 or Z = 27 are absorbing atoms

In some cases, one can be interested by the calculation of the cross-section spectra of a single (or some) site in a structure containing several atoms of the same atomic number. For this purpose, instead of "Z_*Absorber*", use the keyword "*Absorber*", with below the index of the site, in the list under "*Crystal*" or "*Molecule*" :

***Absorber***

3 → absorbing atom number (here the 3$^{rd}$ in the list).

To have several non-equivalent sites, just write the different indexes:

***Absorber***

1 5 → atom numbers whom results will in output files "filename_1" and "file_name_5"

### 6) Energy range

The energy range E that one defines in the input is the energy of the photoelectron relative to the Fermi level.

By default the energy range is -5 to 60 eV by 0.5 eV step. One can change the range, the step or even have a variable step using:

***Range*** → keyword for the energy range
*1. 0.5 60.* → $E_{min}$, step, $E_{max}$

Other example with variable step:

***Range***
*1. 0.1 10. 0.5 20. 1. 60.00* → E min, step, E intermediate, step …

To get a continuously increasing step (k step constant) put:

***Rangel***
*1. 0.1 200.* → E min, step at the Fermi level, E max

By default, the output energy range is relatively to the Fermi level. If one wants that the output energy is the photon energy put the keyword:

***Energpho***

### 7) Multiple scattering mode

If one wants to calculate in the multiple scattering mode use the keyword:

***Green***

Then the potential is automatically a muffin-tin one. The mode is faster than the finite difference method, so one has to use it first.

### 8) Threshold type

By default the threshold is the K one. For other threshold put the keyword:

***Edge*** → keyword preceding the threshold type
*L1* → threshold (K, L1, L2, L3, M1 …)

It is possible in a single run to calculate 2 edges with the same initial (n,l), that is the edges L2 and L3 or M2 and M3 or M4 and M5 … For this write:

***Edge*** → keyword preceding the threshold type
*L23* → threshold L2 and L3, or M23, M45, N23, N45.

### 9) Multipolar expansion

By default only the transition electric dipolar component (E1E1) is calculated. This is modified by the keywords:

| | |
|---|---|
| ***Quadrupole*** | → quadrupolar calculation (E1E2 and E2E2) |
| ***Octupole*** | → octupolar calculation (E1E3 and E3E3) |
| ***Dipmag*** | → magnetic dipole calculation (E1M1) and (M1M1) |
| ***Dip_rel*** | → To take into account the spin-position transitions E1SP+SPSP |
| ***E1E2*** | → calculation of E1E2 |
| ***E1E3*** | → calculation of E1E3 |
| ***E2E2*** | → calculation of E2E2 |
| ***E3E3*** | → calculation of E3E3 |
| ***E1M1*** | → calculation of E1M1 |
| ***E1M2*** | → calculation of E1M2 |
| ***E2M2*** | → calculation of E2M2, the interférence transition channel E1-M2 |
| ***M1M1*** | → calculation of M1M1 |
| ***E1SP*** | → calculation of E1SP, interference E1-spin-position |
| ***SPSP*** | → calculation of SPSP spin-position – spin-position transition channel |
| ***No_E1E1*** | → No calculation of the dipolar-dipolar component (E1E1) |
| ***No_E2E2*** | → No calculation of the quadrupolar-quadrupolar component |
| ***No_E1E2*** | → No calculation of the dipolar-quadrupolar component |
| ***No_E1E3*** | → No calculation of the dipolar-octupolar component |

### 10) Polarization and dichroism

By default the calculation is performed along 1, 2 or 3 orthogonal polarizations in the dipole mode (and up to six in quadrupolar) depending of the symmetry, and only the average corresponding to a powder is given. To get the spectra for the different polarizations, use:

***Polarize***

To get linear or circular dichroism, one must specifies polarization and wave vector (in quadrupolar) orientations. These vectors must then be given in the non-orthogonal, but normalized crystal basis. For example if ($\alpha = \beta = \gamma = 90°$), for 3 polarizations in the (***a***,***b***) plane at 0, 30 and 45° from ***a*** :

***Polarize***
*1 0 0*
*0.866025404 0.5 0*
*0.707106781 0.707106781 0*

Note that the normalization is performed in the code thus for an orthogonal unit cell (*0.707106781, 0.707106781, 0*) is equivalent to (*1,1,0*). For a non-orthogonal unit cell, correct coordinates must be given. For example in the hexagonal case ($\gamma = 120°$), the coordinates of a vector in the (***a***,***b***) plane, at the angle $\delta$ from ***a*** are $\left(\cos\delta + \sin\delta/\sqrt{3}, \cos(120-\delta) + \sin(120-\delta)/\sqrt{3}, 0\right)$.

For a quadrupolar calculation, one has to specify the wave vector after the polarization:

***Polarize***

*1.0 1.0 0.0 0.0 0.0 1.0*
*1.0 -1.0 0.0 0.0 0.0 1.0*
*0.0 0.0 1.0 0.0 0.0 0.0*

Each line contains the polarization vector, the wave vector. If the wave vector is zero (or omitted) this case is calculated in the dipolar approximation.

It is possible to perform an average on different polarizations adding a new number at the end of the line is the corresponding weight. If at least two weights are non-zero, in the output, there will be a new column with the weighted average of the different polarization.

***Polarize***
*1.0 1.0 0.0 0.0 0.0 1.0 1.*
*1.0 -1.0 0.0 0.0 0.0 1.0 1.*
*0.0 0.0 1.0 0.0 0.0 0.0 0.*

If one wants to have a circular polarization, one just has to put this one to zero followed by the value of the wave vector:

***Polarize***
*0.0 0.0 0.0 0.0 0.0 1.0*

In the output one will have the calculation the sum right plus left polarizations, then the difference. In this example, in orthogonal coordinates, the calculation corresponds to the polarization x+iy and x-iy.

To calculate the transmission through a sample of a specific thickness and to check change in polarization to the sample, it is necessary to calculate an absorption σ-π matrix. This is done with the keyword:

***Mat_polar***
*0. 0. 1. 1. 0. 0.* → polarization σ and wave vector

See also the keywords: "*Sample_thickness*", "*Stokes*" and "*Stokes_name*" to define the sample thickness, incoming polarization matrix and analyzer angles.

## II-10) Doping

It is possible, more specifically for crystal to calculate the absorption of a doping element set in substitution in place of another one. For this one uses the keyword « Doping », followed by the atomic number of the doping element and the index of the atom to substitute in the list under « *Crystal* »:

***Doping***
27 1 → substitution by Cobalt of the atom in the first site.

The doping atom is supposed to be at low concentration, thus the cluster built around it, is the same than the one given by the crystal. Symmetries are kept.

### II-11) Getting back the electronic structure from a previous calculation

To save time, it is possible to get back the electronic structure of the absorbing atoms from a previous calculation (it is in the *fdmnes_out_bav.txt* type file) and to compute other polarizations, or ask for the density of state (keyword Density), or make a TDDFT calculation different or not done in the previous calculation, putting the key word "*Extract*" followed by the corresponding name:

***Extract***
*Sim/calcul_prec_bav.txt*

# III- Non resonant X-ray inelastics scattering (NRIXS)

The non-resonant inelastic scattering (NRIXS or X-Raman) is very close to XANES. The first order term is equivalent to the dipolar approximation in XANES. One can obtain a more precise simulation, depending on the momentum transfer $\boldsymbol{q}$, using the keyword:

***NRIXS***
*3. 5.5 9.* → the different values of the modulus of $\boldsymbol{q}$ in Å$^{-1}$

The corresponding output file will have the extension "_nrixs".

When one wants to simulate an experiment performed on a mono-crystal one must specify also the direction of vector $\boldsymbol{q}$. This is done replacing the keyword "*NRIX*S" by "*NRIXS_mono*", followed line by line by the modulus of q and the direction of $\boldsymbol{q}$ which is expressed in the unit cell basis. This direction does not need to be normalized:

***NRIXS_mono***
*3. 1. 0. 0.* → modulus of $\boldsymbol{q}$, then direction of $\boldsymbol{q}$
*3. 1. 1. 1.*
*3. 1. 2. 2.5*
*3.* → when the direction vector is not given (or equal to (0,0,0)) this
5. *1. 0. 0.* corresponds to the calculation of powder, like when using « NRIXS »
*5. 1. 1. 1.*

If one wants the results resolved in ℓ, the Bessel quantum number associated to the expansion of $exp(i\boldsymbol{q}.\boldsymbol{r})$ in the transition operator, write:

***All_nrixs***

By default the maximum value of ℓ is 2. To change it, write :

***Lmax_nrixs***
*3* → ℓ maximum value.

# IV- Anomalous or resonant diffraction

## 1) Reflections and polarization

In case of anomalous (or resonant) diffraction (DAFS, DANES, RXS, RXD) calculation, put the keyword "*DAFS*" (or "*RXS*") followed by the index of the beams to calculate. The orientation of the polarization and wave vector can be described by different way. When working in σ–σ, σ–π, π–π or π–s, or in circular polarization, put the number 1, 2, 3, 4 or 5 for the polarization respectively σ, π, circular right, circular left or linear along a general direction, in input then in output.

***DAFS***
```
 0 0 2  1  2  45.        → reflection indices, σ, π, azimuth
 0 0 2  1  1  45.        → reflection indices, σ, σ, azimuth
```

When the polarization is linear but not σ or π, but with an angle α such that α = 0° when it is σ and 90° when it is π, one must write:

***DAFS***
```
 0 0 2  1 0.  5   10.  45.      → reflection indices, σ, angle, rectilinear, angle, azimuth
 0 0 2  1 0.  5 -10  45.
```

Note that in this case, one must specify both incoming and outgoing polarization angles, even when one of them is σ, π or circular. When it is circular, the angle is not taken into account.

Let's take $\boldsymbol{a}$, $\boldsymbol{b}$ and $\boldsymbol{c}$, the unit cell vectors, $\boldsymbol{i_a} = \boldsymbol{a}/|\boldsymbol{a}|$, $\boldsymbol{i_c} = \boldsymbol{c}/|\boldsymbol{c}|$, and $\boldsymbol{Q}$ the normalized diffraction vector. We define the azimuthal angle, $\varphi$, using the base ($\boldsymbol{I},\boldsymbol{J},\boldsymbol{Q}$) such that:

$$\boldsymbol{I} = \boldsymbol{Q} \times \boldsymbol{i_c} \times \boldsymbol{Q}/|\boldsymbol{i_c} \times \boldsymbol{Q}|, \qquad \boldsymbol{J} = \boldsymbol{Q} \times \boldsymbol{I}$$

When $\boldsymbol{Q}$ is along $\boldsymbol{c}$, $\boldsymbol{i_c}$ is substituted by $\boldsymbol{i_a}$. One then gets, versus the Bragg angle $\theta_B$ the incoming and outgoing wave vectors:

$$\vec{k}_i = \cos\theta_B \cos\varphi \vec{I} - \cos\theta_B \sin\varphi \vec{J} - \sin\theta_B \vec{Q},$$

$$\vec{k}_s = \cos\theta_B \cos\varphi \vec{I} - \cos\theta_B \sin\varphi \vec{J} + \sin\theta_B \vec{Q}.$$

For the σ and π polarizations, one gets:

$$\vec{\varepsilon}_\sigma = \sin\varphi \vec{I} + \cos\varphi \vec{J}$$

$$\vec{\varepsilon}_{\pi i} = \vec{k}_i \times \vec{\varepsilon}_\sigma = \sin\theta_B \cos\varphi \vec{I} - \sin\theta_B \sin\varphi \vec{J} + \cos\theta_B \vec{Q}$$

$$\vec{\varepsilon}_{\pi s} = \vec{k}_s \times \vec{\varepsilon}_\sigma = -\sin\theta_B \cos\varphi \vec{I} + \sin\theta_B \sin\varphi \vec{J} + \cos\theta_B \vec{Q}$$

By this way, $\varphi$ increases when the sample is rotated trigonometrically. For example with an orthogonal unit cell: reflection (h,0,0), ($\boldsymbol{I},\boldsymbol{J},\boldsymbol{Q}$) =($\boldsymbol{i_c}$,$-\boldsymbol{i_b}$, $\boldsymbol{i_a}$)

1 1 0. → corresponds to σ-σ with polarization along $-\boldsymbol{i_b}$.
1 1 90. → corresponds to σ-σ with polarization along $\boldsymbol{i_c}$.

reflexion (0,0,ℓ), ($\boldsymbol{I},\boldsymbol{J},\boldsymbol{Q}$) =($\boldsymbol{i_a}$, $\boldsymbol{i_b}$, $\boldsymbol{i_c}$)

1 1 0. → corresponds to σ-σ with polarization along $\boldsymbol{i_b}$.
1 1 90. → corresponds to σ-σ with polarization along $\boldsymbol{i_a}$.

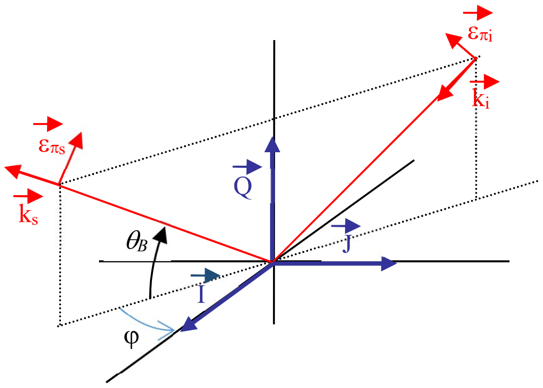


If one does not put the angle, this implies that one performs a $\varphi$ scan and all the amplitudes are calculated for all the angles by 2° step. There is then a supplementary output files with the extension "_scan.txt".

It is also possible to perform a 360° scan by 2° step for the tilinear incoming or outgoing polarizations. For this, one has to write 10 in place of the σ–π notification:

***DAFS***
0 0 2 *10 1 45.* → incoming polarization is scanned
0 0 2 *2 10 45.* → outgoing polarization is scanned

The first value (angle = 0°) corresponds to the σ polarization. 90° corresponds to π polarization.

It is possible to write the exact polarization directions. This can be useful for peculiar uses as in photoemission. In case of linear polarization write:

***DAFS***
*0 0 0* → Reflection index
*0. 0. 1. 0. 1. 0* → $\varepsilon_i$, $k_i$
*0. 0. 1. 0.7071 0.7071 0.* → $\varepsilon_s$, $k_s$

For circular polarization, it is complex:

***DAFS***
*0 0 0* → reflection index
*0.7071 0.7071 0. 0. 0. 0. 0. 1. 0.* → $\varepsilon_i$, $k_i$ ($\varepsilon_{xr}$ $\varepsilon_{xi}$ $\varepsilon_{yr}$ $\varepsilon_{yi}$ $\varepsilon_{zr}$ $\varepsilon_{zi}$ $k_{ix}$ $k_{iy}$ $k_{iz}$)
*0.7071 -0.7071 0. 0. 0. 0. 0. 1. 0.* → $\varepsilon_s$, $k_s$

When one does not want phase term between atoms, for example to simulate photoemission, just write 0 0 0 as reflection index.

Note that it is possible to choose any other origin for the azimuth, just using another vector than $\boldsymbol{i_c}$ to define the basis vector ***I*** and ***J***. For this just write:

***Zero_azim***
*0. 1. 1.* → vector in the direct crystal base

In some cases, as in biology, one has a very large number of diffracted beams. It is then simpler to specify the beams to calculate using experimental files. In this case one must give the corresponding file names containing the experimental spectra, the orientation matrix giving the incoming polarization versus the unit cell and for each file the rotation angle. One thus has to write:

***DAFS_exp***
*11.231547 3.519121 -71.823997* → matrix
*12.573563 -170.027649 -6.364535*
*-86.558281 -5.978752 -11.279972*
*0.* → angle for the first file
*MoFe/Refl_R5to2_1.txt* → name of the first file
*10.* → angle for the second file
*MoFe/Refl_R5to2_2.txt* → name of the second file …

Each experimental file contains some number of spectra in column along the format :
*-1 16 28 -1 20 18* → (h,k,l) for each reflections (integer)
*Energy D[-1,16,28] D[-1,20,18]* → one line of comment
*7080 -3.19 -7.96* → Energy, intensity for each reflections
*7100 9.75 16.65*

One must have at least one space between each number, but no tabulation or coma between the numbers. The number of digit or space between the numbers is free.

For a calculation with DAFS_exp, by default one calculates and compares to the data, the value : $\sqrt{I_{\sigma\sigma}(Q) + I_{\sigma\pi}(Q)} - \sqrt{I_{\sigma\sigma}(-Q) + I_{\sigma\pi}(-Q)}$. If one wants to calculate and compare other values related to the intensity, write "DAFS_exp_type " with below a number between 1 and 4:

***DAFS_exp_type***
*1* → $I_{\sigma\sigma}(Q) + I_{\sigma\pi}(Q)$
*2* → $\sqrt{I_{\sigma\sigma}(Q) + I_{\sigma\pi}(Q)}$
*3* → $I_{\sigma\sigma}(Q) + I_{\sigma\pi}(Q) - I_{\sigma\sigma}(-Q) - I_{\sigma\pi}(-Q)$
*4* → default case : $\sqrt{I_{\sigma\sigma}(Q) + I_{\sigma\pi}(Q)} - \sqrt{I_{\sigma\sigma}(-Q) + I_{\sigma\pi}(-Q)}$

It is now possible to calculate the self-absorption corresponding to the incoming and outgoing photon polarizations. For this put the keyword:

***Self_abs***

Then one gets in the output files, after each reflection two new columns containing the linear absorption coefficients, the unit being the $\mu m^{-1}$. They contain after the convolution also the absorption coming from the other atoms and the other edges. That is that the absorption before the edge is not zero. The new data allows the correction due to the self-absorption in order to compare with experimental spectra. The corrected spectra are, after convolution in columns following the non-corrected spectra. Their names begin with "Ic".

One can make a more sophisticated correction taking into account the birefringence. For this put the keyword:

***Full_self_abs***

It is then necessary to calculate all the reflections along the 4 polarizations σσ, σπ, πσ and ππ and in this order:

***DAFS***
*2 0 0 1 1 0.*
*2 0 0 1 2 0.*
*2 0 0 2 1 0.*
*2 0 0 2 2 0.*

In first approximation, this correction is equivalent to *Self_abs*. It allows the taking into account of the rotation of the polarization when the electromagnetic wave propagates into the media.

Note that from this, it is possible to calculate circular polarizations reflection intensity for any value of the Stokes parameters. This part is performed in the "convolution" step. See thus the corresponding paragraph.

By default the scan step is 2°. To modify it, use:

***Step_azim***
*0.5* → step in degrees of the azimuthal scan

**2) Non resonant magnetic scattering**

The non-resonant magnetic scattering is taken into account for the *DAFS*. This term can be decreased, or put to zero, using a multiplicative factor:

***No_res_mag***
*0.8* → factor

An additional factor can be applied to the contribution from the orbital moment. This factor corresponds to L/2S (and not L/S):

***No_res_mom***
*-0.3* → factor for the orbital moment

By default, this factor is calculated using the Hund rules and multiplied by 0.2.

### 3) Temperature

It is possible to take into account the thermic disorder using the Debye model. For this purpose use the keyword :

***Atom_B_iso***

After keyword "Crystal", one then must add a fifth column after the atom positions the Debye parameter ($8\pi^2\langle u^2\rangle$) in Å². If no number are given, the default zero value is given. When using also "*Occupancy*" keyword, the fifth and sixth columns contain the 2 corresponding parameters in the order given by the order of the keywords in the input file.

For the same purpose but using as Debye parameter $\langle u^2\rangle$ instead of $8\pi^2\langle u^2\rangle$ use:

***Atom_U_iso***

Debye disorder can also be anisotropic with keywords "*Atom_B_ani*" or "*Atom_U_ani*". Up to now only 3 values can be given for xx, yy et zz. When ony 2 values are given, the code sets $U_{xx} = U_{yy} \neq U_{zz}$. When a unique value is given $U_{xx} = U_{yy} = U_{zz}$. Columns adds as for "iso" When using also "Occupancy" it is better to have "*Atom_U_ani*" or "*Atom_B_ani*" after.

***Atom_U_ani***

### 4) Occupancy

In the same way, one can use an occupancy rate on each sites. For this purpose write:

***Occupancy***

Then after keyword "Crystal" in the fifth column, after the atom position, the occupancy rates, between 0 and 1 are given. When no number value, the default 1 value is used. When using also "*Atom_B_iso*" or "*Atom_U_iso*" (or *ani*), see just above for the order.

### 5) Common keywords with SRXRD

"*DAFS_2D*" allows the definition of reflections and polarizations using the operation modes corresponding to the diffractometer geometry with up to 6 circles. It is described in chapter SRXRD.

"*No_analyzer*" allows the calculation of reflections without analyzer. See also the chapter SRXRD.

# V- Potential, magnetism and calculation technique

### 1) Relativistic calculation

By default, simulations are non-relativistic, without spin-orbit and non-magnetic for the valence and conduction band states (but relativistic with spin-orbit for core states). For heavy atoms (say Z > 36), in the structure, including when they are not absorbing atoms, it is recommended to make a relativist calculation using the keyword:

***Relativism***

To be more precise, it can be useful, to start from the Dirac equation, which contains the spin-orbit interaction. Such a calculation, fully relativistic can be done with the keyword:

***Spinorbit***

Note that then the simulations are typically 4 to 8 times longer and need 2 times more memory space. Beside the relativistic aspect, when scattering or absorption depend on conduction band electron spin-orbit, this keyword is mandatory. A spin-polarized simulation can then be done using this keyword plus the keyword "*Atom_conf*" or "*Atom*" (see further on).

With the keyword "Spinorbit", simulation is automatically relativistic. For a non-relativistic simulation without spin-orbit use the keyword:

***Nonrelat***

### 2) Spin polarized calculation

To make a spin polarized calculation, one can use the "*Spinorbit*" keyword, see above, or more simply, when spin-orbit can be neglected (what can be the case of $L_{23}$ edges but never of the K edges) put the keyword:

***Magnetism***

If the polarized potential comes from LAPW, "*Spinorbit*" or "*Magnetism*" must be before "*Flapw*". When the calculation does not use the Wien-2k output, the use of the keywords "*Atom*" or "*Atom_conf*" is mandatory to specify the electronic configuration, different for the spin up and spin down parts. The orbital occupancy must be given for each spin in doubling the corresponding columns.

Example of metal fcc nickel:

***Magnetism***

***Atom***
*28 2  3 2 5. 4.  4 0 0.5 0.5*

***Crystal***
*3.52387 3.52387 3.52387 90. 90. 90.*
*1 0.0 0.0 0.0*
*1 0.5 0.5 0.0*
*1 0.5 0.0 0.5*
*1 0.0 0.5 0.5*

The configuration is $3d^9 4s^1$ with 5 electrons 3d up and 4 electrons 3d down, 0.5 electron 4s up and 0.5 electron 4s down.

In case of an antiferromagnetic structure, one must put a minus sign in front of the atom type number to specify the atom with the reverse spin. For example:

***Atom***
*23 2 3 2 2. 0. 4 1 0.5 0.5*
*8 0*

***Crystal***
*7.255 5.002 5.548 90.0 96.75 90.0*
*1 0.34380 0.00080 0.29910*
*1 0.65620 0.99920 0.70090*
*-1 0.84380 0.50080 0.79910*
*-1 0.15620 0.49920 0.20090*

*2 0.40700 0.84500 0.65200*
*2 0.09300 0.84500 0.34800*
*2 0.59300 0.15500 0.34800*
*2 0.90700 0.15500 0.65200*

Alternatively, it is possible to use the keyword « *Atom_conf* » which allows to specify the orbital occupancy of only some of the a atoms:

***Atom_conf***
*2 1 2 2 3 2 2. 0. 4 1 .5 .5* → nbr of atoms with the configuration, then index of these atoms
*2 3 4 2 3 2 0. 2. 4 1 .5 .5* → then nbr of orb, then n,l occ up, occ down for each orbital…

***Crystal***
*7.255 5.002 5.548 90.0 96.75 90.0 / a, b, c, alfa, beta, gamma*
*23 0.34380 0.00080 0.29910 V8*
*23 0.65620 0.99920 0.70090 V6*
*23 0.84380 0.50080 0.79910 V4*
*23 0.15620 0.49920 0.20090 V5*

*8 0.40700 0.84500 0.65200 O 1 8f*
*8 0.09300 0.84500 0.34800 O 2 8f*

### 3) Spin axis

By default the spin axe is along the *c* axis but in the trigonal unit mesh in which it is along the *c* axis of the associated hexagonal unit mesh. This axis can be orientated along any (but uniform) direction:

***Axe_spin***
*-0.08909 0. -0.15025* → in unit mesh

Another way to specify this axis is using the Euler angles. The orthogonal basis to do that is such that $\boldsymbol{z}$ is along $\boldsymbol{c}$, but for trigonal system in which $\boldsymbol{z}$ is along the hexagonal axis, $\boldsymbol{x}$ is along $\boldsymbol{b} \times \boldsymbol{c}$ and $\boldsymbol{y}$ is along $\boldsymbol{z} \times \boldsymbol{x}$. One then has to write:

***Ang_spin***
45. 90. 0. → rotation around $\boldsymbol{z}$, then around new $\boldsymbol{y}$, then around new $\boldsymbol{z}$. In this case spin axis is along (1,1,0) in the internal basis

Note that it is possible to define non collinear spin using atomic local basis. In this case one has to define the Euler local angles after the keyword "*crystal*" or "*molecule*", in the line just before the corresponding atoms. See "non-spherical atom". Example:

```
  Crystal
 7.7400  7.7400  3.8400  90.  90.  90.
 45. 90.                              ! spin axis along (1 1 0)
  1  0.0    0.0    0.0
  2  0.25   0.25   0.5
 135. 90.                             ! spin axis along (-1 1 0)
  1  0.5    0.0    0.0
  2  0.75   0.25   0.5
 315. 90.                             ! spin axis along (1 -1 0)
  1  0.0    0.5    0.0
  2  0.25   0.75   0.5
 225. 90.                             ! spin axis along (-1 -1 0)
  1  0.5    0.5    0.0
  2  0.75   0.75   0.5
```

### 4) Self-consistent calculations

By default a calculation cycle is performed over the occupied states to determine the Fermi energy. The XANES calculation corresponds to the cycle 2, but this one is done with the initial potential. To avoid this Fermi (non SCF) energy determination, use the keyword :

***No_Fermi***

To perform a self-consistent calculation, use the keyword:

***SCF***

The calculation is then really self-consistent. The potential is calculated again at each cycle. The final XANES calculation uses this SCF potential. By default the convolution uses the calculated Fermi energy as cutting energy.

When using SCF calculation, the cluster radius for the SCF is the same than the one for the XANES. For a single evaluation in one cycle of the Fermi energy the radius is equal to the

minimum between the XANES cluster radius and 3.5 A. In both cases, this radius can be change using the keyword :

***R_self***
*3.* → Value of the radius for the SCF and/or Fermi energy evaluation.

With SCF calculation, the maximum number of cycle is fixed to 100. To modify this number use the keyword:

***N_self***
*20*

The electronic density of the cycle n+1 is interpolated between the electronic density of the two previous cycles. The initial weight of the cycle is 0.1. This weight decreases or increases according to the quality of the convergence between 4 and 0.25 times the initial value. To change this initial weight use:

***P_self***
*0.05* → Initial weight.

The convergence is considered as realized when the variation of the total energy is less than $N_a$ x $\Delta E_{conv}$, in which $N_a$ is the number of atom and $\Delta E_{conv}$ a criteria fixed at 0.1 eV. To modify $\Delta E_{conv}$ put:

***Delta_E_conv***
*2.* → Value of $\Delta E_{conv}$.

It can happend, that when the weight increase up to 2 or 4 times the p_self, value, the calculation become very unstable (it can be seen on the orbital occupancy). On can then limit this increase by the parameter p_self_max:

***P_self_max***
*0.05* → value of the maximum weight. When used, the best is to put it equal to p_self.

The SCF calculation is done with a non-excited cluster. The excited potential of the absorbing atom is taken into account only in the K and $L_1$ edges in the XANES final calculation by difference. If one wants to perform a SCF calculation with an excited cluster, use the keyword:

***SCF_exc***

If one wants perform a simulation with SCF in excited state but without any screening, that is with a cluster charged +1, with SCF_EXC one has to use keyword "*Screening*" with the value 0 (see this keyword).

For a magnetic calculation, by default the spin polarization is kept fixed in amplitude. The total number of spin up and spin down electron is fixed along the self-consistent (for an anti-ferro, for the total on the atoms, the number of majoritarian spin and minoritarian spin electron are kept fixed). To have it free (equivalent to version before 7th of June 2012), use the keyword:

***SCF_mag_free***

It can happen that convergence is difficult to reach. A smoothing procedure is possible using:

***SCF_smooth***

### 5) Hubbard correction

To include a Hubbard correction applied to the "localized valence orbitals", use the keyword "*Hubbard_z*" with below the atomic number and the Hubbard parameter value (U-J) of the corresponding chemical specie.

***Hubbard_z***
*23 5.* → value in eV of the Hubbard parameter (U-J).
*26 4.5* → in case of 2 chemical species with such a correction

It is possible to have several chemical species with such a correction. It is also possible to have this correction with the keyword:

***Hubbard***
*5. 4.5* → value of the Hubbard parameters in the order of the atom types

The order of the atom type is the one of the chemical species appearing under the keyword "*crystal*" or "*molecule*" or under "*atom*" if used. Most often, it is convenient to perform also a self-consistent calculation. Note that in principal this correction can be applied only on insulating compounds.

### 6) TDDFT calculations

TDDFT calculations can be performed using the keyword:

***TDDFT***

Then a first mono-electronic (LSDA) calculation is performed, followed by the TDDFT cycle. Then a correction is performed making a mixing between the edges. This can be useful for the $L_{23}$ edges of the transition *3d* elements. Calculations use a local Kernel with its Coulomb and exchange-correlation parts. To have only the Coulomb part, use the keyword:

***RPA***

The TDDFT simulation contains a matrix inversion whom dimension is proportional to the number of spherical harmonics characterized by a l maximum equal to lmax. In optics lmax = 2 when Z < 21 and 3 in the other cases. To save time, especially in optics, it can be useful to limit it with the keyword:

Lmax_tddft
2 → maximum l value for this part of the calculation.

### 7) Conter-ion and Helmholtz layer

It is possible to add a potential simulating the effect of counter-ions in the case of a charged molecule imbedded in a solvent or to consider a non-ordered Helmholtz layer on top of an electrochemical interface (see the section on 2D diffraction). The corresponding potential is given by : $\Delta V = \frac{\sqrt{\pi}}{2} V_{helm}\,\mathrm{erf}\left(\frac{z-z_0}{\alpha\Delta_{helm}}\right) / \left(\frac{z-z_0}{\alpha\Delta_{helm}}\right)$ where z is the radial coordinate or the coordinate perpendicular to the surface, $z_0$ is the distance between counter_ion (or Helmholtz layer and most outer atom and $\Delta_{helm}$ is the half height width, with avec $\alpha$ = 0.285925223. When finite different method is used, this potential is also added on all the grid of point inside the interval. For the surfaces ans for $z < z_s$, where $z_s$ is the surface atom position, a correcting factor $(z - z_b)/(z_s - z_b)$ is applied to avoid a modification of the potential in the bulk part with $z_b$ the position of the top most bulk atom, the closest to the surface. All this is obtained with:

***Helmholtz***
*-10. 2. 8.* → $V_{helm}$, $\Delta_{helm}$, $P_{helm}$ (en eV et Å)

If , $\Delta_{helm}$ is omitted, its default value is : $\Delta_{helm}$= $2z_0$, which corresponds to an added potential on the most outer atom equal to $\frac{1}{2}V_{helm}$. Alternatively one can use another potential shape for $z \in [z_0, z_0 + P_{helm}]$ with $\Delta V = \frac{1}{2}V_{helm}\left(1 + \cos\left(\pi\frac{z-z_0}{\Delta_{hel}}\right)\right)$ and $\Delta V = 0$ outside. For is write:

***Helm_cos***
*-10. 2. 3.* → $V_{hel}$ , $z_0$, $\Delta_{helm}$ (en eV et Å)

The Helmholtz potential shape is not known inside the material. It can be suspected that a screening effect decreases it. For $z < z_s$, the formula with the erf function can be substituted par parabolic « *Helm_mix* » or linear « *Helm_lin* » functions., the second giving the strongest decrease. One gets them with the keywords :

***Helm_mix***
*-10. 2. 4.* → $V_{helm}$, $z_0$, $\Delta_{helm}$ (en eV et Å)

***Helm_lin***
*-10. 2. 4.* → $V_{helm}$, $z_0$, $\Delta_{helm}$ (en eV et Å)

For the Pt(111) surface case, these different options are illustrated in the figure below:

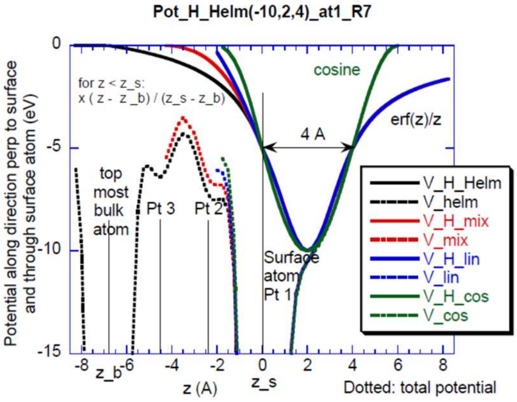

### 8) Exchange-correlation potential

By default the calculations are done using the real Hedin, Lundqvist and Von Barth potential. If one wants to use the Xalpha potential, one must introduce the keyword "*Xalpha*" followed by the value of the corresponding parameter:

***Xalpha***
*0.3333* → value of the Xalpha parameter

Remark : the value of the Xalpha parameter the closest to the other models is 2/3.
In the case of using the exchange-correlation potential coming from "*Flapw*", if one wants to keep this potential independent of the energy put the keyword "*Xalpha*" with any value beneath. The potential will not be Xalpha but the one calculated by *flapw*.

If one prefers to use the Perdew and Wang's potential or the one from Perdew, Burke et Ernzerhof PBE96, but keeping the energy dependency proposed by Hedin and Lundqvist, write respectively the keywords:

***Perdew***

or

***PBE96***

### 9) Screening

By default, the screening is one electron on the first non-full valence orbital of the absorber. If one wants to modify this value or the number of the orbital, use the keyword "*Screening*" followed by the quantum numbers of the valence orbital and the value in number of electron (better inferior to 1 !) of the screening. In this case the cluster is not anymore necessary neutral.

***Screening***
*3 2 0.2* → n, l, and screening on the valence orbital

One can omit the orbital quantum number. In this case the code, by default will choose the first non-full valence orbital and one just write:

***Screening***
*0.2* → Screening on the valence orbital
For magnetic calculations, screening is by default the half of the total screening electron on both spin states. Note that the occupancy rate cannot exceed 2 ℓ+1 for each spin state. If it is the case the exceeding amount is set on the other spin state. Whatever, one can impose a different screening, polarized in spin, just writting 2 numbers below "*Screening*", eventueally following n and ℓ :

***Screening***
*0.5 0.3* → screening 0.5 up, 0.3 down

# VI- Sophisticated keywords

### 1) Calculation of the Crystal Overlap Occupation Population (bounding)

The overlap between orbitals of neighboring atoms can be obtained using the keyword:

***COOP***
***1 2 4*** → indices of the selected atoms. If no index, only COOP with the absorbing atom.

The selected atoms are the ones around which one looks for the first neighbors up to a default distance equal to the sum of the atomic radii. It is between these couples of atom that are calculated the orbital overlaps. When no atoms are specified, the COOP are calculated only around the absorbing atoms.
These ones are given by the formula $n_{COOP} = S_{ab} \int \varphi_a(\boldsymbol{r})\varphi_b(\boldsymbol{r})d\boldsymbol{r}$, where $S_{ab}$ is the overlap integral and $a$ and $b$, 2 neighboring atoms. $n_{COOP}$, positive or negative gives the bounding and antibonding. Results are in files of the type « _coop_n_m » where n and m are the indices of the 2 neighboring atoms. The correspondence between these file names and the atom in the structure is given in the "bav" files looking for: "`File_coop_name`". The files contain versus energy, $n_{COOP}$ total, followed by $n_{COOP}(\ell_a,\ell_b)$, then $n_{COOP}(\ell_a,m_a,\ell_b,m_b)$.

We can modify the limit distance between 2 neighboring atoms to search for second neighbors for instance. This is done with:

***COOP_dist***
4 → Maximum distance in Angström

For calculation with spin-orbit, the harmonics basis for the COOP is such that the *z* axis is along the z axis of the cluster. For the calculations without spin-orbit, the basis is by default along the bond direction. To have a *z* axis, as in the spin-orbit case, along the *z* axis of all the calculation cluster, use the keyword:

***COOP_z_axis***

### 2) Uses of atomic electronic densities of Clementi and Roetti

By default the program calculate the atomic basis using a Hartree-Fock-Dirac procedure. If you prefer the Clementi and Roetti basis use the keyword:

***Clementi***

Note that the Clementi basis exists only for Z < 55. When using this basis, the self-consistent calculations are not anymore possible as well as the automatic evaluation of the Fermi level (The program takes automatically the option « *No_Fermi* »).

### 3) Non spherical atomic electronic densities

When calculating using the finite difference method, it is possible to define non-spherical atomic electronic densities. This is done with the keywords "*Atom_conf*" and "*Atom_nsph*". For each non-spherical atom one performs an expansion in spherical harmonics of these non-spherical orbitals. In practice, one writes an expansion in (l,m) of each of these orbitals with at the end the number of electron it contains. Optionally a rotation of the local atomic basis can be performed using the Euler angles (see keyword "*Ang_spin*", for the Euler angle definition). In this case, the Euler angles must be given in the line in front of the atom. Example:

***Atom_conf***
*1 1 1 3 2 3.*

***Atom_nsph***
*1 1 1* ! nbr of atoms, index of the atoms, nbr of orbitals
*0. 0. 0. 0. 0. 0. 1. 0. 0. 1.* → (l,m) in natural order, here (l=2,m=0) has 1 electron

***Molecule***
*1.9 1.9 1.9 90. 90. 90.* → a, b, c, α, β, γ
0. 45. 0. → 45° rotation around the Oy axis
*23 0.0 0.0 0.0*
*8 1.0 0.0 0.0*
*8 -1.0 0.0 0.0*
*8 0.0 1.0 0.0*
*8 0.0 -1.0 0.0*

Note that the code does not take into account the eventual decrease in symmetry induced by this configuration. One thus must take care.

When hybridizing odd and even orbital one can create an electric moment. This one can then be orientated in any direction thanks to the keyword "*E_moment*":

***Atom_nsph***
*1 1 1* ! nbr of atoms, index of the atoms, nbr of orbitals
*1. 0. 1. 0. 0. 0. 0. 0. 0. 1.* → hybridization s-$p_z$

***E_moment***
*1. 1. 0.* The resulting electric moment is rotated along the (1,1,0) direction

### 4) Mesh or molecule charge

The unit mesh must be neutral. A molecule is also often neutral. A test is performed in the program to verify this neutrality. If one wants to omit it, put:

***Chfree***

### 5) Orbital dilatation

It is possible to modify the valence orbitals defined above expanding or contracting them. This can be very useful for ionic material, for instance oxygen $2^-$, in which the atomic bases are calculated for neutral atoms. For this purpose introduce "*Dilatorb*" then for each orbital one wants to dilate, the atomic type (number in the list "*Atom*" or "Atom_conf"), the valence orbital number and the expansion coefficient. For the absorbing atom, add the same lines, but with "0" in first column, in order both excited and non-excited atoms are expanded:

***Dilatorb***
*3 2 0.3*
*0 2 0.3* → for the "excited absorbing atom

### 6) Reference of the photoelectron wave vector

By default the reference of the electronic wave vector (and so of the kinetic energy outside the sphere of calculation and the muffin-tin ground in case of muffin-tin calculation) is taken as the average of the potential between the absorbing atom and the first crown when calculating in green and at the outer sphere frontier in FDM. It is possible to impose this ground potential at the Fermi energy. The dependence versus the kinetic photoelectron energy is then added automatically to this term. Put then the keyword:

***V0imp***
*-11.5* → value of the ground potential at the Fermi energy.

### 7) Maximum potential

For a calculation performed on a molecule, the potential increases when going away from the atoms. When this molecule is not in the gaz phase, but in solution or in any relatively dense surrounding, this incease is artificial. In case of calculation under the finite difference mode, this reach to faulse bounded states very thin in energy. It can be usefull to give a maximum value to this potential to avois this phenomena with the use of the keyword "*Vmax*", followed by the value of this potential :

***Vmax***
*-6.*

### 8) Complex energy

It is possible to use a complex energy when working in the multiple scattering mode (green). For this, one has to specify the imaginary part (positive) of this energy in a table versus the photoelectron energy, under the keyword "*Eimag*":

***Eimag***
*0. 0.5*
*10. 0.7*

*30. 3.*
*50. 5.*
*100. 6.*

When a uniform broadening is sufficient, it is not necessary to specify the photoelectron energy:

***Eimag***
*0.1* → value of the uniform width (eV)

The use of a small width (0.1 eV) is sometimes useful for the calculations at low kinetic energy of the photoelectron because the localized level (3d or 4f) can be too thin in energy to be correctly evaluated. It is even more true for X-ray emission calculation (see keyword "*XES*"). If this broadening is small it has no effect on the forthcoming convolution to take into account the widths of the hole and final states.

### 9) Radius of the cluster for the superposition of the potential

The potential inside the sphere of calculation with a radius $R_s$ set under the keyword "*Radius*", is calculated by superposition. To avoid frontier problems, the atoms taken into account for the superposition are all the atoms inside the calculation sphere plus an outer shell. By default this outer shell is 2.5 Å thick, giving thus a new sphere with a $R_s$ + 2.5 radius. This new radius define also the cluster for the calculation of the punctual group. If one wants a bigger radius (useful for the oxides) put the keyword:

***Rpotmax***
*15.* → radius of the cluster for superposition in Angstrom

### 10) ℓ+1 approximation for the selection rule

If one wants to make the ℓ +1 approximation in which only the transition Δ ℓ = +1 is authorized (so one neglects Δ ℓ = -1, for example for the threshold $L_2$ and $L_3$, the transitions are only toward the *d* states), put the keyword:

***lplus1***

In the same way if you want only the ℓ -1 states put:

***lminus1***

### 11) Rydberg series

One can add an outer sphere having a -1/r potential to analyze Rydberg series. In this case put the keyword "*Rydberg*":

***Rydberg***

**12) Cluster origin**

By default, the cluster origin is set on the absorbing atom. If one wants that this origin is not changed from the input file put the keyword:

***Noncentre***

If one wants to impose a specific center put:

***Center***
*0 0.25 1* → coordinates in unit cell parameter unit of the center

The center can be automatically chosen at the center of the cluster when writing nothing after the keyword center:

***Center***

The center can be chosen at the center of the cluster considering only the chemical specie of the absorbing atoms with the keyword:

***Center_abs***

To have the center on an axis parallel to *c*, (usefull for surfaces) write the keyword:

***Center_s***
*0.124 0.5* → coordinates (x,y) in unit cell parameter unit of the axis

**13) Cut of the potential**

To get a cut of the potential in specific output files (*Filename_trace.txt*), along a line or a plan inside the FDM grid of points (does not work when using the multiple scattering theory (Green)), put:

***Trace***
*1 1. 0. 0. 0. 0. 0.* → n, a, b, c, p, q, r
n = 1, cut along a line with vector (a,b,c) crossing the point (p,q,r) in unit mesh parameter
n = 2, cut along the plane ax + by + cz = p
n = 3, gives all the points

***Trace***
*4* → To have all the points in the format "cube" : "*Filename_den.cube*" or "*Filename_pot.cube*" respectively for the density and the potential

## 14) Density of state

To get the partial projection of the density of states and their integral for the harmonics on the absorbing atom, put the keyword:

***Density***

Then one gets a new output file with the extension *_sd0.txt*. (if the absorbing atom is excited) or *_sd1.txt* in the other case. By default they are expressed using the cubic harmonics, that is: s, px, py, pz, $dx^2$-$y^2$, $dz^2$… For magnetic calculation, the expansion is split between the "up" and "dn" components.

One can prefer the standard tesseral (or real) harmonics. In that case for $\ell$=1 and $\ell$=2, the order of the harmonics is modified. From $\ell$=3 they are different and when they have no simple expression their corresponding column name is replaced by the conventional "($\ell$,*m*)". For this, with "*Density*", write also:

***Harm_tess***

It can be interesting, for example to get the orbital magnetic moment, to prefer output with the usual complex spherical harmonics. All column names are then of "($\ell$,*m*)" type. For this, use the keyword:

***Density_comp***

General remark on the values one gets: Usually for a transition metal, at the end of the *d* band, the integral must be close to 10 (because there are 10 *d* electrons!). At the end of the rather un-localized *s* and *p* bands, the integrals rarely reach 2 or 6, because the electrons are counted only inside the atomic sphere of radius $R_{mstd}$ (see in the bav file for its value). These orbitals having a rather big radius they are not all inside such atomic sphere. Calculation being performed in the continuum one also has to recall that *n* is not anymore a "good" quantum number. Consequently the integral continue to increase indefinitely with energy. At the end of a *d* (or *f*) band, rather localized, one nevertheless reaches more or less at 10 (or 14). In this case one can find the Fermi level energy just looking the energy in which the integral reach the supposed number of electron in the corresponding level of the atom. Sometimes the integral never reach 10 (or 14) or goes far higher. One of the reasons can be that the starting energy is too high, one thus loses the beginning of the band, and another reason can be the energy grid is not sufficiently thin. One has, in this case to take a path smaller (down to 0.01 eV or even less for 4*f* elements). In order to avoid such a thin grid, it is also possible de broaden a bit these localized states using the keyword "*Eimag*" (this works only in "Green" mode).

To get the projection on all the atoms in the cluster (or unit cell) put the keyword "*Density_all*" in place of "*Density*". Then one gets *n* new output files with the extensions *_sdi.txt*, "*i*" being the atom number. The correspondence between these files and the atoms of the material can be found in the "bav" file looking for : "`Output file name for the projected density of state`".

***Density_all***

## 15) Spherical tensors

To get the spherical tensors (in number of electron) put the keyword:

***Spherical***

One gets new files with the extension *_sph_atom1.txt* and *_sph_xtal.txt* for the atom and crystal. If one uses such file as input for the convolution process, with the keyword "*fprime*", one gets the f' and f'' of each tensor component of the atom. Another file *_sph_int_atom1.txt* contains the integral of the spherical tensors.

To get the contributions of the tensor components on each polarization and reflections, put in place of "*spherical*" the keyword:

***Sphere_all***

Then one gets a series of output files with the extensions *_sph_signal_xan.txt*, *_sph_signal_pol1.txt, _sph_signal_rxs1.txt* for the contributions on each polarization and reflections. The number after *pol* or *rxs* is the number of the polarization or reflection.

### 16) Cartesian tensors

To get the atomic cartesian tensors put the keyword:

***Cartesian***

One gets a new output file with the extension _car.txt. Then, one can use other keywords making that from this output file will be calculated f' and f'' for any tensor component. For this purpose put the keyword "*fprime*" in the input file.

### 17) Calculation area boundary

By default in FDM, the meshing is performed in a sphere extending up to the last atom inside the sphere of radius given under "*Radius*" plus the atomic radius (by default 0.65 Å) plus one inter-point distance (0.2 Å by default). In order to use a bigger sphere use:

***Overad***
*1.2* → distance over the last atom + its radius to take into account.

### 18) Displacement of the absorbing atom

To move the absorbing atom in reference to its position given under "*molecule*" or "*crystal*" use:

***Dpos***
*0.2 0.0 0.0* → displacement vector in Angstrom

**19) Energy shift of the spectra**

If one has gotten a reference for the initial orbital, it is possible to give it under the keyword "*Epsii*". This will produce a shift of the output spectra equal to the difference between this energy and the energy calculated in the program. It is safer to perform this operation with the shift parameters during the convolution step.

***Epsii***
*6253.1* → positive value in eV.

**20) Spectra by atom**

If one wants to get the signal coming from each atom one has to add the keyword:

***Allsite***

One then gets, at most of the usual output files, new output files of the type atom1.txt, atom2.txt, etc… The suffixes _atom1, _atom2 correspond to the number of the atom.

**21) Use of densities and potential coming from FLAPW**

If the potential is imported from a FLAPW calculation, one has to introduce the keyword "*flapw*". The keyword "*Crystal*" or "*Molecule*" becomes also unnecessary. Then must stand the names of the 5 FLAPW output files in case of spin un-polarized calculation and the 7 files in case of spin polarized calculation:

| | |
|---|---|
| ***Flapw*** | → names of the output FLAPW files |
| *tio2.struct* | structure and symmetry |
| *tio2.vcoul* | coulombian potential |
| *tio2.r2v* | exchange-correlation potential |
| *tio2.clmsum* | electronic density |
| *tio2.ti1s* | initial wave function |

In case of polarized calculation, the keyword "*magnetism*" must be before the keyword "*Flapw*"*:*

***Magnetism***

| | |
|---|---|
| ***Flapw*** | |
| *tio2.struct* | structure and symmetry |
| *tio2.vcoul* | coulombian potential |
| *tio2.r2v* | exchange-correlation potential spin up and spin down |
| *tio2.clmsum* | total electronic density |
| *tio2.clmup* | valence electron electronic density, spin up |
| *tio2.clmdn* | valence electron electronic density, spin down |
| *tio2.ti1s* | initial wave function |

The last file contains the wave function of the initial core orbital. If one is ok with the initial wave function calculated internally, one can avoid it. One must in this case substitute the keyword "*Flapw*" by "*Flapw_psi*".

If one wants to have an energy dependent exchange-correlation potential, one must add the keyword "*Hedin*".

By default, the absorbing atom is the first one in the "*struct*" file list. If one wants that it is the n$^{th}$, put the keyword:

***absorber***
*n*

For the actual version of Wien2k and magnetic calculations, the exchange-correlation potential for spin up and spin down is given in two different files. In this case one must use the keyword:

***Flapw_n***
*tio2.struct*
*tio2.vcoul*
*tio2_up.r2v* exchange-correlation spin up
*tio2_dn.r2v* exchange-correlation spin down
*tio2.clmsum*
*tio2.clmup*
*tio2.clmdn*
*tio2.ti1s*

when not using the wien2k core state use :

***Flapw_n_p***
*tio2.struct*
*tio2.vcoul*
*tio2_up.r2v*
*tio2_dn.r2v*
*tio2.clmsum*
*tio2.clmup*
*tio2.clmdn*

Note also that sometimes, it is necessary to increase the value of number of point in the radial mesh. In this case the value to put is given in an error message. It is possible using the keyword nrato.

***Nrato***
950 → new value given in the error message

### 22) Jump in the input file

It is possible to jump over a part of the information written in the input file with the keywords "*Jump*" and "*Endjump*". All what is between them is not red. For example:

***Jump***

*Quadrupole*
*Spinorbit*

***Endjump*** → the keywords "*Quadrupole*" and "*Spinorbit*" are not considered.

### 23) Comment

It is possible to introduce a line of comment which will be copied in the output files. For this purpose write:

***Comment***
*Iron K-edge in magnetite* → line of comment

### 24) Atomic spectra

To get in last column the atomic absorption spectra (without the neighbour atoms), put the keyword:
***Xan_atom***

### 25) Different absorbing atoms calculated in one run

The electronic structure is calculated in all the cluster of calculation and thus in all atoms in it. Consequently, it is in principal possible to get the absorption spectra of all the atoms in only one run. In principal, the absorbing atom is nevertheless "excited", thus becomes intrinsically different and one calculation must be performed for each absorbing atom. When neglecting this difference, it is possible to get all the absorption spectra of the all the atoms, equivalent and non-equivalent by symmetry operation, of the same chemical specie in only one run using the keyword:

***One_run***

For the $L_{23}$ and $M_{45}$ edges, the default calculation is such that the absorbing atom is not excited, thus the result in *one run* is in principal good. With "*One_run*" all the absorbing atoms are calculated, one thus must take care that they are all in the sphere of calculation. The use of keyword "*Center*" or "*Center_abs*" is sometimes helpful is this context.

For surface studies, with the use of "*Surface*" keyword, the calculation of all non-equivalent atom inside a layer is possible using the keyword:

***One_run_s***

The code calculates then automatically, for each layer of thickness by default 0.3 Å, the center of the spheres of calculation surrounding all the non-equivalent atoms of each layer. To change the default value, just add the corresponding number in the next line.

### 26) Increase the L maximum for the writing of the DOS and COOP

By default the pDOS and COOP are given in the output files up to :
L = 1 for $Z \leq 18$,
L = 2 for $19 \leq Z \leq 54$ et
L = 3 for $Z \geq 55$.
To increase these L maximum, use the keyword « *Lmax_DOSout* » :

***Lmax_DOSout***
*2* ! maximum value which replaces the default value when it is bigger.

### 27) Optimisation of self-consistency

The calculation is performed on a cluster and the atom symmetry corresponds thus to the punctual group of this cluster. It has nevertheless been observed that a faster self-consistency, by imposing at each SCF cycle, that the potential of all the atoms equivalent by symmetry of the space group, be identical. The chosen potential among all the equivalent atoms, is the one of the atom the closest to the cluster center, where it is supposed to be the best.

It nevertheless appears, more specifically for the oxides, that this way can leads to a beating phenomenon versus the cluster radius on the Fermi level value. This can be improved, keeping the potential identical only for the atoms equivalent by symmetry of the punctual group. This is done by the use of the keyword:

***Full_atom***

### 28) Core state energy

The core state energies (E_core or Epsii in the "bav" files) are used to perform the relative shift between the spectra of the different absorbing atoms. By default, these energies are the Kohn-Sham energies, that is $E = \langle\varphi|H|\varphi\rangle/\langle\varphi|\varphi\rangle$. In order these energies are the total energy of the corresponding state, use the keyword:

***Core_energy_tot***

Note that this energy is not the edge energy, because this one takes also into account the loss of total energy of all the other atom states during the photon absorption process.

### 29) Buiding cylinder clusters

For surfaces with charges, and specifically for surface diffraction, it can be helpful to build cylindrical cluster instead of spherical cluster between the absorbing atom and the surface. It remains half-spherical below the absorbing atom. The radius of the cylinder is the same than the one of the sphere and by default the absorber is on the axis of the cylinder. To have this structure only for the superposition of the atomic potentials use the keyword:

Cyl_pot

To have this structure also for the self-consistent calculation uses:

Cyl_SCF

The calculation of absorption and scattering remains done as usual in the sphere.

# VII- Technical keywords

Here stand the technical keywords necessary for specific tests and optimization of the code, for expert users.

## 1) Details on the calculations

To get details on the calculations done in every routine, put the keyword:

***Check***
*3 3 3 3 1 1 1 2 1 1*
*1 1 2*

Up to 30 numbers can be given. The correspondence between the number and the routine is given by :

| | | | |
|---|---|---|---|
| 1 : Lecture | 2 : Atom / Dirac | 3 : Symsite | 4 : Init-run |
| 5 : Agregat | 6 : Polarization | 7 : Atom_selec | 8: Etafin |
| 9 : Reseau | 10 : Potato | 11 : Orbval | 12 : Pot0 |
| 13 : Potentiel | 14: Enrgseuil | 15 : Ylm | 16 : Potex |
| 17 : | 18 : Sphere | 19 : Mat / MSM | 20 : Tenseur |
| 21 : Coabs | 22 : Tddft – sphere | 23 : Tddft – Kernel | 24 : Tddft - Chi_0 |
| 25 : Tddft – Chi | 26 : Hubbard | 27 : SCF | 28: State |
| 30 : Convolution | | | |

Values can go from 0 to 4 giving more and more for each routine. By default, there is 1 for all the routines. One can also write:

***check_all*** → equivalent to icheck = 3 for all the subroutines
***no_check*** → equivalent to icheck = 0 for all the subroutines
***check_conv*** → équivalent to icheck = 3 for the convolution
***check_pot*** → equivalent to icheck = 3 for all the subroutines concerning the potential
***check_mat*** → equivalent to icheck = 3 for the matrices MSM or FDM
***check_sph*** → equivalent to icheck = 3 for the subroutine "sphere"
***check_coabs*** → equivalent to icheck = 3 for the subroutine "coabs"
***check_tens*** → equivalent to icheck = 3 for the subroutine "tenseur"

***check_mpi*** → for parallel computation, gives also n_bav files containing the output of the not-central computer. "n" is the index of the computer. No index is the central computer.

## 2) Symmetry

The point symmetry is calculated automatically. Anyway the expert users can impose it by the keyword:

***Sym***

*2/m* → Schoenflies coefficient or international table of crystallography coefficient. Be careful that these symmetries are possible only for the mesh axis defined in the input.

### 3) Calculation basis

By default the connection to the continuum is performed in real base (neuman, bessel). To use a complex base (bessel, hankel) put:

***Basecomp***

### 4) Equivalent atoms

The program automatically calculates the total signal resulting from the equivalent atoms by symmetry. It is nevertheless possible, for the expert *fdmnes* user, to impose the symmetry relation between the atoms and thus to impose this summation. One must then use the keyword "*Symsite*". Following this keyword, stands the number of non-equivalent atoms (or number of group of atoms), then for each of them, the number of equivalent atoms followed the list of the relative symmetry to the first atom of the list and the atomic position in mesh unit. The symmetry are codified by number going from 1 to 64, see the list given bellow.

***Symsite***

```
 3               ! Number of non-equivalent atoms (or group of atoms)
 4               ! Number of equivalent atoms, group 1
  1 0.2500  0.2500  0.2500
 24  0.7500  0.7500  0.2500
 23  0.7500  0.2500  0.7500
 22  0.2500  0.7500  0.7500
 4                ! Number of equivalent atoms, group 2
  1 0.0000   0.0000    0.0000
 22  0.0000   0.5000    0.5000
 23  0.5000   0.0000    0.5000
 24  0.5000   0.5000    0.0000
 4               ! Number of equivalent atoms, group 3
  1 0.5000   0.5000    0.5000
 23  0.0000   0.5000    0.0000
 22  0.5000   0.0000    0.0000
 24  0.0000   0.0000    0.5000
```

The negative indices correspond to the same symmetry plus time reversal (in case of magnetism).

Symmetry code:

1: identity

2: rot 2π/3 around (1,1,1)
3: rot 4π/3 around (1,1,1)
4: rot 2π/3 around (1,-1,1)
5: rot 4π/3 around (1,-1,1)
6: rot 2π/3 around (-1,1,1)
7: rot 4π/3 around (-1,1,1)
8: rot 2π/3 around (1,1,-1)
9: rot 4π/3 around (1,1,-1)

10: rot 2π/2 around (1,1,0)
11: rot 2π/2 around (-1,1,0)
12: rot 2π/2 around (1,0,1)
13: rot 2π/2 around (-1,0,1)
14: rot 2π/2 around (0,1,1)
15: rot 2π/2 around (0,-1,1)

16: rot 2π/4 around 0x
17: rot 2π/4 around 0y
18: rot 2π/4 around 0z
19: rot -2π/4 around 0x
20: rot -2π/4 around 0y
21: rot -2π/4 around 0z

22: rot 2π/2 around 0x
23: rot 2π/2 around 0y
24: rot 2π/2 around 0z

25: inversion

26: rot 2π/4 around 0x and mirror
27: rot 2π/4 around 0y and mirror
28: rot 2π/4 around 0z and mirror
29: rot -2π/4 around 0x and mirror
30: rot -2π/4 around 0y and mirror
31: rot -2π/4 around 0z and mirror

32: rot 2π/3 around (1,1,1) and inversion
33: rot 4π/3 around (1,1,1) and inversion
34: rot 2π/3 around (1,-1,1) and inversion
35: rot 4π/3 around (1,-1,1) and inversion
36: rot 2π/3 around (-1,1,1) and inversion
37: rot 4π/3 around (-1,1,1) and inversion
38: rot 2π/3 around (1,1,-1) and inversion
39: rot 4π/3 around (1,1,-1) and inversion
40: plane perpendicular with 0x
41: plane perpendicular with 0y
42: plane perpendicular with 0z

43: diagonal plane y = z containing Ox
44: diagonal plane x = z containing Oy
45: diagonal plane x = y containing Oz
46: diagonal plane y = -z containing Ox
47: diagonal plane x = -z containing Oy
48: diagonal plane x = -y containing Oz

49: rot 2π/3 around 0z
50: rot 4π/3 around 0z
51: rot 2π/6 around 0z
52: rot 10π/6 around 0z
53: rot 2π/3 around 0z, negative axe
54: rot 4π/3 around 0z, negative axe
55: rot 2π/6 around 0z, negative axe
56: rot 10π/6 around 0z, negative axe

57: plane at 30° containing 0z
58: rot 2π/2 around axe at 30° perp. 0z
59: plane at 60° containing 0z
60: rot 2π/2 around axe at 60° perp. 0z
61: plane at 120° containing 0z
62: rot 2π/2 around axe at 120° perp. 0z
63: plane at 150° containing 0z
64: rot 2π/2 around axe at 150° perp. 0z

### 5) Bounded states

If one wants to make positive the kinetic energy for the calculation of bounded states (beneath the average potential), put the keyword "*E_out_min*" followed by the kinetic energy in eV:

***E_out_min***
 *0.05 0.5*

The first value is for the atomic scattering, the second one only used in FDM is for the cluster scattering. Default values are respectively 0.2 and 1 eV.

### 6) Inversion of the matrix in the multiple scattering mode

By default one inverts the matrix ($\kappa^{-1}$+i + G) in which κ corresponds to the Neuman-Bessel normalization. To avoid eventual convergence problems (not seen yet), one can prefer to calculate $(1 + G\tau)^{-1}\tau$. For this use the keyword:

***Normaltau***

### 7) Muffin-tin potential

If one wants to use the muffin-tin approximation in FDM put the keyword:

***Muffintin***

### 8) Non excited absorbing atom

At the K, L1, M1 edges the absorbing atoms is calculated excited that is with a hole in the core level and an extra electron in the first non-occupied valence state. To perform a calculation with a non-excited absorbing atom, that is with an absorbing atom without a hole in its core level and without screening put:

***Nonexc***

At the other edges, the absorbing atom is not excited. To make it excited put the keyword:

***Excited***

### 9) Modification of the grid of point parameters

By default the finite difference calculation is performed at order 4 with an inter point distance equal to 0.25 Å, the radius of the spherically symmetric area is around 0.65 Å (less for the light elements). To modify them write:

***Rmt*** → to modify the muffin-tin radius
*0.65*

***Iord*** → To modify the order of the Taylor expansion.
2

***Adimp*** → to modify the interpoint distance
*0.20*

It is possible to use inter-point distance, δ, decreasing with energy. This can be useful in EXAFS, because at high photoelectron kinetic energy, lower δ are necessary to get the same precision. For this, write :

***Adimp***
*0.24 100. 0.20 250. 0.16 400. 0.12 500. 0.08*

→ δ = 0.24 up to 100 eV, δ = 0.2 between 100 and 250 eV,… δ = 0.08 beyond 500 eV

Remark: when using different cluster radius, the energy values in which δ change, can be the same than for the radius (see "*Radius*" keyword).

### 10) Expansion in spherical harmonics

An expansion in spherical harmonics is performed in the atoms both in the multiple scattering mode and in the finite difference mode, but with a smaller radius. The maximum value of $\ell$ is obtained from the formula $kR = \sqrt{\ell_{max}(\ell_{max} - 1)}$, in which $k$ is the photo electron wave vector and R the muffin-tin radius (Rmtg in the bav file) in atomic units. By default, to this value one adds 1. To fix a value of $\ell_{max}$ independent of energy just write:

***Lmax*** → The $\ell_{max}$ value is fixed at 3
*3*

If one wants to keep the energy dependent formula but adding 1, 2 or more to this formula, put a negative sign in from of 1 :

***Lmax*** → The $\ell_{max}$ value is now given by the formula plus 2.
*-2*

When the energy is increasing, $\ell_{max}$ increases. By default the maximum value of $\ell_{max}$ is set at 2 for Z = 1, at 3 for Z = 2 then at 4 for Z ≤ 18, then at 5 for Z ≤ 36, then at 6 for Z ≤ 54, then at 7 for Z ≤ 86 and 8 for Z over. To avoid this limitation put the keyword:

***Lmaxfree***

When working using the finite difference mode, the connection to the outer sphere also needs an expansion in spherical waves. This one uses the same formula that for the atom but R is now the radius of the outer sphere. To modify this $\ell_{max}$ one proceeds exactly in the same way with positive value to fix an energy independent value, and negative value to keep the formula but adding to the formula. By default the additional value is 5. The key word is now "*Lmaxso*":

***Lmaxso***
*15*

It is possible to give a maximum value to the $\ell_{max}$ of the outer sphere writing:

***Lmaxso_max***
28 → maximum value

### 11) Muffin-tin radius

By default the muffin-tin radius (used in multiple scattering theory, for the electronic density calculation and for the SCF) is calculated to have a jump of potential the smallest as possible and closed between the atoms. One can modify it by the use of 3 different keywords.

If one wants that this radius is calculated using the Norman procedure introduce the keyword:

***Norman***

By default there is a 10% overlap between the spheres. If one wants modify this put the keyword "*Overlap*" followed by the value of the overlap:

***Overlap***
0.15 → 15% overlap

If one wants that the muffin-tin radius be determined by the value it gets when the atomic potential is equal to the interstitial potential, put the keyword "*Rmtv0*", followed by the value of this potential ( takes place the one define by the keyword "*V0imp*"):

***Rmtv0***
*-12.*

If one wants to impose the radius for each chemical species put "*Rmtg_Z*" followed by the atomic number and the radius in Angstrom for each of them, and line after line:

***Rmtg_Z***
*8 0.65* → Z = 8, Oxygen, radius = 0.65 A
*26 1.1* → Z = 26, Iron, radius = 1.1 A

### 12) Continuity of the potential at the muffin-tin radius

To limit potential jump between the interstitial area and the muffin-tin sphere, a linear interpolation on the potential is performed in the last 10% of the radius. For this put:

***Supermuf***

### 13) Atomic charge

The program gives in the output file the atomic charge integrated up to the muffin-tin radius. If one wants to get this value for a different radius put:

***Rcharge*** → corresponding keyword
*1.2 0.7* → radius value for each type of atom

To have this parameter define versus the atomic number, write:

***Rcharge_Z*** → corresponding keyword
*26 1.2* → atomic number and value of the radius for this chemical specie
*8 0.9*

In both cases it is not necessary to define all the radius. The other ones keep the value defined by the code.

The code also gives the charge integrated up to the ionic radius. These ones are tabulated in FDMNES versus the atomic number. To select another radius use:

***Rionic***
74 0.62 → atomic number and value of the radius for this chemical specie
8 1.50

### 14) Line length

When reading a file, the maximum number of columns of number is by default 10001. This number can be insufficient for example when making resonant diffraction. It can thus been increased using the keyword:

***Length_line***
20000 → maximum number of columns in one line

### 15) Resolution in initial state

The outputs are resolved in initial states only for magnetic calculation. For a non-magnetic calculation, it is possible to get anyway the output resolved be initial state using the keyword:

***Core_resolved***

It is then possible in the step convolution to select one of these states.

### 16) Memory save

For low symmetrical material, it can be useful to save some memory space. This makes an approximation (but a good one) on the potential calculation. For this use the following keyword:

***Memory_save***

### 17) Radius of the Dirac orbitals

By default, the atomic orbitals, are calculated solving a spherical Hartree-Fock-Dirac equation, up to a radius equal to 20 a.u. = 10.58 Å. In some very rare cases, this radius can be insufficient and an error message is shown, asking for an increased radius. This can be done with:

***Ray_max_dirac***
***12.*** → value of the radius of the new atomic orbitals in Å.

### 18) Header and lecture by Xas Viewer (Larix)

It is possible to have a header at the beginning of the output files giving the code release, the date and time of calculation. By this way the files are also more easily readable by Xas Viewer (Larix). It also gives for the file after convolution the convolution parameters and the edge energy.

***Header***

### 19) Output files format

The output files containing the spectra of x-ray absorption cross sections and diffracted intensities have column names containing parenthesis. Some soft-wares used for plotting misunderstand these parentheses. It is thus possible to substitute them by underlines "_" using the keyword:

***Python***

### 20) Cluster rotation

To optimize time calculation using the symmetries, fdmnes performs automatically cluster rotations. When demanded by keyword "Density", the density of states is given in this rotated basis. To avoid any rotation and keep the (eventually orthogonalized) crystal basis use the keyword:

***No_cluster_rot***

Note that the calculation can then be longer, because all the symmetry operations cannot be used.

# D- Convolution

## I- Introduction

The f*dmnes* program allows performing:

1) the convolution by a lorentzian of absorption spectra eliminating the occupied states. For the anomalous diffraction, it makes the integration over energy of the unoccupied states then calculates the intensity of the diffracted peaks.

2) a weighted summation over different outputs of the *fdmnes* program. This summation can include a relative shift between the spectra.

This step of the calculation can be done together with the previous one, just adding the corresponding keywords. It can also be done independently, writing another input file contains only the keywords related to the convolution. Here comes an example:

***Calculation*** → To give the file name resulting from the previous step.
*g_rs43* → name of the file (extension ".txt" is not necessary)

***Conv_out*** → To give the file name of the convoluted spectra
*g_rs43_conv* → name of the file

***Convolution*** → keyword to specify an "arctangent" shape for the broadening"

***E_cut*** → keyword to specify a cutting energy
*-1.5*

## II- Keywords for the convolution

### 1) File names

The input files for the convolution step are the output files of the previous step whose names are defined under "*Filout*". When the convolution is performed together with the main calculation, it is not necessary to specify again this name. In the other cases one has to introduce it or them with the keyword "*Calculation*":

***Calculation*** → To give the file names resulting from the previous step.
*g_rs43* → name of the file

When the main calculation contains several non-equivalent absorbing sites and has thus produced several output files: "File_name_1", "File_name_2", …, it is not anymore necessary to specify all of the but only "File_name".

It is not true in the following example where we want to have 2 different relative shifts or weights between the spectra different:

***Calculation*** → To give the file names resulting from the previous step.
*Fe_rs64_01* → name of the file 1
*1.0 0.2* → weight, shift
*Fe_rs64_02* → name of the file 2
*1.0 -0.2* → weight, shift

The weight is applied on the XANES and the anomalous scattered amplitudes. For the *DAFS* calculations, the output files, before convolution contain in second line the Thomson factors plus the anomalous part of the non-resonant atoms. By this way it is possible to take into account an average configuration for substitutions of atoms of different chemical specie on the same sites.

The name of the convolution output file is by default the input file name with the added suffix "*_conv.txt*". Anyway it is possible to impose another name with the keyword:

***Conv_out***
*Fe_rs64_sum_conv* → name of the convoluted spectra file

To specify a working directory put the keyword "*Directory*" followed by the directory name with at the end the separator "/":

***Directory***
*C:/Documents and Settings/joly/Mes documents/xanes/xanout/v2o3/*

When there are more than one absorbing sites, it is possible to have not only the total convoluted file but also the individual convolutions. For this write the keyword:

***All_conv***

Before the edge, the absorption is zero. It is possible to take into account the background coming from the edges of lower energy from all the chemical elements in the material. For this use the keyword:

***Abs_before***

### 2) Fermi level

The zero of the energy is set at the Fermi level. The states beneath the Fermi level are occupied. At the beginning of the convolution step, the cross section is thus set to zero, for negative energies. It is possible to change this cutting energy with the keyword:

***E_cut*** → keyword to specify the new cutting energy ("*EFermi*" keyword in previous
*-1.5* versions)

The cutting value is applied without additional shift because in principle this last is used to simulate the core level shift. Anyway it is possible to apply it added with the E_cut value by the keyword:

***Dec***

It is also possible to have different values of the cutting energy for the different files. This is written in third column after each file name:

***Calculation*** → To give the file names resulting from the previous step.
*Fe_rs64_01* → name of the file 1
*1.0 0.2 -2.* → weight, shift, cutting energy
*Fe_rs64_02* → name of the file 2
*1.0 -0.2 -1.9* → weight, shift, cutting energy

The cutting energy written under "*E_cut*" when specified, is then not considered.

### 3) Convolution width

The convolution to apply depends on the core level width ($\Gamma_{Hole}$) and the final state width. There are different ways to calculate the resulting energy dependent broadening. The first one uses an arctangent formula:

$$\Gamma = \Gamma_{Hole} + \Gamma_m \left( \frac{1}{2} + \frac{1}{\pi} \arctan\left( \frac{\pi}{3} \frac{\Gamma_m}{E_{Larg}} \left( e - \frac{1}{e^2} \right) \right) \right)$$

with: $e = \frac{E - E_F}{E_{cent}}$ in which $\Gamma_m$, $E_{cent}$ and $E_{larg}$ are respectively the maximum width (at high energy) of the final state, the center and the width of the arctangent function. The depth at the center of the arctangent is $\Gamma_m/E_{larg}$. A typical curve is given bellow:

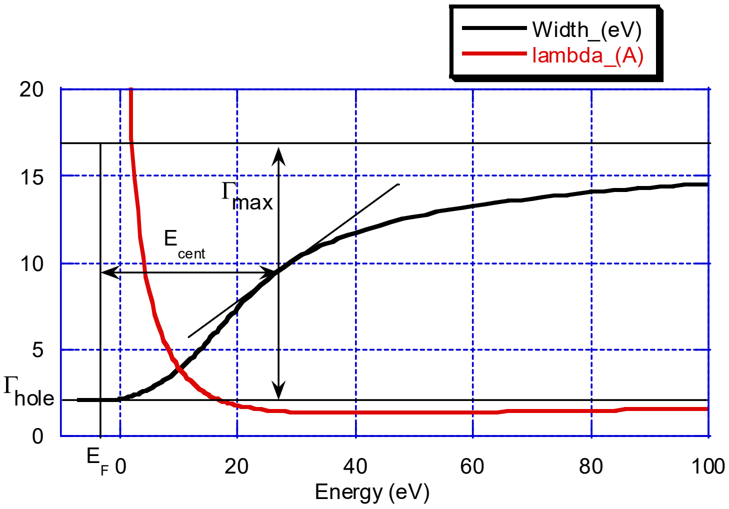


Such a convolution is introduced with the keyword:

***Convolution***

The $\Gamma_{Hole}$ values come by default from the tables of M. O. Krauze and J. H. Oliver, (*J. Phys. Chem. Ref. Data* **8**, 329 (1979)). Nevertheless, it is possible to modify them, especially in case of simulation of high energy resolution experiments, with the keyword:

***Gamma_hole***
*2.05* → Core level width ($\Gamma_{Hole}$) in eV.

To have different values of $\Gamma_{Hole}$ for a double edge like $L_{23}$, just put 2 numbers:

***Gamma_hole***
*1.5 1.* → First edge $L_2$ then $L_3$

Default values for $E_{larg}$ , $E_{cent}$ and $\Gamma_m$ are respectively: 30, 30 and 15 eV. It is possible to modify them with the keywords:

***Ecent***
*30.* → $E_{cent}$

***Elarg***
*30.* → $E_{larg}$

***Gamma_max***
*20.* → $\Gamma_m$

In the convolution, along the integration it is the width of the running energy which is taken. It is possible to use the width of the final state energy corresponding to the energy of the elastic photon. One then makes the integration with a constant width. This procedure improves the agreement with experiment especially in the pre-edge range in which the other procedure increases the background. To impose nevertheless a variable width along the integration in XANES uses the keyword :

***Gamma_var***

It is also possible to use the Seah-Dench formula for the calculation of the broadening. In this case one gets:

$$\Gamma = \frac{A\Gamma_m E_p}{\Gamma_m + AE_p} + \Gamma_{Hole}, \qquad \lambda = \frac{1}{A\sqrt{E_p}} + \frac{\sqrt{E_p}}{\Gamma_m}, \qquad \text{with: } E_p = E - E_F.$$

This is performed with the keyword:

***Seah***
*1. 20.* → A,$\Gamma_m$

One can also use a simple table with the keyword "*Table*":

***Table***
*0.0 1.* → Energy ($E-E_F$) and broadening $\Gamma(E-E_F)$
*10.0 1.5*
*20.0 2.*
*30.0 3.5*
*100.0 5.*

When the keyword "*E_cut*" is used without the keywords "*Convolution*" or "*Seah*", the width of the convolution is constant and equal to the width of the core hole. In all cases, the curve shape is written in the talkative "bav" file when the convolution is done in the same run than the main calculation. When convolution is done alone, to get this curve write the keyword:

***Check_conv***

To simulate the experimental resolution, it is also possible to convolute by a Gaussian. This convolution is performed after the lorentzian. For this write:

***Gaussian*** → keyword
*1.* → width of the gaussian

#### 4) Starting energy

To get output spectra starting at a lower energy put:

***Estart***
*-8.* → Value of the starting energy (eV)

#### 5) X-ray emission spectroscopy

With the keyword "*XES*", it is the emission spectrum which is calculated, and more accurately the valence to core decays. In such calculation the cutting is simply above the Fermi level and not beneath it as in XANES. The convolution broadening is the independent of energy and is fixed by default to the hole width. This with can nevertheless been modified by the use of the keyword "*Gamma_hole*". Note that would have interest having previously performed a calculation in "green" mode with a minimum energy width (keyword "*Eimag*") at least 0.1 eV, all this within an energy range convenient for emission:

***XES***

#### 6) Damping due to multi-electronic effects

In a simple approach, the multi-electronic phenomena can be seen as a simple damping in the absorption cross section and in the anomalous scattering amplitude in diffraction. This damping not taken into account, by default, can be introduced using the keyword "*S0_2*". It is typically around 0.8.

***S0_2***
*0.8* → value of the damping

When setting S0_2 = 0, in diffraction, one keeps only the non-resonant term.

## 7) Selection of the transition from a peculiar initial state

The fdmnes output files, before convolution, contain the spectra by transition from all initial states when the calculations are magnetic and by threshold in the other case. It is possible to perform the convolution for only one, or some, of these components (avoiding thus their total summation) with the keyword "*Selec_core*" with below the selected state. The order of these states goes from $j_z$ = -j to j ; for example at a $L_3$ edge: -3/2, -1/2, 1/2, 3/2. For a $L_{23}$ edge, $L_2$ is before $L_3$. For example, applied to a K edge, the 1s state with $j_z$ = -1/2 is selected. It is purely spin down and after transition probes the valence down states:

***Selec_core***
*1 2* → the transitions from the first and second core state are selected.

## 8) Sample thickness

When the "*Mat_polar*" keyword in the main calculation has been used, in order to calculate the transmission through a sample, the sample thickness in µm must be given. This is done with the keyword:

***Sample_thickness***
*100.* → Sample thickness in µm

See also the "*Stokes*" and "*Stokes_name*" keyword, below, which also apply in absorption when the "*Mat_polar*" keyword have been used.

## 8) Thermal disorder

It is possible to take into account the thermic disorder using the Debye model. For this purpose use the keyword:

***Abs_B_iso***
*0.72* → Debye parameter ($8\pi^2\langle u^2\rangle$) in Å²

For the same purpose but using as Debye parameter $\langle u^2\rangle$ instead of $8\pi^2\langle u^2\rangle$ use:

***Abs_U_iso***
*0.01* → Debye parameter ($\langle u^2\rangle$) in Å²

When these parameters are already given in the input file of the main calculation (using "*Atom_U_iso*" or "*Atom_B_iso*"), they are replaced for this part of the calculation by these new ones.

To have a uniform energy step after the convolution, use the keyword "E_step_conv". By default, the step is then the one of the beginning of the energy scale of the main calculation. To have a specific one, just write its value below the keyword:
***E_step_conv***
0.1 → if présent, energy step in eV. By default it is the one of the beginning of the main calculation

# III- Keywords specific for resonant diffraction

## 1) Azimuthal scan

If there is an azimuthal scan in anomalous diffraction, one has also to give the corresponding input file names (type *_scan.txt*) and the new output file. This is set after the keyword "*scan_file*":

***Scan_file***
*Fe_rs64_01_scan.txt* → name of the input dafs file 1
*Fe_rs64_02_scan.txt* → name of the input dafs file 2

***Scan_conv***
*Fe_rs64_scan_conv.txt* → name of the new output file with the convoluted scan

When the convolution file is included in the "main" calculation file, it is not necessary to specify the scan file names because they are known by the code. In this case, if one wants to convolute also these files just uses the "*Scan*" keyword:

***Scan***

## 2) Thomson

Thomson term is calculated by default. It is possible to modify it using the following keywords.

To eliminate the Thomson factor $f_0$ (as for a forbidden beam) put:

***Forbidden***

It can be useful to impose specific value for each *DAFS* reflections for the non-resonant (Thomson) structure factors and the resonant one of the other atoms. That is: $\sum_a p_a(f_{0a} + f'_{0a} + if''_{0a})exp(i\boldsymbol{Q}.\boldsymbol{R}_a)$, with $f'_{0a}$ and $f''_{0a}$ considered only for the other chemical species and in which $p_a$is the site $a$ occupancy rate. This is the case for example for occupancy rate non equal to one. These complex terms taken as a constant in all the energy range can be introduced through the keyword:

***Thomson***
*1.1670478E+02 1.0583769E+02 1.1477827E+02 -8.2670689E+01*

In the second line stand these terms, here for 2 reflections. They replace the values calculated by the program and placed in the second line of the output file.

## 3) Getting f' and f"

To get in the output file, the f' and f" values, put the keyword:

***Fprime***

In the output file for each reflection, there are three columns, f', f'' then intensity. The intensity is for the mesh, f' and f'' are for one atom of the mesh. f' contains also $f_0$. To get only f', put also the keyword "*forbidden*".

It is also possible to get the atomic (but in the solid) f' and f'' values without *DAFS* simulations. For this the best is to run the main simulation with the keyword "*spherical*". In a second step one convolute with the keyword fprime the corresponding output file "…_sph_atom1.txt"

To get the isolated atomic f' and f'':

***Fprime_atom***

The values of the atomic f' and f'' are then in the talkative "bav" file, eventually created at this time when the convolution is done in an independent job.

**4) Corrections**

It is possible to make a double absorption correction, corresponding to a measurement done at the maximum of the « rocking curve » and thus not integrated over it. For this write the keyword:

***Double_cor***

This correction is effective only with *self_abs* or *Full_self_abs* in the main calculation step. One gets then in the output file after convolution a new column with 'Id' which contains the intensity doubly corrected.

Another correction is possible to take into account a dead layer, that is a disordered layer at the surface, not participating to the diffraction, but damping the signal in the incoming and in the outgoing. This is done with the keyword:

***Dead_layer***
*150* → thickness in Angstrom of the dead layer.

This correction is also done during the convolution part of the calculation.

The absorption correction depends on the incidence and exit angles with the surface. Be default one uses the (rather good) approximation of taking the average angles. To use the exact angles, one must define the surface plane. This is done with :

***Surface_plane***
*1 2 0* → indices of the surface plane

# III- Getting other polarization conditions

One can be interested by material with birefringence or rotating power properties with effect in resonant diffraction or in absorption measured in transmission mode and with an analyser not necessarily parallel to the incoming polarization. One can make this choice on the analyser condition at the convolution step calculation when in the previous step, the main calculation had been performed with all σσ, σπ, πσ and ππ polarization conditions. In absorption calculation this is done when using the keyword "*Mat_polar*".

In that case, to get the intensities (only for diffraction) corresponding to incoming polarization right and left, add the keyword:

***Circular***

It is also possible to get the intensity corresponding to any Stokes parameters (S1= +/-1 sigma and pi, S2 diagonal S3 = +1(-1) circular right (left)) or with or without analyzer, this one with any angle. For example without analyzer one writes:

***Stokes***
*0 0 1* → S1, S2, S3
*0 0 -1*
*0 0.31 0.95*
*0 0.31 -0.95*

***Stokes***
*0 0 1 30. 50.* → S1, S2, S3, rotation and Bragg analyzer angles

If the Bragg angle analyzer is not specify, it is supposed perfect, that is 45°:

***Stokes***
*0 0 1 30* → S1, S2, S3, rotation analyzer angle (0 = σ, 90 = π)

Note that when using the "*check_conv*" keyword, the Stokes parameters of the transmitted beam are printed in the "File_name_bav.txt" file.

It is possible then to specify column name in the output file to make simplest the column identification, writing:

***Stokes_name***
no_name → default name
no_name
+00+31+95 → name appearing in the first line, in the corresponding column
+00+31-95

In absorption one can check the different contributions on the transmitted signal coming from birefringence or from dichroism alone. For this purpose, write:

***Check_biref***

Then for each polarization condition one gets 3 new columns in the output file with in their name “nB” for “no birefringence”, “nD” for “no dichroism” and “nO” for no off-diagonal components.

To have the weighted average between 2 sets of spectra $(J_a, J_b)$, giving $(I_a, I_b)$, using the formulas: $I_a = wJ_a + (1-w)J_b$ and $I_b = (1-w)J_a + wJ_b$, use the keyword:

***Weight_co***
0.7 → weight
1 2  5 6  8 9 → couple of spectra (column index in the output file, without energy) to average

When using the default value (0.5) for the average, it is not necessary to specify its value:

***Weight_co***
1 2  5 6  8 9

## IV- Independent convolution by a gaussian

Independently from any calculation the *fdmnes* code makes possible to perform a simple convolution by a gaussian of a table of data. This table must have in first column the abscissa then a set of column with the ordinates. All the columns must have the same number of elements. These data can be of any type, for example experimental spectra. Any number of text lines can be before the data.

The input file asking for such a convolution is of the type:

***File_in*** → keyword demanding the file name containing the data to convolute
*File_name_data* → name of the file

***Conv_gaus*** → keyword demanding the width of the gaussian
*2.5* → width in the same unit that the abscissa in the first column of the data file

***File_out*** → keyword demanding the output file name of the convoluted data
*File_name_out_conv* → name of the file

***End***

# E- Parameter optimization

It is possible to compare the calculated spectra to the experimental ones with the help of metric distances and R factor. The metric distance to compare the individual theoretical, $I_{i,th}(E)$, and experimental, $I_{i,exp}(E)$, spectra is given by:

$$D_1^i = \frac{1}{2}\int \left| \frac{1}{c_{i,th}} I_{i,th}(E) - \frac{1}{c_{i,exp}} I_{i,exp}(E) \right| dE$$

with the normalization factors: $c_i = \int_{E_{min}}^{E_{max}} I_i(E) dE$

The R factor is a conventional one (see E. Zanazzi and F. Jona, Surf. Sci. 62, 61 (1977)) given by:

$$R_X^i = \frac{\sum_j \left| c_i I_{i,th}(E_j) - I_{i,exp}(E_j) \right|^2}{\sum_j \left| I_{i,exp}(E_j) \right|^2}$$

With the normalization factor, $c_i$, such that: $\frac{\partial R_X^i}{\partial c_i} = 0$

Then for n different spectra, one gets the total metric distance or R factor by:

$$D_1 = \sum_{i=1,n} p_i D_1^i \qquad \text{and} \qquad R_X = \sum_{i=1,n} p_i R_X^i$$

In which $p_i$ is the relative weight for the spectra $"i"$ given by $p_i = \frac{E_{max}^i - E_{min}^i}{\sum_{k=1,n} E_{max}^k - E_{min}^k}$

It is possible to vary some parameters in order to optimize the agreement between calculation and experiment. Then results are given for a multi-dimensional grid of parameters. The values of the metric distances are given in a special output file. By default, for a complete calculation, the file name is the *fdmnes* conventional output file name with the suffix *_fit.txt*. For a calculation starting calculating the convoluted spectra, the default output file name is *fdmfit_out.txt*. This name can be modified using a specific keyword.

To do this task different keyword must be added in the main input file:

***Experiment*** → Keyword preceding the file names containing the experimental
*Nom_exp* spectra.

***Gen_shift*** → Minimum and maximum energy shift between calculation and
*7108 7114 21* experiment and number of value to test

***Metric_out*** → Keyword preceding the output file name containing the metric
*File_name_fit.txt* distances

***Parameter*** → Keyword preceding any group of correlated parameters
***Par_Gamma_max*** → Keyword to specify the convolution broadening $\Gamma_m$ as a parameter
*15. 25. 5* → First and last values and number of values
***Parameter***
***Par_Posx***
*-0.05 0.05 3*

***Par_Posy***
*-0.05 0.05 3*

In the previous example the parameters *Posx* and *Posy* of the atom 1 are completely correlated. Thus the atom is displaced along the diagonal.

The parameters can be fitted are:

For the convolution:

*Par_ecent* → Central energy for the arctangent
*Par_elarg* → Energy width for the arctangent
*Par_efermi* → Fermi (or cutting) energy
*Par_gamma_hole* → Hole width
*Par_gamma_max* → Maximum width for the final states
*Par_gauss* → Gaussian width (or resolution)
*Par_shift* → Energy shift
*Par_weight* → weight
*Par_weight_co* → weight for the average weight between 2 sets of calculated spectra
*Par_aseah* → First parameter of the Seah-Dench formula
*Par_abs_u_iso* → Mean square displacement of the absorbing atoms

For the spectra calculation:

*Par_a* → Contraction or expansion of the mesh parameter a in %
*Par_b* → Contraction or expansion of the mesh parameter b in %
*Par_c* → Contraction or expansion of the mesh parameter c in %
*Par_abc* → General contraction or expansion in %
*Par_anga* → Value of the unit mesh angle $\alpha$
*Par_angb* → Value of the unit mesh angle $\beta$
*Par_angc* → Value of the unit mesh angle $\gamma$
*Par_poporb* → Orbital occupancy
*Par_posx* → Atom position along x
*Par_posy* → Atom position along y
*Par_posz* → Atom position along z
*Par_occup* → occupancy of the atom
*Par_dposx* → shift of the atom position along x from the original position
*Par_dposy* → shift of the atom position along y from the original position
*Par_dposz* → shift of the atom position along z from the original position
*Par_theta* → position along $\theta$ for an atom in spherical coordinate
*Par_phi* → position along $\phi$ for an atom in spherical or cylindrical coordinate
*Par_v_helm* → Helmholtz potential
*Par_delta_hel* → Distance of the Helmholtz layer from the topmost atom layer
*Par_width_hel* → Width of the Helmholtz layer

Under each parameter must be written the first and last values of the parameter followed by the number of values.

For some parameters a $4^{th}$ index must be given specifying:

- the index of the atom in the case of *Par_posx*, *Par_posy*, *Par_posz*, *Par_theta*, *Par_phi*, *Par_occup*
- the index of orbital given under the keyword "*Atom*", or "*Atom_conf*" for *Par_poporb* and *Par_occup*
- the index of file (under “Calculation”) for *Par_shift* and *Par_weight*

For example, to specify an exchange of charge between two atoms (titanium and oxygen), one has to write:

***Atom***
*22 2 3 2 2. 4 0 2.* → Ti initial occupancy: $3d^2$-$4s^2$
*8 1 2 1 4.* → Oxygen initial occupancy: $2p^4$

***Parameter***
***Par_poporb***
*2. 0. 3 1* → The first orbital under *atom* (or *Atom_conf*) is the 3d titanium.
***Par_poporb***
*4. 6. 3 3* → The third orbital under *atom* (or *Atom_conf*) is the 2p oxygen.

By default the metric distances are calculated in all the energy range is the intersection between the experimental and calculated spectra. It is possible to cut the lower or and the higher energy part of the spectra by the use of the keyword:

***Emin***
*-10.* → Minimum energy for all the spectra

***Emax***
*100.* → Maximum energy for all the spectra

It is possible to have different values for the different spectra:

***Emin***
*-10. -5. -20. -20.* → Minimum energy for each spectra

***Emax***
*45. 100. 100. 100.* → Maximum energy for each spectra

If the energy of the experimental spectra is in keV and not in eV, put the keyword:

***Kev***

When there are several spectra to compare simultaneously, for the calculation they must be in the same output file. One then must give the number of the column contains the calculated spectra and associate it at the corresponding experimental file. This is done adding a line after each experimental file contains the number of the column. If there are 2 numbers, the first one is the number of the column in the experimental file, the second one being the number of the column in the calculated file. When there is no number, this means than in both experimental and calculated file, the spectra are in the second column, the first one being the energy. Example:

***Experiment*** → keyword
*Nom_exp_1.txt* → Name of the file containing the first experimental spectra
2 → Number of the column in the calculated file containing the corresponding spectra
*Nom_exp_2.txt* → Name of the file containing the second experimental spectra

3 → Number of the column in the calculated file containing the corresponding spectra
*Nom_exp_2.txt* → Name of the file containing the third experimental spectra
4 3 → Numbers of the column in the experimental file *Nom_exp_2.txt*, followed by the number of the column in the calculated file

By default, 2 confidence factors are used (D1 and RX). By default, D1 is used for searching the minimum in the space of parameters. If one wants it is Rx, use the keyword:

***Rx***

A third one "Rxg" can also be calculated. It is equivalent to Rx but with the spectra set in n group. In each group the normalization factor between theory and data is the same. When there is a single spectrum, Rxg is equivalent to Rx. To get this confidence factor put the keyword:

***Rxg***
1 2 3 ! index of the spectra with the same normalization factor
4 6 ! second set
5 ! this spectrum has a specific normalization factor

The indexes correspond to the order under "experiment".
When nothing is written under Rxg, there is a single normalization factor for all the spectra.

It is also possible to compare spectra without "fit". In that case we use the keyword "*File_met*". When followed by 2 file names, the comparison is done between the 2 set of spectra included in the files (they must have the same number of columns):

***File_met***
*File_1_conv* → name of file number 1
*File_2_conv* → name of file number 2

The keyword "*Gen_shift*" or "*Metric_out* "can be used with "*File_met*" as usual. This keyword can be used alone when both files are existing.

To compare one set of spectra with others in a series of files, we use "*File_met*" followed by a single file name. Its set of spectra is then compared with the ones given under the keyword "*Experiment*", with the column attribution as described in this keyword description. For example:

***File_met***
*File_conv* → File name with several columns, first energy, then the spectra

***Experiment***
*Nom_exp_1.txt* → Name of the file containing the 1$^{er}$ spectra
2 → Number of the column of the file *File_conv*
*Nom_exp_2.txt* → Name of the file containing the 2$^{ème}$ spectra
3 → Number of the column of the file *File_conv*
*Nom_exp_2.txt* → Name of the file containing the 3$^{ème}$ spectra
4 3 → Number of the column of *Nom_exp_2* then of *File_conv*

When the data are given with the errors in 3rd column, the R-factor Rx can be calculated in order the decrease the weight of the spectra with a large error. One uses $K_i = 1/\sum_i e_{i,j}^2$, where $e_{i,j}$ are the errors, $j$ index the energy points and $i$ the reflections. The weight is then given by $w_i = K_i/\sum_k K_k$ For this, use the keyword:

***Error***

# F- Extraction of DAFS scans and spectra

When realizing a DAFS simulation with azimuth dependence (scan), the output scan file after convolution contains the intensity of all the reflections at all energies and all azimuth angles. Often, it is useful to extract from this big file some spectra at specific azimuth angle or some scan at specific energy. It is what is done in this part with the following input file:

*! Input file for FDMNES, Selection part*

*Selec_inp* → keyword for the input file name (output of the convolution part)
*xanout/fe3o4/2008_bland/cc_1221_1221_orig_1m10_scan_conv.txt*

*Selec_out* → keyword for the output file name
*xanout/test/fe3o4_scan_selec_conv.txt*

*Energy* → keyword for the selected energy for the scan
*4.* → value of the energy for the scan in eV.

*Reflection* → keyword for the selected reflections
*2 5 6 9* → number of the selected reflections

*End*

The azimuth scan is extracted at the energy (in eV) given after keyword "*Energy*".
It is also possible to extract spectra at a given azimuth angle. For this, no keyword "*Energy*" but the keyword "*Azimuth*" and next line the value of the selected azimuth:
*Azimuth*
*30.* → Value of the azimuth

It is possible to have several reflections in the output file at one energy (or one azimuth). It is also possible to have several energies but only one reflection for the scan or several azimuth angles but one reflection for the spectra.

*Energy*
*4 7.5* → selected energy for the scan

*Reflection*
*2* → reflection number 2 selected

or:

*Reflection*
*2* → reflection number 2 selected

*Azimuth*
30. 60. 90. → Azimuth selected

Without the keyword « *Reflection* », all reflections are given when there is only one energy or one azimuth asked.

# G – Unit cell modification

FDMNES contains a tool to build the list of atoms of a unit cell superstructure from the atom list of a simple unit cell. This can be used as the starting list corresponding to a unit cell corresponding to a decrease in symmetry or to specific magnetic order.

***Filout*** → Output file in which is given the list of atom of the superstructure unit cell
*xanout/test_stand/mult_out*

***Mult_cell*** → Multiplication factor along a, b and c
*2 2 1* → Here one buids a (2a, 2b, c) unit cell

***Atomic_nu*** → Atomic number corresponding to the atom type under "*Unit_cell*"
*26 38 33*

***Unit_cell*** → to introduce the original unit cell
*3.92430 3.92430 12.36440 90. 90. 90.* → a, b, c, α, β, γ
*1 0.50000 0.00000 0.25000* → list of all the atoms of the unit cell
*1 0.00000 0.50000 0.25000*
*1 0.50000 0.00000 0.75000*
*1 0.00000 0.50000 0.75000*
*2 0.00000 0.00000 0.00000*
*2 0.50000 0.50000 0.50000*
*3 0.00000 0.00000 0.36000*
*3 0.00000 0.00000 0.64000*
*3 0.50000 0.50000 0.86000*
*3 0.50000 0.50000 0.14000*

The original unit cell is introduced with the keyword "*Unit_cell*" under which are given the unit cell parameters, and the list and the list of all the atoms in it. In first column is set the atomic number, but if using the optional keyword "*Atomic_nu*". In this case, first column contains the atom type number and the correspondence between atom type number and atomic number is given under "*Atomic_nu*".

To tranform a cubic unit cell in a hexagonal one with:
(1,0,-1) new x axis
(-1,1,0) new y axis,
(1,1,1) new z axis, use the keyword:

***Cub_hexa***

For the general transformation case, use:

***Mat_cul***
*1 0 -1* → new x axis
*-1 1 0* → new y axis
*1 1 1* → new z axis, with this matrix the transformation is equivalent to "*Cub_hexa*"

# H - FDMX User's guide

FDMX is an extension from J. Bourke and C. Chantler, University of Melbourne, Australia. When using it, thanks to cite:

Jay Daniel Bourke, Christopher Thomas Chantler and Yves Joly
"*Extended X-ray Absorption Fine Structure Calculations Using the Finite Difference Method*"
J. Synchrotron Rad. **23**, 551-559 (2016).

FDMX is an enhanced approach to calculating both XANES and XAFS spectra using the finite difference method and the core routines of FDMNES. The easiest way to use FDMX is to simply include the keyword ***fdmx*** in your regular input file, and the code will automatically optimize computational parameters to ensure accuracy over a wide energy range. FDMX will process the spectrum to include thermal effects and electron/hole lifetimes, and add background absorption from more loosely bound electrons. The code is currently designed for use with K-edge calculations only, however most functionality will also work with other edges (note keywords for hole widths and background absorption). Additional (optional) controls for FDMX are possible with specific keywords.

As calculations using FDMX may take several hours, particularly for structures with few or no axes of symmetry, it is strongly recommended that you compile and run the code using the MUMPS libraries.

***fdmx***

Activates FDMX computation and optimization of parameters with respect to energy, allowing for accurate EXAFS spectra, and triggers the processing routine at the end of the calculation. When this keyword is used, *the Radius keyword is no longer required.*

***fdmx_proc***

Use to activate a post-processing routine from FDMX, implementing thermal, inelastic scattering, background, and other effects without explicitly calculating the absorption spectra. This requires an existing output file with absorption cross sections from a previous calculation.

***E_cut***
4.0 ! Val

Similar to the keyword for ***convolution*** in FDMNES, this will suppress transitions below the nominated Fermi level, expressed in eV. Some absorption will still be possible below this level due to the hole width and IMFP effects, and background absorption from other edges. **Use of this keyword is strongly recommended.**

***Radius***
8.3 100. 7. 250. 5. 400. 4. ! *Val, En, Val ...*

Allows the user to specify how the cluster radius changes with energy. By default when using fdmx procedure, the radius is set (as above) to 8.3 Å for energies up to 100 eV, then 7 Å for energies up to 250 eV, then 5 Å etcetera. Using a high or constant radius at high energies may

result in very long calculations, while low radii will cause XANES and XAFS structure to be missing.

***adimp***
0.24 0. 100. 0.20 250. 0.16 400. 0.12 500. 0.08 ! *Val, En, Val ...*

Allows the user to specify how the inter-point distance (grid density) changes with energy. By default, the inter-point distance is set (as above) to 0.25 Å for energies up to 0 eV, then 0.22 Å for energies up to 10 eV, then 0.18 Å etcetera. Using a high or constant inter-point distance at high energies may produce inaccurate results, while low values will lead to long calculations.

***Gamma_hole***
2.3 ! *Val*

By default, FDMX will include a core-hole relaxation based on K-shell tabulations from Scofield and Kostroun (Z=21-50) and "B" (Z=51-100)? Alternatively one may provide an explicit core-hole lifetime (in eV) with the keyword ***Gamma_hole*** or suppress this effect by using the keyword:

***nohole***

It is required that you use either ***Gamma_hole*** or ***nohole*** for calculations involving edges other than K.

***IMFPin***
imfpdatafile.txt ! *Filename*

By default, FDMX will include photoelectron lifetime broadening based on the electron inelastic mean free path (IMFP) of the absorbing material. For elements the IMFP values are tabulated from optical data model calculations, while for compounds they are estimated using the TPP-2M equation. Users may instead include their own IMFP values with the ***IMFPin*** keyword followed by a filename that contains IMFPs in the format:

*En Val*

where *En* is in eV and *Val* is in Å. Alternatively, IMFPs can be calculated directly from optical energy loss data (i.e. optical ELFs), which may be obtained from band structure packages such as WIEN2k (in WIEN2k this data is obtainable using the *optic* routines and is output to a file named *case.eloss*). To use ELF data to compute the IMFP, use the keyword:

***ELFin***
elfdatafile.txt

where the data is formatted the same as for an IMFP input file, excepting that the values are unitless. The use of ***ELFin*** will overwrite other IMFP values up to the maximum energy of the ELF provided (or 120 eV, whichever is lower), after which default IMFP values will be used. By default, the IMFP is calculated from the ELF using a Lindhard (fast) representation of the

dielectric function. However, it is recommended that you use an additional keyword for a Mermin representation:

***Mermin***
1 ! Val

The number following the ***Mermin*** keyword specifies an iteration value, with 0 corresponding to a Lindhard representation, 1 to a standard Mermin representation (recommended), and higher values to successive iterations of a self-consistent Mermin model. This calculation may take some hours, and iteration values higher than 3 will not normally be useful for XAFS. For more information see Bourke & Chantler, *J. Phys. Chem. Lett.* **6** 314 (2015).

Use of the ***ELFin*** keyword will generate an additional output file with the extension _ELF.txt containing the energy- and momentum-dependent ELF of the system. Use of ***Mermin*** without ***ELFin*** will produce no effect.

***noIMFP***

Use to omit the effects of photoelectron lifetime, and hence represent an infinite IMFP.

***Tmeas***
298 ! Val

Use to define the temperature, in Kelvin, at which the absorption is being calculated. This value will affect the Debye-Waller Factor, and is set to 0 by default.

***TDebye***
345 ! Val

Use to define a Debye temperature (in Kelvin). This is strongly recommended for compound structures.

***DWfactor***
0.13 ! Val

Use to define an effective Debye-Waller factor (in Å). By default, this will otherwise be calculated based on the dominant oscillators contributing to the high energy XAFS spectrum.

***noDW***

Use to omit thermal effects – effectively a zero Debye-Waller factor.

***noBG***

By default, FDMX will include background absorption from more loosely bound electrons (i.e. L, M edges etc). Use this keyword to omit such contributions. Note that the background absorption currently only works properly for K-edge calculations, and therefore for other edges this keyword is required.

For comparison with experimental data, it is useful to define an extra function to address the edge jump discrepancy (triangle effect). This function comes in two flavors, and is implemented by the following relation:

$$\mu_0(E) \rightarrow \mu_0(E)(1 + f(E))$$

where $\mu_0$ is the atomic (or atom-like) background absorption.

***Victoreen***
0.8 0.4 ! Val A, Val B

Adds a background function from the Victoreen Equation with *A* and *B* parameters following:

$$f(E) = A\left(\frac{E_0^3}{E^3}\right) + B\left(\frac{E_0^4}{E^4}\right)$$

This applies to all energies above the absorption edge $E_0$. *A*+*B* provides the relative edge jump correction, while *A*/*B* controls the rate of decay.

***Expntl***
0.1 100. ! Val A, Val B

Adds a background function of an exponential form with *A* and *B* parameters following:

$$f(E) = Ae^{-\frac{(E-E_0)}{B}}$$

This applies to all energies above the absorption edge $E_0$. *A* provides the relative edge jump correction, while *B* controls the rate of decay.

***cm2g***

Output results in units of $cm^2/g$ instead of Mbarn.

# I - 2D Diffraction

FDMNES can simulate 2D – resonant diffraction experiments. The corresponding output are (*h,k,l*) peaks with non-integer indices. Truncation rods at a specific energy can be given as well as spectra at specific points of the truncation rods.

## *1) Surface structure description*

The surface is supposed perpendicular to the (*a,b*) plane, thus eventually, the unit cell must be especially written in this way. Note that *c* is not necessarily perpendicular to the surface. The surface to simulate must contain some atomic layers that we call "surface" and most often a substrate taken as semi-infinite crystal that we call "bulk".

The substrate is taken into account with the keyword:

***Bulk*** ! Structure of the substrate
*8.1724 8.1724 4.0862 90. 90. 90.* ! a, b, c, alpha, beta, gamma
*47 0.0 0.0 0.0* ! Z, x, y, z
*47 0.5 0.5 0.0*
*47 0.5 0.0 0.5*
*47 0.0 0.5 0.5*

On top of the bulk atoms, the surface atoms are set using keyword "*Surface*":

***Surface***
*8.1724 8.1724 4.0862 90. 90. 90. = a, b, c, alpha, beta, gamma*
*27 0.0 0.5 0.2* ! Z, x, y, z
…..

Note that the unit cell parameters are not necessary the same than the bulk unit cell parameters. The distances between the top most bulk atom and the bottom less atom of the surface is given by the lowest z value (in the 4$^{th}$ column). This can be change using keyword "Surface_shift". When using it, this distance is given under this keyword:

***Surface_shift***
*1.25* → distance between bottom most surface atom and top most bulk layer (A)

To give a shift in the 3 directions one writes:

***Surface_shift***
*0 0.23 1.25* → shifts along a and b and distance between bulk and surface (A)

When there is many surface atoms, it can be convenient to consider it as a film which is considered as "bulk-like" along some unit cell length. In such a film the atomic form factor are calculated as in a 3D material. For this purpose, one uses the keyword "*Film*":

***Film*** → Set a simulation on a 2D film, followed by the unit cell description
*8.1724 8.1724 4.0862 90. 90. 90. = a, b, c, alpha, beta, gamma*
*47 0.0 0.0 0.0* ! Z, x, y, z
….

By default the film thickness is equal to the '*c*' unit cell parameter, eventually multiplied by the cosines of the angle between c and the basal plane. One can use a specific thickness using the "*Film_thickness*" keyword, followed by its value in Angstrom not necessarily equal to a multiple of *c*.

***Film_thickness***
*75*

The interlayer distance between the topmost substrate layer and the bottom film layer is by default zero. This can (and must) be changed with the following keyword:

***Film_shift***
*3.5* → z when only one number is given (in Angström), z is perpendicular to the surface, direction outwards.

***Film_shift***
*0. 0. 3.5* → x, y, z, all in Angstöm, x and y are along ***a*** and ***b*** of the film.

***Film_shift***
*0. 0. 3.5 45.* ! x, y, z, angle

A fourth number can be added. It is the angle between the ***a*** film axis and the *a* bulk axis. Can be useful for epitaxial growth when the a film corresponds to the (***a***,***b***) diagonal of the bulk

By default, the bottom atom in the film is the one defined with the lowest z position under the keyword "*Crystal*". This can be changed defining a new zero, in reduced unit. The atoms between z = 0 and this value are just shifted by +1.

***Film_zero***
*0.25* → The bottom atom will be the next one with z >= 0.25. Then the stacking is as usual.

Note that the keyword "*Spgroup*" can be used for a film, but not for the description of "*Surface*" or "*Interface*" as seen bellow. It is possible to use only a film and a bulk. To improve the calculation it is possible to add some surface layer atoms on top of the film. For this purpose, we just use the same "*Surface*" keyword as before. Because there is a "film", the code understands that the "surface" is on top of the film and not on top of the bulk.

It is also possible to consider an interface between the bulk and the film. This is done with the same format than with the surface, film or bulk atoms but with the keyword:

***Interface***
*8.1724 8.1724 4.122 90. 90. 90. = a, b, c, alpha, beta, gamma*
*26 0.0 0.0 0.0*
….

The interface distance from the bulk is given in the same format than with the “surface” using the keyword:

***Inter_shift***
*1.26*

One can add a cap layer with:

***Cap_layer***
*2.8837228875 2.8837228875 7.0636496 90. 90. 120.*
*79 0. 0. 0. 1.*
*79 0.6666666667 0.3333333333 0.3333333333*
*79 0.3333333333 0.6666666667 0.6666666667*

***Cap_thickness***
*17.1*

A Debye disorder in Angström can be given using "*Cap_B_iso*" or "*Cap_U_iso*". This disorder is the same for all the atoms of the cap layer:

***Cap_B_iso***
*80.0* → $= 8\pi^2\langle u^2\rangle$ in $Å^2$

***Cap_U_iso***
*1.0* → $= \langle u^2\rangle$ in $Å^2$

By default the distance between film and cap is the sum of the atom radii. This can be changed with:

***Cap_shift***
*3.0*

The roughness of the « bulk » or the interface bulk/film, of the film or the interface film/cap layer and of the cap layer can be taken into account. They are described by the function $0.5\,\mathrm{erfc}\big(z - z0/\sqrt{2}R\big)$, where z0 is the origin taken at the middle in the inter-layer above the last atomic layer. This is done with :

***Bulk_roughness*** → roughness at the bulk surface or at the interface bulk/film
*1.5* → R

***Film_roughness*** → roughness at the film surface or at the interface film/cap layer
*4.*

***Cap_roughness*** → roughness at the cap layer surface
*17.1*

With the keywords "*Occupancy*" and/or "*Atom_B_iso*" (or "*Atom_U_iso*", "*Atom_U_ani*", "*Atom_B_ani*") another 1 or 2 other columns, in the $5^{th}$ and $6^{th}$ columns, can be

added for surface, film, inter-face and bulk slabs, containing the occupancy rate (between 0 and 1) and the Debye parameter, $8\pi^2\langle u^2\rangle$, in Å². The column order of these parameter corresponds to the order of these keywords in the input file.

***Atom_B_iso***

*47  0.0    0.0    0.0    0.82*                    ! Z, x, y, z
….

***Occupancy***
***Atom_B_iso***

*47  0.0    0.0    0.0    0.8    0.82*                    ! Z, x, y, z, Occupancy, Debye

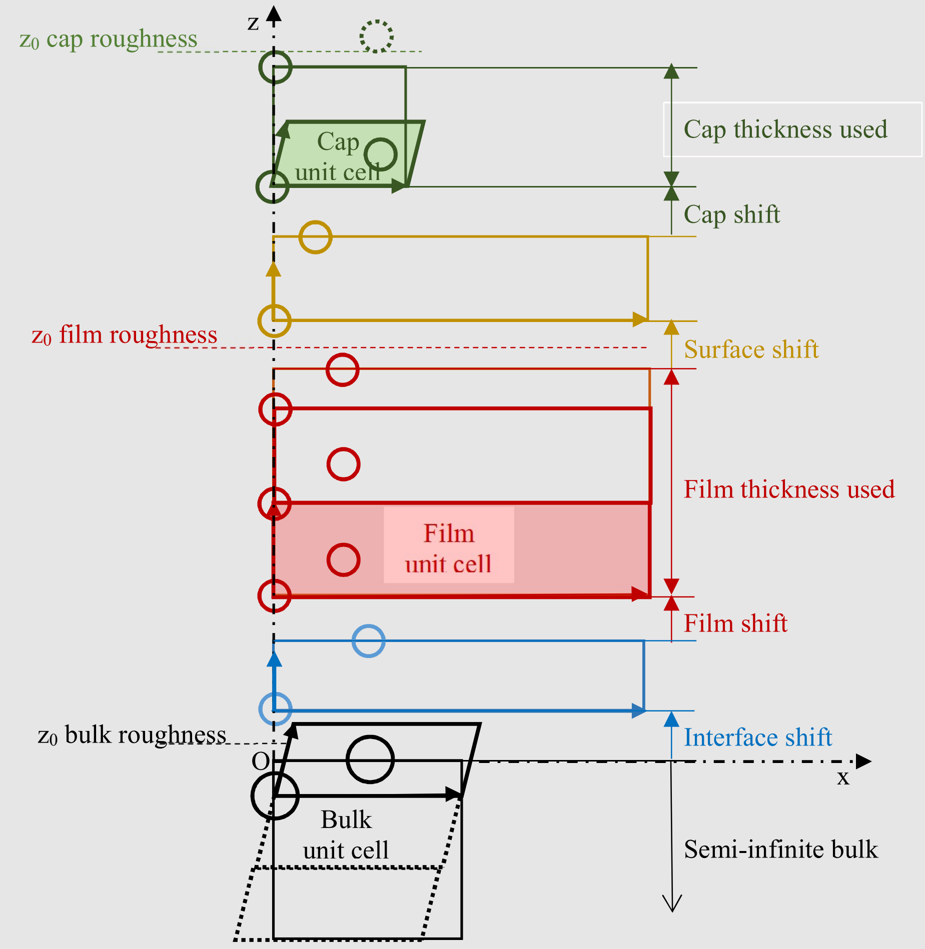


Fig. The most complete possible description with film, cap, interface, surface and semi-infinite bulk. All the elements are optional. For example only “surface” and “bulk” atoms can be given. Cap and Film thickness used = distance between topmost and bottommost atoms inside Film Thickness or Cap Thickness. The origin $z_0$ of the roughness function is at half of the interreticular distance on top of the considered slab.

## 2) Reflection choice

***DAFS***

*0 0 0.04 0.04 8.16 1 1 0.* ! h, k, $l\_min$, $l_{step}$, $l_{max}$, sigma, sigma, azimuth
*0 0 8.18 0.002 8.24 1 1 0.*
*0 0 8.28 0.04 10.00 1 1 0.*

There is thus a scan along the rod from $l_{min}$ of to $l_{max}$.
Note that *h* and *k* can be used as non-integer when the (*a,b*) film periodicity is rational (and not equal) to the bulk periodicity.

It can be more convenient to use the experimental operation mode as the one defined in "Spec". In that case use :

***DAFS_2D***

*0 0 0. 0.05 5. 4 1 4 0 0 0. 0.* ! h, k, $l\_min$, $l_{step}$, $l_{max}$, Operation mode, Angle
*0 0 0. 0.05 5. 4 1 4 0 0 90. 0.* ! h, k, $l\_min$, $l_{step}$, $l_{max}$, Operation mode, Angle
*0 1 0. 0.05 5. 2 1 2 0 0 30. 10.*
*0 0 1. 4 1 4 0 0 90. 0.* ! h, k, l, Operation mode, Angle

After h,k,l (with or without scan along a truncature rod), one gives the 5 integers of the operation mode of the same table than in "Spec" (and nearly corresponding to the You'th paper (J. Appl. Cryst, 32, 614 (1999)), then the values of the fixed angles :

| | Detector | Reference | Sample | Sample | Sample |
|---|---|---|---|---|---|
| 1 | delta | alfa = beta | eta | eta | Eta |
| 2 | nu | Alfa | mu | mu | Mu |
| 3 | Qaz | Beta | chi | chi | Chi |
| 4 | Naz | Psi | phi | phi | Phi |
| 5 | x | X | eta = delta / 2 | eta = delta / 2 | eta = delta / 2 |
| 6 | x | X | mu = nu / 2 | mu = nu / 2 | mu = nu / 2 |

delta = $\delta$: detector polar angle
nu = $\nu$: azimuthal detector angle (independent from mu)
Qaz : azimuth of the rotation of the diffraction vector / laboratory
Naz : azimuth of the rotation of the normal to the surface / laboratory. Naz = 0 when normal is horizontal. Specular case: polarizations $\sigma$ et $\pi$ → Naz = 90 and 0°.
alfa = $\alpha$: incidence angle
beta = $\beta$: outgoing angle
psi = $\psi$: azimuthal angle of the sample rotation around the diffraction vector. Its zero is when the reference vector is in the incidence plane in the positive direction of the incoming beam; diffraction vector is then horizontal. By default the reference vector (in reciprocal space) is (0,0,1). To change it, see keword "*Setaz*".
eta = $\eta$: polar angle of the sample
mu = $\mu$: azimuthal angle of the sample

chi = $\chi$: second angle of the sample
phi = $\phi$: azimuthal angle of the sample

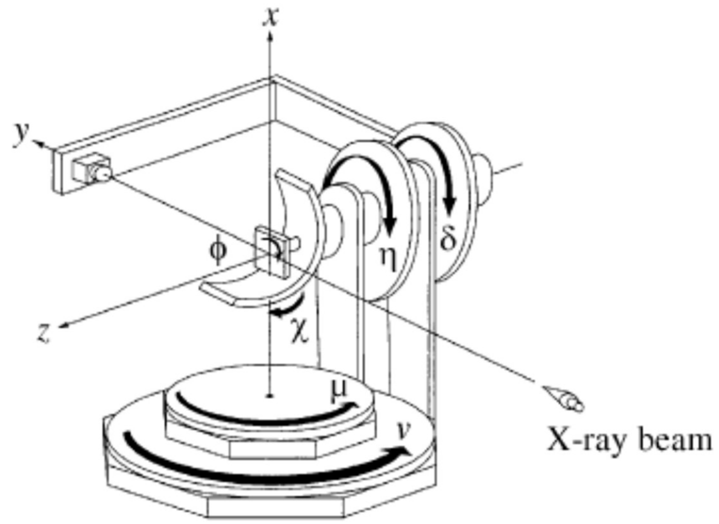


Figure from You's paper showing the angle definitions corresponding to a « 4+2 » diffractometer when all angles are 0

For example, the operation mode *4 1 4 0 0* means « Naz fixed, alfa=beta, phi fixed ».
The following angles are the values of the fixed angles. For example :
"*4 1 4 0 0  0. 0.*" means « Naz =0., alfa=beta, phi =0 ».
"*4 1 4 0 0  90. 0.*" means « Naz =0., alfa=beta, phi =90 ».
"*2 1 2 0 0 30. 10.* " means « nu =30., alfa=beta, mu =90 ».

Note that the "z-axis mode" used on 4-circles needs to take chi = eta = 0. The free angle can then be the incidence angle alpha, which is equal to mu. This gives: "*0 2 3 1 0 alpha 0. 0.* "

Some operation modes are not possible or not coded:

| | |
|---|---|
| 4 0 i j 0 | for any i and j |
| i 0 1 3 0 | for i = 1, 2 or 3 |
| i 0 1 4 0 | for i = 1, 2 or 3 |

For the specular reflection, one may only use :

| | |
|---|---|
| 4 1 4 0 0 | |
| 0 1 3 4 0 | with only chi = 0 or 90 |
| 0 1 4 3 0 | with only chi = 0 or 90 |

With the same keyword, it is possible to have circular polarization writing with a negative sign one of the 2 first number in the operation mode. For example:

***DAFS_2D***
*0 0 0. 0.05 5. -4 1 4 0 0 0. 0.* ! h, k, $\ell_{min}$, $\ell_{step}$, $\ell_{max}$, Operation mode, Angle

Then one obtains in the output file, 2 columns with respectively right and left incoming circular polarization.

When the experimental orientation matrix (U) times the (h,k,l)→Q matrix transformation (B) is known (the so call UB matrix), this one can be used:

***Mat_UB***
*-0.6713131898 -0.331076068 -0.005363453371*
*0.331087442 -0.6713288502 -0.0004569418448*
*-0.004608158701 -0.002782139698 0.7485135622*

By default, the reference axis from which the psi angle is defined is the normal to the surface. It is possible to define another direction, as in the experiment using:

***Setaz***
*1 1 0* → direction (in reciprocal space coordinate)

It can be interesting in the convolution part of the calculation to get not only the spectra but also the scan along the rod at specific energies. To get the corresponding new file (with extension "_tr"), write:

***Transpose_file***
*15. 17.*
The new file is in fact just a transpose of the standard output file, taken in the example at the energies 15 and 17 eV.

The film, surface, interface and bulk unit cell parameters can be all different. By default the (*h,k,l*) indices correspond to the bulk parameters. One can makes that it corresponds to the film ones with keyword:

***hkl_film***

Note that when film and unit cell parameters with components parallel to the surface not rational between them, film and bulk non-specular reflections do not interfere.

By default the code calculate the intensity for well-defined outgoing polarization. Using "*DAFS_2D*" keyword, one thus obtains output spectra for both σ and π outgoing conditions. To have only the sum of them, that is as being without analyzer, use the keyword:

***No_analyzer***

In SRXRD, it is possible to consider a Helmholtz layer. See the keyword "*Helmholtz*" in another section of this manual.

Because the bulk is often a dense simple metal, it can be faster to simplify the calculation in this part. It is possible to avoid the SCF calculation and/or the finite difference method. For this use:

***No_SCF_bul***k → no SCF calculation in the bulk, whatever it is SCF or not in the surface

***Green_bul***k → in the bulk, the calculation is done using the multiple scattering theory, whatever is the calculation mode in the surface.

***Bulk_atom_not_recalculated*** → With this option the potential of the bulk atoms inside the area of calculation of a surface atom are not recalculated. Their potential obtained at the bulk step is kept.

To simulate the effect of a plane of atoms, possibly charged, at a certain distance from the surface plane, as in the Helmholtz model, it is preferable to use the concept of counter-atoms, which allows, unlike the Helmholtz model seen above, to have a construction of the potential responding to a chemical reality. For this we use the keyword "*Counter_atom*", which allows to list a series of atoms positioned relative to the surface atoms, possibly having a certain charge, and therefore the potential is convolved by a Gaussian to simulate the positioning disorder:

***Counter_atom***
*1 2.5 8 0.5 0.1 1.* → index of referent atom (in the list under "*Surface*"), the counter-atom is just above it), distance from reference atom (2.5 Å), atomic number, Z = 8 is for oxygen, width of the gaussian distribution $\sigma$ = 0.5 Å, ch = 0.1, charge on the atom and occupancy (which is optional, default is 1).

It is possible to shift the counter atom position parallel to the surface versus the refence atom. For this add between the index of the referent atom and the distance, these displacements along the "**a**" and "**b**" unit cell parameters in Angström and not in reduced units.

***Counter_atom***
*1 0.1 0.15  2.5 8 0.5 0.1 1.* → reference atom, index, displacements along "**a**" and "**b**" in Angström, the distance between the reference atom plane and the counter atom plane in Angström …

An example with 2 counter atoms, to simulate Hydronium:

***Counter_atom***
*1  2.5 8 0.5 -2 0.5* → occupancy is to have half hydronium
*1  2.5 1 0.5  1  1.5*

It is generally interesting to associate the charge of the counter atom with an opposite charge of the reference atom by:

***Atom_conf*** → To have the reference atom with the opposite charge (see this keyword).
*1 1    1 5 2 8.1* ! For this occupancy, the corresponding Pt atomic charge is -0.1

***Surface***

*2.77482843 2.77482843 6.796913779 90.0 90.0 120.0*

*78 0.0000000000 0.0000000000 1.00489999999*

*78 0.3333333333 0.6666666667 0.66666666666*

*78 0.6666666667 0.3333333333 0.33333333333*

*78 0.0000000000 0.0000000000 0.0000000000*

It is possible to have one single SCF calculations for all the non-equivalent atoms from the surface down to the top most one in the bulk. Calculation in *Full_atom* mode is then forbidden. This step of the calculation is done in a cylinder extended from the top most surface down to the first bulk atom. The axis of the cylinder goes through this bulk atom. A center of symmetry is artificially set on this atom. The second part of the job, the calculation of the atomic resonant form factors is done as usual, calculated inside the spheres, each of them centered along the same cylinder axis but at the z position of the considered atoms. For this purpose, use the keyword:

**One_SCF_surf**

For surface in vacuum, to have the potential resulting from the charge image, use the keyword:

***Charge_image***

The exchange-correlation potential is then substituted by $V(z) = \frac{1}{z - z_0}$ (in Rydberg and bohr radius), with $z_0 = z_s + \frac{1}{2}R$, $z_s$ being the surface atomic plane position and $R$ the atomic radius of the surface atom.

# List of *fdmnes* keywords


| | |
|---|---|
| Abs_B_iso | 66 |
| Abs_U_iso | 66 |
| Abs_before | 62 |
| Absorber | 21, 45, 47 |
| Adimp | 55, 56, 82 |
| All_conv | 62 |
| All_nrixs | 27 |
| Allsite | 47 |
| Ang_spin | 35 |
| Atom | 19, 33, 34, 72 |
| Atom_B_ani | 32, 88 |
| Atom_B_iso | 32, 88 |
| Atom_conf | 20, 41 |
| Atom_U_ani | 32, 88 |
| Atom_U_iso | 32, 88 |
| Atom_nsph | 41 |
| Atomic_nu | 79 |
| Axe_spin | 35 |
| Azimuth | 77 |
| Basecomp | 53 |
| Bulk | 85 |
| Bulk_atom_not_recal | 92 |
| Bulk_roughness | 87 |
| Calculation | 61 |
| Cap_B_iso | 87 |
| Cap_layer | 87 |
| Cap_roughness | 87 |
| Cap_shift | 87 |
| Cap_thickness | 87 |
| Cap_U_iso | 87 |
| Cartesian | 46 |
| Center | 45 |
| Center_abs | 45 |
| Center_s | 45 |
| Charge_image | 93 |
| Check | 52 |
| Check_all | 52 |
| Check_biref | 69 |
| Check_coabs | 52 |
| Check_conv | 64 |
| Check_mat | 52 |
| Check_mpi | 53 |
| Check_pot | 52 |
| Check_sph | 52 |
| Check_tens | 52 |
| Chfree | 41 |
| Circular | 69 |
| Clementi | 40 |
| cm2g | 84 |
| Comment | 49 |
| Conv_gaus | 70 |
| Conv_out | 62 |
| Convolution | 61, 63 |
| COOP | 40 |
| COOP_dist | 40 |
| COOP_z_axis | 40 |
| Core_energy_tot | 50 |
| Core_resolved | 58 |
| Counter_atom | 93 |
| Crystal | 17, 31, 40 |
| Crystal_p | 19 |
| Cub_hexa | 79 |
| Cyl_pot | 50 |
| Cyl_SCF | 51 |
| Dafs | 28, 29, 31, 89 |
| DAFS_2D | 89 |
| Dafs_exp | 30 |
| Dafs_exp_type | 30 |
| Dead_layer | 68 |
| Density | 44 |
| Density_comp | 45 |
| Density_all | 45 |
| Dec | 63 |
| Delta_E_conv | 36 |
| Dilatorb | 42 |
| Dip_rel | 23 |
| Dipmag | 23 |
| Directory | 62 |
| Doping | 24 |
| Double_cor | 68 |
| Dpos | 46 |
| DWfactor | 83 |
| E_cut | 62 |
| E_moment | 41 |
| E_out_max | 53 |
| E_step_conv | 66 |
| E1E2 | 23 |
| E1E3 | 23 |
| E1M1 | 23 |
| E1M2 | 23 |
| E1SP | 23 |
| E2E2 | 23 |
| E2M2 | 23 |
| E3E3 | 23 |

| | | | |
|---|---|---|---|
| Ecent | 64 | Helm_cos | 38 |
| Edge | 22 | Helm_lin | 38 |
| Eimag | 42 | Helm_mix | 38 |
| Elarg | 64 | Helmholtz | 38 |
| Emax | 73 | hkl_film | 91 |
| Emin | 73 | Hubbard | 37 |
| End | 15 | Hubbard_Z | 37 |
| Endjump | 48 | IMFPin | 82 |
| Energpho | 22 | Interface | 86 |
| Energy | 77 | Inter_shift | 87 |
| Epsii | 47 | iord | 55 |
| Error | 74 | Jump | 48 |
| Estart | 65 | Kev | 73 |
| ELFin | 82 | Length_line | 58 |
| Excited | 55 | Lmax | 56 |
| Experiment | 71, 73 | Lmax_DOSout | 50 |
| Expntl | 84 | Lmax_nrixs | 27 |
| Extract | 25 | Lmax_tddft | 37 |
| File_in | 70 | Lmaxfree | 56 |
| File_out, Filout | 17, 70, 79 | Lmaxso | 14 |
| File_met | 74 | Lmaxso_max | 56 |
| Fdmx | 81 | Lminus1 | 43 |
| Fdmx_proc | 81 | Lplus1 | 43 |
| Film | 18, 86 | M1M1 | 23 |
| Film_Cif_file | 19 | Magnetism | 32, 47 |
| Film_Pdb_file | 19 | Mat_mul | 79 |
| Film_roughness | 87 | Mat_polar | 24 |
| Film_shift | 86 | Mat_UB | 91 |
| Film_thickness | 86 | Memory_save | 58 |
| Film_zero | 86 | Mermin | 82 |
| Flapw | 47 | Metric_out | 71 |
| Flapw_n | 48 | Molecule | 17, 38 |
| Flapw_n_p | 48 | Muffintin | 55 |
| Flapw_psi | 47 | Mult_cell | 79 |
| Forbidden | 67 | N_self | 36 |
| Fprime | 68 | No_analyzer | 91 |
| Fprime_atom | 68 | No_check | 50 |
| Full_atom | 50 | No_cluster_rot | 59 |
| Full_potential | 50 | No_E1E1 | 23 |
| Full_self_abs | 29 | No_E1E2 | 23 |
| Gamma_hole | 64 | No_E1E3 | 23 |
| Gamma_var | 64 | No_E2E2 | 23 |
| Gamma_max | 64 | No_Fermi | 35 |
| Gaussian | 64 | No_res_mag | 32 |
| Gen_shift | 73 | No_res_mom | 32 |
| Green | 22 | No_SCF_bulk | 91 |
| Green_bulk | 91 | noBG | 84 |
| Harm_tess | 44 | noDW | 83 |
| Header | 59 | noIMFP | 83 |
| Hedin | 40, 47 | Noncentre | 44 |

Nonexc 55
Nonrelat 33
Normaltau 55
Norman 57
Nrato 48
NRIXS 27
NRIXS_mono 27
Occupancy 19, 32, 88
Octupole 23
One_run 49
One_run_s 49
One_SCF_Surf 93
Overad 46
Overlap 57
P_self 36
P_self_max 36
Parameter 71
PBE96 39
Perdew 39
Polarize 23
Python 59
Quadrupole 23
R_self 36
Radius 17
Range 22
Rangel 22
Ray_max_dirac 59
Rcharge 58
Rcharge_Z 58
Reflection 75
Relativism 33
Rionic 58
Rmtg_Z 57
Rmt 55
Rmtv0 57
RPA 37
Rpotmax 43
Rx 74
Rxg 74
Rydberg 43
S0_2 65
Sample_thickness 66
Scan 67
Scan_conv 67
Scan_file 67
SCF 35
SCF_exc 36
SCF_mag_free 37
SCF_smooth 37
Screening 39
Seah 64
Selec_core 66
Selec_inp 77
Selec_out 77
Self_abs 30
Setaz 88
Spgroup 18
Sphere_all 46
Spherical 44
Spinorbit 32
SPSP 23
Step_azim 31
Stokes 69
Stokes_name 69
Supermuf 57
Surface 85
Surface_plane 68
Surface_shift 85
Sym 52
Symsite 53
Table 64
TDDFT 37
TDebye 83
Thomson 67
Tmeas 83
Trace 44
Transpose_file 91
Unit_cell 79
V0imp 42
Victoreen 84
Vmax 42
Weight_co 70
Xalpha 39
Xan_atom 49
XES 63
Z_absorber 21
Zero_azim 29
